# Manipulating nitration and stabilization to achieve high energy

**DOI:** 10.1126/sciadv.adk3754

**Published:** 2023-11-15

**Authors:** Jatinder Singh, Richard J. Staples, Jean’ne M. Shreeve

**Affiliations:** ^1^ Department of Chemistry, University of Idaho, Moscow, ID 83844-2343 USA.; ^2^ Department of Chemistry, Michigan State University, East Lansing, MI 48824, USA.

## Abstract

Nitro groups have played a central and decisive role in the development of the most powerful known energetic materials. Highly nitrated compounds are potential oxidizing agents, which could replace the environmentally hazardous used materials such as ammonium perchlorate. The scarcity of azole compounds with a large number of nitro groups is likely due to their inherent thermal instability and the limited number of ring sites available for bond formation. Now, the formation of the first azole molecule bonded to seven nitro groups, 4-nitro-3,5-bis(trinitromethyl)-1*H*-pyrazole (**4**), by the stepwise nitration of 3,5-dimethyl-1*H*-pyrazole is reported. Compound **4** exhibits exceptional physicochemical properties with a positive oxygen balance (OB_CO2_ = 13.62%) and an extremely high calculated density (2.04 g cm^−3^ at 100 K). This is impressively high for a C, H, N, O compound. This work is a giant step forward to highly nitrated and dense azoles and will accelerate further exploration in this challenging field.

## Teaser

Design and construction of small molecules with seven energetic nitro groups encourages chemists to think outside the box.

## INTRODUCTION

**Fig. 1.**
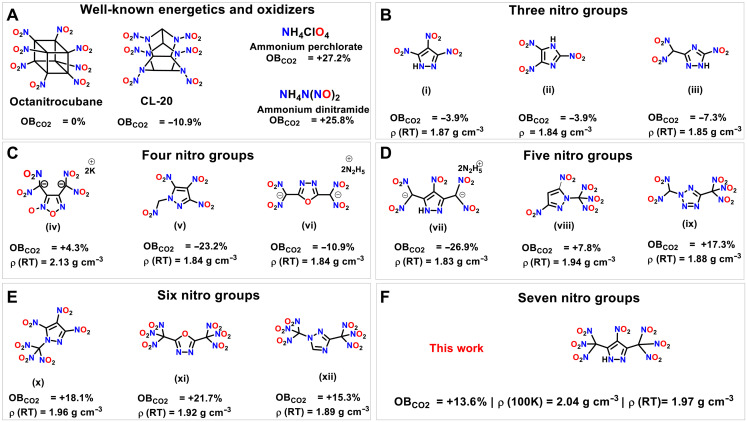
Known energetic oxidizers and current work. (**A**) Structures of well-known energetic oxidizers. (**B** to **E**) Representative examples of azoles containing polynitro groups. (**F**) This work.

The development and investigation of non-nuclear energetic materials are an exciting and challenging area of chemistry with application in the fields of defense and aerospace industries (*1*, *2*). Highly nitrated acyclic and cyclic hydrocarbons, in which organic backbones act as fuels and nitro groups act as oxidizers, are widely used products for energetic applications, and their efficient preparation remains an important goal (Fig. 1A) (*3*). The extension of highly nitrated materials as oxidizers in propellant formulations to replace ammonium perchlorate (AP) remains an important and challenging task from both environmental and economical perspectives in both academia and industry (*4*, *5*). The direct design and isolation of compounds that combine high density, positive oxygen balance (OB), and high energy without compromising chemical and thermal stability is challenging. Therefore, the expansion of different strategies for stepwise construction of highly nitrated organic frameworks has become an increasingly important research area in material chemistry.

In recent years, the azole class of energetic materials has been the richest source of high-energy density materials discovery, leading to different strategies for the construction of explosives, propellants, and pyrotechnics that are used for a variety of military purposes and civilian applications (*6*–*12*). Azole-rich energetic compounds have important thermal stability and encompass a very wide range of possibilities for substituent introduction, which offers the potential for productive application on which researchers can focus (*13*–*16*). Among azoles, the pyrazole ring has the advantage of being available for functionalization at the C3, C4, C5, and N1 positions (*17*). In 2021, we reported pyrazole-based energetic salts containing five nitro groups through introduction at C3, C4, and C5 positions (Fig. 1, compound vii) (*18*). Very recently, Zhang *et al.* (*19*) reported a pyrazole compound with six nitro groups via functionalization at the C3, C4, C5, and N1 positions. (Fig. 1, compound x). Until now, six is the maximum number of nitro groups reported bonded to an azole moiety (Fig. 1E). The paucity of azole derivatives with more than six nitro groups is likely due to their thermal instability and the limited number of ring sites available for bond formation. For instance, the maximum number of nitro groups found bonded to a two carbon oxadiazole ring is six (Fig. 1, compound xi) (*7*, *20*). In our continuing efforts to introduce as many nitro groups as possible associated with azoles, 4-nitro-3,5-bis(trinitromethyl)-1*H*-pyrazole (**4**) (Fig. 1F), which contains seven nitro groups, was obtained by the stepwise nitration of 3,5-dimethyl-1*H*-pyrazole.

## RESULTS

### Synthesis

**Fig. 2.**
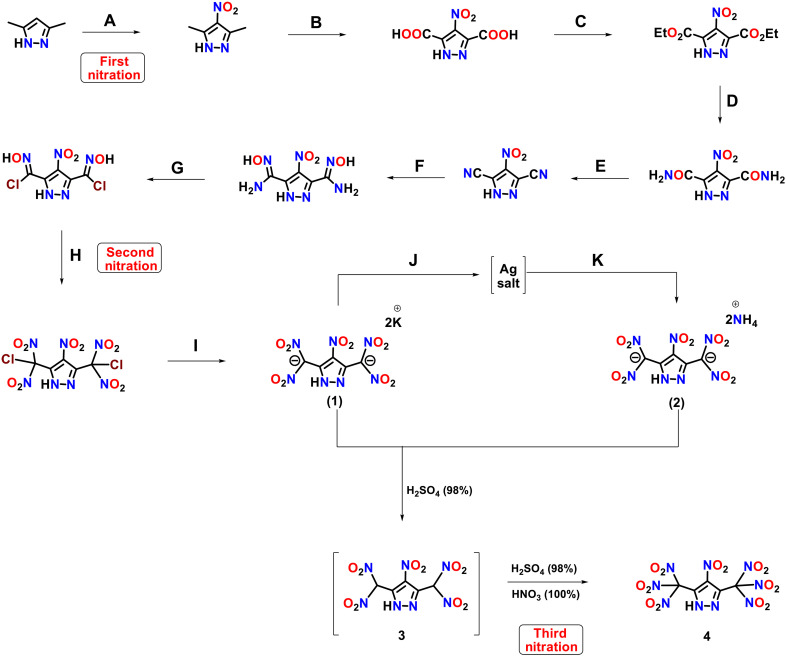
Route to 4-nitro-3,5-bis(trinitromethyl)-1*H*-pyrazole (4) (an azole with seven nitro groups). Reaction conditions: (**A**) H_2_SO_4_/KNO_3_, 110°C; (**B**) KMnO_4_/H_2_O; (**C**) H_2_SO_4_, EtOH, 80°C; (**D**) NH_3_(g), CH_3_OH, 80°C; (**E**) POCl_3_, CH_3_CN, 80°C; (**F**) NH_2_OH (50% in H_2_O), EtOH; (**G**) NaNO_2_/HCl, H_2_O; (**H**) TFAA/HNO_3_, 0°C; (**I**) KI, MeOH; (**J**) AgNO_3_, H_2_O; (**K**) NH_4_Cl, H_2_O.

The effort to obtain compound **4** began with the classical nitration (first nitration) of 3,5-dimethyl-1*H*-pyrazole at the 4-position to form 3,5-dimethyl-4-nitro-1*H*-pyrazole. Dipotassium 3,5-bis(dinitromethyl)-4-nitro-1*H*-pyrazole (**1**), which has five nitro groups, was obtained by the reaction of a chloroxime derivative with trifluoroacetic anhydride (TFAA)/HNO_3_ (second nitration), followed by the reaction with KI in methanol (Fig. 2). Diammonium 3,5-*bis*(dinitromethyl)-4-nitro-1*H*-pyrazole (**2**) is obtained from **1** by reaction with AgNO_3_ and NH_4_Cl, respectively (*18*). Either compound **1** or **2** is converted to **4** by the reaction with mixed acid, H_2_SO_4_ (98%) and HNO_3_ (100%), (third nitration) in 1:2 ratio (Fig. 2). The reaction goes through the formation of a neutral unstable intermediate **3**, which can be isolated and characterized (see the Supplementary Materials). At 0°C, a white solid (**4**) precipitates from the reaction mixture. It is removed by filtration and washed with trifluoracetic acid (TFA).

### Structure features of compound 4

**Fig. 3.**
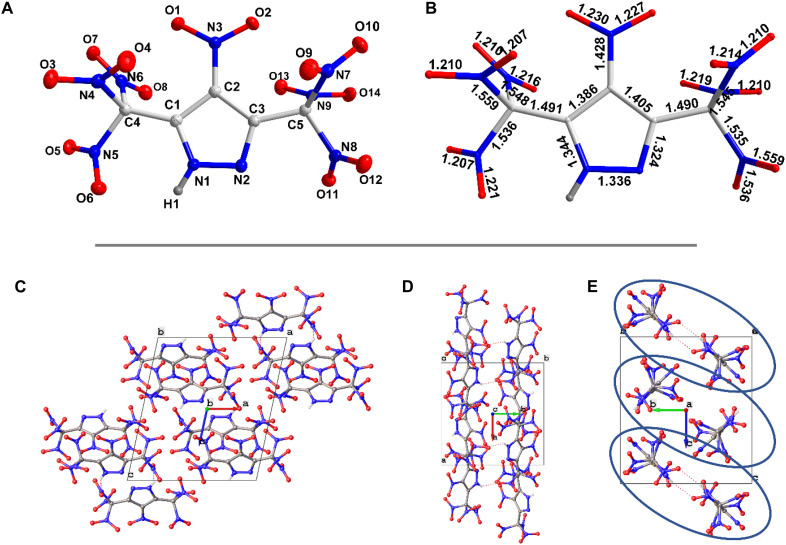
Crystal structure details for 4. (**A**) Thermal ellipsoid (50%) plot and tagging scheme for **4**. (**B**) Bond lengths in **4**. (**C** to **E**) Packing diagram for **4**.

Crystals suitable for single-crystal x-ray diffraction analysis (SC-XRD) for compound **4** were obtained from TFA. A colorless plate-shaped crystal with dimensions 0.12 mm by 0.08 mm by 0.02 mm was mounted on a nylon loop with Paratone oil. Data were collected using a XtaLAB Synergy, Dualflex, HyPix diffractometer equipped with an Oxford Cryosystems low-temperature device, operating at *T* = 99.9 (4) K. Crystallographic data are provided in the Supplementary Materials. Compound **4** crystallizes in the monoclinic space group *P*2_1_*/n* (Fig. 3A). The C2-N3 bond length in the C─NO_2_ group bonded to the pyrazole ring (1.428 Å) is much shorter than, for example, the analogous C4-N4 in the C─NO_2_ bond of the trinitromethyl group (1.535 to 1.559 Å) (Fig. 3B). Compound **4** has a calculated density of 2.039 g cm^−3^ at 100 K. The SC-XRD data for **4** was also collected at 298 K. A suitable crystal with dimensions 0.22 mm by 0.07 by 0.05 mm was selected and mounted on a nylon loop with Paratone oil on a XtaLAB Synergy, Dualflex, HyPix diffractometer. The crystal was kept at a steady *T* = 298 (10) K during the data collection. Compound **4** has a calculated density of 1.973 g cm^−3^ at 298 K (see the Supplementary Materials).

**Fig. 4.**
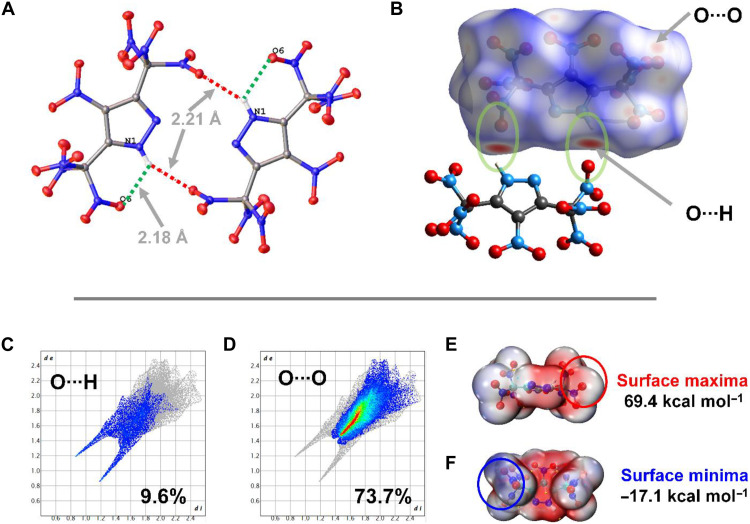
Understanding the physical properties and structure of 4. (**A**) Inter- and intramolecular interactions in **4**. (**B**) Hirshfield surfaces. (**C** and **D**) 2D fingerprint plots. (**E** and **F**) ESP.

The unusually high crystal density of **4** can be attributed to the presence of 14 oxygen atoms contained in the seven nitro groups. The unit cell has four molecules (Fig. 3, C to E). The packing index is calculated to be 76.3%. In the structure of **4**, H-bond interactions are observed. The packing diagram has two pyrazole molecules in the dimeric form with H-bonds and the O···O contacts on the periphery (Fig. 3E). As shown in Fig. 4A, the dimeric pyrazole moieties are stabilized by two intermolecular hydrogen bonds of length 2.21 Å. Also observed is the formation of an intramolecular H-bond (2.18 Å) between the –NH and –NO_2_ of the trinitromethyl group (Fig. 4a). To better understand the relationship between molecular structure and physical characteristics, Hirshfeld surfaces and two-dimensional (2D) fingerprints were examined using CrystalExplorer 21.5 (Fig. 4, B to D) (*21*, *22*). The red regions on the Hirshfeld surface represent interactions between neighboring molecules. Red spots located near the ring –NH group indicate strong intermolecular interactions, whereas the red spots on the periphery correspond to O···O contacts (Fig. 4B). Two spikes were observed in the 2D fingerprint plot, which represent O···H/H···O hydrogen bond interactions (Fig. 4C). The total calculated population of O···O interactions is 73.7% (Fig. 4D). The impact sensitivity of a material is vastly related to its electrostatic surface potentials (ESPs). The ESP of **4** was calculated using B3LYP/6-311+ G(d,p) level of theory and plotted with Multiwfn and VMD software (*23*). The maximum ESP is 69.4 kcal mol^−1^, and the minimum ESP is −17.1 kcal mol^−1^ (Fig. 4, E and F). Strong positive potential areas result in high impact sensitivity for compound **4**.

The nuclear magnetic resonance (NMR) spectrum of compound **4** was recorded in acetone-d_6_. In contrast with the ^1^H NMR of compound **1** in DMSO-d6, the resonance for the NH proton [13.7 parts per million (ppm)] (proton-locked) is not observed for compound **4**. Because of proton locking (localization of NH), five ^13^C NMR signals (123.2, 126.3, 128.0, 134.4, and 140.4 ppm) are observed for **1** (*18*, *24*), whereas for compound **4**, only three signals at 131.2, 133.8, and 155.4 ppm are present. The chemical environments of different nitro groups were determined using ^14^N NMR. A broad peak assigned to the NO_2_ group directly attached to the pyrazole ring was observed at δ −26.2 ppm. The trinitromethyl groups were seen at δ −31.4 to −34.5 ppm, and the pyrazole ring nitrogens (N1 and N2) were assigned at δ −71.7 ppm. The ^14^N NMR spectrum of the intermediate **3** shows a broad peak of five nitro groups at δ −22.2 ppm. High-resolution mass spectrometry (HRMS) also confirmed the formation of **4**. The high-resolution mass spectrum contained a characteristic peak at mass/charge ratio (*m*/*z*) = 409.9550, relative to its expected mass of 409.9565 [M − H] (see the Supplementary Materials).

### Physicochemical properties

The amount of oxygen in the molecule compared with the additional oxygen needed to oxidize all carbons to CO (OB_CO_) or CO_2_ (OB_CO2_) is expressed as OB. Compound **4** exhibits a positive OB (OB_CO_ = 33.08% and OB_CO2_ = 13.62%; Table 1), which makes it a potential oxidizer. The density measured using a gas pycnometer at 25°C is 1.971 g cm^−3^. To investigate the energetic properties of compound **4**, the enthalpy of formation (**Δ*H***_***f***_) was computed by using the method of isodesmic reactions with the Gaussian 03 suite of program (*25*) (see the Supplementary Materials). The calculated **Δ*H***_***f***_ for compound **4** is 25.7 kJ mol^−1^ (Table 1).

**Table 1.** Properties of 4. Physicochemical properties of **4** for comparison with (x), (xi), RDX, AP, and ADN.

	OB*(%)	ρ†(g cm^−3^)	Δ*H*_*f*_ ‡(kJmol^−1^)	*P*§(GPa)	*D*_*v*_||(m s^−1^)	IS¶(J)	FS#(N)	*T*_d_**(°C)
**4**	+13.6	1.97††/2.04‡‡	25.7	34.8	8745	5	80	131
(x)§§	+18.2	1.96||||	190.2	33.8	8602	7	120	160¶¶
(xi)##	+21.7	1.92	29.4	29.8	8191	4	240	102
RDX***	−21.6	1.80	92.6	34.2	8836	7.5	120	204
AP†††	+34.0	1.95	−295.8	18.4	6858	15	>360	>200
ADN‡‡‡	+25.8	1.81	−135.0	30.3	8495	4	64	159

*Oxygen balance (CO_2_).

†Density (experimental), 25°C.

‡Molar enthalpy of formation.

§Detonation pressure (calculated using EXPLO5 v7.01.01).

||Detonation velocity (calculated using EXPLO5 v7.01.01).

¶Sensitivity to impact (IS).

#Sensitivity to friction (FS).

**Temperature (onset) of decomposition (5°C/min).

††Crystal density (calculated), 298 K.

‡‡Crystal density (calculated), 100 K.

§§(*19*, *26*).

||||Density (calculated), 296 K.

¶¶Temperature (onset) of decomposition (10°C/min).

##(*7*, *26*).

***(*26*–*28*).

†††(*26*, *27*).

‡‡‡(*26*, *27*).

With the room temperature density and calculated **Δ*H***_***f***_ in hand, the detonation performance of **4** was calculated using EXPLO5 version 7.01.01 (*26*). The detonation velocity (calculated) is 8745 m s^−1^, and the detonation pressure (calculated) is 34.8 GPa. The detonation velocity and detonation pressure of compound **4** are comparable to 1,3,5-Trinitro-1,3,5-triazinane (RDX) (8836 m s^−1^, 34.2 GPa) (Table 1). The sensitivity to impact (IS) and sensitivity to friction (FS) for **4** were measured by using BAM standard methods, and it was found to be sensitive toward impact and friction (IS, 5 J; FS, 80 N) (Table 1). Specific impulse (*I*_SP_) of **4**, AP, and ammonium dinitramide (ADN) was calculated using EXPLO5 v7.01.01 software at chamber pressure of 7 MPa, expansion pressure ratio (Pc/Pe) of 70, initial *T* of 3500 K, ambient pressure of 0.1 MPa at the equilibrium, and expansion through the nozzle. The *I*_SP_ value for compound **4** is 244 s, which is higher than AP (157 s) and ADN (206 s). The differential scanning calorimetric (DSC) scans of **4** show two exothermic peaks upon heating at 5° and 10°C min^−1^. The thermal decomposition of **4** occurs at 131° (onset) and at 159°C (peak) in the DSC.

**Fig. 5.**
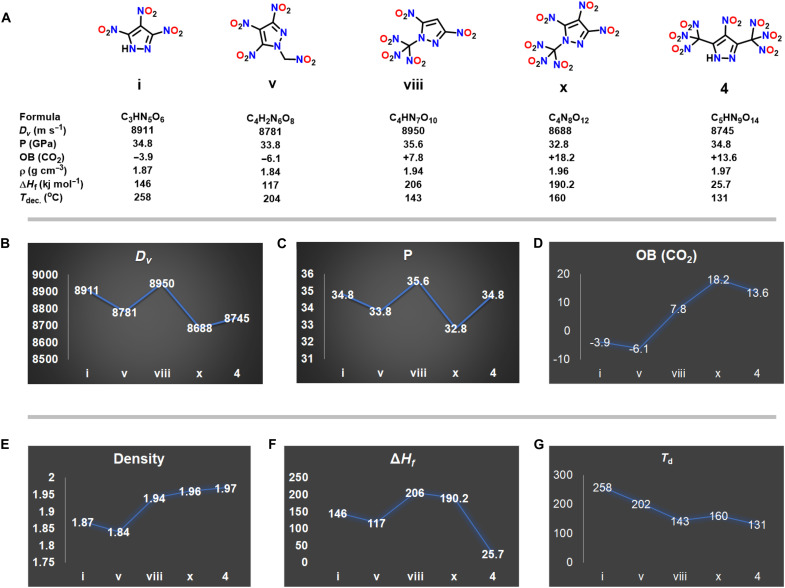
Comparison of structure and properties of pyrazoles. (**A**) Structure and properties of nitrated pyrazole compounds. Trends in (**B**) detonation velocity, (**C**) detonation pressure, (**D**) OB, (**E**) density, (**F**) enthalpy of formation, and (**G**) decomposition temperature.

A comparison of physiochemical properties of nitrated pyrazole compounds is given in Fig. 5A. The graphs of varying properties of pyrazole compounds containing three to seven nitro groups are shown in Fig. 5 (B to G). The detonation velocity and detonation pressure of compound **4** and the reported pyrazole derivatives (**i**, **v**, **viii**, and **x**) (*6*, *10*, *17*, *19*) containing three to six nitro groups are calculated using EXPLO5 v7.01.01 (Fig. 5, B and C). Compounds **4, viii**, and **x** have positive OB (OB_CO2_). The OB of compound (**x**) is higher than compound **4** because of a smaller carbon content (Fig. 5D). The densities of compounds increase with the increase in the number of oxygen atoms. Therefore, the high density of compound **4** i**s** attributed to the presence of 14 oxygen atoms present in the seven nitro groups, and it is higher than the previously reported pyrazole derivatives containing three to six nitro groups (Fig. 5E). Compound (**viii**) has the highest enthalpy of formation (206 kJ mol^−1^), detonation velocity (8950 m s^−1^), and detonation pressure (35.6 GPa) (Fig. 5F). The decomposition temperature found for compound **4** is the lowest (131°C) (Fig. 5G).

## DISCUSSION

The stepwise introduction of –NO_2_, –CH(NO_2_)_2_, or –C(NO_2_)_3_ on a pyrazole molecule leads to the first example of a highly nitrated azole bonded to seven nitro groups. The calculated density for **4** based on single-crystal x-ray analysis is 2.04 g cm^−3^ at 100 K, which is impressively high for a C, H, N, O compound. It also has a positive OB (OB_CO_ = 33.08%, OB_CO2 =_ 13.62%), which may give rise to a potential oxidizer comparable to AP and ADN. Its detonation properties (*D_v_* = 8745 m s^−1^, *P* = 34.8 GPa) are comparable to those of the traditional benchmark explosive RDX (*D_v_* = 8836 m s^−1^, *P* = 34.2 GPa). Its density is superior to previously reported nitrated pyrazole derivatives. Our future efforts are directed toward applying the insights gained from this study to other azole functionalization reactions.

## MATERIALS AND METHODS

### Safety precautions

Nitro compounds are powerful explosives and should be handled with extreme care using the best safety practices. All compounds should be synthesized in milligram amounts. Proper safety precautions such as leather gloves, face shield, and eye protection must be observed at all times when synthesizing and handling these materials.

### General methods

All reagents (analytical grade) were purchased from AK Scientific, VWR, or Oakwood chemicals and were used as supplied. ^1^H, ^13^C, and ^14^N NMR spectra were recorded using a 500-MHz (Bruker Advance) NMR spectrometer operating at 500.19, 125.77, and 36.15 MHz, respectively. Chemical shifts in the ^1^H and ^13^C NMR spectra are reported relative to Me_4_Si and ^14^N NMR spectra to MeNO_2_ as an external standard. The decomposition temperatures (onset) were obtained on a differential scanning calorimeter (TA Instruments Company, Q2000). Infrared spectra were recorded on a Fourier transform infrared spectrometer (Thermo Nicolet AVATAR 370) as thin films using KBr plates. The room temperature densities were measured at 25°C by using a gas pycnometer (Micromeritics AccuPyc II 1340). The impact and friction sensitivities were determined by using a standard BAM drop hammer and BAM friction tester. For MS, a Waters Q-Tof Premier quadrupole time-of-flight mass spectrometer was used.

Dipotassium 3,5-bis(dinitromethyl)-4-nitro-1*H*-pyrazole (**1**) and diammonium 3,5-bis(dinitromethyl)-4-nitro-1*H*-pyrazole (**2**) were obtained by following the literature method (*18*). Intermediate **3** is chemically unstable and decomposes in dried form. It can be isolated and characterized with NMR analysis (see the Supplementary Materials).

### Synthesis of compound 4

To the mixture of concentrated sulfuric acid (98%, 2 ml) and red fuming nitric acid (freshly distilled) (*d* = 1.5 g cm^−3^) (1.0 ml, 23.8 mmol), stirred at 0°C, was added **1** (0.10 g, 2.58 mmol) in small portions, with stirring continued at the same temperature for 3 hour. The resulting off-white precipitate was filtered, washed with small amounts of TFA (3 × 1 ml), and recrystallized from TFA. The crystalline material is found to be chemically stable and nonhygroscopic at room temperature. Physical description: Crystalline white solid. Yield: (0.05 g, 49%); *T*_d_ = 131°C (onset); ^13^C NMR (125 MHz, ppm, DMSO–d_6_): 131.2, 133.8, and 155.4; infrared (ν, cm^−1^): 1727, 1635, 1623, 1589, 1529, 1476, 1362, 1293, 1256, 1219; HRMS (ESI) *m*/*z*: [M − H] + calcd for C_5_HN_9_O_14_ 409.9565 [M − H]+; found: 409.9550.

## Supplementary Materials

### This PDF file includes:

Sections S1 to S3 Figs. S1 to S17 Tables S1 to S12 Legends for data S1 and S2 References
