# Supplementary material for: Manipulating nitration and stabilization to achieve high energy
Source: Sci Adv. 2023 Nov 15;9(46):eadk3754. doi: 10.1126/sciadv.adk3754 (PMC10651134; doi:10.1126/sciadv.adk3754)
Supplement: Supplementary file 1 — Sections S1 to S3 Figs. S1 to S17 Tables S1 to S12 Legends for data S1 and S2 References [file sciadv.adk3754_sm.pdf]

Science Advances
AAAS

Supplementary Materials for

Manipulating nitration and stabilization to achieve high energy

Jatinder Singh *et al.*

Corresponding author: Jean'ne M. Shreeve, jshreeve@uidaho.edu

Sci. Adv. **9**, eadk3754 (2023)
DOI: 10.1126/sciadv.adk3754

The PDF file includes:

Sections S1 to S3
Figs. S1 to S17
Tables S1 to S12
Legends for data S1 and S2
References

Other Supplementary Material for this manuscript includes the following:

Data S1 and S2

Section S1. X-ray crystallography details and crystallographic data

Section S1.1. Data collection

Data collection at 100K : A colorless plate-shaped crystal with dimensions 0.12×0.08×0.02 mm^3 was mounted on a nylon loop with Paratone oil. Data were collected using a XtaLAB Synergy, Dualflex, HyPix diffractometer equipped with an Oxford Cryosystems low-temperature device, operating at T = 99.9(4) K.

Data collection at 298K: A suitable crystal with dimensions 0.22 × 0.07 × 0.05 mm^3 was selected and mounted on a nylon loop with Paratone oil on a XtaLAB Synergy, Dualflex, HyPix diffractometer. The crystal was kept at a steady *T* = 298 (10) K during data collection.

The structures were solved with the ShelXT (*29*) solution program using dual methods and by using Olex2. (*30*) The model was refined with ShelXL (*31*) using full matrix least squares minimization on F^2. The thermal ellipsoid and packing diagrams of X-ray structures in the main article and supplementary material are plotted using Diamond 3.2 software.

Section S1.2. Crystal structures and crystallographic data.

Crystal structure compound **4** (Data collected at 100 K) (CCDC # 2262452):

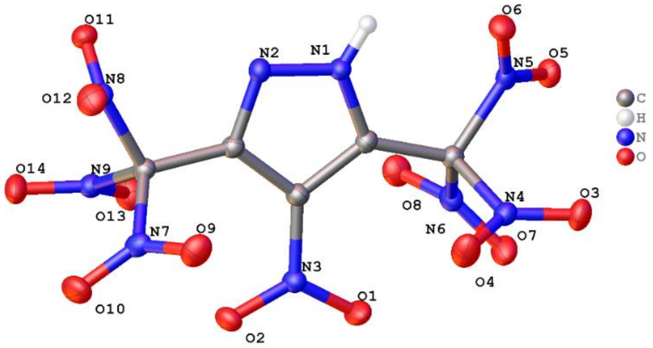

Fig. S1. Thermal ellipsoid plot for compound **4 (Data collected at 100 K)**. Thermal ellipsoids are drawn at 50% probability level.

Crystal structures of compound **4** (Data collected at 298 K) (CCDC # 2298841):

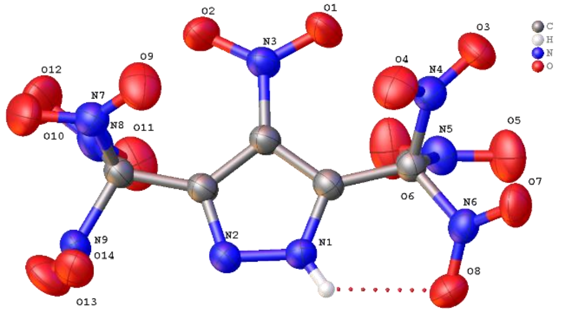

Fig. S2. Thermal ellipsoid plot for compound **4 (Data collected at 298 K)**. Thermal ellipsoids are drawn at 50% probability level.

Table S1. Crystallographic data for compound **4**.

Compound	4 (100K)	4 (298K)
CCDC #	2262452	2298841
Formula	$C_5HN_9O_{14}$	$C_5HN_9O_{14}$
$D_{calc.}$/ g cm^{-3}	2.039	1.973
m/mm^{-1}	1.870	1.810
Formula Weight	411.15	411.15
Colour	colourless	colourless
Shape	plate-shaped	needle-shaped
Size/mm^3	0.12×0.08×0.02	0.22×0.07×0.05
T/K	99.9(4)	298.00(10)
Crystal System	monoclinic	monoclinic
Space Group	$P2_1/n$	$P2_1/n$
a/Å	10.7133(2)	10.8108(6)
b/Å	10.5771(2)	10.7147(6)
c/Å	12.0923(2)	12.2728(9)
α/°	90	90
β/°	102.182(2)	103.218(6)
γ/°	90	90
V/Å^3	1339.39(4)	1383.95(15)
Z	4	4
Z'	1	1
Wavelength/Å	1.54184	1.54184
Radiation type	Cu K_a	Cu K_a
Q_{min}/°	5.017	4.924
Q_{max}/°	79.846	80.019
Measured Refl's.	10226	13956
Indep't Refl's	2872	2960
Refl's I≥2 s(I)	2600	2565
R_{int}	0.0316	0.0500
Parameters	257	257
Restraints	0	0
Largest Peak	0.333	0.283
Deepest Hole	-0.313	-0.235
GooF	1.075	1.086
wR_2 (all data)	0.0862	0.1243
wR_2	0.0836	0.1172
R_1 (all data)	0.0363	0.0477
R_1	0.0327	0.0427

Table S2. Bond Lengths in Å for **4** at 100K.

Atom	Atom	Length/Å
O1	N3	1.2304(16)
O2	N3	1.2266(16)
O3	N4	1.2095(16)
O4	N4	1.2068(17)
O5	N5	1.2072(16)
O6	N5	1.2211(16)
O7	N6	1.2101(17)
O8	N6	1.2159(17)
O9	N7	1.2144(18)
O10	N7	1.2103(17)
O11	N8	1.2183(17)
O12	N8	1.2091(16)
O13	N9	1.2190(16)
O14	N9	1.2101(16)
N1	N2	1.3357(16)
N1	C1	1.3443(18)
N2	C3	1.3240(19)
N3	C2	1.4278(18)
N4	C4	1.5594(18)
N5	C4	1.5356(17)
N6	C4	1.5480(18)
N7	C5	1.5454(18)
N8	C5	1.5351(17)
N9	C5	1.5330(18)
C1	C2	1.3856(19)
C1	C4	1.4911(18)
C2	C3	1.4049(18)
C3	C5	1.4896(18)

Table S3. Bond Lengths in Å for **4** at 298K.

Atom	Atom	Length/Å
O1	N3	1.2211(19)
O2	N3	1.217(2)
O3	N4	1.209(2)
O4	N4	1.208(2)
O5	N5	1.200(2)
O6	N5	1.195(2)
O7	N6	1.200(2)
O8	N6	1.213(2)
O9	N7	1.205(2)
O10	N7	1.206(2)
O11	N8	1.203(3)
O12	N8	1.201(3)
O13	N9	1.199(2)
O14	N9	1.210(2)
N1	N2	1.3333(19)
N1	C1	1.344(2)
N2	C3	1.317(2)
N3	C2	1.425(2)
N4	C4	1.536(2)
N5	C4	1.560(2)
N6	C4	1.534(2)
N7	C5	1.527(2)
N8	C5	1.540(2)
N9	C5	1.530(2)
C1	C2	1.381(2)
C1	C4	1.492(2)
C2	C3	1.407(2)
C3	C5	1.491(2)

Table S4. Torsion Angles in ° for **4** at 100K.

Atom	Atom	Atom	Atom	Angle/°
O1	N3	C2	C1	-3.7(2)
O1	N3	C2	C3	-179.73(14)
O2	N3	C2	C1	175.61(14)
O2	N3	C2	C3	-0.4(2)
O3	N4	C4	N5	-41.48(14)
O3	N4	C4	N6	69.97(14)
O3	N4	C4	C1	-165.90(12)
O4	N4	C4	N5	139.09(12)
O4	N4	C4	N6	-109.46(13)
O4	N4	C4	C1	14.67(17)
O5	N5	C4	N4	98.05(13)
O5	N5	C4	N6	-16.04(15)
O5	N5	C4	C1	-139.03(12)
O6	N5	C4	N4	-79.17(13)
O6	N5	C4	N6	166.74(11)
O6	N5	C4	C1	43.75(16)
O7	N6	C4	N4	-18.51(16)
O7	N6	C4	N5	92.63(14)
O7	N6	C4	C1	-142.76(13)
O8	N6	C4	N4	163.73(11)
O8	N6	C4	N5	-85.13(13)
O8	N6	C4	C1	39.48(16)
O9	N7	C5	N8	-88.78(13)
O9	N7	C5	N9	157.31(11)
O9	N7	C5	C3	30.50(15)
O10	N7	C5	N8	90.19(14)
O10	N7	C5	N9	-23.73(15)
O10	N7	C5	C3	-150.53(12)
O11	N8	C5	N7	-163.65(11)
O11	N8	C5	N9	-46.96(14)
O11	N8	C5	C3	75.16(14)
O12	N8	C5	N7	17.88(16)
O12	N8	C5	N9	134.57(12)
O12	N8	C5	C3	-103.31(14)
O13	N9	C5	N7	-113.31(13)
O13	N9	C5	N8	131.82(12)
O13	N9	C5	C3	12.64(17)
O14	N9	C5	N7	67.09(15)
O14	N9	C5	N8	-47.77(15)
O14	N9	C5	C3	-166.96(12)
N1	N2	C3	C2	-0.04(15)
N1	N2	C3	C5	-179.81(12)
N1	C1	C2	N3	-175.58(13)
N1	C1	C2	C3	1.12(15)
N1	C1	C4	N4	105.63(15)
N1	C1	C4	N5	-13.02(19)
N1	C1	C4	N6	-132.13(14)
N2	N1	C1	C2	-1.25(16)
N2	N1	C1	C4	179.24(12)
N2	C3	C5	N7	-121.62(13)
N2	C3	C5	N8	-3.52(17)
N2	C3	C5	N9	113.66(14)
N3	C2	C3	N2	176.01(13)
N3	C2	C3	C5	-4.3(2)
C1	N1	N2	C3	0.82(16)
C1	C2	C3	N2	-0.69(16)
C1	C2	C3	C5	179.04(14)
C2	C1	C4	N4	-73.77(18)
C2	C1	C4	N5	167.59(14)
C2	C1	C4	N6	48.5(2)
C2	C3	C5	N7	58.67(19)
C2	C3	C5	N8	176.77(14)
C2	C3	C5	N9	-66.05(19)
C4	C1	C2	N3	3.9(2)
C4	C1	C2	C3	-179.40(14)

Table S5. Torsion Angles in ° for **4** at 298K.

**Atom**	**Atom**	**Atom**	**Atom**	**Angle/°**
O1	N3	C2	C1	-3.1(3)
O1	N3	C2	C3	-179.53(18)
O2	N3	C2	C1	176.45(17)
O2	N3	C2	C3	0.0(3)
O3	N4	C4	N5	-17.5(2)
O3	N4	C4	N6	93.21(18)
O3	N4	C4	C1	-142.15(16)
O4	N4	C4	N5	163.95(15)
O4	N4	C4	N6	-85.35(17)
O4	N4	C4	C1	39.3(2)
O5	N5	C4	N4	70.6(2)
O5	N5	C4	N6	-40.6(2)
O5	N5	C4	C1	-164.84(17)
O6	N5	C4	N4	-109.4(2)
O6	N5	C4	N6	139.44(19)
O6	N5	C4	C1	15.2(2)
O7	N6	C4	N4	-14.45(19)
O7	N6	C4	N5	99.65(17)
O7	N6	C4	C1	-137.53(16)
O8	N6	C4	N4	167.99(14)
O8	N6	C4	N5	-77.91(17)
O8	N6	C4	C1	44.9(2)
O9	N7	C5	N8	-113.48(18)
O9	N7	C5	N9	131.98(17)
O9	N7	C5	C3	12.5(2)
O10	N7	C5	N8	66.6(2)
O10	N7	C5	N9	-47.9(2)
O10	N7	C5	C3	-167.41(17)
O11	N8	C5	N7	156.66(16)
O11	N8	C5	N9	-89.83(18)
O11	N8	C5	C3	29.8(2)
O12	N8	C5	N7	-23.9(2)
O12	N8	C5	N9	89.61(19)
O12	N8	C5	C3	-150.80(17)
O13	N9	C5	N7	134.26(17)
O13	N9	C5	N8	17.7(2)
O13	N9	C5	C3	-103.49(18)
O14	N9	C5	N7	-47.05(18)
O14	N9	C5	N8	-163.56(16)
O14	N9	C5	C3	75.21(19)
N1	N2	C3	C2	-0.10(19)
N1	N2	C3	C5	-179.36(15)
N1	C1	C2	N3	-176.16(17)
N1	C1	C2	C3	0.90(18)
N1	C1	C4	N4	-132.06(16)
N1	C1	C4	N5	105.23(18)
N1	C1	C4	N6	-13.0(2)
N2	N1	C1	C2	-1.05(19)
N2	N1	C1	C4	179.56(15)
N2	C3	C5	N7	112.85(17)
N2	C3	C5	N8	-122.36(17)
N2	C3	C5	N9	-4.3(2)
N3	C2	C3	N2	176.56(17)
N3	C2	C3	C5	-4.3(3)
C1	N1	N2	C3	0.7(2)
C1	C2	C3	N2	-0.5(2)
C1	C2	C3	C5	178.63(17)
C2	C1	C4	N4	48.7(2)
C2	C1	C4	N5	-74.0(2)
C2	C1	C4	N6	167.77(17)
C2	C3	C5	N7	-66.2(2)
C2	C3	C5	N8	58.5(2)
C2	C3	C5	N9	176.65(17)
C4	C1	C2	N3	3.2(3)
C4	C1	C2	C3	-179.76(16)

Table S6. Bond Angles in ° for **4** at 100K.

Atom	Atom	Atom	Angle/°
N2	N1	C1	113.59(12)
C3	N2	N1	105.18(11)
O1	N3	C2	117.92(11)
O2	N3	O1	124.53(12)
O2	N3	C2	117.54(11)
O3	N4	C4	114.76(11)
O4	N4	O3	129.67(13)
O4	N4	C4	115.57(11)
O5	N5	O6	128.02(12)
O5	N5	C4	116.86(11)
O6	N5	C4	115.05(11)
O7	N6	O8	128.41(13)
O7	N6	C4	117.55(12)
O8	N6	C4	114.00(11)
O9	N7	C5	113.83(12)
O10	N7	O9	128.60(13)
O10	N7	C5	117.56(12)
O11	N8	C5	114.14(11)
O12	N8	O11	128.45(12)
O12	N8	C5	117.39(12)
O13	N9	C5	116.11(11)
O14	N9	O13	127.63(13)
O14	N9	C5	116.26(11)
N1	C1	C2	105.16(12)
N1	C1	C4	124.78(12)
C2	C1	C4	130.06(13)
C1	C2	N3	127.37(12)
C1	C2	C3	105.47(12)
C3	C2	N3	127.07(12)
N2	C3	C2	110.60(12)
N2	C3	C5	118.82(12)
C2	C3	C5	130.58(13)
N5	C4	N4	104.40(10)
N5	C4	N6	104.87(10)
N6	C4	N4	108.56(10)
C1	C4	N4	112.11(11)
C1	C4	N5	114.43(11)
C1	C4	N6	111.90(11)
N8	C5	N7	106.97(10)
N9	C5	N7	109.64(11)
N9	C5	N8	105.49(10)
C3	C5	N7	111.94(11)
C3	C5	N8	108.96(11)
C3	C5	N9	113.44(11)

Table S7. Bond Angles in ° for **4** at 298K.

Atom	Atom	Atom	Angle/°
N2	N1	C1	113.52(14)
C3	N2	N1	105.30(13)
O1	N3	C2	117.84(14)
O2	N3	O1	124.42(15)
O2	N3	C2	117.73(14)
O3	N4	C4	117.51(16)
O4	N4	O3	128.36(18)
O4	N4	C4	114.11(15)
O5	N5	C4	115.03(15)
O6	N5	O5	129.62(17)
O6	N5	C4	115.36(15)
O7	N6	O8	127.59(16)
O7	N6	C4	117.46(15)
O8	N6	C4	114.90(14)
O9	N7	O10	126.95(19)
O9	N7	C5	116.26(15)
O10	N7	C5	116.78(16)
O11	N8	C5	114.04(17)
O12	N8	O11	128.25(19)
O12	N8	C5	117.70(19)
O13	N9	O14	128.19(17)
O13	N9	C5	117.10(16)
O14	N9	C5	114.70(15)
N1	C1	C2	105.15(14)
N1	C1	C4	124.35(15)
C2	C1	C4	130.50(15)
C1	C2	N3	127.48(14)
C1	C2	C3	105.40(14)
C3	C2	N3	127.05(14)
N2	C3	C2	110.62(14)
N2	C3	C5	118.72(14)
C2	C3	C5	130.66(14)
N4	C4	N5	108.76(13)
N6	C4	N4	104.74(13)
N6	C4	N5	103.99(13)
C1	C4	N4	112.03(13)
C1	C4	N5	112.24(13)
C1	C4	N6	114.46(13)
N7	C5	N8	109.67(14)
N7	C5	N9	105.20(13)
N9	C5	N8	106.81(13)
C3	C5	N7	113.53(13)
C3	C5	N8	111.89(14)
C3	C5	N9	109.32(13)

Table S8. Hydrogen bond information for **4** at 100K.

D	H	A	d(D-H)/Å	d(H-A)/Å	d(D-A)/Å	D-H-A/deg
N1	H1	O6	0.84(2)	2.179(19)	2.6814(15)	118.5(16)
N1	H1	O111	0.84(2)	2.21(2)	2.9551(16)	147.7(17)

Table S9. Hydrogen bond information for **4** at 298K.

D	H	A	d(D-H)/Å	d(H-A)/Å	d(D-A)/Å	D-H-A/deg
N1	H1	O6	0.84(2)	2.179(19)	2.6814(15)	118.5(16)
N1	H1	O111	0.84(2)	2.21(2)	2.9551(16)	147.7(17)

Section S2. Enthalpy of formation

Section S2.1. Isodesmic reactions

The ΔHf (Enthalpy of formation) of compound **4** was calculated by using isodesmic reactions (Fig. S3.).

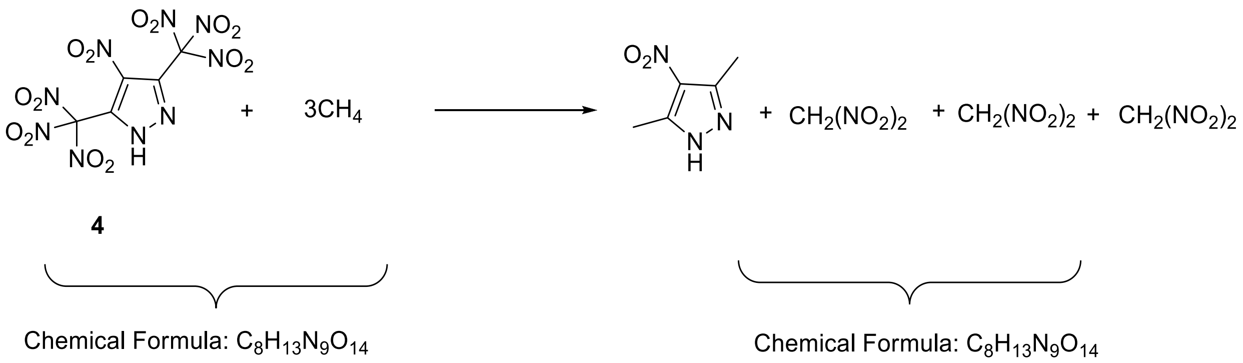

Fig. S3. Isodesmic reactions for compound **4**.

The single crystal structure was used for the geometric optimization and frequency analyses using the B3LYP functional with the 6-31+G** basis set. The single-point energies were obtained at the MP2/6-311++G** level (Table S8) (*32*).

Table S10. Calculated zero point energy (ZPE), values of the correction (Hr), total energy (E0) and gas-state $\Delta\mathbf{H_f}$.

Compound	ZPE	H_{corr}	scaled ZPE	ΔH_T	Mp-6-311++g**	Corrected	ΔH_r	ΔH_r (kJ/mol)	ΔH_f (kJ/mol)
4	0.141263	0.166606	0.13561	0.025343	-1732.683161	-1732.52221	-0.00394578	-10.3596444	101.6322705

The atomization energies for cations were calculated by using the G^2*ab initio* method (*33*) All of the optimized structures were characterized to be true local energy minima on the potential energy surface without imaginary frequencies.

Section S2.2. Gas-phase enthalpy of formation to solid state enthalpy of formation

The gas state $\Delta\mathbf{H_f}$ of compound **4** is 101.6 kJ mol^{-1}.

Method A. The calculated gas-phase enthalpy for compound **4** is converted to solid phase values by subtracting the empirical heat of sublimation obtained based on Trouton's rule (*34*, *35*).

ΔH_{sub} (kJ mol^{-1}) = 0.188·(273+131) = 75.9 kJ mol^{-1}

Method B. ΔH_{sub} (kJ mol^{-1}) = 0.15×T_m(K) + 3.27×[H] + 5.30×[N] + 3.30×[O]. Where, Tm is the melting temperature, and [H], [N] and [O] are the number of hydrogen, nitrogen and oxygen atoms present in the molecule, respectively (*36*).

ΔH_{sub} (kJ mol^{-1}) = 0.15×T_m(K) + 3.27×[H] + 5.30×[N] + 3.30×[O].
ΔH_{sub} (kJ mol^{-1}) = 60.6+3.27+47.7+46.2 = 157.8 kJ mol^{-1}

Table S11. Solid state $\Delta\mathbf{H_f}$ for compound **4** using Trouton's rule.

ΔH_f (g) kJ mol^{-1}	ΔH_{sub} kJ mol^{-1}	ΔH_f (s) kJ mol^{-1}
101.6	75.9	25.7

Section S2.3. Cartesian coordinates and computational details

Table S12. Cartesian coordinates (in Å) for optimized structure of compound **4**.

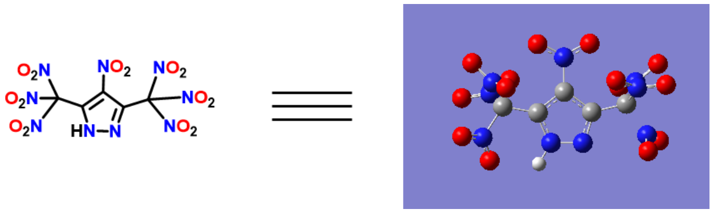

MP2 = - -1732.6831613

Number of imaginary frequencies (NImag) = 0

```
Zero-point correction=                           0.141263 (Hartree/Particle)
Thermal correction to Energy=                    0.165662
Thermal correction to Enthalpy=                  0.166606
Thermal correction to Gibbs Free Energy=         0.085301
Sum of electronic and zero-point Energies=          -1736.125560
Sum of electronic and thermal Energies=             -1736.101161
Sum of electronic and thermal Enthalpies=           -1736.100217
Sum of electronic and thermal Free Energies=        -1736.181522
```

Center Number	Atomic Number	Atomic Type	Coordinates (Angstroms) X	Y	Z
1	6	0	-1.159337	-0.710054	-0.144951
2	6	0	0.064318	0.182216	-0.082002
3	6	0	1.111216	-0.631018	-0.209149
4	7	0	0.627794	-1.981452	-0.575650
5	1	0	1.106468	-2.699020	-0.069717
6	6	0	-2.621174	-0.235624	-0.047238
7	6	0	2.584179	-0.233365	0.000259
8	7	0	0.090343	1.640743	0.099443
9	8	0	-0.969783	2.269317	0.212060
10	8	0	1.172379	2.240110	0.139584
11	7	0	-2.732837	1.123759	-0.595424
12	8	0	-1.968885	1.492223	-1.496683
13	8	0	-3.590797	1.899767	-0.155687
14	7	0	-3.042086	-0.232100	1.361208
15	8	0	-3.892150	0.578530	1.750983
16	8	0	-2.546390	-1.039763	2.157211
17	7	0	-3.483990	-1.145667	-0.814227
18	8	0	-3.709118	-0.923377	-2.010704
19	8	0	-3.985270	-2.134125	-0.263481
20	7	0	2.764120	1.182681	-0.350929
21	8	0	2.620830	2.057457	0.512719
22	8	0	3.058904	1.500082	-1.510245
23	7	0	3.442503	-1.063689	-0.856913
24	8	0	3.719964	-0.688927	-2.003309
25	8	0	3.887669	-2.137503	-0.432175
26	7	0	2.951925	-0.439511	1.408509
27	8	0	2.398621	-1.328012	2.068868
28	8	0	3.814836	0.275435	1.933762
29	7	0	-0.770363	-1.945282	-0.283878

Section S3. Spectrum Analysis

^{1}H, ^{13}C NMR, ^{14}N NMR, mass, IR, DSC of compounds in this study are provided in this section.

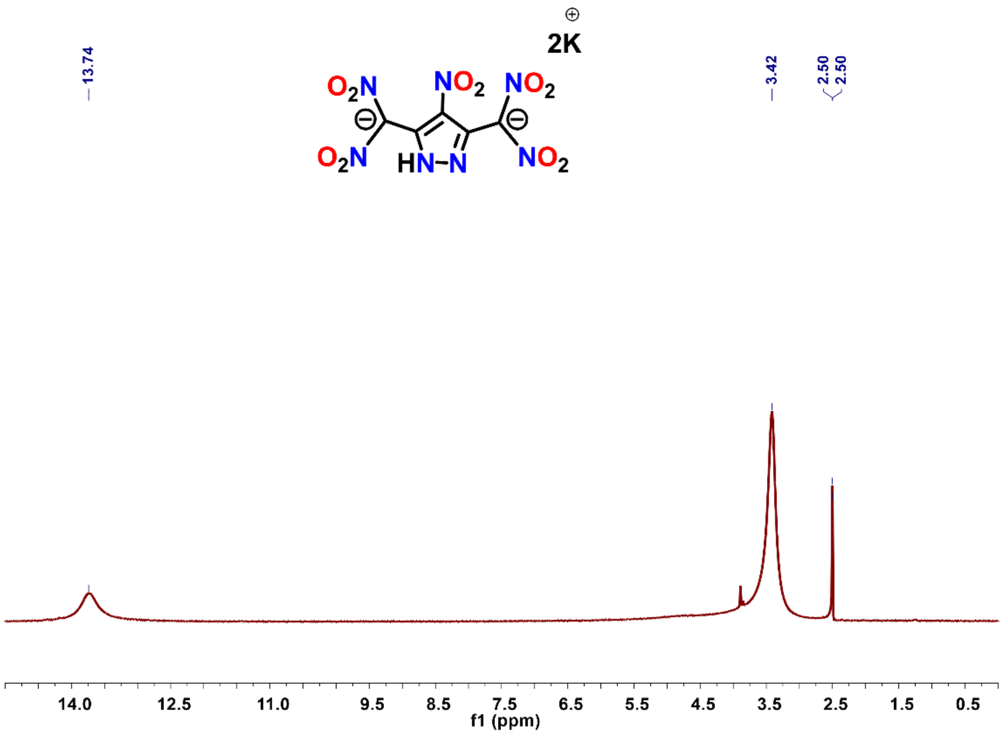

Fig. S4. ^{1}H NMR spectrum for compound **1**.

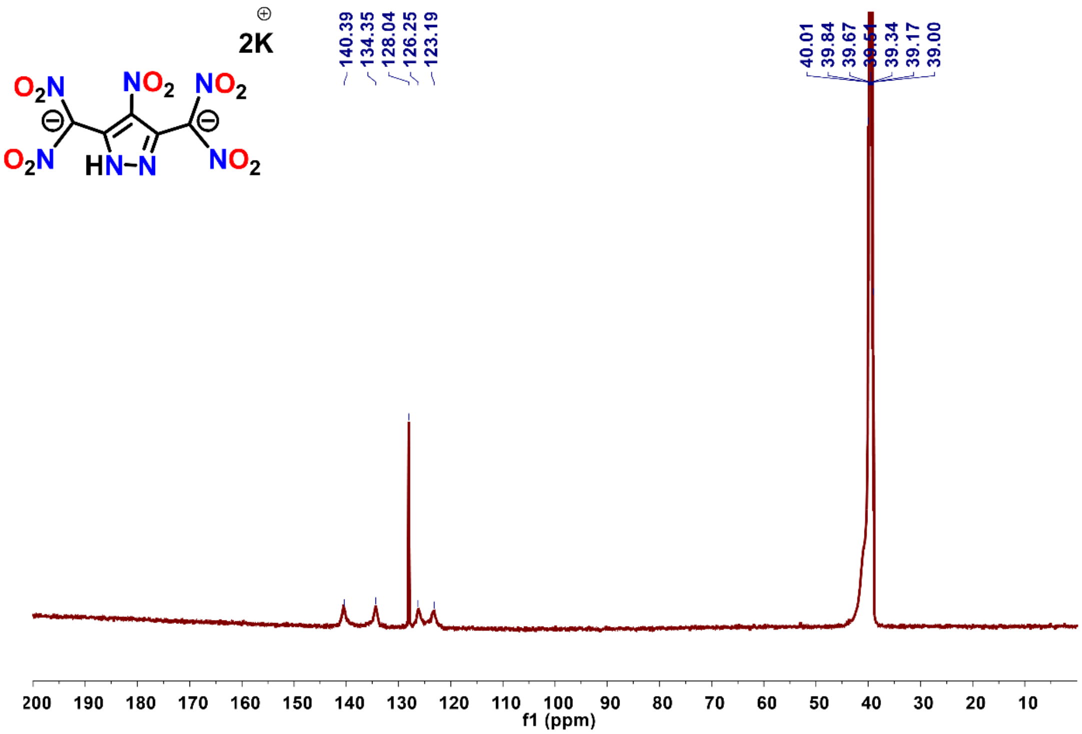

Fig. S5. ^{13}C NMR spectrum for compound **1**.

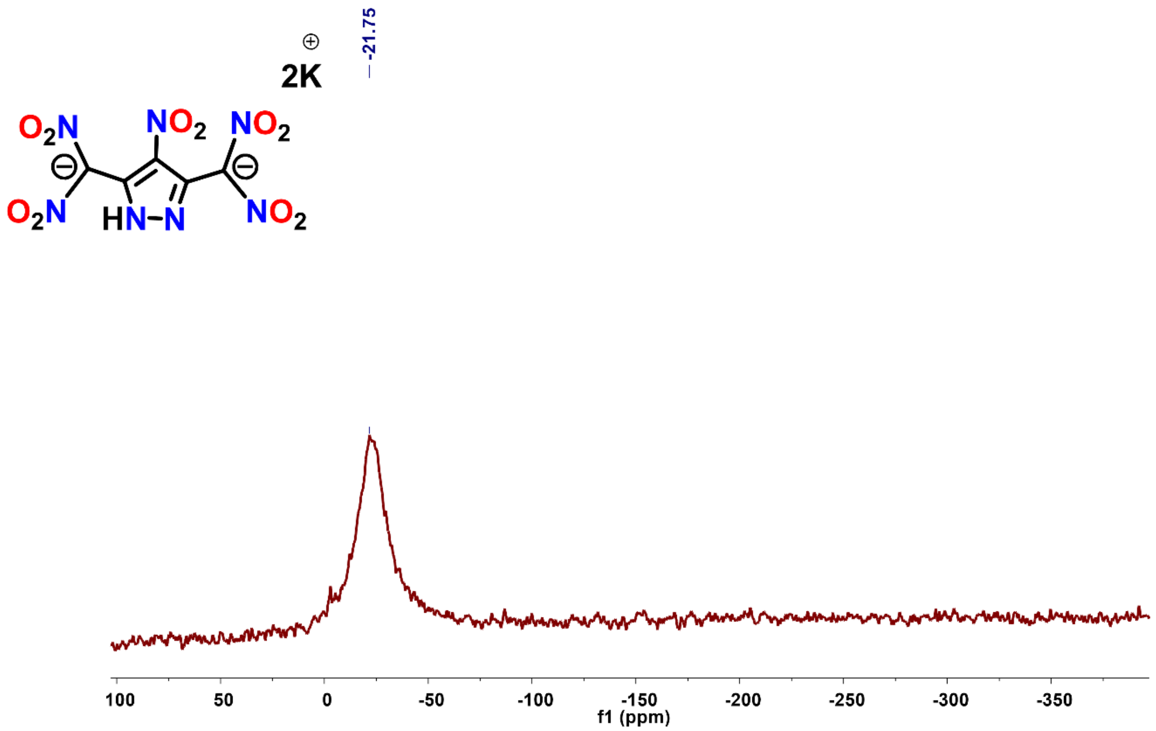

Fig. S6. ^{14}N NMR spectrum for compound **1**.

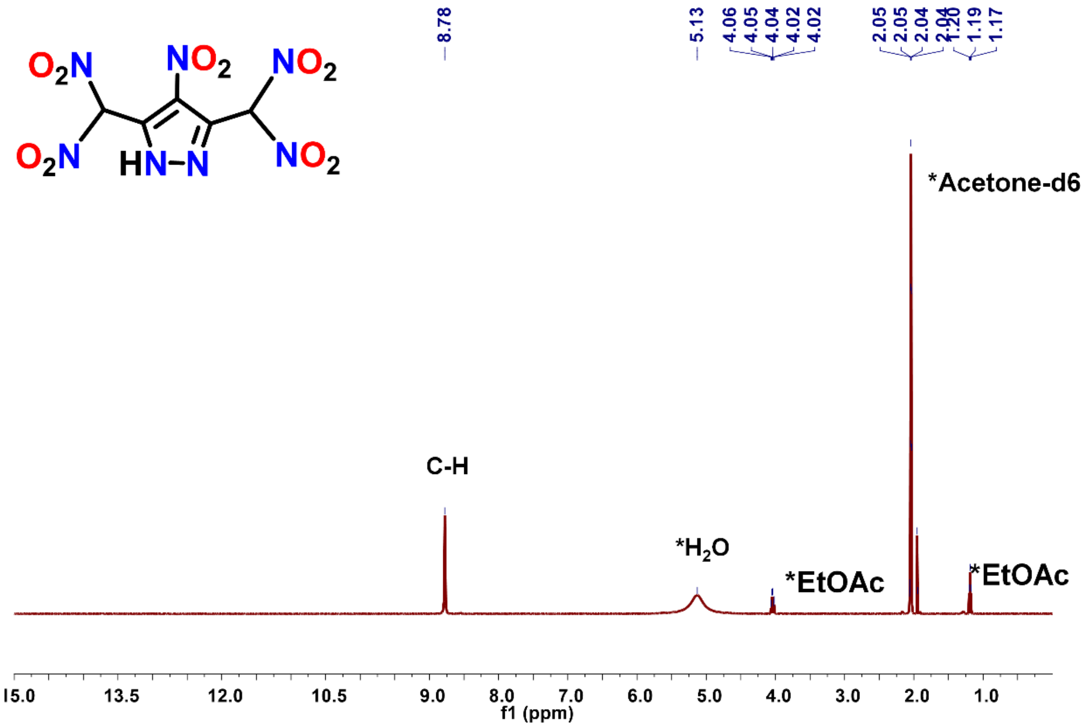

Fig. S7. ^{1}H NMR spectrum for intermediate **3**.

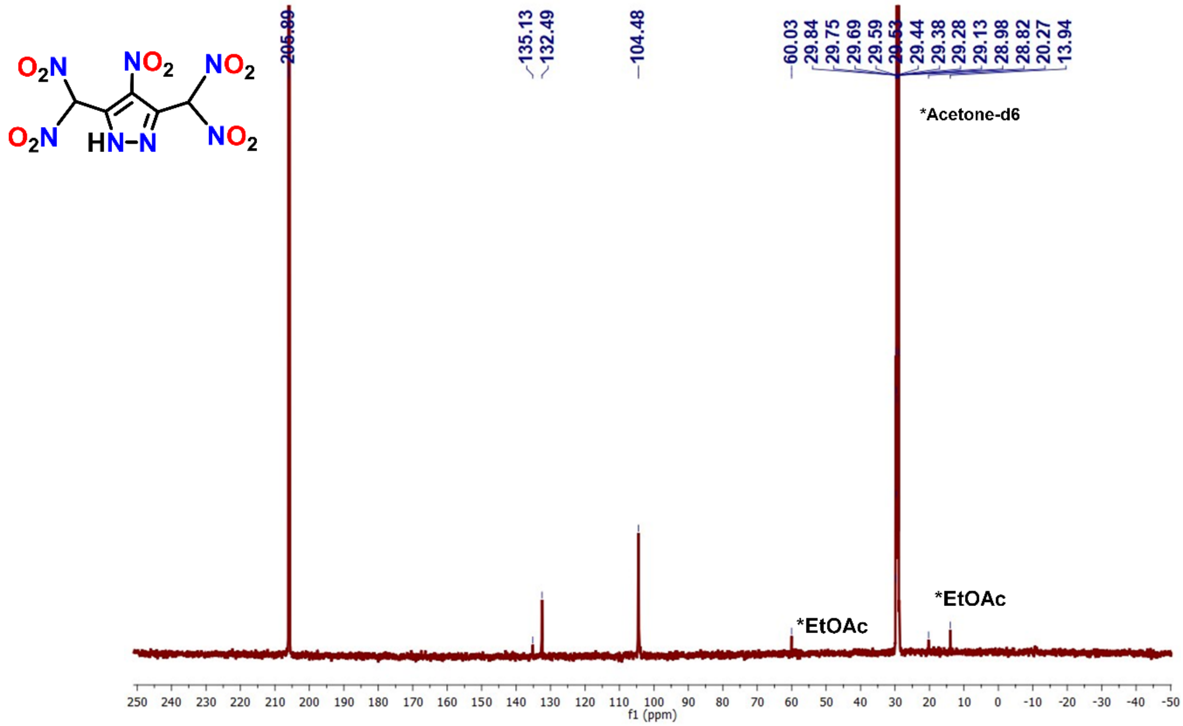

Fig. S8. ^{13}C NMR spectrum for intermediate **3**.

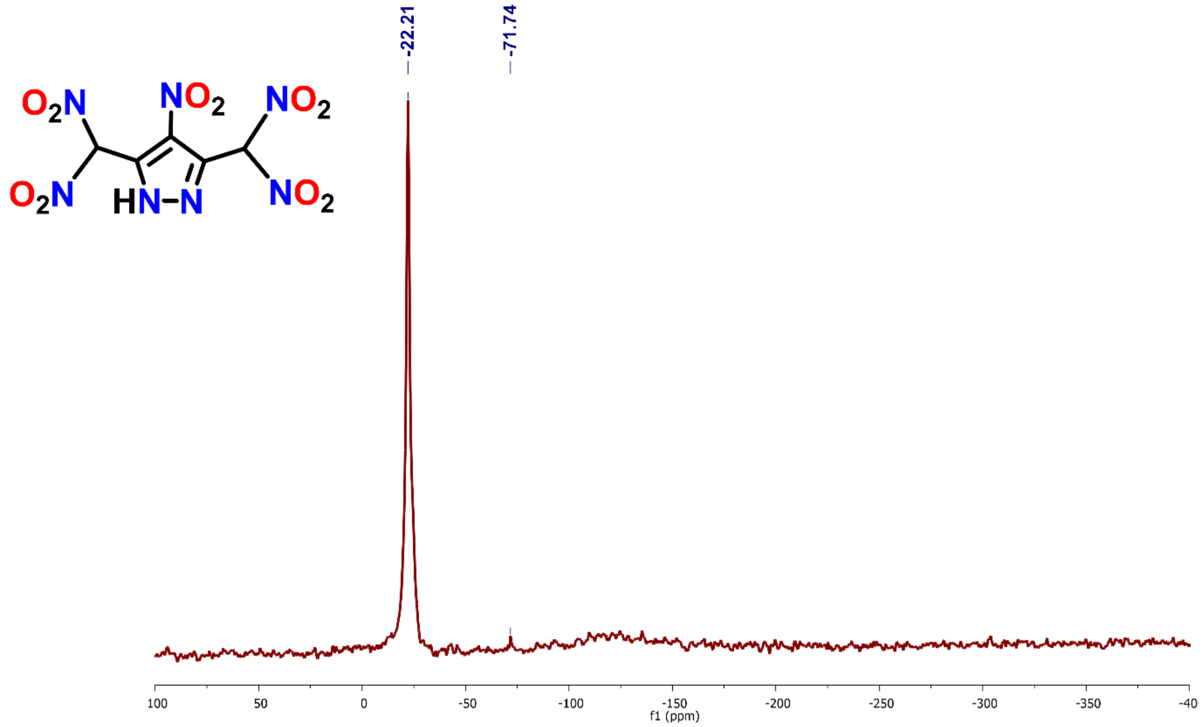

Fig. S9. [14]N NMR spectrum for intermediate **3**.

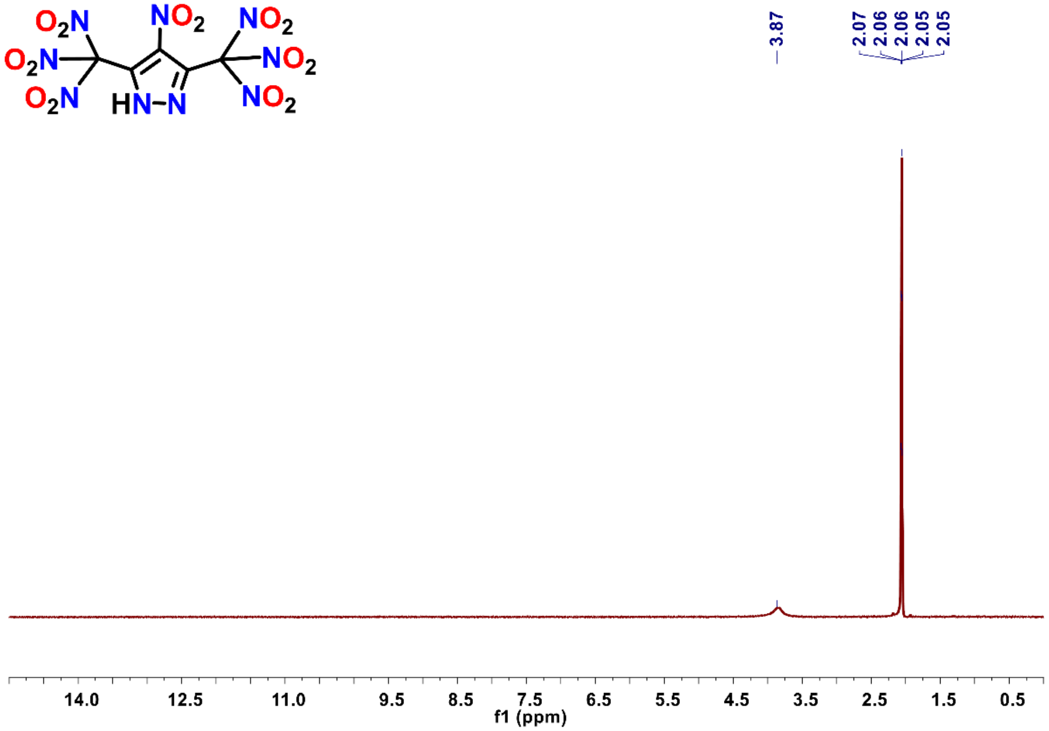

Fig. S10. ^{1}H NMR spectrum for compound **4**.

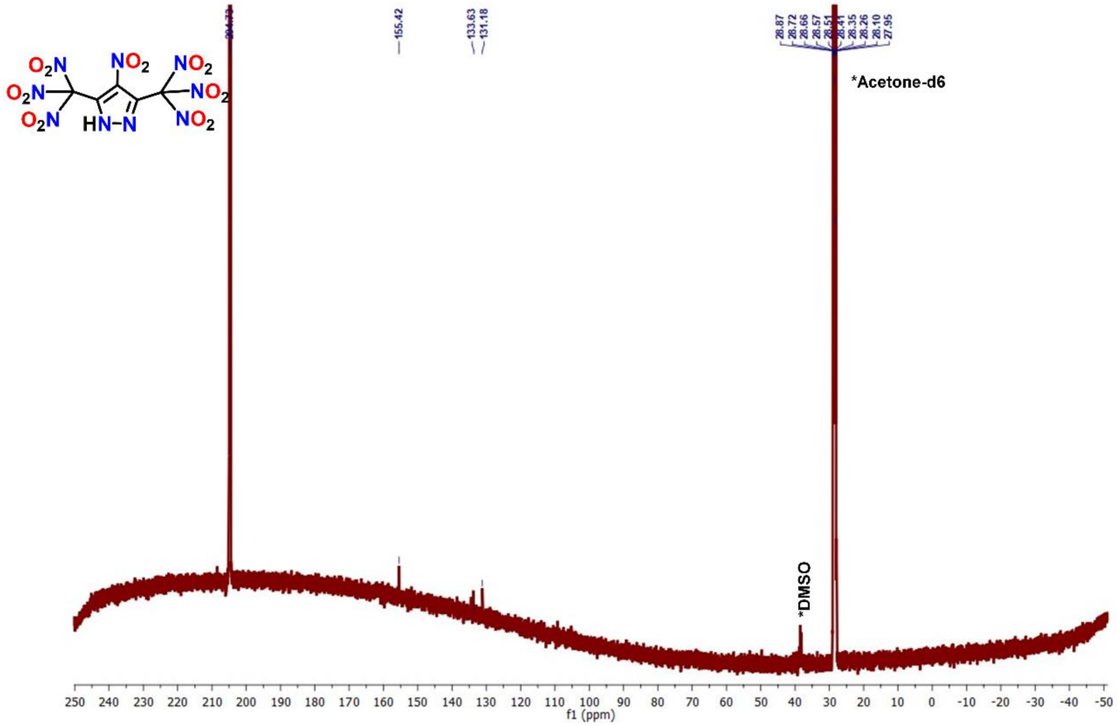

Fig. S11. ^{13}C NMR spectrum for compound **4**.

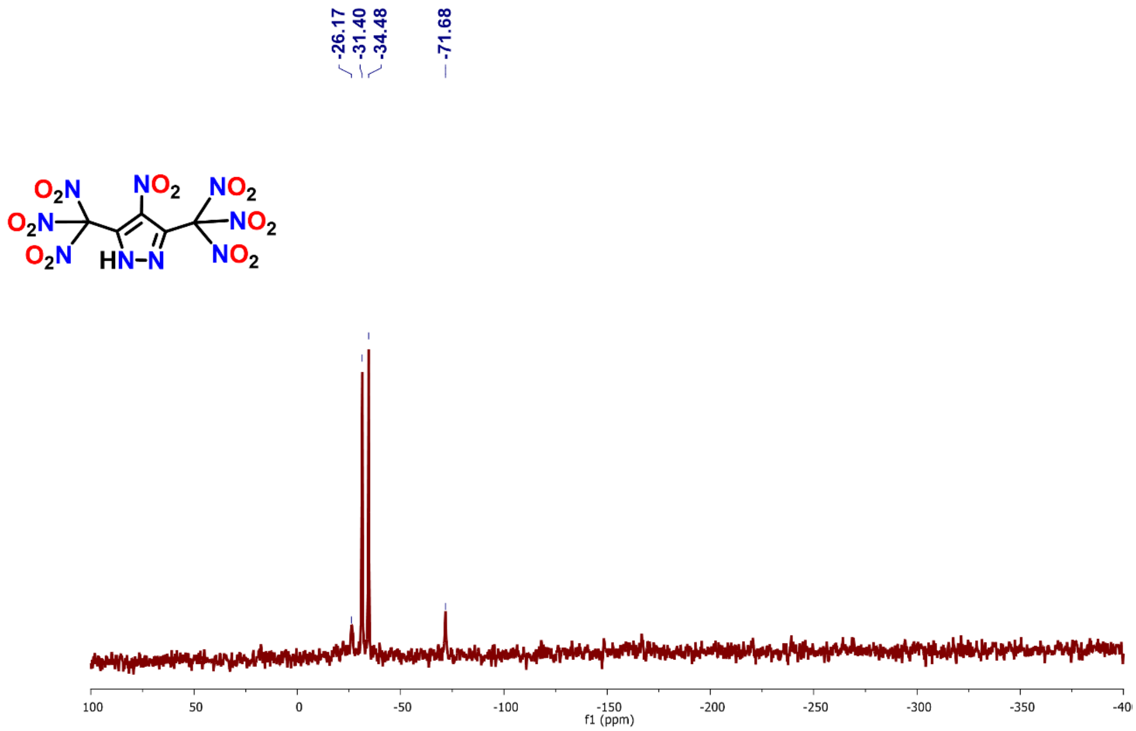

Fig. S12. ^{14}N NMR spectrum for compound **4**.

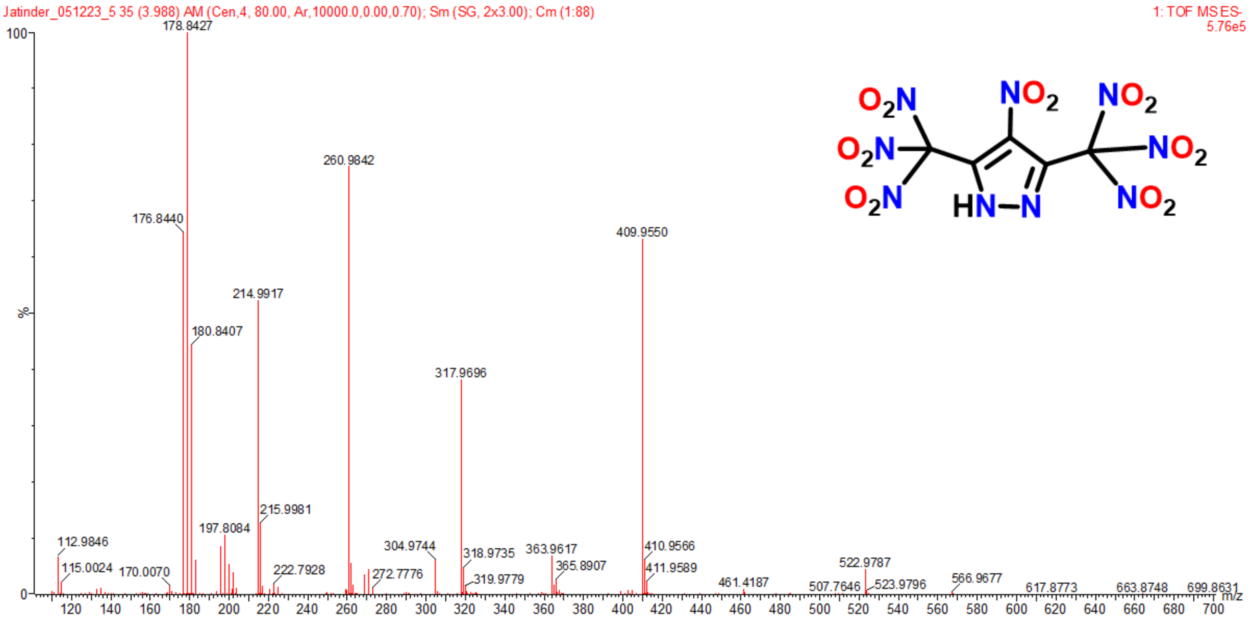

Fig. S13. Mass spectrum (full) for compound **4**.

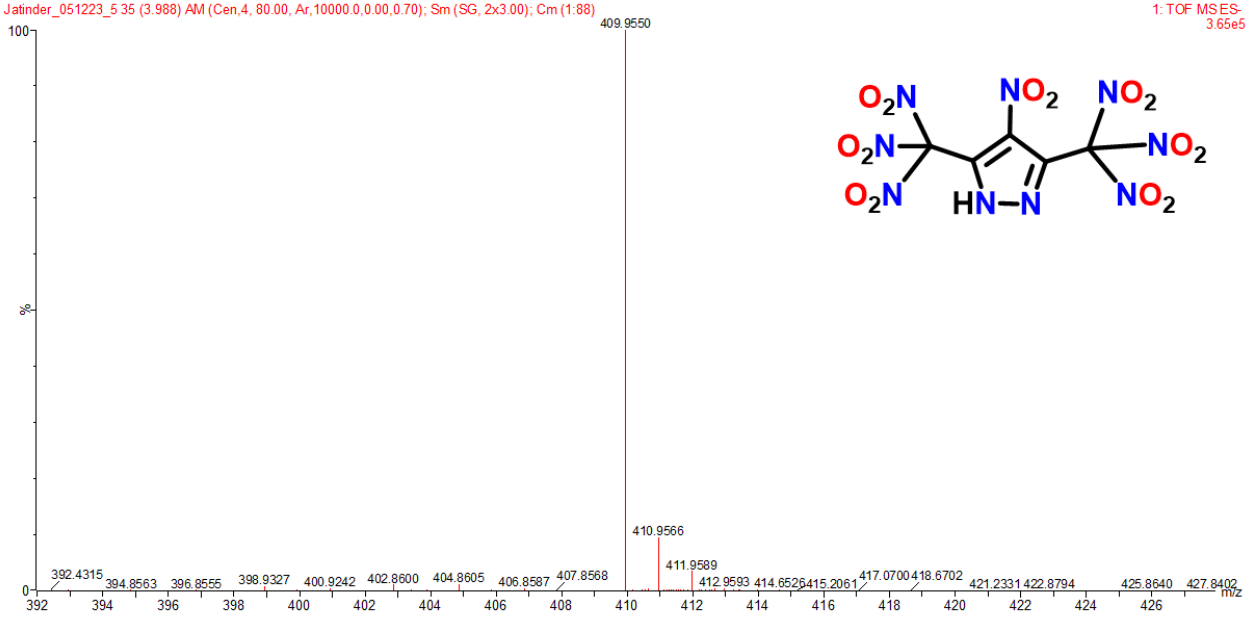

Fig. S14. Mass spectrum (expanded) for compound **4**.

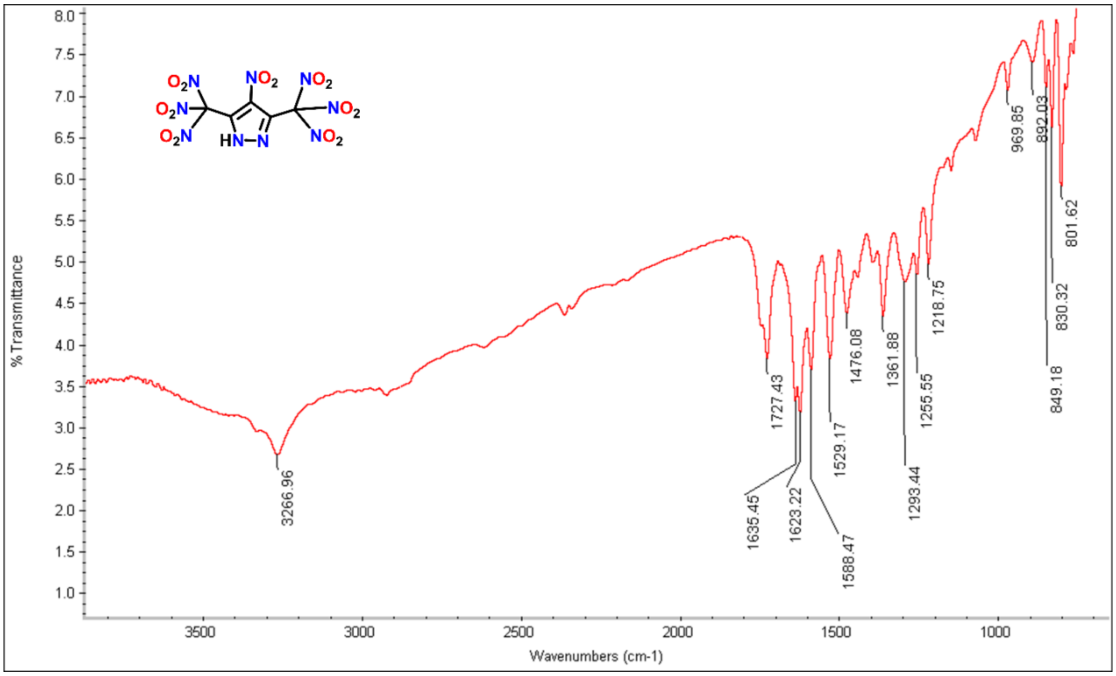

Fig. S15. FTIR spectrum for compound **4**.

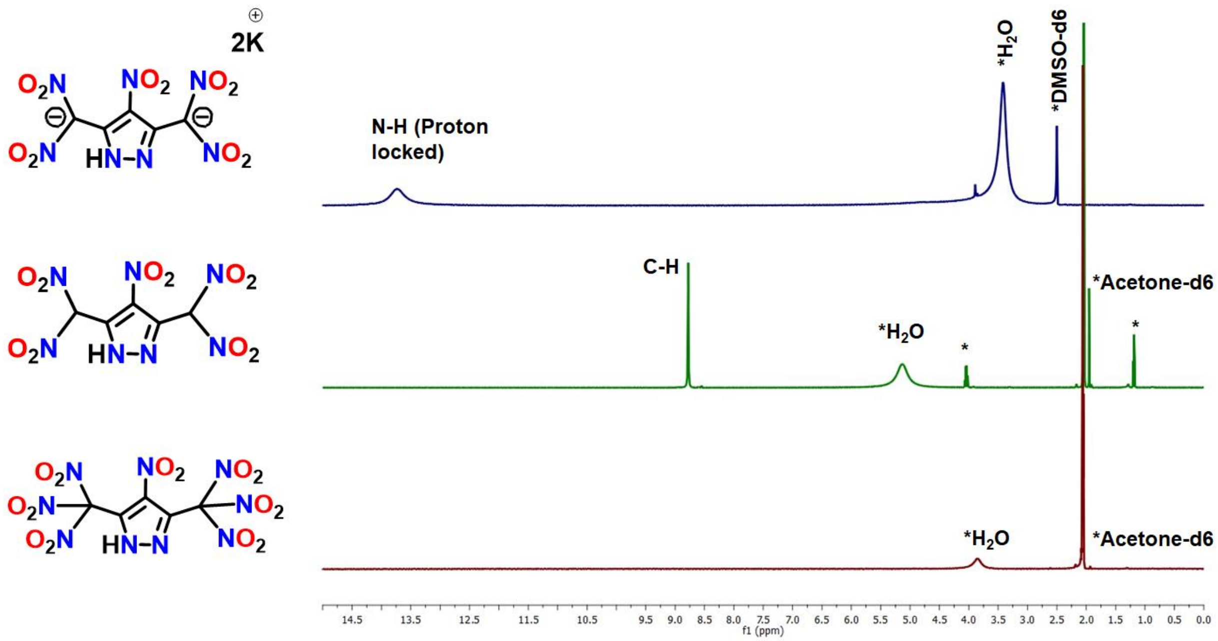

Fig. S16. ^{1}H NMR spectra (stacked) for compounds **1**, **3** and **4**.

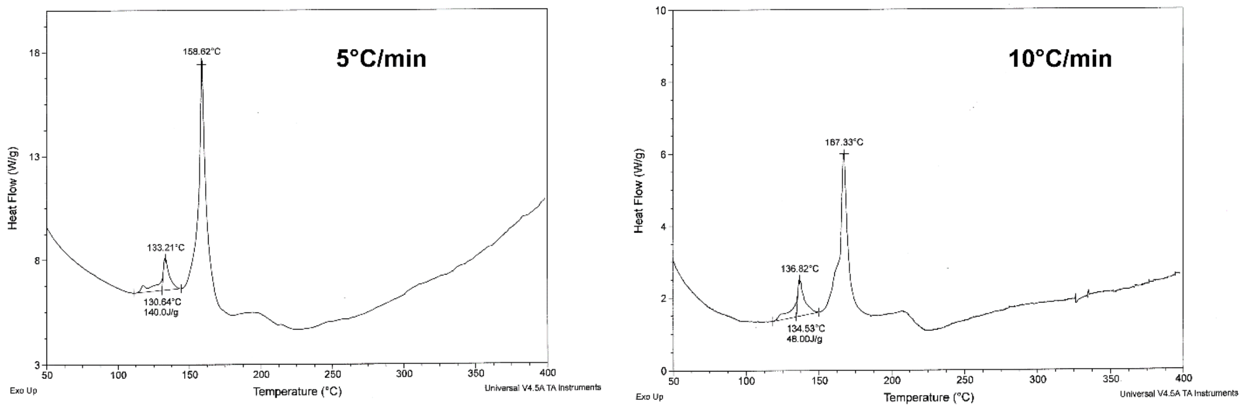

Fig. S17. DSC plot for compound **4** at the heating rate of 5 °C/min and 10 °C/min.

Other Supplementary Materials for this manuscript include the following:

Data S1: CIF files for Compound **4** (100K) (Data collected at 100 K) CCDC # 2262452 and Compound **4** (298K) (Data collected at 298 K) CCDC # 2298841

Data S2: Checkcif file for Compound **4** (100K) (Data collected at 100 K) CCDC # 2262452 and Compound **4** (298K) (Data collected at 298 K) CCDC # 22

REFERENCES AND NOTES

1. T. M. Klapötke, *Chemistry of high-energy materials* (De Gruyter, ed. 6. 2022).

2. J. P. Agrawal, High energy materials (Wiley, 2010).

3. M.-X. Zhang, P. E. Eaton, R. Gilardi, Hepta- and Octanitrocubanes. *Angew. Chem. Int. Ed.* **39**, 401–404 (2000).

4. C. C. Unger, M. Holler, B. Krumm, T. M. Klapötke, Oxygen-rich bis(trinitroethyl esters): Suitable oxidizers as potential ammonium perchlorate replacements. *Energy Fuels* **34**, 16469–16475 (2020).

5. T. T. Vo, D. A. Parrish, J. M. Shreeve, Tetranitroacetimidic acid: A high oxygen oxidizer and potential replacement for ammonium perchlorate. *J. Am. Chem. Soc.* **136**, 11934–11937 (2014).

6. G. Hervé, C. Roussel, H. Graindorge, Selective preparation of 3,4,5-Trinitro-1*H*-Pyrazole: A stable all-carbon-nitrated arene. *Angew. Chem. Int. Ed.* **49**, 3177–3181 (2010).

7. Q. Yu, P. Yin, J. Zhang, C. He, G. H. Imler, D. A. Parrish, J. M. Shreeve, Pushing the limits of oxygen balance in 1,3,4-oxadiazoles. *J. Am. Chem. Soc.* **139**, 8816–8819 (2017).

8. X. X. Zhao, S. H. Li, Y. Wang, Y. C. Li, F. Q. Zhao, S. P. Pang, Design and synthesis of energetic materials towards high density and positive oxygen balance by N-dinitromethyl functionalization of nitroazoles. *J. Mater. Chem. A* **4**, 5495–5504 (2016).

9. J. Zhang, S. Dharavath, L. A. Mitchell, D. A. Parrish, J. M. Shreeve, Energetic salts based on 3,5-bis(dinitromethyl)-1,2,4-triazole monoanion and dianion: Controllable preparation, characterization, and high performance. *J. Am. Chem. Soc.* **138**, 7500–7503 (2016).

10. G. Zhao, P. Yin, D. Kumar, G. H. Imler, D. A. Parrish, J. M. Shreeve, Bis(3-nitro-1-(trinitromethyl)-1*H*-1,2,4-triazol-5-yl)methanone: An applicable and very dense green oxidizer. *J. Am. Chem. Soc.* **141**, 19581–19584 (2019).

11. N. Ding, Q. Sun, X. Xu, Y. Li, C. Zhao, S. Li, S. Pang, Can a heavy trinitromethyl group always result in a higher density? *Chem. Commun.* **59**, 1939–1942 (2023).

12. K. Mohammad, V. Thaltiri, N. Kommu, A. A. Vargeese, Octanitropyrazolopyrazole: A gem-trinitromethyl based green high-density energetic oxidizer. *Chem. Commun.* **56**, 12945–12948 (2020).

13. M. A. Kettner, K. Karaghiosoff, T. M. Klapötke, M. Sućeska, S. Wunder, 3,3′-bi(1,2,4-oxadiazoles) featuring the fluorodinitromethyl and trinitromethyl groups. *Chemistry* **20**, 7622–7631 (2014).

14. Y. Zhang, D. A. Parrish, J. M. Shreeve, Synthesis and properties of 3,4,5-trinitropyrazole-1-ol and its energetic salts. *J. Mater. Chem.* **22**, 12659–12665 (2012).

15. D. Kumar, G. H. Imler, D. A. Parrish, J. M. Shreeve, 3,4,5-trinitro-1-(nitromethyl)-1*H*-pyrazole (TNNMP): A perchlorate free high energy density oxidizer with high thermal stability. *J. Mater. Chem. A* **5**, 10437–10441 (2017).

16. C. He, J. M. Shreeve, Potassium 4,5-bis(dinitromethyl)furoxanate: A green primary explosive with a positive oxygen balance. *Angew. Chem. Int. Ed.* **55**, 772–775 (2016).

17. I. L. Dalinger, I. A. Vatsadze, T. K. Shkineva, A. V. Kormanov, M. I. Struchkova, K. Y. Suponitsky, A. A. Bragin, K. A. Monogarov, V. P. Sinditskii, A. B. Sheremetev, Novel highly energetic pyrazoles: N-trinitromethyl-substituted nitropyrazoles. *Chem. Asian J.* **10**, 1987–1996 (2015).

18. J. Singh, R. J. Staples, J. M. Shreeve, Pushing the limit of nitro groups on a pyrazole ring with energy-stability balance. *ACS Appl. Mater. Interfaces* **13**, 61357–61364 (2021).

19. W. Zhang, Y. Yang, Y. Wang, T. Fei, Y. Wang, C. Sun, S. Pang, Challenging the limits of the oxygen balance of a pyrazole ring. *Chem. Eng. J.* **451**, 138609 (2023).

20. Q. Yu, G. H. Imler, D. A. Parrish, J. M. Shreeve, Challenging the limits of nitro groups associated with a tetrazole ring. *Org. Lett.* **21**, 4684–4688 (2019).

21. M. A. Spackman, J. J. McKinnon, Fingerprinting intermolecular interactions in molecular crystals. *CrstEngComm* **4**, 378–392 (2002).

22. M. A. Spackman, D. Jayatilaka, Hirshfeld surface analysis. *CrstEngComm* **11**, 19–32 (2009).

23. T. Lu, F. Chen, Multiwfn: A multifunctional wavefunction analyzer. *J. Comput. Chem.* **33**, 580–592 (2012).

24. L. I. Larina, Tautomerism and Structure of Azoles in *Advances in Heterocyclic Chemistry*, A. R., Katrisky, Ed. (Academic Press, 2018), pp. 233–321.

25. M. J. Frisch, G. W. Trucks, H. B. Schlegel, G. E. Scuseria, M. A. Robb, J. R. Cheeseman, G. Scalmani, V. Barone, G. A. Petersson, H. Nakatsuji, X. Li, M. Caricato, A. Marenich, J. Bloino, B. G. Janesko, R. Gomperts, B. Mennucci, H. P. Hratchian, J. V. Ortiz, A. F. Izmaylov, J. L. Sonnenberg, D. Williams-Young, F. Ding, F. Lipparini, F. Egidi, J. Goings, B. Peng, A. Petrone, T. Henderson, D. Ranasinghe, V. G. Zakrzewski, J. Gao, N. Rega, G. Zheng, W. Liang, M. Hada, M. Ehara, K. Toyota, R. Fukuda, J. Hasegawa, M. Ishida, T. Nakajima, Y. Honda, O. Kitao, H. Nakai, T. Vreven, K. Throssell, J. A. Montgomery Jr., J. E. Peralta, F. Ogliaro, M. Bearpark, J. J. Heyd, E. Brothers, K. N. Kudin, V. N. Staroverov, T. Keith, R. Kobayashi, J. Normand, K. Raghavachari, A. Rendell, J. C. Burant, S. S. Iyengar, J. Tomasi, M. Cossi, J. M. Millam, M. Klene, C. Adamo, R. Cammi, J. W. Ochterski, R. L. Martin, K. Morokuma, O. Farkas, J. B. Foresman and D. J. Fox, *Gaussian 09, Revision E.01* (Gaussian Inc., 2013).

26. M. Sućeska, *EXPLO5, Version 6.01* (Brodarski Institute, 2019).

27. R. Mayer , J. Köhler, A. Homburg, *Explosives* (Wiley-VCH, 2007).

28. H. Gao, J. M. Shreeve, Azole-based energetic salts. *Chem. Rev.* **111**, 7377–7436 (2011).

29. G. M. Sheldrick, A short history of *SHELX*. *Acta Crystallogr. A* **64**, 112–122 (2008).

30. G. M. Sheldrick, *SHELXT* – Integrated space-group and crystal-structure determination. *Acta Crystallogr. Sect. A Found. Adv.* **71** (Pt 1), 3–8 (2015).

31. O. V. Dolomanov, L. J. Bourhis, R. J. Gildea, J. A. K. Howard, H. Puschmann, *OLEX2*: A complete structure solution, refinement and analysis program. *J. Appl. Cryst.* **42**, 339–341 (2009).

32. R. G. Parr, Y. Weitao, *Density-Functional Theory of Atoms and Molecules* (Oxford Univ. Press, 1995).

33. O. Suleimenov, T.-K. Ha, Ab initio calculation of the thermochemical properties of polysulphanes (H_2S_n). *Chem. Phys. Lett.* **290**, 451–457 (1998).

34. M. S. Westwell, M. S. Searle, D. J. Wales, D. H. Williams, Empirical correlations between thermodynamic properties and intermolecular forces. *J. Am. Chem. Soc.* **117**, 5013–5015 (1995).

35. S. Wahler, P. Chung, T. M. Klapötke, Training machine learning models based on the structural formula for the enthalpy of vaporization and sublimation and a thorough analysis of Trouton's rules. *J. Energ. Mater.* 10.1080/07370652.2023.2219678 (2023).

36. N. V. Muravyev, K. A. Monogarov, I. N. Melnikov, A. N. Pivkina, V. G. Kiselev, Learning to fly: Thermochemistry of energetic materials by modified thermogravimetric analysis and highly accurate quantum chemical calculations. *Phys. Chem. Chem. Phys.* **23**, 15522–15542 (2021).
