# Supplementary material for: Manipulating nitration and stabilization to achieve high energy
Source: Sci Adv. 2023 Nov 15;9(46):eadk3754. doi: 10.1126/sciadv.adk3754 (PMC10651134; doi:10.1126/sciadv.adk3754)
Supplement: Supplementary file 2 — Data S1 and S2 [file sciadv.adk3754_data_s1_and_s2.zip › adk3754_Data_S1.pdf]

data_js323f_rm

_audit_creation_date 2023-09-28

_audit_creation_method

;

Olex2 1.5

(compiled 2023.08.24 svn.re1ec1418 for OlexSys, GUI svn.r6817)

;

_shelx_SHELXL_version_number '2018/3'

loop_

_audit_author_name

_audit_author_email

_audit_author_address

'Shreeve, J.M.' ''

;

University of Idaho

Chemistry

Univeristy of Idaho

;

'Staples, R.J.' staples@chemistry.msu.edu

;

Michigan State University

Department of Chemistry

Michigan State University

East Lansing, MI

48824

;

_audit_contact_author_address ?

_audit_contact_author_email ?

```
_audit_contact_author_name        ''
_audit_contact_author_phone       ?
_publ_contact_author_id_orcid     ?
_publ_section_references
;
Dolomanov, O.V., Bourhis, L.J., Gildea, R.J, Howard, J.A.K. & Puschmann, H.
 (2009), J. Appl. Cryst. 42, 339-341.

Sheldrick, G.M. (2015). Acta Cryst. A71, 3-8.

Sheldrick, G.M. (2015). Acta Cryst. C71, 3-8.
;
_chemical_name_common             ?
_chemical_name_systematic         ?
_chemical_formula_moiety          'C5 H N9 O14'
_chemical_formula_sum             'C5 H N9 O14'
_chemical_formula_weight          411.15
_chemical_melting_point           ?
_chemical_oxdiff_formula          C5HN9O14
_chemical_oxdiff_usercomment      'JS323F Room'
loop_
  _atom_type_symbol
  _atom_type_description
  _atom_type_scat_dispersion_real
  _atom_type_scat_dispersion_imag
  _atom_type_scat_source
 'C' 'C' 0.0181 0.0091 'International Tables Vol C Tables 4.2.6.8 and 6.1.1.4'
 'H' 'H' 0.0000 0.0000 'International Tables Vol C Tables 4.2.6.8 and 6.1.1.4'
 'N' 'N' 0.0311 0.0180 'International Tables Vol C Tables 4.2.6.8 and 6.1.1.4'
```

'O' 'O' 0.0492 0.0322 'International Tables Vol C Tables 4.2.6.8 and 6.1.1.4'

_shelx_space_group_comment
;
The symmetry employed for this shelxl refinement is uniquely defined
by the following loop, which should always be used as a source of
symmetry information in preference to the above space-group names.
They are only intended as comments.
;
_space_group_crystal_system 'monoclinic'
_space_group_IT_number 14
_space_group_name_H-M_alt 'P 1 21/n 1'
_space_group_name_Hall '-P 2yn'
loop_
 _space_group_symop_operation_xyz
 'x, y, z'
 '-x+1/2, y+1/2, -z+1/2'
 '-x, -y, -z'
 'x-1/2, -y-1/2, z-1/2'

_cell_length_a 10.8108(6)
_cell_length_b 10.7147(6)
_cell_length_c 12.2728(9)
_cell_angle_alpha 90
_cell_angle_beta 103.218(6)
_cell_angle_gamma 90
_cell_volume 1383.95(15)
_cell_formula_units_Z 4
_cell_measurement_reflns_used 8481

_cell_measurement_temperature 298.00(10)

_cell_measurement_theta_max 79.3140

_cell_measurement_theta_min 4.9110

_shelx_estimated_absorpt_T_max 0.915

_shelx_estimated_absorpt_T_min 0.692

_exptl_absorpt_coefficient_mu 1.810

_exptl_absorpt_correction_T_max 1.000

_exptl_absorpt_correction_T_min 0.709

_exptl_absorpt_correction_type gaussian

_exptl_absorpt_process_details

;

CrysAlisPro 1.171.42.102a (Rigaku Oxford Diffraction, 2023)

Numerical absorption correction based on gaussian integration over

a multifaceted crystal model

Empirical absorption correction using spherical harmonics,

implemented in SCALE3 ABSPACK scaling algorithm.

;

_exptl_absorpt_special_details ?

_exptl_crystal_colour colourless

_exptl_crystal_colour_primary colourless

_exptl_crystal_density_diffrn 1.973

_exptl_crystal_density_meas ?

_exptl_crystal_density_method ?

_exptl_crystal_description needle

_exptl_crystal_F_000 824

loop_

_exptl_crystal_face_index_h

_exptl_crystal_face_index_k

_exptl_crystal_face_index_l

_exptl_crystal_face_perp_dist

0 -1 0 0.0284

-7 0 3 0.1134

7 -1 -3 0.0983

-1 -1 -7 0.0286

1 1 7 0.0210

0 7 0 0.0413

0 -5 -5 0.0278

0 3 -7 0.0211

_exptl_crystal_recrystallization_method 'used as received'

_exptl_crystal_size_max 0.22

_exptl_crystal_size_mid 0.07

_exptl_crystal_size_min 0.05

loop_

_exptl_oxdiff_crystal_face_indexfrac_h

_exptl_oxdiff_crystal_face_indexfrac_k

_exptl_oxdiff_crystal_face_indexfrac_l

_exptl_oxdiff_crystal_face_x

_exptl_oxdiff_crystal_face_y

_exptl_oxdiff_crystal_face_z

-0.0003 -0.9817 0.1125 0.0883 0.0001 0.1110

-6.9204 0.3550 3.0118 0.7612 -0.0209 -0.6482

6.9043 -0.6576 -2.9646 -0.7320 0.0202 0.6810

-1.0209 -0.9286 -7.3383 0.0263 0.9994 0.0243

0.9683 0.9947 7.3498 -0.0264 -0.9990 -0.0364

-0.0295 6.9444 -0.4001 -0.6129 -0.0492 -0.7886

0.1740 -5.3193 -5.0211 0.3361 0.7061 0.6233

-0.2180 2.6197 -7.1362 -0.3637 0.8793 -0.3076

_exptl_special_details

;

Data was collected using a Rigaku Synergy S Diffractometer diffractometer equipped

with an Oxford 800 low-temperature apparatus. A suitable

crystal was chosen and mounted on a nylon loop using Paratone oil. Data were

measured based on the Pre-Experiment plugin of CrysAlisPro software. Cell

parameters were retrieved using CrysAlisPro software and data reduction was

performed using the intergration software inside the CrysAlisPro which

corrects for Lp. The structure was solved by the direct method using the

SHELXT program and refined by least squares method on F2, SHELXL,

 incorporated in OLEX2.

;

_exptl_transmission_factor_max   ?

_exptl_transmission_factor_min   ?

_diffrn_reflns_av_R_equivalents   0.0500

_diffrn_reflns_av_unetI/netI   0.0367

_diffrn_reflns_Laue_measured_fraction_full 0.997

_diffrn_reflns_Laue_measured_fraction_max 0.979

_diffrn_reflns_limit_h_max   13

_diffrn_reflns_limit_h_min   -12

_diffrn_reflns_limit_k_max   12

_diffrn_reflns_limit_k_min   -13

_diffrn_reflns_limit_l_max   15

_diffrn_reflns_limit_l_min   -15

_diffrn_reflns_number   13956

_diffrn_reflns_point_group_measured_fraction_full 0.997

_diffrn_reflns_point_group_measured_fraction_max 0.979

_diffrn_reflns_theta_full   67.684

_diffrn_reflns_theta_max 80.019

_diffrn_reflns_theta_min 4.924

_diffrn_ambient_environment air

_diffrn_ambient_temperature 298.00(10)

_diffrn_detector 'Hybrid Pixel Array Detector'

_diffrn_detector_area_resol_mean 10.0000

_diffrn_detector_type HyPix

_diffrn_measured_fraction_theta_full 0.997

_diffrn_measured_fraction_theta_max 0.979

_diffrn_measurement_details

;

List of Runs (angles in degrees, time in seconds):

 # Type Start End Width t~exp~ \w \q \k \f Frames

----

 1 \w 36.00 93.00 0.50 0.08 -- 47.12-125.00-150.00 114

 2 \w 36.00 93.00 0.50 0.08 -- 47.12-125.00 -30.00 114

 3 \w 42.00 104.00 0.50 0.32 -- 113.25 -94.00 0.00 124

 4 \w 42.00 104.00 0.50 0.32 -- 113.25 -94.00 -60.00 124

 5 \w 42.00 94.00 0.50 0.32 -- 113.25 -77.00-120.00 104

 6 \w 42.00 69.00 0.50 0.32 -- 113.25 -61.00-180.00 54

 7 \w 42.00 94.00 0.50 0.32 -- 113.25 -77.00-150.00 104

 8 \w 42.00 94.00 0.50 0.32 -- 113.25 -77.00 -60.00 104

 9 \w 42.00 69.00 0.50 0.32 -- 113.25 -61.00 0.00 54

 10 \w 42.00 69.00 0.50 0.32 -- 113.25 -61.00 -90.00 54

 11 \w 42.00 94.00 0.50 0.32 -- 113.25 -77.00 90.00 104

 12 \w 42.00 69.00 0.50 0.32 -- 113.25 -61.00 120.00 54

 13 \w 42.00 69.00 0.50 0.32 -- 113.25 -61.00 150.00 54

 14 \w 42.00 94.00 0.50 0.32 -- 113.25 -77.00 150.00 104

```
 15  \w    42.00  69.00   0.50    0.32    --  113.25 -61.00  30.00   54
 16  \w    42.00  69.00   0.50    0.32    --  113.25 -61.00 -30.00   54
 17  \w   -24.00  57.00   0.50    0.08    --   47.12 -50.00 106.00  162
 18  \w   106.00 178.00   0.50    0.32    --  113.25  61.00   0.00  144
 19  \w   101.00 178.00   0.50    0.32    --  113.25  45.00 -30.00  154
 20  \w   101.00 178.00   0.50    0.32    --  113.25  45.00 -90.00  154
 21  \w   100.00 178.00   0.50    0.32    --  113.25  50.00-106.00  156
 22  \w   100.00 178.00   0.50    0.32    --  113.25  15.00-180.00  156
 23  \w    34.00 120.00   0.50    0.08    --   47.12  50.00-106.00  172
 24  \w    35.00  90.00   0.50    0.08    --   47.12  37.00   0.00  110
 25  \w   -58.00  25.00   0.50    0.08    --  -47.12  50.00-106.00  166
 26  \w   -58.00 -19.00   0.50    0.32    --  -91.75  50.00-106.00   78
 27  \w   -53.00 -20.00   0.50    0.32    --  -91.75  45.00  30.00   66
 28  \w   -53.00 -20.00   0.50    0.32    --  -91.75  45.00  60.00   66
 29  \w  -163.00 -78.00   0.50    0.32    --  -91.75 -50.00 106.00  170
 30  \w  -119.00 -34.00   0.50    0.08    --  -47.12 -50.00 106.00  170
;
_diffrn_measurement_device        'four-circle diffractometer'
_diffrn_measurement_device_type   'XtaLAB Synergy, Dualflex, HyPix'
_diffrn_measurement_method        '\w scans'
_diffrn_orient_matrix_type
 'CrysAlisPro convention (1999,Acta A55,543-557)'
_diffrn_orient_matrix_UB_11       -0.1048771829
_diffrn_orient_matrix_UB_12       -0.0874346253
_diffrn_orient_matrix_UB_13       0.0220648733
_diffrn_orient_matrix_UB_21       -0.0529861235
_diffrn_orient_matrix_UB_22       -0.0146228340
_diffrn_orient_matrix_UB_23       -0.1269614533
_diffrn_orient_matrix_UB_31       0.0873568579
```

_diffrn_orient_matrix_UB_32 -0.1132559920

_diffrn_orient_matrix_UB_33 -0.0011347494

_diffrn_radiation_monochromator mirror

_diffrn_radiation_probe x-ray

_diffrn_radiation_type 'Cu K\a'

_diffrn_radiation_wavelength 1.54184

_diffrn_source 'micro-focus sealed X-ray tube'

_diffrn_source_current 1

_diffrn_source_type 'PhotonJet (Cu) X-ray Source'

_diffrn_source_voltage 50

_reflns_Friedel_coverage 0.000

_reflns_Friedel_fraction_full .

_reflns_Friedel_fraction_max .

_reflns_number_gt 2565

_reflns_number_total 2960

_reflns_special_details

;

Reflections were merged by SHELXL according to the crystal

class for the calculation of statistics and refinement.

_reflns_Friedel_fraction is defined as the number of unique

Friedel pairs measured divided by the number that would be

possible theoretically, ignoring centric projections and

systematic absences.

;

_reflns_threshold_expression 'I > 2\s(I)'

_computing_cell_refinement 'CrysAlisPro 1.171.42.102a (Rigaku OD, 2023)'

_computing_data_collection 'CrysAlisPro 1.171.42.102a (Rigaku OD, 2023)'

_computing_data_reduction 'CrysAlisPro 1.171.42.102a (Rigaku OD, 2023)'

_computing_molecular_graphics

;

O. V. Dolomanov, L. J. Bourhis, R. J. Gildea, J. A. K. Howard and H. Puschmann,

OLEX2: a complete structure solution, refinement and analysis program.

J. Appl. Cryst. (2009). 42, 339-341.

;

_computing_publication_material

;

O. V. Dolomanov, L. J. Bourhis, R. J. Gildea, J. A. K. Howard and H. Puschmann,

OLEX2: a complete structure solution, refinement and analysis program.

J. Appl. Cryst. (2009). 42, 339-341.

;

_computing_structure_refinement 'SHELXL 2018/3 (Sheldrick, 2015)'

_computing_structure_solution 'SHELXT (Sheldrick, 2015)'

_refine_diff_density_max 0.283

_refine_diff_density_min -0.235

_refine_diff_density_rms 0.053

_refine_ls_extinction_coef .

_refine_ls_extinction_method none

_refine_ls_goodness_of_fit_ref 1.086

_refine_ls_hydrogen_treatment refall

_refine_ls_matrix_type full

_refine_ls_number_parameters 257

_refine_ls_number_reflns 2960

_refine_ls_number_restraints 0

_refine_ls_R_factor_all 0.0477

_refine_ls_R_factor_gt 0.0427

_refine_ls_restrained_S_all 1.086

_refine_ls_shift/su_max 0.000

```
_refine_ls_shift/su_mean          0.000
_refine_ls_structure_factor_coef   Fsqd
_refine_ls_weighting_details
 'w=1/[\s^2^(Fo^2^)+(0.0622P)^2^+0.4277P] where P=(Fo^2^+2Fc^2^)/3'
_refine_ls_weighting_scheme       calc
_refine_ls_wR_factor_gt           0.1172
_refine_ls_wR_factor_ref          0.1243
_refine_special_details
;
The structure was refined by Least Squares SHELXL incorporated
in Olex2 software program. All non-hydrogen atoms were refined anisotropically.
Hydrogen atom positions were calculated geometrically and refined using the
riding model, except for the hydrogen atom on the non-carbon atom(s) which were found
by difference Fourier methods and refined isotropically when data permits.
;
_olex2_refinement_description
;
;
_atom_sites_solution_hydrogens     difmap
_atom_sites_solution_primary       dual
_atom_sites_solution_secondary     ?
loop_
  _atom_site_label
  _atom_site_type_symbol
  _atom_site_fract_x
  _atom_site_fract_y
  _atom_site_fract_z
  _atom_site_U_iso_or_equiv
  _atom_site_adp_type
```

_atom_site_occupancy

_atom_site_site_symmetry_order

_atom_site_calc_flag

_atom_site_refinement_flags_posn

_atom_site_refinement_flags_adp

_atom_site_refinement_flags_occupancy

_atom_site_disorder_assembly

_atom_site_disorder_group

O1 O 0.20430(13) 0.42548(15) 0.20413(15) 0.0637(5) Uani 1 1 d . . . . .

O2 O 0.40331(12) 0.42713(14) 0.20213(13) 0.0520(4) Uani 1 1 d . . . . .

O3 O -0.04597(14) 0.32670(18) 0.20096(14) 0.0648(4) Uani 1 1 d . . . . .

O4 O 0.07854(16) 0.17475(16) 0.17673(13) 0.0611(4) Uani 1 1 d . . . . .

O5 O -0.02567(14) 0.39352(15) 0.41886(16) 0.0652(4) Uani 1 1 d . . . . .

O6 O 0.16745(16) 0.45541(16) 0.4287(2) 0.0767(6) Uani 1 1 d . . . . .

O7 O -0.05823(12) 0.12193(14) 0.36034(14) 0.0546(4) Uani 1 1 d . . . . .

O8 O 0.09764(14) 0.13215(15) 0.50654(12) 0.0540(4) Uani 1 1 d . . . . .

O9 O 0.48930(15) 0.18526(19) 0.15910(14) 0.0673(5) Uani 1 1 d . . . . .

O10 O 0.69373(15) 0.1988(2) 0.20524(15) 0.0702(5) Uani 1 1 d . . . . .

O11 O 0.58629(17) 0.44276(16) 0.42723(18) 0.0720(5) Uani 1 1 d . . . . .

O12 O 0.69655(16) 0.42574(18) 0.30139(19) 0.0780(6) Uani 1 1 d . . . . .

O13 O 0.74813(15) 0.22534(18) 0.49714(15) 0.0691(5) Uani 1 1 d . . . . .

O14 O 0.67414(14) 0.06149(14) 0.40047(14) 0.0571(4) Uani 1 1 d . . . . .

N1 N 0.31845(13) 0.15534(14) 0.44368(13) 0.0369(3) Uani 1 1 d . . . . .

H1 H 0.290(2) 0.112(2) 0.483(2) 0.046(6) Uiso 1 1 d . . . . .

N2 N 0.44105(13) 0.15187(14) 0.44120(12) 0.0388(3) Uani 1 1 d . . . . .

N3 N 0.31308(13) 0.38728(14) 0.23534(13) 0.0408(3) Uani 1 1 d . . . . .

N4 N 0.04036(14) 0.25375(16) 0.23119(13) 0.0443(4) Uani 1 1 d . . . . .

N5 N 0.08094(14) 0.38329(14) 0.40683(15) 0.0451(4) Uani 1 1 d . . . . .

N6 N 0.04297(13) 0.15930(14) 0.41179(13) 0.0392(3) Uani 1 1 d . . . . .

N7 N 0.58906(15) 0.20756(16) 0.22333(14) 0.0455(4) Uani 1 1 d . . . . .

N8 N 0.62657(14) 0.38776(15) 0.35713(16) 0.0495(4) Uani 1 1 d . . . . .

N9 N 0.67894(13) 0.17202(16) 0.42106(14) 0.0446(4) Uani 1 1 d . . . . .

C1 C 0.25002(14) 0.23584(14) 0.36980(14) 0.0334(3) Uani 1 1 d . . . . .

C2 C 0.33624(14) 0.29008(15) 0.31678(14) 0.0348(3) Uani 1 1 d . . . . .

C3 C 0.45364(14) 0.23359(15) 0.36433(14) 0.0342(3) Uani 1 1 d . . . . .

C4 C 0.11071(15) 0.25701(15) 0.35494(14) 0.0350(3) Uani 1 1 d . . . . .

C5 C 0.58149(14) 0.25136(15) 0.34002(14) 0.0364(4) Uani 1 1 d . . . . .

loop_

_atom_site_aniso_label

_atom_site_aniso_U_11

_atom_site_aniso_U_22

_atom_site_aniso_U_33

_atom_site_aniso_U_23

_atom_site_aniso_U_13

_atom_site_aniso_U_12

O1 0.0397(7) 0.0654(9) 0.0821(11) 0.0387(8) 0.0057(7) 0.0095(6)

O2 0.0431(7) 0.0528(8) 0.0584(8) 0.0203(6) 0.0079(6) -0.0094(6)

O3 0.0419(7) 0.0937(12) 0.0543(9) 0.0196(8) 0.0018(6) 0.0156(7)

O4 0.0684(9) 0.0700(10) 0.0452(8) -0.0144(7) 0.0134(7) -0.0074(8)

O5 0.0520(8) 0.0603(9) 0.0883(12) -0.0148(8) 0.0265(8) 0.0115(7)

O6 0.0604(9) 0.0497(9) 0.1206(16) -0.0294(9) 0.0218(10) -0.0075(7)

O7 0.0351(6) 0.0577(8) 0.0714(10) 0.0032(7) 0.0126(6) -0.0076(6)

O8 0.0532(8) 0.0656(9) 0.0459(8) 0.0124(7) 0.0173(6) 0.0006(6)

O9 0.0557(8) 0.0927(12) 0.0505(9) -0.0179(8) 0.0060(7) -0.0052(8)

O10 0.0510(8) 0.1009(13) 0.0654(10) 0.0145(9) 0.0275(7) 0.0187(8)

O11 0.0677(10) 0.0545(9) 0.0884(13) -0.0224(9) 0.0067(9) -0.0078(8)

O12 0.0595(9) 0.0672(10) 0.1096(15) 0.0214(10) 0.0237(10) -0.0206(8)

O13 0.0507(8) 0.0847(12) 0.0588(10) 0.0080(8) -0.0148(7) 0.0027(8)

O14 0.0521(8) 0.0478(8) 0.0723(10) 0.0187(7) 0.0158(7) 0.0131(6)

N1 0.0349(7) 0.0376(7) 0.0388(7) 0.0090(6) 0.0098(6) 0.0020(5)

N2 0.0332(7) 0.0420(7) 0.0403(7) 0.0074(6) 0.0065(5) 0.0036(5)

N3 0.0355(7) 0.0395(7) 0.0452(8) 0.0114(6) 0.0047(6) -0.0004(6)

N4 0.0373(7) 0.0558(9) 0.0391(8) 0.0025(7) 0.0071(6) -0.0034(6)

N5 0.0416(7) 0.0390(8) 0.0544(9) -0.0022(7) 0.0103(7) 0.0060(6)

N6 0.0362(7) 0.0391(7) 0.0456(8) 0.0023(6) 0.0164(6) 0.0034(5)

N7 0.0428(8) 0.0494(8) 0.0450(8) 0.0069(7) 0.0115(6) 0.0053(6)

N8 0.0361(7) 0.0425(8) 0.0639(11) 0.0049(8) -0.0007(7) -0.0055(6)

N9 0.0310(7) 0.0537(9) 0.0485(9) 0.0147(7) 0.0076(6) 0.0056(6)

C1 0.0318(7) 0.0328(7) 0.0347(8) 0.0011(6) 0.0057(6) 0.0022(6)

C2 0.0320(7) 0.0340(7) 0.0369(8) 0.0062(6) 0.0047(6) 0.0000(6)

C3 0.0313(7) 0.0341(7) 0.0355(8) 0.0046(6) 0.0045(6) 0.0005(6)

C4 0.0323(7) 0.0351(8) 0.0377(8) 0.0003(6) 0.0082(6) 0.0013(6)

C5 0.0299(7) 0.0376(8) 0.0397(9) 0.0066(7) 0.0036(6) 0.0008(6)

_geom_special_details

;

All esds (except the esd in the dihedral angle between two l.s. planes)

are estimated using the full covariance matrix. The cell esds are taken

into account individually in the estimation of esds in distances, angles

and torsion angles; correlations between esds in cell parameters are only

used when they are defined by crystal symmetry. An approximate (isotropic)

treatment of cell esds is used for estimating esds involving l.s. planes.

;

loop_

_geom_bond_atom_site_label_1

_geom_bond_atom_site_label_2

_geom_bond_distance

_geom_bond_site_symmetry_2

_geom_bond_publ_flag

O1 N3 1.2211(19) . ?

O2 N3 1.217(2) . ?

O3 N4 1.209(2) . ?

O4 N4 1.208(2) . ?

O5 N5 1.200(2) . ?

O6 N5 1.195(2) . ?

O7 N6 1.200(2) . ?

O8 N6 1.213(2) . ?

O9 N7 1.205(2) . ?

O10 N7 1.206(2) . ?

O11 N8 1.203(3) . ?

O12 N8 1.201(3) . ?

O13 N9 1.199(2) . ?

O14 N9 1.210(2) . ?

N1 H1 0.78(3) . ?

N1 N2 1.3333(19) . ?

N1 C1 1.344(2) . ?

N2 C3 1.317(2) . ?

N3 C2 1.425(2) . ?

N4 C4 1.536(2) . ?

N5 C4 1.560(2) . ?

N6 C4 1.534(2) . ?

N7 C5 1.527(2) . ?

N8 C5 1.540(2) . ?

N9 C5 1.530(2) . ?

C1 C2 1.381(2) . ?

C1 C4 1.492(2) . ?

C2 C3 1.407(2) . ?

C3 C5 1.491(2) . ?

loop_

_geom_angle_atom_site_label_1

_geom_angle_atom_site_label_2

_geom_angle_atom_site_label_3

_geom_angle

_geom_angle_site_symmetry_1

_geom_angle_site_symmetry_3

_geom_angle_publ_flag

N2 N1 H1 122.0(17) . . ?

N2 N1 C1 113.52(14) . . ?

C1 N1 H1 124.5(17) . . ?

C3 N2 N1 105.30(13) . . ?

O1 N3 C2 117.84(14) . . ?

O2 N3 O1 124.42(15) . . ?

O2 N3 C2 117.73(14) . . ?

O3 N4 C4 117.51(16) . . ?

O4 N4 O3 128.36(18) . . ?

O4 N4 C4 114.11(15) . . ?

O5 N5 C4 115.03(15) . . ?

O6 N5 O5 129.62(17) . . ?

O6 N5 C4 115.36(15) . . ?

O7 N6 O8 127.59(16) . . ?

O7 N6 C4 117.46(15) . . ?

O8 N6 C4 114.90(14) . . ?

O9 N7 O10 126.95(19) . . ?

O9 N7 C5 116.26(15) . . ?

O10 N7 C5 116.78(16) . . ?

O11 N8 C5 114.04(17) . . ?

O12 N8 O11 128.25(19) . . ?

O12 N8 C5 117.70(19) . . ?

O13 N9 O14 128.19(17) . . ?

O13 N9 C5 117.10(16) . . ?

O14 N9 C5 114.70(15) . . ?

N1 C1 C2 105.15(14) . . ?

N1 C1 C4 124.35(15) . . ?

C2 C1 C4 130.50(15) . . ?

C1 C2 N3 127.48(14) . . ?

C1 C2 C3 105.40(14) . . ?

C3 C2 N3 127.05(14) . . ?

N2 C3 C2 110.62(14) . . ?

N2 C3 C5 118.72(14) . . ?

C2 C3 C5 130.66(14) . . ?

N4 C4 N5 108.76(13) . . ?

N6 C4 N4 104.74(13) . . ?

N6 C4 N5 103.99(13) . . ?

C1 C4 N4 112.03(13) . . ?

C1 C4 N5 112.24(13) . . ?

C1 C4 N6 114.46(13) . . ?

N7 C5 N8 109.67(14) . . ?

N7 C5 N9 105.20(13) . . ?

N9 C5 N8 106.81(13) . . ?

C3 C5 N7 113.53(13) . . ?

C3 C5 N8 111.89(14) . . ?

C3 C5 N9 109.32(13) . . ?

```
loop_
  _geom_hbond_atom_site_label_D
  _geom_hbond_atom_site_label_H
  _geom_hbond_atom_site_label_A
  _geom_hbond_distance_DH
  _geom_hbond_distance_HA
  _geom_hbond_distance_DA
  _geom_hbond_angle_DHA
  _geom_hbond_site_symmetry_A
  _geom_hbond_publ_flag
 N1 H1 O8 0.78(3) 2.18(2) 2.683(2) 123(2) . yes
 N1 H1 O14 0.78(3) 2.32(3) 2.999(2) 145(2) 3_656 yes

loop_
  _geom_torsion_atom_site_label_1
  _geom_torsion_atom_site_label_2
  _geom_torsion_atom_site_label_3
  _geom_torsion_atom_site_label_4
  _geom_torsion
  _geom_torsion_site_symmetry_1
  _geom_torsion_site_symmetry_2
  _geom_torsion_site_symmetry_3
  _geom_torsion_site_symmetry_4
  _geom_torsion_publ_flag
 O1 N3 C2 C1 -3.1(3) . . . . ?
 O1 N3 C2 C3 -179.53(18) . . . . ?
 O2 N3 C2 C1 176.45(17) . . . . ?
 O2 N3 C2 C3 0.0(3) . . . . ?
```

O3 N4 C4 N5 -17.5(2) . . . . ?

O3 N4 C4 N6 93.21(18) . . . . ?

O3 N4 C4 C1 -142.15(16) . . . . ?

O4 N4 C4 N5 163.95(15) . . . . ?

O4 N4 C4 N6 -85.35(17) . . . . ?

O4 N4 C4 C1 39.3(2) . . . . ?

O5 N5 C4 N4 70.6(2) . . . . ?

O5 N5 C4 N6 -40.6(2) . . . . ?

O5 N5 C4 C1 -164.84(17) . . . . ?

O6 N5 C4 N4 -109.4(2) . . . . ?

O6 N5 C4 N6 139.44(19) . . . . ?

O6 N5 C4 C1 15.2(2) . . . . ?

O7 N6 C4 N4 -14.45(19) . . . . ?

O7 N6 C4 N5 99.65(17) . . . . ?

O7 N6 C4 C1 -137.53(16) . . . . ?

O8 N6 C4 N4 167.99(14) . . . . ?

O8 N6 C4 N5 -77.91(17) . . . . ?

O8 N6 C4 C1 44.9(2) . . . . ?

O9 N7 C5 N8 -113.48(18) . . . . ?

O9 N7 C5 N9 131.98(17) . . . . ?

O9 N7 C5 C3 12.5(2) . . . . ?

O10 N7 C5 N8 66.6(2) . . . . ?

O10 N7 C5 N9 -47.9(2) . . . . ?

O10 N7 C5 C3 -167.41(17) . . . . ?

O11 N8 C5 N7 156.66(16) . . . . ?

O11 N8 C5 N9 -89.83(18) . . . . ?

O11 N8 C5 C3 29.8(2) . . . . ?

O12 N8 C5 N7 -23.9(2) . . . . ?

O12 N8 C5 N9 89.61(19) . . . . ?

O12 N8 C5 C3 -150.80(17) . . . . ?

O13 N9 C5 N7 134.26(17) . . . . ?

O13 N9 C5 N8 17.7(2) . . . . ?

O13 N9 C5 C3 -103.49(18) . . . . ?

O14 N9 C5 N7 -47.05(18) . . . . ?

O14 N9 C5 N8 -163.56(16) . . . . ?

O14 N9 C5 C3 75.21(19) . . . . ?

N1 N2 C3 C2 -0.10(19) . . . . ?

N1 N2 C3 C5 -179.36(15) . . . . ?

N1 C1 C2 N3 -176.16(17) . . . . ?

N1 C1 C2 C3 0.90(18) . . . . ?

N1 C1 C4 N4 -132.06(16) . . . . ?

N1 C1 C4 N5 105.23(18) . . . . ?

N1 C1 C4 N6 -13.0(2) . . . . ?

N2 N1 C1 C2 -1.05(19) . . . . ?

N2 N1 C1 C4 179.56(15) . . . . ?

N2 C3 C5 N7 112.85(17) . . . . ?

N2 C3 C5 N8 -122.36(17) . . . . ?

N2 C3 C5 N9 -4.3(2) . . . . ?

N3 C2 C3 N2 176.56(17) . . . . ?

N3 C2 C3 C5 -4.3(3) . . . . ?

C1 N1 N2 C3 0.7(2) . . . . ?

C1 C2 C3 N2 -0.5(2) . . . . ?

C1 C2 C3 C5 178.63(17) . . . . ?

C2 C1 C4 N4 48.7(2) . . . . ?

C2 C1 C4 N5 -74.0(2) . . . . ?

C2 C1 C4 N6 167.77(17) . . . . ?

C2 C3 C5 N7 -66.2(2) . . . . ?

C2 C3 C5 N8 58.5(2) . . . . ?

C2 C3 C5 N9 176.65(17) . . . . ?

C4 C1 C2 N3 3.2(3) . . . . ?

C4 C1 C2 C3 -179.76(16) . . . . ?

_shelx_res_file

;

TITL js323f_rm_a.res in P2(1)/n

js323f_rm.res

created by SHELXL-2018/3 at 11:22:11 on 28-Sep-2023

REM Old TITL JS323F_RM in P2(1)/n

REM SHELXT solution in P2(1)/n: R1 0.215, Rweak 0.123, Alpha 0.060

REM <I/s> 0.482 for 163 systematic absences, Orientation as input

REM Formula found by SHELXT: C18 N3 O7

CELL 1.54184 10.8108 10.7147 12.2728 90 103.218 90

ZERR 4 0.0006 0.0006 0.0009 0 0.006 0

LATT 1

SYMM 0.5-X,0.5+Y,0.5-Z

SFAC C H N O

UNIT 20 4 36 56

EQIV $1 1-X,-Y,1-Z

L.S. 4 0 0

PLAN 3

SIZE 0.05 0.07 0.22

TEMP 25

CONF

HTAB N1 O8

HTAB N1 O14_$1

list 4

```
MORE -1
BOND $H
fmap 2 53
acta
REM <olex2.extras>
REM <HklSrc "%.\\JS323F_RM.hkl">
REM </olex2.extras>

WGHT    0.062200    0.427700
FVAR       6.27010
O1    4    0.204302    0.425485    0.204129    11.00000    0.03968    0.06542 =
        0.08212    0.03869    0.00566    0.00955
O2    4    0.403308    0.427129    0.202135    11.00000    0.04310    0.05279 =
        0.05841    0.02035    0.00793   -0.00939
O3    4   -0.045972    0.326703    0.200957    11.00000    0.04190    0.09369 =
        0.05428    0.01955    0.00179    0.01559
O4    4    0.078536    0.174747    0.176733    11.00000    0.06844    0.06999 =
        0.04522   -0.01436    0.01344   -0.00745
O5    4   -0.025671    0.393519    0.418864    11.00000    0.05197    0.06033 =
        0.08834   -0.01477    0.02647    0.01148
O6    4    0.167453    0.455405    0.428655    11.00000    0.06037    0.04974 =
        0.12062   -0.02940    0.02183   -0.00749
O7    4   -0.058234    0.121933    0.360337    11.00000    0.03509    0.05766 =
        0.07135    0.00318    0.01261   -0.00757
O8    4    0.097643    0.132155    0.506538    11.00000    0.05323    0.06560 =
        0.04588    0.01244    0.01726    0.00055
O9    4    0.489304    0.185260    0.159098    11.00000    0.05567    0.09267 =
        0.05054   -0.01795    0.00597   -0.00517
O10   4    0.693725    0.198786    0.205238    11.00000    0.05104    0.10089 =
```

0.06542 0.01451 0.02753 0.01872

O11 4 0.586293 0.442764 0.427226 11.00000 0.06774 0.05451 =

0.08837 -0.02243 0.00671 -0.00782

O12 4 0.696551 0.425737 0.301394 11.00000 0.05951 0.06719 =

0.10958 0.02136 0.02375 -0.02057

O13 4 0.748131 0.225343 0.497142 11.00000 0.05066 0.08468 =

0.05884 0.00800 -0.01481 0.00272

O14 4 0.674140 0.061486 0.400472 11.00000 0.05212 0.04779 =

0.07229 0.01869 0.01582 0.01314

N1 3 0.318445 0.155337 0.443675 11.00000 0.03489 0.03759 =

0.03880 0.00897 0.00979 0.00199

H1 2 0.290145 0.112063 0.483270 11.00000 0.04618

N2 3 0.441052 0.151873 0.441199 11.00000 0.03315 0.04197 =

0.04025 0.00742 0.00654 0.00362

N3 3 0.313077 0.387276 0.235339 11.00000 0.03548 0.03948 =

0.04522 0.01140 0.00472 -0.00043

N4 3 0.040365 0.253751 0.231192 11.00000 0.03732 0.05580 =

0.03908 0.00253 0.00706 -0.00338

N5 3 0.080940 0.383292 0.406827 11.00000 0.04161 0.03905 =

0.05442 -0.00223 0.01027 0.00597

N6 3 0.042973 0.159303 0.411788 11.00000 0.03616 0.03911 =

0.04559 0.00228 0.01635 0.00339

N7 3 0.589064 0.207558 0.223332 11.00000 0.04277 0.04938 =

0.04499 0.00693 0.01154 0.00525

N8 3 0.626569 0.387757 0.357133 11.00000 0.03610 0.04254 =

0.06391 0.00494 -0.00074 -0.00551

N9 3 0.678939 0.172023 0.421055 11.00000 0.03096 0.05372 =

0.04853 0.01468 0.00759 0.00564

C1 1 0.250025 0.235840 0.369800 11.00000 0.03178 0.03275 =

```
        0.03474    0.00112    0.00569    0.00220
C2    1    0.336242    0.290080    0.316779    11.00000    0.03201    0.03397 =
        0.03688    0.00624    0.00467    0.00003
C3    1    0.453636    0.233588    0.364330    11.00000    0.03127    0.03414 =
        0.03555    0.00463    0.00455    0.00049
C4    1    0.110709    0.257007    0.354941    11.00000    0.03231    0.03505 =
        0.03771    0.00034    0.00824    0.00129
C5    1    0.581492    0.251357    0.340016    11.00000    0.02987    0.03763 =
        0.03966    0.00655    0.00361    0.00079
HKLF 4




REM  js323f_rm_a.res in P2(1)/n
REM wR2 = 0.1243, GooF = S = 1.086, Restrained GooF = 1.086 for all data
REM R1 = 0.0427 for 2565 Fo > 4sig(Fo) and 0.0477 for all 2960 data
REM 257 parameters refined using 0 restraints

END

WGHT      0.0622      0.4276

REM Highest difference peak  0.283,  deepest hole -0.235,  1-sigma level  0.053
Q1    1   0.5213  0.2403  0.3554  11.00000  0.05    0.28
Q2    1   0.1830  0.2390  0.3608  11.00000  0.05    0.28
Q3    1   0.3997  0.2739  0.3398  11.00000  0.05    0.25
;
_shelx_res_checksum              14675
```

```
_shelx_hkl_file
;
   0   0   1 35.3318 23.3780  30
   0   0   2 207633. 9862.46  30
   0   0   3 38.1125 73.0468  30
   0   0  -4 66809.3 3350.93  23
   0   0  -5-24.9748 84.1764  23
   0   0  -6 189.968 132.949  23
   0   0  -7-39.6431 127.398  23
   0   0  -8 25053.3 1650.87  23
   0   0  -9 58.2713 145.049  23
   0   0  -9 34.8730 57.8954  20
   0   0 -10 3821.10 304.503  20
   0   0 -11 90.2348 158.819  17
   0   0 -11 107.173 121.765  24
   0   0 -11 27.9042 66.5123  20
   0   0  11-66.8641 117.689  30
   0   0 -12 2165.97 217.185  20
   0   0  12 2082.23 215.561  29
   0   0 -13 91.8438 72.8519  20
   0   0  13-37.4453 67.1126  29
   0   0  14 9557.22 572.454  29
   0   0 -14 10905.8 577.206  20
   0   0 -15 4.47759 27.2997  20
   0   1   0 3.36484 16.7682  24
   0  -1   0 54.2168 28.3111   2
   0  -1  -1 3923.97 255.218  23
   0   1  -1 4147.32 242.778  23
   0  -1   1 4642.69 246.429  30
```

0	1	1	4722.09	258.604	30
0	-1	-1	4331.68	236.762	1
0	1	-2	806855.	37972.9	23
0	1	2	813537.	37979.4	30
0	-1	2	796190.	37968.2	30
0	1	-3	21284.1	1111.25	23
0	-1	3	19496.3	1090.82	30
0	1	3	20837.7	1104.66	30
0	1	-4	844447.	38729.7	23
0	1	4	829641.	38714.7	30
0	-1	4	786141.	38699.5	30
0	1	-5	35707.0	1951.95	23
0	-1	5	37268.6	1939.82	30
0	1	5	35290.4	1933.12	30
0	-1	-6	259382.	12615.8	23
0	1	-6	264152.	12601.5	23
0	-1	6	261955.	12345.3	29
0	1	6	265021.	12351.1	29
0	-1	6	245737.	12543.0	30
0	1	6	253525.	12564.8	30
0	-1	-7	-40.8257	109.758	23
0	1	-7	-38.9508	111.947	23
0	1	7	9.96594	50.6348	29
0	-1	7	-9.68885	48.2325	29
0	-1	7	-35.0949	128.578	30
0	1	7	36.0910	115.971	30
0	1	8	25742.3	1281.27	29
0	-1	8	24768.4	1277.06	29
0	-1	8	24644.0	1590.47	30

0	1	8	26002.2	1602.47	30
0	-1	-8	24955.6	1632.55	23
0	1	9	18313.7	925.483	29
0	-1	9	18814.0	1308.79	30
0	1	9	17334.8	1276.01	30
0	-1	9	18424.2	922.116	29
0	-1	-9	21444.4	1407.08	23
0	1	-9	14492.6	886.722	20
0	1	-10	11691.3	1268.46	17
0	-1	10	11426.6	685.133	29
0	1	10	11501.1	1041.83	30
0	-1	10	12114.2	1074.32	30
0	1	10	12555.8	698.817	29
0	1	-10	12113.3	1034.10	24
0	1	-10	10396.4	668.289	20
0	1	-11	621.241	121.846	20
0	1	-11	857.986	338.949	17
0	-1	-11	917.756	362.388	17
0	1	-11	354.116	182.329	24
0	-1	11	263.518	191.637	30
0	1	11	737.323	274.043	30
0	1	11	632.113	130.431	29
0	-1	11	914.481	146.767	29
0	1	12	3447.46	296.515	29
0	-1	12	4291.96	321.334	29
0	1	-12	3707.73	301.093	20
0	-1	13-60.3662		58.8396	29
0	1	13	12.4000	61.7315	29
0	1	-13	11.1815	60.1256	20

0 -1 14 1209.79 133.659 29

0 1 -14 1625.73 145.197 20

0 1 -15 522.610 58.7118 20

0 2 0 826341. 38467.9 24

0 -2 0 802378. 38493.4 2

0 2 -1 47091.1 2444.71 23

0 -2 -1 55865.1 2484.72 23

0 2 -1 56085.7 2530.71 24

0 -2 1 47469.9 2445.97 30

0 2 1 55954.4 2477.61 30

0 -2 -1 56529.7 2545.12 2

0 -2 -1 57849.5 2505.07 1

0 -2 -2 101574. 4725.49 23

0 2 -2 98657.2 4685.00 23

0 -2 2 98824.3 4684.22 30

0 2 2 99788.4 4708.22 30

0 -2 -2 93514.1 4664.28 1

0 -2 -3 168387. 7886.32 23

0 2 -3 169262. 7845.51 23

0 -2 3 171192. 7838.04 30

0 2 3 170705. 7862.16 30

0 -2 -3 154665. 7760.26 1

0 -2 -4-21.1959 101.037 23

0 2 -4 35.4324 69.7446 23

0 -2 4-48.9658 74.1932 30

0 -2 -4 44.2544 32.4311 1

0 -2 -5 15539.6 868.932 23

0 -2 5 14636.8 804.420 30

0 2 5 15787.1 837.565 30

0	2	-6	115556.	5751.81	23
0	-2	6	118927.	5730.93	30
0	2	6	108213.	5524.65	29
0	2	6	131679.	5784.52	30
0	-2	6	110455.	5502.49	29
0	2	-7	616.287	184.680	23
0	-2	7	381.068	90.8532	29
0	2	7	412.176	88.3977	29
0	-2	7	358.165	167.990	30
0	2	7	602.097	184.485	30
0	2	8	7467.72	445.728	29
0	-2	8	7339.40	440.099	29
0	-2	8	7039.20	672.557	30
0	2	8	7373.91	700.367	30
0	2	9	1998.67	202.805	29
0	-2	9	2236.71	388.888	30
0	2	9	2161.75	392.012	30
0	-2	9	1716.29	185.087	29
0	2	-9	1733.33	182.911	20
0	-2	-9	1954.25	191.074	20
0	2	-10	7405.62	987.733	17
0	2	10	6917.25	769.633	30
0	2	10	7557.09	470.247	29
0	-2	10	4671.68	666.450	30
0	-2	10	7804.52	472.284	29
0	2	-10	8412.39	828.810	24
0	2	-10	6942.37	448.187	20
0	-2	-10	7174.42	452.068	20
0	-2	-10	4275.73	452.738	21

0	2	-11	7572.69	911.581	17
0	2	-11	7854.63	824.008	24
0	-2	-11	6397.36	455.154	20
0	2	-11	6831.58	459.851	20
0	2	11	6377.08	771.218	30
0	-2	11	5003.87	696.748	30
0	-2	11	7677.40	479.352	29
0	2	11	7623.29	483.776	29
0	2	-12	214.399	80.5980	20
0	-2	-12	115.877	80.1508	20
0	2	12	238.049	90.4955	29
0	-2	12	254.961	83.4890	29
0	-2	-13	384.893	87.7686	20
0	2	-13	331.080	82.3486	20
0	-2	13	165.506	68.3740	29
0	2	13	224.394	78.7303	29
0	2	14	1513.29	145.895	29
0	2	-14	1976.54	151.526	20
0	-2	-14	1799.79	150.137	20
0	-2	-15	109.229	29.3855	20
0	3	0	10.4997	46.5146	24
0	-3	0	42.4038	65.8240	2
0	3	-1	258655.	11947.6	24
0	-3	-1	256921.	11865.6	23
0	3	1	243449.	11855.1	30
0	-3	-1	264439.	11937.8	1
0	-3	1	239124.	11973.8	2
0	-3	-1	243220.	11963.9	2
0	-3	-2	151635.	6984.23	23

0	3	-2	146511.	7043.68	24
0	-3	2	139104.	6924.86	30
0	3	2	152327.	6969.51	30
0	-3	-2	144573.	6969.22	1
0	-3	-3	304090.	14074.5	23
0	3	-3	295442.	14016.0	23
0	-3	3	303243.	14013.4	30
0	3	3	301244.	14048.8	30
0	-3	-3	281696.	13975.8	1
0	-3	-4	219062.	9662.77	23
0	3	-4	205932.	9586.31	23
0	-3	4	212802.	9583.62	30
0	3	4	208614.	9614.96	30
0	-3	-4	186479.	9454.73	1
0	-3	5	32822.4	1685.61	27
0	-3	-5	37933.6	1936.98	23
0	-3	5	37428.3	1847.90	30
0	3	5	35982.9	1868.77	30
0	-3	-6	2475.93	354.076	23
0	3	-6	1767.58	285.463	23
0	3	6	2141.73	301.660	30
0	-3	6	1564.58	151.557	29
0	-3	6	2439.13	301.613	30
0	3	6	1984.44	173.134	29
0	-3	-7	8794.41	753.728	23
0	-3	7	8454.23	463.293	29
0	3	7	8930.47	482.069	29
0	3	7	8615.01	709.049	30
0	-3	7	7928.81	670.017	30

0	-3	-8	34572.4	2044.28	23
0	3	8	35144.1	1606.49	29
0	-3	8	34118.0	1591.03	29
0	-3	8	23713.7	1809.64	30
0	3	8	30157.0	1925.04	30
0	-3	-8	27557.2	1563.28	20
0	-3	-9	248.572	81.6732	20
0	3	-9	57.8344	60.0637	20
0	-3	9	52.9685	107.720	30
0	3	9	395.430	207.001	30
0	3	-9	-68.1462	183.249	17
0	3	-9	308.465	188.106	24
0	-3	9	48.1350	58.7782	29
0	3	9	92.4793	79.5200	29
0	-3	-9	144.815	67.2832	15
0	-3	-10	2926.08	250.407	20
0	3	-10	2854.69	245.651	20
0	3	-10	3636.76	591.939	17
0	-3	10	3114.12	257.652	29
0	3	10	2879.44	259.472	29
0	3	10	2952.20	482.795	30
0	3	-10	3475.65	537.901	24
0	-3	-11	196.320	77.0778	20
0	3	-11	318.243	89.2648	20
0	3	11	114.943	77.9266	29
0	-3	11	265.761	80.5319	29
0	3	-12	470.012	96.7970	20
0	-3	-12	418.001	99.9337	20
0	-3	12	456.500	95.1664	29

0	3	12	383.874	96.3280	29
0	3	-13	775.303	103.538	20
0	-3	-13	808.138	110.334	20
0	-3	13	472.699	87.8080	29
0	3	13	569.005	101.985	29
0	-3	14	1272.99	103.524	10
0	3	-14	1479.99	113.333	20
0	-3	-14	1561.86	125.515	20
0	4	0	147391.	7051.68	24
0	-4	0	144918.	7110.81	2
0	4	-1	659731.	30681.3	24
0	-4	1	643084.	30713.1	2
0	-4	-1	639185.	30706.2	2
0	4	2	61214.4	2089.48	30
0	-4	2	55792.9	2291.43	2
0	4	-2	62097.3	2259.63	24
0	-4	-2	64662.7	2122.81	23
0	4	3	26538.6	988.115	30
0	-4	3	24358.3	883.817	30
0	-4	-3	27731.3	1017.96	23
0	4	-3	30000.0	1168.38	24
0	4	-4	-93.3794	100.437	17
0	4	4	42.1982	64.3491	30
0	-4	4	192.831	80.0173	30
0	4	-4	156.020	109.855	24
0	-4	-4	96.2987	100.883	23
0	-4	5	38328.7	1787.22	27
0	-4	5	39401.2	1926.06	30
0	4	5	39843.1	1988.31	30

0	-4	-5	42021.4	2042.61	23
0	-4	6	24680.5	1290.55	27
0	-4	-6	27194.2	1578.16	23
0	4	-6	28936.8	1666.94	24
0	4	6	24264.6	1503.61	30
0	-4	6	22114.7	1428.31	30
0	4	6	24527.8	1257.43	29
0	4	-7	10373.5	862.189	24
0	-4	-7	8903.48	780.720	23
0	-4	7	5520.98	595.153	30
0	4	7	9402.56	484.658	29
0	-4	7	7927.88	447.414	29
0	4	7	5584.60	621.753	30
0	4	-7	4328.66	640.938	17
0	-4	-8	25319.8	1151.10	21
0	-4	-8	18991.7	1038.07	20
0	4	-8	23332.4	1552.39	24
0	4	8	22584.4	1068.69	29
0	-4	8	20664.6	1040.11	29
0	4	8	16450.4	1322.59	30
0	-4	9	3661.80	609.409	30
0	-4	9	9615.63	524.705	29
0	4	9	3669.51	634.697	30
0	4	9	10442.9	555.272	29
0	-4	-9	8775.86	536.433	15
0	-4	-9	8974.49	525.444	20
0	4	-9	9468.66	524.678	20
0	-4	-10	3549.71	269.035	20
0	4	-10	3528.61	262.563	20

0	4	-10	4546.45	622.124	24
0	-4	-10	3297.75	268.164	15
0	-4	-10	5411.59	816.787	17
0	4	10	4220.77	296.870	29
0	-4	10	2953.13	255.592	29
0	-4	-11	2907.14	238.984	15
0	4	11	2815.30	250.390	29
0	-4	11	2330.91	224.893	29
0	4	-11	2835.70	236.838	20
0	-4	-11	3195.34	252.104	20
0	4	12	1386.59	161.858	29
0	-4	-12	1563.14	161.673	20
0	4	-12	1627.73	157.534	20
0	4	-13	2042.42	149.166	20
0	-4	-13	2203.44	163.298	20
0	5	0	19.2529	67.7769	24
0	-5	0	-51.5528	101.447	2
0	5	-1	8677.40	594.886	24
0	-5	-1	7536.24	600.265	2
0	-5	1	7839.18	621.826	2
0	5	-2	47275.4	2322.02	24
0	-5	2	41119.7	2108.54	28
0	-5	2	46109.1	2358.46	2
0	5	3	76106.7	2659.05	30
0	-5	3	75442.7	2549.44	28
0	-5	3	81993.0	2553.97	27
0	-5	3	81884.7	2970.71	2
0	-5	4	40473.3	2466.67	2
0	5	4	42496.8	2303.47	30

```
0   5  -5 104211. 4392.39  24
0   5   5 70179.3 4053.93  30
0  -5   6 18195.2 900.586  27
0   5  -6 18756.2 1275.88  24
0   5   6 16872.0 835.571  29
0  -5  -7-14.7073 68.4912   9
0   5   7 43.9777 109.465  30
0   5   7-81.2501 77.9209  29
0  -5   7 77.1807 58.2240   5
0   5  -7-59.0299 146.929  24
0  -5  -8 16593.7 807.200  20
0  -5   8 17006.8 823.852   5
0   5   8 18810.8 842.785  29
0  -5  -9 33.2660 55.2297  20
0   5   9 50.4107 58.7128  29
0  -5  -9 14.1259 70.3422  15
0  -5  -9 79.4477 161.469  17
0   5   9 56.1251 127.537  30
0   5  -9 69.5403 158.015  24
0  -5 -10 67.3250 58.1404  20
0  -5 -10-13.5363 68.7752  15
0   5  10-12.8089 63.7647  29
0  -5 -11 10.1967 51.8539  20
0   5  11 142.108 64.8400  29
0  -5 -11 11.5516 64.3296  15
0   5  12 894.622 116.135  29
0  -5  13-44.1531 46.1182  10
0  -5  13-18.2666 35.5724   4
0  -5 -14 11.8699 18.8439  11
```

0	5	-14	20.9027	25.8490	19
0	6	0	88456.8	4425.18	24
0	-6	0	87372.9	4261.87	28
0	-6	0	92108.0	4532.27	2
0	6	-1	10173.5	726.085	24
0	-6	1	10275.9	575.723	28
0	-6	1	10420.0	785.767	2
0	-6	-1	8910.30	747.049	2
0	6	-2	47109.3	2369.99	24
0	-6	2	43321.6	2119.57	27
0	-6	2	40484.1	2115.21	28
0	-6	2	43738.4	2398.50	2
0	6	-3	2796.84	355.910	24
0	-6	3	2175.34	344.140	2
0	-6	3	2136.65	191.888	27
0	-6	3	1826.34	189.959	28
0	6	-4	1194.24	260.446	24
0	-6	4	1156.41	273.250	2
0	-6	4	1443.02	159.404	27
0	6	-5	1435.01	311.623	24
0	-6	5	1063.27	272.019	2
0	-6	-6	1104.79	295.029	17
0	-6	6	1162.60	154.799	5
0	6	-6	1475.66	332.568	24
0	-6	-6	1584.58	183.585	9
0	-6	7	8723.37	931.126	2
0	-6	7	8296.00	535.532	5
0	6	-7	9502.38	924.915	24
0	-6	-7	9722.28	582.797	9

0 -6 8 23283.0 1285.75 5

0 6 -8 25450.2 1807.38 24

0 -6 8 28674.4 1902.62 2

0 -6 -8 24983.7 1335.86 9

0 -6 9 5471.64 376.222 5

0 -6 -9 5377.78 394.573 15

0 6 -9 4660.14 674.671 24

0 -6 9 5660.03 749.791 2

0 -6 -10 107.065 75.8835 15

0 -6 11 271.425 85.9861 4

0 -6 -11 577.250 107.085 15

0 -6 11 361.464 98.4924 10

0 -6 12 1058.09 131.366 10

0 -6 12 1016.63 117.558 4

0 -6 13 92.1242 38.1079 10

0 -7 0-39.4680 126.822 2

0 -7 0 11.5369 51.0997 28

0 7 -1 22146.5 1424.51 24

0 -7 1 22921.1 1503.82 2

0 -7 -1 24265.3 1246.54 28

0 -7 1 23934.6 1243.46 28

0 7 -2 44301.3 2439.27 24

0 -7 2 42083.3 2178.10 28

0 -7 2 45011.0 2179.86 27

0 -7 2 42059.0 2483.11 2

0 -7 3 1686.67 177.111 27

0 -7 3 1072.61 154.766 28

0 -7 3 944.048 261.028 2

0 7 -3 1488.36 286.603 24

0	-7	-4	67322.0	3232.44	3
0	-7	4	65245.9	3241.82	4
0	-7	4	64279.0	3251.44	10
0	-7	-4	64453.6	3266.13	8
0	-7	4	69384.2	3661.46	2
0	-7	4	68627.9	3251.32	27
0	-7	4	61804.7	3220.68	5
0	-7	-4	65103.7	3259.49	16
0	7	-4	69938.1	3625.74	24
0	-7	5	279.822	104.138	10
0	-7	-5	311.527	102.398	9
0	-7	5	310.101	104.346	27
0	7	-5	440.940	194.564	24
0	-7	5	272.445	88.2259	4
0	-7	5	260.858	86.1138	5
0	-7	5	172.783	166.175	2
0	-7	-5	485.403	103.569	3
0	7	-6	18518.2	1395.56	24
0	-7	-6	19262.4	1019.36	9
0	-7	6	17365.7	1015.54	10
0	-7	6	19067.0	1426.75	2
0	-7	6	17030.7	998.532	4
0	-7	-6	17480.9	975.330	3
0	-7	6	16917.0	978.216	5
0	7	-7	8365.70	875.210	24
0	-7	7	7479.50	535.440	10
0	-7	-7	7948.73	537.603	9
0	-7	7	7730.58	855.322	2
0	-7	7	7932.11	533.586	4

0	-7	7	7583.98	509.950	5
0	7	-8	13666.7	1249.84	24
0	-7	8	14249.8	1273.61	2
0	-7	-8	15985.8	896.305	9
0	-7	8	14561.7	896.106	10
0	-7	8	14814.0	853.334	5
0	-7	8	15390.6	883.398	4
0	-7	9	3631.57	320.050	10
0	-7	-9	3225.67	288.322	15
0	-7	9	3486.42	280.730	5
0	-7	9	3669.65	309.627	4
0	-7	-10	534.395	111.990	15
0	-7	10	547.891	127.617	10
0	-7	10	662.241	124.656	4
0	-7	-11	370.059	84.7231	15
0	-7	11	290.528	78.1203	4
0	-7	11	498.979	101.757	10
0	-7	12	135.027	65.5970	8
0	-7	12	122.340	53.6016	10
0	-7	-13	386.581	50.7676	11
0	7	-13	716.774	64.9693	19
0	-8	0	13.8597	58.0864	28
0	-8	0	75.5791	68.7071	4
0	-8	0	16.2449	71.9681	8
0	-8	1	18335.8	1299.62	2
0	-8	-1	18294.6	1037.05	8
0	-8	1	17735.1	1032.65	8
0	-8	1	18807.7	1004.36	27
0	-8	1	19882.5	1024.03	28

0	-8	-1	18951.4	1015.57	28
0	-8	-1	19845.7	1044.48	16
0	-8	1	16633.6	1007.04	4
0	-8	-1	17091.8	1017.22	4
0	-8	-1	17691.7	1010.92	3
0	-8	2	3850.96	508.191	2
0	-8	-2	4214.55	358.035	8
0	-8	2	4213.10	356.577	8
0	-8	-2	4562.80	342.159	28
0	-8	2	4797.08	345.280	27
0	-8	2	4203.05	335.285	28
0	8	-2	3556.47	472.441	24
0	-8	-2	4529.22	345.048	3
0	-8	-2	4236.50	352.866	4
0	-8	2	4519.98	349.679	4
0	-8	3	6050.76	434.083	4
0	-8	-3	5776.32	424.975	3
0	-8	-3	6154.38	453.959	4
0	-8	3	6110.00	451.575	8
0	-8	-3	6155.17	452.092	8
0	-8	3	6744.87	705.352	2
0	-8	3	6447.78	435.361	27
0	-8	3	5649.53	422.950	5
0	8	-3	5889.88	645.952	24
0	-8	4	4118.31	336.971	4
0	-8	-4	4440.90	335.478	3
0	-8	4	3438.33	332.321	10
0	-8	4	4367.06	359.341	8
0	-8	4	4082.46	555.992	2

0	-8	4	4399.94	345.149	27
0	-8	4	3505.81	320.400	5
0	-8	-4	4345.09	357.944	16
0	8	-4	4883.52	580.739	24
0	-8	5	1783.00	222.192	10
0	-8	5	1676.69	224.158	8
0	-8	-5	1614.24	207.880	9
0	-8	5	1475.55	193.813	5
0	8	-5	1708.63	362.230	24
0	-8	-5	1713.51	197.180	3
0	-8	5	1567.94	198.069	4
0	-8	5	1598.21	209.469	27
0	-8	5	1324.09	320.943	2
0	-8	6	1976.08	222.369	5
0	-8	6	1923.98	237.248	10
0	-8	-6	2465.51	255.895	9
0	8	-6	1646.89	375.137	24
0	-8	6	1821.09	216.957	4
0	-8	-6	1746.85	201.618	3
0	-8	6	1885.46	230.746	27
0	-8	-7	3022.06	284.751	9
0	-8	7	2758.12	282.082	10
0	-8	7	2881.01	272.330	4
0	-8	-7	2530.41	234.724	3
0	8	-7	3026.76	493.172	24
0	-8	7	2122.03	239.218	5
0	-8	8	35354.2	1832.47	10
0	-8	-8	33232.2	1788.35	15
0	-8	8	35874.3	1805.33	4

0 -8 -9 7625.86 488.122 15

0 -8 9 7305.39 504.930 10

0 -8 9 7498.19 482.882 4

0 -8 -10 7010.13 435.277 15

0 -8 10 7149.17 466.546 8

0 -8 10 7205.14 457.268 10

0 -8 10 6312.74 418.065 4

0 -8 -11 1340.49 135.136 15

0 -8 11 1640.79 163.960 8

0 -8 11 1580.08 153.273 10

0 8 -12 241.982 44.6280 19

0 -8 -12 226.136 45.2563 11

0 -8 12 207.724 46.9183 8

0 -9 0 27.4277 57.4557 27

0 -9 0-79.0816 80.3572 28

0 -9 0 18.1829 86.6609 16

0 -9 0-18.6183 88.7363 8

0 -9 0 103.572 76.4568 4

0 -9 0-16.5871 71.5084 3

0 -9 -1 4032.00 312.859 27

0 -9 -1 3850.64 327.646 28

0 -9 1 4074.21 337.278 28

0 -9 1 4001.81 324.367 27

0 -9 -1 4107.48 343.973 3

0 -9 -1 4537.15 363.004 4

0 -9 1 4013.72 344.186 4

0 -9 -1 4088.36 354.660 16

0 -9 -1 4200.09 363.245 8

0 -9 1 4039.57 354.507 8

0	-9	2	35758.1	1943.62	4
0	-9	-2	36594.2	1958.83	4
0	-9	-2	38417.6	1948.81	3
0	-9	-2	35400.5	1959.77	8
0	-9	2	38043.4	1974.25	8
0	-9	2	38799.2	1951.59	28
0	-9	-2	38725.2	1939.45	28
0	-9	2	37903.4	1934.62	27
0	-9	-3	470.211	121.542	3
0	-9	3	324.441	106.085	27
0	-9	-3	453.556	114.077	28
0	-9	3	638.690	141.812	8
0	-9	-3	290.415	122.854	8
0	-9	3	409.508	115.376	4
0	-9	4	3491.29	309.559	27
0	-9	-4	3162.90	291.036	28
0	-9	4	3559.43	328.594	10
0	-9	4	3443.32	327.407	8
0	-9	-4	3909.56	315.867	3
0	-9	4	3249.89	302.018	4
0	-9	-4	3291.71	326.113	16
0	-9	5	5913.55	437.173	27
0	-9	5	5719.88	442.325	10
0	-9	-5	5413.70	429.098	9
0	-9	5	5818.19	452.077	8
0	-9	-5	5230.03	402.560	3
0	-9	5	6133.98	433.165	4
0	-9	6	1804.68	225.193	8
0	-9	6	1218.42	194.411	10

0 -9 -6 1386.89 196.214 9

0 -9 6 1075.95 168.976 4

0 -9 -6 1480.52 175.282 3

0 -9 -7 18259.3 983.086 9

0 -9 7 17051.4 989.495 10

0 -9 7 17664.0 1003.41 8

0 -9 -7 15691.2 914.437 3

0 -9 7 17446.5 961.267 4

0 -9 8 720.298 139.482 8

0 -9 8 947.832 154.552 10

0 -9 -8 867.100 139.480 15

0 -9 8 765.607 124.326 4

0 -9 9 94.9582 80.4489 8

0 -9 9 180.272 67.5747 4

0 -9 9 39.1854 69.0515 10

0 -9 -9-10.8397 64.3173 15

0 -9 10 240.622 79.8917 8

0 -9 10 326.738 80.3571 10

0 -9 -10 396.886 78.0049 15

0 9 -10 379.950 64.8287 19

0 -9 -11 68.2730 28.5328 11

0 -9 11 114.051 43.9161 8

0 -10 0 16196.4 946.093 16

0 -10 0 16514.3 938.129 4

0 -10 0 16539.9 933.786 3

0 -10 0 14952.9 936.215 8

0 -10 0 15670.5 912.789 28

0 -10 0 15076.0 882.275 27

0 -10 1-19.5855 101.483 8

0	-10	-1	58.7602	93.3572	8
0	-10	1	-17.6467	84.1112	3
0	-10	-1	34.5881	76.6055	3
0	-10	1	101.883	72.4602	27
0	-10	-1	-15.9834	74.4333	28
0	-10	1	100.726	85.2985	28
0	-10	-1	25.0409	50.8996	27
0	-10	-1	-19.2183	82.8577	16
0	-10	1	-18.8846	85.8226	16
0	-10	1	17.7566	67.5183	4
0	-10	-1	91.6577	87.3752	4
0	-10	-2	7880.66	552.738	4
0	-10	2	7797.70	540.430	4
0	-10	-2	7702.57	534.375	3
0	-10	2	7683.19	553.054	8
0	-10	-2	8580.10	576.370	8
0	-10	2	8163.65	529.853	27
0	-10	-2	7915.11	528.531	28
0	-10	3	7913.62	546.842	4
0	-10	-3	8018.40	540.486	3
0	-10	3	8221.27	573.183	8
0	-10	-3	8508.88	556.819	9
0	-10	3	8613.44	547.545	27
0	-10	-3	7764.89	527.505	28
0	-10	-4	2104.67	225.059	3
0	-10	4	2148.20	229.338	4
0	-10	-4	2005.72	214.664	28
0	-10	4	2378.69	238.701	27
0	-10	-4	1838.44	225.002	9

0	-10	4	2348.26	254.718	8
0	-10	5	1686.57	196.404	4
0	-10	-5	1578.03	180.219	3
0	-10	-5	1343.25	167.759	28
0	-10	5	1429.90	206.006	8
0	-10	-6	2478.77	221.163	28
0	-10	-6	2486.49	237.263	15
0	-10	6	2727.49	269.006	10
0	-10	-6	2935.11	265.837	9
0	-10	6	2501.49	259.723	8
0	-10	6	2314.32	233.333	4
0	-10	-6	2816.12	232.040	3
0	-10	7	195.965	90.1339	8
0	-10	-7	299.074	96.4679	9
0	-10	7	252.896	103.237	10
0	-10	7	281.042	86.3376	3
0	-10	7	341.444	88.9074	4
0	-10	-7	214.274	66.5194	3
0	-10	8	12.4919	65.9607	8
0	-10	8	16.7379	46.5837	4
0	-10	-9	892.218	109.218	15
0	-10	9	1054.38	123.674	8
0	-10	10	502.620	65.5868	8
0	-11	0	36.3527	92.3957	8
0	-11	0	16.5000	78.6966	4
0	-11	0	65.3138	87.8463	3
0	-11	0	141.290	87.3805	9
0	-11	0	87.9739	89.4426	16
0	-11	-1	1235.97	189.143	16

0	-11	1	1605.02	204.288	16
0	-11	-1	1274.68	178.760	3
0	-11	1	1402.75	187.917	4
0	-11	1	1442.18	189.204	3
0	-11	1	1194.23	187.104	8
0	-11	-1	1291.41	176.656	9
0	-11	-1	1552.85	205.630	8
0	-11	1	1079.51	146.509	27
0	-11	-1	1489.11	174.610	28
0	-11	2	2249.00	240.065	16
0	-11	-2	2387.28	252.285	16
0	-11	2	1999.45	226.446	4
0	-11	-2	2084.52	227.383	3
0	-11	2	1796.62	230.541	8
0	-11	-2	2477.85	239.770	9
0	-11	2	2289.66	216.746	27
0	-11	-2	2099.31	211.828	28
0	-11	3	329.824	106.730	16
0	-11	-3	268.125	96.0830	3
0	-11	3	481.487	113.725	4
0	-11	-3	245.490	80.0446	28
0	-11	3	210.759	78.0820	27
0	-11	3	292.201	105.670	8
0	-11	-3	329.917	105.347	9
0	-11	4	4482.84	353.212	16
0	-11	-4	4599.95	331.251	15
0	-11	4	4432.27	351.644	3
0	-11	-4	4354.38	336.245	3
0	-11	4	4643.00	346.888	4

0	-11	4	5108.54	381.393	8
0	-11	-4	5474.85	375.425	9
0	-11	5	14.8242	73.8010	8
0	-11	-5	-10.7974	65.8361	15
0	-11	-5	31.1869	58.8215	3
0	-11	5	50.5650	65.5045	3
0	-11	5	57.7665	62.1275	4
0	-11	5	-12.4266	61.8670	16
0	-11	6	-29.7554	55.2028	16
0	-11	6	-31.1316	57.7559	3
0	-11	-6	15.1019	47.2989	3
0	-11	-6	108.039	67.6892	15
0	-11	6	-18.5676	52.5248	4
0	-11	6	-38.8480	70.8597	8
0	-11	-6	-11.8415	71.1860	9
0	-11	-7	4181.35	268.270	15
0	-11	7	4366.30	293.849	8
0	-11	8	113.244	52.8614	8
0	-12	0	799.373	130.615	3
0	-12	0	451.617	94.1613	9
0	-12	0	531.131	120.766	8
0	-12	0	696.436	127.869	16
0	-12	1	851.588	138.707	3
0	-12	-1	795.746	130.921	3
0	-12	-1	661.664	112.610	9
0	-12	1	781.356	137.622	8
0	-12	1	476.968	110.707	16
0	-12	-1	630.405	129.329	16
0	-12	2	260.609	96.9033	8

0	-12	-2	122.399	74.5871	9
0	-12	2	179.030	77.5411	4
0	-12	2	211.106	82.1788	16
0	-12	-2	80.6648	69.7491	3
0	-12	2	287.135	96.9511	3
0	-12	3	6777.18	432.805	8
0	-12	-3	6341.27	407.209	9
0	-12	3	6097.93	396.732	4
0	-12	-3	6330.28	401.913	3
0	-12	3	5965.79	404.495	16
0	-12	4	381.530	94.0418	8
0	-12	-4	264.437	82.2850	9
0	-12	4	220.815	66.0420	4
0	-12	4	177.306	66.8194	16
0	-12	-4	247.771	67.7195	3
0	-12	4	389.107	89.7322	3
0	-12	5	39.3957	60.0468	8
0	-12	-5	-16.8478	35.6372	3
0	-12	5	24.8808	51.2618	3
0	-12	5	-5.64464	33.4445	4
0	-12	6	236.279	61.6558	8
0	-12	6	167.287	48.2692	3
0	-12	-7	1709.50	117.797	15
0	-12	7	1997.79	133.957	8
0	-13	0	47.5997	48.4192	3
0	-13	1	9606.45	535.108	3
0	-13	-1	9482.27	528.279	3
0	-13	2	81.9386	54.1259	3
0	-13	-2	44.5908	43.5104	3

0	-13	-3	579.537	68.5117	3
0	-13	4	-4.66457	31.0818	3
1	0	-15	1817.33	140.636	20
-1	0	14	-9.73654	52.3539	29
1	0	-14	-9.37988	53.9183	20
-1	0	13	5585.20	393.640	29
1	0	-13	6093.96	402.893	20
1	0	-12	84.4883	88.2064	20
-1	0	12	-86.3408	94.7656	29
1	0	-11	6281.53	451.940	20
1	0	-11	6206.73	686.892	24
1	0	-10	-85.7065	174.181	17
1	0	-10	26.7868	63.8420	20
1	0	-10	49.5232	112.528	24
1	0	-9	10449.5	653.411	20
1	0	-9	12911.3	961.981	24
1	0	-7	2363.22	359.057	23
1	0	-6	230.840	138.588	23
1	0	-5	42921.3	2292.64	23
1	0	-4	703.288	146.920	23
-1	0	3	27416.0	1419.47	30
1	0	-1	6470.46	359.540	25
1	0	0	20.4644	24.9502	25
1	0	1	63475.0	3149.84	1
1	0	1	72433.3	3203.87	30
1	0	2	147.610	65.6357	30
1	0	3	9309.32	603.063	30
-1	0	-4	-21.3116	75.0229	23
-1	0	-5	2145.12	289.876	23

-1	0	-6	66.9516	117.876	23
-1	0	-7	24708.4	1563.75	23
-1	0	-8	49.8108	113.201	23
-1	0	-9	25813.1	1779.49	23
-1	0	-9	26148.0	1358.82	20
-1	0	-10	-26.1866	70.4068	20
1	0	10	66.5389	165.618	30
1	0	11	138.615	186.336	30
-1	0	-11	89.1268	181.137	17
-1	0	-11	13.5502	64.6048	20
-1	0	-11	55.1690	137.327	24
1	0	12	43.3419	71.9436	29
-1	0	-12	-88.4556	72.6607	20
1	0	13	393.215	93.2710	29
-1	0	-13	394.602	94.4273	20
1	0	14	8.36165	40.7495	29
-1	0	-14	7.15539	42.3973	20
1	1	-15	1216.28	106.468	20
-1	-1	14	655.661	97.7580	29
1	1	-14	702.229	102.743	20
-1	-1	13	-12.2204	60.8347	29
-1	1	13	12.5728	66.3852	29
1	1	-13	36.1069	64.7152	20
1	1	-12	3374.11	282.081	20
-1	1	12	3551.58	295.229	29
-1	-1	12	3143.93	283.825	29
-1	1	11	960.383	285.572	30
-1	-1	11	628.711	249.704	30
1	-1	-11	455.816	335.686	17

-1	1	11	733.767	134.293	29
-1	-1	11	668.662	134.721	29
1	1	-11	1009.26	269.343	24
1	1	-11	684.964	130.006	20
-1	-1	10	1026.38	318.133	30
-1	1	10	1515.87	183.166	29
-1	-1	10	1511.99	179.158	29
-1	1	10	988.563	307.621	30
1	1	-10	1228.58	436.890	17
1	-1	-10	1227.70	440.927	17
1	1	-10	1603.95	333.530	24
1	-1	-10	917.020	238.771	24
1	1	-10	1306.81	167.044	20
-1	1	9	10511.9	918.089	30
-1	1	9	11515.6	663.395	29
-1	-1	9	10718.7	913.462	30
-1	-1	9	11157.3	648.975	29
1	1	-9	9289.11	613.677	20
1	1	-9	10958.7	927.538	24
-1	-1	8	14155.2	849.035	29
-1	-1	8	14907.8	1077.63	30
-1	1	8	15813.6	842.874	29
-1	1	8	15120.3	1091.66	30
-1	1	7	36909.8	1958.35	30
-1	-1	7	38328.9	1970.32	30
-1	1	7	35448.9	1707.55	29
1	-1	-7	39922.2	2070.98	23
1	1	-7	36904.0	2015.66	23
-1	1	6	1787.55	265.858	30

-1	-1	6	1863.61	276.395	30
1	1	-6	1350.76	270.182	23
-1	1	5	1035.16	193.455	30
-1	-1	5	729.569	161.662	30
1	1	-5	1149.49	209.332	23
-1	1	4	198371.	9573.32	30
-1	-1	4	197818.	9560.36	30
1	1	-4	202085.	9587.19	23
-1	1	3	5107.98	353.351	30
-1	-1	3	4171.11	329.447	30
1	1	-3	4926.98	354.109	23
1	1	-2	51290.9	2537.74	17
1	-1	-1	4407.37	258.345	25
1	1	-1	4369.65	257.759	25
1	1	-1	4206.35	263.265	17
1	-1	-1	4389.54	288.696	17
1	-1	0	334.980	51.3705	25
1	1	0	403.602	56.1000	25
1	1	0	416.715	60.0347	17
1	-1	0	271.784	47.9665	17
1	-1	0	343.494	65.9896	2
1	-1	1	15429.2	837.784	1
1	-1	1	16921.1	844.397	30
1	1	1	16260.2	851.077	30
1	1	2	7386.45	481.353	30
1	-1	2	7824.57	479.299	30
-1	1	-2	8288.48	485.777	23
1	1	3	1391.25	188.165	30
1	-1	3	1659.61	191.593	30

-1	1	-3	1388.84	182.588	23
1	-1	4	65779.6	3362.19	30
1	1	4	68311.2	3384.57	30
-1	1	-4	66406.7	3368.14	23
1	1	5	6026.32	532.329	30
1	-1	5	7478.57	555.921	30
-1	1	-5	6522.34	541.190	23
-1	-1	-5	5933.74	525.955	23
1	1	6	201844.	9719.26	30
1	-1	6	200175.	9417.08	29
1	-1	6	200968.	9699.65	30
1	1	6	194311.	9417.58	29
-1	-1	-6	201376.	9712.39	23
-1	1	-6	197481.	9684.01	23
1	1	7	8374.10	737.742	30
1	1	7	8277.27	492.667	29
1	-1	7	7998.15	720.639	30
1	-1	7	8364.80	486.978	29
-1	1	-7	7544.88	697.795	23
-1	-1	-7	8115.54	733.872	23
1	1	8	1727.20	342.205	30
1	-1	8	1922.54	190.754	29
1	-1	8	1802.75	356.314	30
1	1	8	2064.42	191.123	29
-1	-1	-8	1632.97	331.708	23
-1	1	-8	1565.72	334.180	23
1	1	9	3057.99	499.985	30
1	1	9	3174.24	268.359	29
1	-1	9	2892.82	481.823	30

1	-1	9	3799.33	282.313	29
-1	1	-9	2687.91	241.218	20
-1	-1	-9	2821.28	493.007	23
-1	1	-9	1900.64	400.322	23
1	-1	10	1254.00	339.841	30
1	-1	10	2348.52	227.872	29
1	1	10	2007.94	422.720	30
1	1	10	2770.87	243.027	29
-1	-1	-10	1953.79	467.382	23
-1	1	-10	1975.85	201.304	20
1	1	11	10179.8	628.368	29
1	-1	11	11184.6	1058.53	30
1	-1	11	9960.14	619.704	29
1	1	11	8666.31	1018.11	30
-1	1	-11	9362.90	596.513	20
-1	1	-11	9758.13	941.597	24
-1	1	-12	1509.70	171.775	20
1	1	12	1481.06	180.670	29
1	-1	12	1678.97	190.661	29
-1	1	-13	6684.34	404.445	20
1	-1	13	5792.77	402.405	29
1	1	13	6392.72	414.986	29
1	-1	14	380.834	73.1390	29
-1	-1	-14	574.233	81.3815	20
-1	1	-14	573.756	82.1995	20
1	2	-15	531.077	59.4440	20
1	-2	-15	725.020	70.7291	20
-1	-2	14	160.803	55.6153	29
-1	2	14	190.756	59.8538	29

1	-2	-14	219.150	69.6966	20
1	2	-14	258.157	66.7642	20
-1	2	13	325.080	89.1477	29
-1	-2	13	317.030	81.6549	29
1	2	-13	449.358	99.1272	20
1	-2	-13	460.572	100.165	20
-1	-2	12	664.538	130.609	29
-1	2	12	783.472	138.555	29
1	2	-12	531.736	111.819	20
1	-2	-12	797.426	134.103	20
-1	2	11	317.599	183.736	30
-1	-2	11	123.019	188.651	30
1	2	-11	696.647	284.349	17
1	2	-11	482.841	213.461	24
1	2	-11	613.081	118.585	20
1	-2	-11	507.070	107.205	20
-1	2	11	500.196	121.380	29
-1	-2	11	320.893	106.227	29
-1	2	10	472.596	225.609	30
-1	-2	10	792.917	258.253	30
-1	-2	10	434.608	102.702	29
-1	2	10	551.474	122.570	29
1	2	-10	420.145	203.395	24
1	-2	-10	534.913	106.526	20
1	2	-10	318.658	90.9978	20
-1	2	9	1355.92	168.819	29
-1	2	9	1064.97	287.844	30
-1	-2	9	342.035	223.672	30
1	2	-9	910.747	380.901	17

1	2	-9	1101.49	290.894	24
1	2	-9	767.582	128.212	20
1	-2	-9	883.742	139.249	20
-1	-2	9	1065.20	150.498	29
-1	2	8	1031.56	150.344	29
-1	-2	8	1015.48	139.446	29
-1	-2	8	720.627	220.370	30
-1	2	8	1256.78	276.623	30
1	2	-8	1040.84	271.791	24
1	2	-7	3617.31	547.418	17
1	2	-7	4192.39	467.259	24
1	-2	-7	5271.07	539.632	23
-1	2	7	2128.35	264.969	29
-1	-2	7	2785.05	237.877	29
-1	2	7	4042.32	428.364	30
-1	-2	7	3900.77	413.965	30
-1	-2	6	2671.69	306.437	30
-1	2	6	2510.39	308.542	30
1	-2	-6	2391.52	236.630	1
1	-2	-6	2915.60	369.561	23
-1	-2	6	2639.37	187.951	27
-1	2	5	12003.0	872.012	30
1	-2	-5	12866.4	922.579	23
-1	-2	4	241494.	10845.2	30
-1	2	4	232027.	10860.5	30
1	-2	-4	204683.	10785.4	1
1	-2	-4	239755.	10898.8	23
-1	-2	3	27042.7	1347.98	30
-1	2	3	26238.2	1357.07	30

1	-2	-3	26236.7	1343.05	1
1	2	-3	26592.7	1399.14	24
1	-2	-3	30779.5	1407.43	23
1	2	-2	94162.0	4113.02	17
1	-2	-2	89202.6	4167.10	17
1	-2	-2	105851.	4136.33	1
-1	2	2	101033.	4115.87	30
1	2	-1	13852.6	615.675	25
1	2	-1	13757.4	687.780	24
-1	2	1	12540.2	581.635	24
1	-2	-1	12092.4	647.439	17
-1	-2	1	12632.7	636.795	2
1	-2	-1	14590.8	682.181	1
1	2	0	51047.6	1916.89	25
1	2	0	48064.4	1981.85	24
1	-2	0	46216.0	1895.57	25
-1	-2	0	47792.1	1921.98	2
-1	-2	-1	2175.21	168.871	23
1	-2	1	1757.56	137.807	25
1	2	1	1936.24	159.987	30
-1	2	-2	85921.3	4336.72	23
-1	-2	-2	93335.6	4374.26	23
1	2	2	90417.1	4367.59	30
1	-2	2	90667.3	4346.11	30
-1	2	-3	149154.	7869.68	23
-1	-2	-3	166858.	7921.90	23
1	2	3	173872.	7925.78	30
1	-2	3	171348.	7903.76	30
-1	-2	-4	139641.	6745.57	23

-1	2	-4	126124.	6699.93	23
1	2	4	138647.	6734.75	30
1	-2	4	147987.	6726.07	30
-1	-2	-5	124523.	5918.72	23
-1	2	-5	105395.	5827.22	23
1	-2	5	128424.	5895.66	30
1	2	5	120190.	5911.83	30
-1	-2	-6	66187.1	3437.83	23
-1	2	-6	56065.0	3354.12	23
1	-2	6	61701.9	3135.25	29
1	-2	6	70718.2	3440.44	30
1	2	6	73485.7	3473.15	30
1	2	6	67642.4	3149.99	29
-1	2	-7	437.090	176.931	23
1	-2	7	249.375	153.352	30
1	2	7	522.530	193.719	30
1	2	7	400.744	83.5465	29
1	-2	7	376.777	78.1793	29
1	-2	8	4264.43	529.746	30
1	-2	8	4984.43	318.278	29
1	2	8	4815.98	570.031	30
1	2	8	4080.04	307.826	29
1	-2	9	7145.86	783.918	30
1	2	9	8522.61	527.776	29
1	-2	9	9553.45	530.448	29
1	2	9	7799.21	822.513	30
-1	-2	-9	8750.18	506.633	20
-1	2	-9	8457.51	502.540	20
1	2	10	15446.3	873.429	29

1	2	10	13687.2	1241.41	30
1	-2	10	17258.2	878.784	29
-1	2	-10	15025.9	847.790	20
-1	-2	-10	14871.7	846.829	20
1	-2	11	190.535	89.0521	29
1	2	11	136.716	84.1241	29
-1	-2	-11	395.302	107.058	20
-1	2	-11	129.235	73.0804	20
1	-2	12	52.9059	60.1279	29
1	2	12	55.5999	64.7466	29
-1	2	-12	23.4648	65.3187	20
-1	-2	-12	12.0000	63.3816	20
1	2	13	22.4802	56.0231	29
1	-2	13	73.4435	59.4248	29
-1	2	-13	73.5467	54.5424	20
-1	-2	-13	76.8537	56.1463	20
1	2	14	-15.2688	38.7977	29
-1	2	-14	39.7293	39.1247	20
-1	-2	-14	94.3998	49.5420	20
-1	3	14	79.4515	45.9614	29
1	-3	-14	99.0531	47.4265	20
1	3	-14	157.021	49.2942	20
1	-3	-13	1590.00	162.638	20
-1	-3	13	1198.41	139.905	29
-1	3	13	1386.01	157.963	29
1	-3	-12	11278.2	682.158	20
1	3	-12	12787.1	691.725	20
-1	-3	12	11343.2	676.701	29
-1	3	12	11776.2	693.443	29

-1	-3	11	1111.99	312.755	30
-1	-3	11	3409.89	260.477	29
-1	3	11	3381.60	272.417	29
-1	3	11	2068.89	427.624	30
1	-3	-11	3741.99	644.403	17
1	-3	-11	2932.50	254.955	20
1	3	-11	2532.82	240.184	20
1	3	-11	2425.22	448.912	24
1	3	-10	259.798	86.7308	20
1	-3	-10	145.353	69.9877	20
-1	3	10	59.3817	135.037	30
-1	-3	10	-221.586	275.819	30
1	-3	-10	175.361	235.787	17
1	3	-10	-69.2911	243.966	17
1	-3	-10	212.472	83.3645	15
-1	-3	10	139.760	75.4830	29
-1	3	10	70.0750	74.2051	29
1	3	-10	64.3969	113.473	24
1	-3	-9	4251.11	698.153	17
1	-3	-9	3295.64	275.586	15
1	3	-9	3493.86	529.005	24
1	-3	-9	3148.36	269.498	20
1	3	-9	3142.67	262.178	20
-1	3	9	3794.20	517.432	30
-1	3	9	4128.83	298.080	29
-1	-3	9	3321.49	270.549	29
-1	-3	9	2742.76	434.211	30
1	3	-8	34822.9	2093.59	17
1	3	-8	32144.5	2004.04	24

-1	3	8	33139.5	1584.17	29
-1	-3	8	26484.6	1795.95	30
-1	-3	8	30526.2	1560.29	29
-1	3	8	30267.8	1874.58	30
1	-3	-8	26413.3	1597.95	21
1	3	-7	12334.1	942.210	17
-1	3	7	13403.2	859.012	30
-1	3	7	11573.8	601.555	29
-1	-3	7	12388.3	805.040	30
1	3	-6	12963.3	797.932	17
-1	-3	6	11996.8	469.335	27
1	-3	-6	16650.2	881.259	23
-1	-3	6	12630.4	668.988	30
-1	3	6	4719.86	428.374	29
-1	3	6	12393.4	698.322	30
-1	3	5	63258.9	3062.74	30
-1	-3	5	65180.1	3032.10	30
1	3	-5	47339.4	3050.44	17
-1	-3	5	57409.3	2871.12	27
1	-3	-5	56825.9	2922.81	1
1	3	-5	64544.4	3155.44	24
1	-3	-5	71031.0	3159.72	23
-1	3	4	41081.4	1968.30	30
-1	-3	4	38110.5	1918.12	30
1	-3	-4	36338.0	1887.29	1
1	3	-4	37325.7	2027.57	24
1	-3	-4	41576.4	2013.70	23
-1	3	3	941653.	41387.0	30
-1	-3	3	999999.	41351.9	30

1	-3	-3	925845.	41366.5	1
1	3	-3	936107.	41467.8	24
1	-3	-2	275.419	103.615	17
1	3	-2	431.378	74.7795	17
-1	3	2	558.591	96.4848	30
-1	-3	2	511.323	78.7292	30
1	-3	-2	790.515	162.458	2
1	-3	-2	736.630	118.759	1
1	3	-2	651.607	131.284	24
1	-3	-1	16656.8	371.013	17
1	3	-1	19080.9	555.973	24
-1	3	1	16104.0	323.330	24
1	-3	-1	18754.9	539.481	1
1	-3	-1	17410.6	590.383	2
-1	-3	1	17475.3	469.185	2
1	3	0	53864.5	2566.18	25
1	3	0	55444.1	2673.29	24
-1	-3	0	53417.1	2633.11	2
1	-3	0	55085.6	2709.85	2
-1	3	0	56891.5	2617.49	24
-1	-3	0	49525.2	2573.12	23
1	3	1	22869.8	1152.40	25
1	-3	1	21205.7	1116.12	25
-1	-3	-1	24382.2	1169.97	23
-1	3	-1	21744.8	1113.99	23
1	-3	1	24061.3	1251.45	1
1	-3	1	23575.2	1274.66	2
1	3	2	19753.7	985.644	30
-1	-3	-2	18373.6	976.807	23

-1	3	-2	17100.2	918.559	23
1	-3	2	16059.5	922.857	25
-1	-3	-3	243854.	12513.9	23
-1	3	-3	260092.	12468.3	23
1	-3	3	271927.	12496.9	30
1	3	3	272038.	12541.2	30
-1	3	-4	3450.66	321.777	23
-1	-3	-4	3803.89	368.251	23
1	-3	4	3531.99	339.468	30
1	3	4	3683.50	362.947	30
-1	3	-5	3831.79	394.257	23
-1	-3	-5	5506.58	497.643	23
1	3	5	3985.70	274.711	29
1	3	5	5007.85	470.381	30
1	-3	5	5024.89	439.969	30
-1	-3	-6	27112.8	1579.30	23
1	3	6	26511.6	1555.46	30
1	-3	6	27907.3	1540.71	30
1	-3	6	24142.0	1251.60	29
1	3	6	27263.6	1272.10	29
-1	-3	-7	17735.5	1217.69	23
1	3	7	19939.3	1250.35	30
1	3	7	18176.6	890.850	29
1	-3	7	17318.7	874.784	29
1	-3	8	10055.7	504.424	29
1	3	8	7315.55	755.134	30
1	3	8	10037.4	511.807	29
-1	3	-8	8610.53	471.250	20
1	-3	8	7753.54	451.469	7

-1	-3	-8	9926.28	866.650	23
1	-3	9	1271.59	328.843	30
1	3	9	2963.62	259.180	29
1	-3	9	2911.14	249.576	29
1	3	9	1725.07	390.096	30
-1	-3	-9	2561.51	225.864	20
-1	3	-9	2788.99	234.039	20
-1	-3	-9	3696.22	535.269	23
1	-3	10	6167.09	427.569	29
1	3	10	6354.36	442.444	29
-1	-3	-10	6549.69	423.669	20
-1	3	-10	6728.64	423.141	20
-1	3	-10	6793.65	779.434	24
-1	3	-11	5955.11	393.308	20
-1	-3	-11	5747.98	391.669	20
1	-3	11	5414.98	392.566	29
1	3	11	5663.80	406.574	29
1	-3	12	82.4962	60.2826	29
1	3	12	219.484	88.2823	29
-1	-3	-12	154.652	73.9366	20
-1	3	-12	188.867	69.4492	20
1	3	13	442.772	92.1061	29
-1	3	-13	733.986	93.9904	20
-1	-3	-13	572.758	89.8418	20
1	-3	14	2525.24	160.735	10
-1	-3	-14	2833.11	170.553	20
-1	4	13	94.8903	52.8631	29
-1	-4	13	263.780	65.0981	10
1	-4	-13	339.366	74.8210	20

1	-4	-13	75.5902	89.2447	21
1	4	-13	320.572	68.4786	20
-1	4	12	-11.9184	65.2200	29
1	-4	-12	-11.0845	49.0981	20
1	4	-12	-10.3739	50.5568	20
1	-4	-11	3886.82	310.130	15
-1	4	11	4225.11	320.395	29
-1	-4	11	3515.17	294.657	29
1	4	-11	4265.35	307.761	20
1	-4	-11	4283.33	314.822	20
1	4	-10	73.5780	63.5467	20
1	-4	-10	12.7366	56.4232	20
-1	4	10	26.9199	73.6609	29
-1	-4	10	-11.5550	68.4714	29
1	-4	-10	56.2872	71.5017	15
1	4	-10	67.6815	119.123	24
-1	4	10	-58.8076	103.506	30
-1	4	9	11757.5	1337.52	30
-1	-4	9	9665.81	1268.82	30
-1	4	9	27424.7	1213.56	29
-1	-4	9	26098.6	1184.72	29
1	-4	-9	29786.4	1981.65	17
1	-4	-9	23835.2	1205.56	15
1	-4	-9	22618.4	1179.62	20
1	4	-9	23100.6	1175.02	20
1	4	-9	25943.0	1722.84	24
1	-4	-8	24201.3	1685.90	17
1	-4	-8	17148.6	949.251	15
-1	4	8	13444.7	1151.99	30

-1	-4	8	10425.7	1062.13	30
-1	4	8	20969.0	964.321	29
-1	-4	8	18054.1	920.618	29
1	-4	-8	17136.8	925.287	20
1	4	-7	124.717	186.398	17
-1	4	7	755.989	215.807	30
-1	-4	7	688.646	178.686	30
1	4	-7	984.628	264.882	24
-1	-4	7	572.072	88.2898	29
-1	-4	7	999.489	137.152	27
-1	4	7	501.503	107.980	29
1	-4	-6	2192.96	446.328	17
1	4	-6	1682.41	281.320	17
-1	4	6	1865.35	274.708	30
-1	-4	6	1794.72	248.987	30
1	4	-6	1549.94	300.043	24
-1	-4	6	2391.37	193.012	27
-1	-4	5	66392.1	3312.78	27
1	-4	-5	68353.2	3362.50	1
1	-4	-5	73920.5	3712.15	17
1	4	-5	63558.3	3460.69	17
1	4	-5	74575.7	3659.82	24
-1	-4	5	66213.6	3413.68	30
-1	4	5	74252.6	3494.90	30
-1	-4	4	68494.4	3557.87	27
-1	-4	4	69510.8	3561.51	28
1	4	-4	80473.1	3657.57	17
1	-4	-4	73661.6	3647.81	1
1	4	-4	79870.3	3843.29	24

-1	4	4	79521.2	3696.97	30
-1	-4	4	79357.7	3634.81	30
1	-4	-3	1623.47	245.046	17
1	-4	-3	2138.21	225.770	1
1	4	-3	2502.48	295.414	24
-1	4	3	2314.99	221.008	30
-1	-4	3	2415.97	179.970	30
-1	-4	2	32269.3	1455.18	2
1	-4	-2	34774.6	1454.34	1
1	-4	-2	32372.8	1531.63	2
1	4	-2	33446.0	1496.13	24
-1	4	2	35244.3	1338.14	30
1	-4	-1	85839.9	3456.61	2
1	-4	-1	88947.0	3411.59	1
-1	-4	1	81046.4	3346.81	2
-1	4	1	76322.3	3175.68	30
1	4	-1	87231.2	3408.02	24
-1	4	1	78219.1	3246.13	24
1	4	0	14170.1	695.545	24
-1	4	0	13662.4	609.126	24
1	-4	0	11903.5	717.743	2
-1	-4	0	12869.5	644.574	2
1	-4	0	14599.2	730.485	1
1	4	1	47805.0	2437.26	25
-1	4	-1	52643.9	2543.93	24
-1	-4	-1	51356.6	2456.21	23
1	-4	1	50377.4	2629.32	2
1	-4	2	8297.89	494.251	25
1	4	2	9805.97	565.305	25

1	-4	2	9497.13	698.241	2
-1	-4	-2	10255.8	576.879	23
-1	4	-2	8265.43	473.755	23
-1	4	-2	10106.6	641.468	24
-1	4	-3	40587.5	2064.63	23
-1	-4	-3	42074.5	2161.82	23
1	4	3	45662.7	2189.10	30
1	-4	3	39294.1	2086.60	25
1	-4	4	8604.47	496.838	27
1	4	4	8924.58	643.466	30
-1	4	-4	7929.79	557.207	23
-1	-4	-4	8836.28	645.725	23
1	-4	5	18826.0	1143.50	30
1	4	5	18483.2	1188.27	30
1	4	5	19828.7	963.162	29
1	-4	6	11642.9	580.918	29
1	4	6	10235.7	831.944	30
1	-4	6	8277.68	745.759	30
1	4	6	14151.4	617.514	29
-1	-4	-6	13022.7	901.540	23
1	-4	7	3314.19	454.620	30
1	-4	7	6600.01	330.310	29
1	4	7	7024.35	354.433	29
1	4	7	4116.38	516.914	30
-1	4	-7	980.530	302.497	23
-1	-4	-7	5372.91	582.672	23
1	-4	8	10925.2	622.670	7
1	-4	8	10264.8	613.667	5
-1	-4	-8	11162.4	972.438	23

-1	-4	-8	10210.9	612.857	20
1	4	8	12486.2	655.184	29
1	-4	8	11373.4	627.347	29
1	-4	9	-50.9441	146.440	25
-1	4	-9	68.0318	154.600	24
-1	-4	-9	126.321	181.546	23
-1	4	-9	66.2248	57.2503	20
-1	-4	-9	93.2371	63.8303	20
1	4	9	193.267	87.6422	29
1	-4	9	134.211	62.0466	29
-1	-4	-9	-25.0014	58.2706	15
1	4	9	123.552	166.102	30
1	-4	10	1548.10	178.229	29
1	4	10	1829.75	201.965	29
-1	-4	-10	1677.74	179.562	20
-1	4	-10	1857.30	184.484	20
-1	-4	-10	1799.42	184.850	15
-1	-4	-11	648.694	108.828	20
-1	4	-11	697.479	107.626	20
1	-4	11	382.489	90.7433	29
1	4	11	727.938	125.516	29
-1	4	-12	2157.97	174.658	20
-1	-4	-12	2670.61	197.682	20
1	4	13	934.941	124.003	29
-1	-4	-13	2625.21	158.776	20
1	-5	-14	790.801	80.1381	11
1	5	-14	1490.48	109.737	19
-1	-5	13	299.508	59.3565	10
-1	5	13	42.2766	38.9527	29

-1	5	12	1892.68	155.203	29
1	-5	-12	-26.5274	79.7409	21
1	-5	-11	6936.66	429.217	15
1	-5	-11	6248.32	400.502	20
1	-5	-10	75.4624	187.827	17
1	-5	-10	72.5242	80.7305	15
1	-5	-10	34.6155	52.4384	20
-1	5	10	-74.9314	85.1298	29
1	-5	-9	3387.47	611.930	17
1	-5	-9	3437.43	283.164	15
-1	5	9	3754.40	275.615	29
1	-5	-9	3248.53	251.578	20
-1	5	8	2719.75	227.791	29
-1	5	8	1036.86	275.096	30
1	5	-8	3840.48	546.916	24
1	-5	-8	2219.93	228.084	15
1	-5	-8	2467.17	235.470	3
1	-5	-7	18233.6	931.349	9
-1	-5	7	17168.7	915.817	27
1	5	-7	18455.0	1321.59	24
-1	5	7	16052.4	861.122	29
1	-5	-7	19897.7	1412.16	17
-1	-5	6	1167.23	151.086	27
1	5	-6	1250.92	290.955	24
-1	5	6	829.925	110.165	29
-1	5	6	846.604	192.261	30
1	-5	-6	785.511	294.751	17
-1	-5	5	78.1456	60.1477	27
1	-5	-5	84.0587	104.681	17

-1	5	5	154.400	114.043	30
1	5	-5	43.0577	107.239	24
-1	-5	4	48661.7	2527.30	2
1	-5	-4	46518.8	2359.20	1
-1	-5	4	45377.6	2250.35	27
-1	-5	4	45330.5	2254.02	28
-1	5	4	46448.9	2354.31	30
1	5	-4	52175.5	2573.43	24
-1	5	3	37967.6	1838.14	30
1	5	-3	35827.0	2012.34	24
1	-5	-3	39420.9	1938.77	1
-1	-5	3	35449.8	1982.48	2
1	5	-2	108767.	4979.25	24
-1	-5	2	106922.	4936.05	2
1	-5	-2	121012.	4968.03	1
1	5	-1	140462.	6699.17	24
-1	-5	1	131330.	6650.74	2
1	-5	-1	138456.	6755.87	2
1	5	0	80968.4	4112.68	24
-1	5	0	80876.8	4025.78	24
1	-5	0	83084.1	4190.16	2
-1	-5	0	83030.7	4090.68	2
-1	5	-1	2057.32	246.536	24
1	-5	1	2058.42	303.658	2
1	-5	2	6689.16	577.656	2
-1	-5	-2	5435.96	394.911	23
-1	5	-2	5991.12	497.231	24
1	-5	2	5691.83	346.158	27
1	-5	2	5462.09	346.950	28

1	5	3	2689.29	281.133	25
1	-5	3	2761.74	218.543	27
-1	-5	-3	2673.41	297.745	23
-1	5	-3	3640.37	390.252	24
1	-5	3	3030.79	406.910	2
1	-5	4	3378.06	263.539	27
1	-5	4	3642.27	486.289	2
-1	-5	-4	3692.83	381.191	23
-1	5	-4	4075.23	466.499	24
1	-5	5	77656.5	3372.96	2
-1	5	-5	80313.0	3268.75	24
1	5	6	16406.5	1693.70	30
1	5	6	38685.3	1587.44	29
-1	-5	-6	24710.7	1804.19	23
-1	5	-6	45374.9	2173.54	24
1	5	7	5259.50	316.123	29
-1	-5	-7	5190.30	344.001	9
1	-5	7	4718.59	308.722	5
1	-5	7	4249.96	300.566	7
1	-5	8	5539.93	403.383	5
-1	5	-8	6854.67	783.515	24
1	5	8	6384.49	419.219	29
-1	-5	-8	6275.38	443.605	9
-1	-5	-8	5574.19	388.335	20
-1	-5	-9	13176.4	720.694	20
1	-5	9	12692.3	725.699	5
-1	-5	-9	13202.9	743.584	15
1	5	9	11665.7	1044.93	25
1	5	9	11797.4	728.797	29

-1	-5	-10	8382.33	481.915	15
-1	-5	-10	7715.13	456.059	20
1	5	11	-11.6460	54.2387	29
-1	-5	-11	-58.0743	59.0193	15
-1	-5	-11	28.3835	45.1035	20
1	5	12	271.522	69.3905	29
1	-5	13	265.537	58.5760	4
1	-5	13	350.385	66.0322	10
-1	-6	13	188.688	43.2549	10
-1	-6	12	29.1714	49.4222	10
-1	-6	11	1065.19	150.001	10
1	-6	-11	877.550	136.960	15
-1	-6	11	956.658	129.985	4
-1	-6	10	7292.88	481.131	4
1	-6	-10	7583.02	490.073	15
-1	-6	10	6817.59	491.066	10
1	6	-9	4313.25	624.587	24
1	-6	-9	3833.17	323.951	15
1	-6	-8	1779.28	203.230	3
-1	-6	8	2097.40	420.227	2
1	-6	-8	2664.64	464.580	17
-1	-6	8	2001.01	203.718	5
1	-6	-8	1995.87	218.384	15
1	6	-8	1235.51	339.197	24
1	6	-7	2149.37	410.079	24
1	-6	-7	2270.89	230.644	9
-1	-6	7	1858.19	380.135	2
-1	-6	7	1489.10	195.822	27
-1	-6	7	1676.57	188.366	5

1	-6	-7	1855.37	379.895	17
1	-6	-7	1872.16	200.503	3
1	-6	-6	16969.9	953.773	3
1	-6	-6	18449.1	982.427	9
-1	-6	6	17455.8	1302.95	2
-1	-6	6	18113.5	977.913	27
-1	-6	6	15875.4	933.369	5
1	-6	-6	16559.1	1256.91	17
1	6	-6	18811.5	1356.86	24
-1	-6	5	4509.49	327.821	27
1	6	-5	4564.24	558.440	24
-1	-6	5	4034.32	511.156	2
1	-6	-5	3502.59	479.177	17
-1	-6	4	1360.70	273.027	2
1	6	-4	1435.37	280.263	24
-1	-6	3	22189.9	1147.68	27
1	6	-3	22361.1	1402.17	24
-1	-6	3	20441.2	1347.41	2
-1	-6	2	1874.62	278.620	2
1	6	-2	1638.34	278.934	24
-1	-6	1	5106.83	342.177	28
1	-6	-1	4945.32	326.600	28
1	6	-1	5854.84	517.970	24
-1	-6	1	5252.27	475.247	2
1	-6	-1	5160.80	531.091	2
1	-6	0	72964.6	3613.88	28
-1	6	0	73483.5	3738.13	24
1	-6	0	80243.7	3963.06	2
-1	-6	0	79243.9	3832.67	2

1	-6	1	6399.07	399.593	27
1	-6	1	6757.29	410.017	28
-1	6	-1	7184.45	544.939	24
1	-6	1	7280.05	647.395	2
1	-6	2	13386.9	704.331	27
1	-6	2	11705.7	698.758	28
-1	6	-2	13094.7	887.037	24
1	-6	2	13922.5	997.637	2
1	-6	3	6618.49	420.818	27
1	-6	3	6345.42	655.858	2
-1	6	-3	6537.91	604.996	24
1	-6	4	20339.1	1099.41	27
-1	6	-4	21641.5	1392.11	24
1	-6	4	19909.7	1442.33	2
-1	6	-5	50.6162	115.209	24
1	-6	5	120.597	137.176	2
-1	-6	-5	163.341	77.3104	9
1	-6	5	142.213	64.2188	7
1	-6	5	125.106	83.8413	27
1	-6	5	198.357	79.8257	5
1	-6	6	28620.0	1918.79	2
-1	6	-6	30694.0	1855.53	24
-1	-6	-6	27988.8	1404.82	9
1	-6	6	24869.6	1370.64	5
1	-6	6	25297.2	1365.50	7
1	-6	7	10842.2	1085.98	2
-1	6	-7	11480.7	1046.06	24
1	-6	7	10383.3	622.604	5
-1	-6	-7	11393.7	658.625	9

1	-6	8	10066.2	1065.76	2
-1	6	-8	11467.3	1056.39	24
1	-6	8	10676.1	623.141	5
-1	-6	-8	10437.5	643.178	9
-1	6	-9	487.782	224.806	24
1	-6	9	314.626	226.949	2
-1	-6	-9	467.444	109.161	15
1	-6	9	400.660	100.565	5
-1	-6	-10	168.788	71.8379	15
1	-6	10	155.775	67.1724	5
-1	-6	-11	2505.82	202.631	15
1	-6	11	2344.34	224.240	4
1	-6	12	-19.2785	59.5796	10
1	-6	12	8.40660	47.5615	4
1	-6	13	2062.85	144.655	10
1	7	-13	164.942	41.9620	19
1	-7	-13	89.1547	36.1342	11
-1	-7	12	331.215	69.3312	10
-1	-7	12	296.912	74.0666	8
-1	-7	11	-11.6467	62.6365	10
1	-7	-11	-42.8148	72.9726	15
-1	-7	10	3572.22	302.991	10
-1	-7	10	3506.64	276.317	4
1	-7	-10	3846.63	301.790	15
-1	-7	9	543.507	126.582	10
-1	-7	9	428.069	100.904	4
1	-7	-9	541.421	124.423	15
1	7	-8	5187.90	673.568	24
1	-7	-8	4720.55	363.236	15

-1	-7	8	4793.59	376.372	10
-1	-7	8	4208.54	341.495	4
1	-7	-8	3915.46	330.289	3
1	-7	-7	73.4420	76.1523	3
-1	-7	7	17.5909	92.9187	27
1	-7	-7	17.7339	93.6730	9
-1	-7	7	88.5504	82.5109	10
-1	-7	7	86.9154	75.1037	5
-1	-7	7	44.9160	74.5793	4
1	7	-7	67.2261	136.647	24
1	-7	-6	6865.22	475.558	9
-1	-7	6	6462.97	473.801	10
1	7	-6	6600.12	759.421	24
-1	-7	6	6989.71	480.746	27
-1	-7	6	6467.62	451.386	5
-1	-7	6	6754.42	457.516	4
1	-7	-6	6177.76	449.398	3
-1	-7	5	513.028	118.023	10
1	-7	-5	433.121	105.857	3
-1	-7	5	440.375	166.891	2
-1	-7	5	552.353	112.561	5
-1	-7	5	656.997	117.532	4
1	-7	-4	36633.1	1846.71	3
1	-7	-4	33439.7	1860.23	4
1	-7	-4	35347.9	1846.92	9
-1	-7	4	35095.1	1851.73	10
-1	-7	4	38052.1	2157.13	2
-1	-7	4	37241.8	1852.27	27
-1	-7	4	34582.4	1831.35	5

1	7	-4	38450.0	2229.18	24
1	-7	-4	38125.2	1872.90	16
-1	-7	3	13360.9	738.036	27
-1	-7	3	11346.1	925.392	2
1	7	-3	12018.6	981.843	24
-1	-7	2	-12.8641	61.3128	28
1	-7	-2	21.6789	49.2590	28
1	7	-2	-40.8173	82.9523	24
-1	-7	2	72.2186	82.0462	2
-1	-7	1	411.063	96.0191	28
1	-7	-1	605.908	96.5332	28
1	7	-1	409.191	162.131	24
1	-7	-1	270.393	190.306	2
-1	-7	0	3037.31	254.464	28
1	-7	0	3018.13	230.409	27
1	-7	0	2774.76	233.463	28
1	-7	0	3008.73	429.390	2
1	-7	1	3179.50	237.528	27
1	-7	1	2517.56	227.764	28
1	-7	1	3442.32	457.741	2
1	-7	2	2198.18	203.318	27
1	-7	2	2453.57	217.330	28
-1	7	-2	1843.95	299.525	24
1	-7	2	2173.87	381.527	2
1	-7	3	6750.09	460.672	4
-1	-7	-3	6825.57	469.371	8
1	-7	3	6339.80	456.988	10
1	-7	3	8170.97	781.039	2
1	-7	3	7219.47	451.798	27

-1	-7	-3	6326.86	456.012	16
-1	7	-3	7074.58	659.596	24
-1	-7	-4	14001.4	787.947	22
-1	7	-4	14884.1	1111.29	24
1	-7	4	16069.9	1228.10	2
1	-7	4	14510.2	825.625	27
1	-7	4	14229.3	814.690	5
-1	-7	-4	14805.8	850.141	16
1	-7	4	14027.8	837.802	4
-1	-7	-4	14154.8	804.459	3
-1	-7	-5	30332.3	1588.45	9
1	-7	5	31910.8	2061.96	2
-1	7	-5	33092.5	2004.99	24
1	-7	5	30799.0	1589.54	27
1	-7	5	29719.7	1592.36	4
1	-7	5	28403.3	1559.07	5
1	-7	6	-70.6080	189.828	2
-1	7	-6	-122.681	176.299	24
1	-7	6	174.862	95.6930	4
1	-7	6	-14.5087	70.7143	5
-1	-7	-6	48.9695	90.8560	9
1	-7	7	9478.50	1022.59	2
-1	-7	-7	10729.4	654.330	9
1	-7	7	10102.2	656.939	4
-1	7	-7	9656.18	982.444	24
1	-7	7	10028.4	628.122	5
1	-7	8	27256.5	2024.29	2
-1	-7	-8	28939.8	1507.58	9
-1	7	-8	28805.7	1980.57	24

1	-7	8	28489.8	1524.70	4
1	-7	8	27629.5	1482.32	5
1	-7	9	365.649	118.263	10
-1	-7	-9	346.779	104.240	15
1	-7	9	249.058	85.1932	5
1	-7	9	324.763	109.177	4
1	-7	10	2024.80	186.012	5
1	-7	10	2220.98	228.537	10
1	-7	10	2336.10	223.032	4
-1	-7	-10	2035.27	197.029	15
1	-7	11	979.665	132.099	10
-1	-7	-11	826.809	102.805	15
1	-7	11	720.336	110.319	4
1	-7	12	56.7675	46.1728	10
1	-7	12	-9.71436	29.6190	4
1	-7	12	40.4456	51.9886	8
1	-8	-12	706.580	79.5197	11
-1	-8	12	548.792	69.5386	8
1	8	-12	748.773	80.9049	19
1	-8	-11	2477.41	192.093	15
1	8	-11	2340.36	190.495	19
-1	-8	11	2127.31	184.875	10
-1	-8	11	2023.35	190.481	8
1	-8	-10	23.1421	73.4924	15
-1	-8	10	191.099	83.9537	10
-1	-8	10	13.5886	73.1302	8
1	-8	-9	207.041	98.8572	15
-1	-8	9	172.235	77.3791	4
-1	-8	9	391.250	116.348	10

-1	-8	9	312.501	105.010	8
-1	-8	8	10620.6	662.591	10
1	-8	-8	9500.36	606.140	3
-1	-8	8	10459.7	623.556	4
1	-8	-8	10551.1	640.830	15
-1	-8	7	5160.94	402.671	10
-1	-8	7	4537.59	402.220	8
1	-8	-7	5365.22	404.875	9
-1	-8	7	5069.18	372.393	4
1	-8	-7	4795.89	367.102	3
1	8	-7	3947.62	607.588	24
-1	-8	6	1476.41	202.538	10
1	-8	-6	1734.71	209.991	9
1	8	-6	1117.90	313.943	24
-1	-8	6	1402.37	204.649	8
-1	-8	6	1466.77	182.665	4
1	-8	-6	1449.65	185.480	3
-1	-8	5	8456.04	556.882	27
-1	-8	5	7187.67	544.033	10
-1	-8	5	8056.50	559.877	8
1	8	-5	8612.50	862.340	24
-1	-8	5	8174.71	532.299	4
1	-8	-5	8613.06	548.518	3
-1	-8	4	14446.8	828.336	4
1	-8	-4	13387.4	851.634	4
1	-8	-4	14449.8	836.638	3
-1	-8	4	13682.9	849.284	8
1	-8	-4	15529.7	856.994	9
-1	-8	4	13599.4	847.556	10

-1	-8	4	15833.8	856.472	27
1	-8	-4	13345.1	852.370	16
1	8	-4	14447.2	1175.56	24
1	8	-3	-51.3140	104.349	24
-1	-8	3	83.7848	78.1170	10
1	-8	-3	46.3455	76.9922	3
1	-8	-3	181.976	73.6326	28
-1	-8	3	80.7105	75.2573	28
-1	-8	3	244.333	95.7261	27
-1	-8	3	101.306	80.5573	8
1	-8	-3	120.540	89.2990	16
-1	-8	3	57.4738	65.3997	4
1	-8	-3	-17.2674	68.0564	4
1	8	-2	46.6381	95.0635	24
-1	-8	2	227.320	87.6114	10
1	-8	-2	121.013	80.2031	3
-1	-8	2	259.897	88.3340	27
1	-8	-2	101.984	67.7065	28
-1	-8	2	184.338	85.8087	28
-1	-8	2	146.730	83.1550	8
-1	-8	2	199.266	78.2287	4
1	-8	-2	115.590	82.5270	4
1	-8	-2	180.683	91.7116	16
1	-8	-1	1162.35	163.187	3
-1	-8	1	866.977	142.550	28
1	-8	-1	1091.48	142.434	28
1	-8	-1	1031.48	130.256	27
-1	-8	1	880.270	148.869	8
1	-8	-1	926.455	152.974	4

-1	-8	1	1240.53	156.774	4
1	-8	-1	1046.66	154.526	16
1	-8	0	16134.6	1204.20	2
1	-8	0	15476.7	854.861	28
-1	-8	0	16166.2	880.175	28
1	-8	0	15426.4	841.084	27
-1	-8	0	14602.3	864.938	4
1	-8	0	14736.6	879.313	4
-1	-8	0	14764.7	882.416	8
1	-8	1	27209.1	1500.34	10
-1	-8	-1	28608.3	1515.68	8
1	-8	1	28424.3	1520.49	8
1	-8	1	29657.6	1885.62	2
-1	-8	-1	28609.9	1504.86	28
1	-8	1	28272.1	1484.21	28
1	-8	1	30630.9	1480.33	27
1	-8	1	26977.7	1477.52	5
-1	-8	-1	28745.7	1506.87	4
-1	-8	-1	27942.0	1487.61	3
1	-8	1	27812.9	1509.04	4
-1	-8	-1	28817.0	1523.36	16
1	-8	2	1063.85	161.101	10
1	-8	2	980.721	278.223	2
-1	-8	-2	919.992	141.655	3
1	-8	2	899.987	136.978	27
-1	-8	-2	743.564	143.809	8
1	-8	2	1025.64	160.733	8
1	-8	2	1016.48	151.096	5
1	-8	2	787.980	142.244	4

-1	-8	-2	800.224	140.690	4
-1	-8	-2	947.580	151.970	16
-1	-8	-3	8519.42	575.815	8
1	-8	3	8339.63	563.730	10
1	-8	3	8271.01	572.436	8
1	-8	3	8730.68	885.899	2
1	-8	3	8553.58	544.045	27
-1	-8	-3	8619.49	571.298	16
1	-8	3	8257.69	559.623	4
-1	-8	-3	8240.56	536.033	3
1	-8	4	9335.06	602.038	4
-1	-8	-4	8249.76	554.837	3
1	-8	4	9395.53	622.414	8
1	-8	4	8899.74	920.918	2
1	-8	4	9419.27	588.616	27
1	-8	4	8521.41	575.250	5
-1	-8	-4	10019.9	624.167	16
-1	8	-4	9187.31	848.177	24
-1	-8	-5	17.0106	84.8250	9
-1	-8	-5	147.441	75.9958	3
1	-8	5	136.590	138.883	2
1	-8	5	139.134	92.6878	8
1	-8	5	67.6036	82.5115	27
-1	8	-5	170.524	129.247	24
1	-8	5	94.3294	87.6410	5
1	-8	5	159.695	90.2876	4
1	-8	6	7746.65	880.556	2
1	-8	6	8527.92	574.975	10
-1	-8	-6	8222.11	555.408	9

1	-8	6	8589.59	570.336	4
1	-8	6	8419.51	546.373	5
-1	8	-6	6605.21	788.552	24
1	-8	7	7249.63	511.077	4
-1	-8	-7	6984.23	495.858	9
1	-8	7	6905.16	512.353	10
1	-8	7	7510.10	494.097	5
1	-8	8	282.887	113.064	10
-1	-8	-8	286.569	102.618	9
1	-8	8	466.807	118.774	4
1	-8	8	220.669	88.6297	5
1	-8	9	77.3230	83.1640	10
1	-8	9	-40.9946	82.1596	4
-1	-8	-9	119.941	81.7697	15
1	-8	10	640.945	113.698	10
1	-8	10	632.789	123.396	8
1	-8	10	545.590	97.7457	4
-1	-8	-10	482.182	90.7626	15
-1	-8	-11	2310.33	167.270	15
1	-8	11	2789.86	201.153	10
1	-8	11	2591.79	200.731	8
-1	-8	-12	6.22258	17.3281	11
1	-8	12	32.9167	26.7789	8
-1	-9	11	-10.9608	37.3624	8
1	-9	-11	14.8463	32.2054	11
1	9	-11	31.1270	27.1186	19
1	-9	-10	3005.51	227.500	15
-1	-9	10	2775.83	226.701	8
-1	-9	10	3015.43	229.706	10

1	9	-10	2806.70	209.014	19
-1	-9	9	224.989	88.7123	8
1	-9	-9	449.452	102.757	15
1	9	-9	400.719	83.5521	19
-1	-9	9	351.278	102.034	10
-1	-9	8	412.580	116.401	8
-1	-9	8	327.294	80.2133	4
1	-9	-8	263.722	80.5849	3
1	9	-8	425.972	91.9968	19
-1	-9	8	501.274	119.267	10
-1	-9	7	2589.04	234.736	4
1	-9	-7	2437.19	235.250	3
1	9	-7	2464.46	223.232	19
-1	-9	7	2594.74	265.133	8
-1	-9	7	2535.19	266.117	10
1	-9	-7	2716.27	267.087	9
1	-9	-6	11484.9	722.711	28
-1	-9	6	14706.8	808.761	27
1	-9	-6	12316.2	758.921	15
1	-9	-6	12994.4	766.483	3
-1	-9	6	13237.9	758.831	4
-1	-9	6	13012.1	795.153	8
-1	-9	6	13393.3	801.516	10
1	9	-5	553.863	217.560	24
1	-9	-5	569.353	128.562	3
-1	-9	5	428.241	125.930	27
1	-9	-5	302.560	92.8804	28
1	-9	-5	498.212	137.988	9
-1	-9	5	435.446	123.335	10

-1	-9	5	365.674	103.512	4
-1	-9	5	550.587	137.901	8
1	-9	-4	268.802	93.4166	3
1	-9	-4	367.611	96.2878	28
-1	-9	4	660.330	143.323	27
1	-9	-4	436.305	116.120	9
-1	-9	4	264.781	121.158	10
-1	-9	4	528.986	135.400	8
-1	-9	4	218.608	92.6461	4
1	-9	-4	347.979	119.027	16
-1	-9	3	2693.80	262.964	27
1	-9	-3	2352.75	227.542	28
-1	-9	3	2381.61	254.820	28
-1	-9	3	2253.43	252.810	8
1	-9	-3	1960.19	234.456	9
1	-9	-3	2293.62	247.793	3
-1	-9	3	1881.27	223.205	4
1	-9	-3	2529.05	271.988	4
1	-9	-3	2627.47	270.629	16
1	-9	-2	1332.15	168.140	28
-1	-9	2	1561.05	195.122	27
-1	-9	2	1305.80	189.831	28
1	-9	-2	1640.66	210.025	16
-1	-9	2	1248.28	188.216	8
1	-9	-2	1188.86	181.576	4
-1	-9	2	1434.52	188.650	4
1	-9	-2	1295.96	181.473	3
1	-9	-1	32198.0	1631.21	28
-1	-9	1	32748.3	1647.93	27

-1	-9	1	31593.9	1658.99	28
1	-9	-1	28341.7	1660.36	8
-1	-9	1	31365.7	1670.36	8
1	-9	-1	31822.2	1672.79	4
1	-9	-1	30280.5	1651.88	3
-1	-9	1	29775.1	1640.76	4
1	-9	-1	33485.1	1678.91	16
1	-9	0	545.170	115.898	28
-1	-9	0	552.946	127.708	28
1	-9	0	648.745	109.843	27
1	-9	0	583.581	132.084	16
1	-9	0	509.839	129.824	8
-1	-9	0	657.029	143.536	8
1	-9	0	359.239	119.638	4
-1	-9	0	430.221	120.448	4
1	-9	0	646.166	136.265	3
-1	-9	0	547.878	127.300	3
1	-9	1	1009.20	150.201	28
-1	-9	-1	873.695	151.279	28
1	-9	1	787.325	128.595	27
-1	-9	-1	727.647	154.789	16
1	-9	1	743.467	155.578	8
-1	-9	-1	682.406	144.859	8
1	-9	1	715.976	145.479	4
-1	-9	-1	681.837	141.126	4
1	-9	1	647.717	242.066	2
-1	-9	-1	778.831	139.912	3
1	-9	2	1888.81	382.848	2
-1	-9	-2	1388.35	186.122	28

1	-9	2	1480.72	182.688	27
1	-9	2	1083.15	178.893	10
-1	-9	-2	1426.29	203.656	8
1	-9	2	1493.46	205.971	8
-1	-9	-2	1484.25	188.403	3
1	-9	2	1635.10	212.326	4
1	-9	2	1474.53	196.364	3
-1	-9	-2	1533.80	201.718	4
-1	-9	-2	1738.01	222.941	16
-1	-9	-3	1170.60	177.685	3
1	-9	3	1069.65	306.302	2
-1	-9	-3	1398.70	190.618	28
1	-9	3	1385.58	179.732	27
1	-9	3	1392.04	196.846	10
1	-9	3	1266.94	198.739	8
1	-9	3	1409.50	193.053	4
-1	-9	-3	1379.74	198.826	16
-1	-9	-4	724.875	135.102	3
1	-9	4	589.662	132.107	27
1	-9	4	845.052	161.827	8
-1	-9	-4	887.285	162.674	16
-1	-9	-4	750.929	149.017	9
1	-9	4	633.300	142.749	4
1	-9	4	628.038	137.393	5
-1	-9	-5	2333.90	252.770	9
1	-9	5	2351.89	270.484	8
1	-9	5	2637.67	267.150	4
-1	-9	-5	2287.39	224.238	3
1	-9	5	2415.43	251.244	5

-1	-9	-6	-16.6015	82.6440	9
1	-9	6	-37.0880	92.3135	10
1	-9	6	-34.0943	77.4685	4
1	-9	6	78.8935	84.8413	5
1	-9	6	-19.5432	95.2389	8
1	-9	7	93.3369	79.1096	4
1	-9	7	11.7797	61.1297	3
1	-9	7	72.8429	88.8104	8
-1	-9	-7	106.383	83.2320	9
1	-9	7	207.984	99.6848	10
1	-9	8	16.0320	78.1808	8
-1	-9	-8	72.4038	74.6431	15
1	-9	8	15.2944	80.8061	10
1	-9	8	132.504	75.0212	4
1	-9	9	-26.0037	73.5603	8
1	-9	9	-40.3844	59.8213	4
-1	-9	-9	-9.73257	62.5488	15
1	-9	9	86.9574	75.7385	10
1	-9	10	-55.9123	73.3467	8
1	-9	10	-12.0487	35.1656	4
-1	-9	-10	33.8377	43.4921	15
-1	9	-10	9.83814	31.2151	19
1	-9	10	17.7967	54.9997	10
1	-9	11	143.476	37.9332	8
-1	-10	10	91.2931	35.1308	8
-1	-10	9	643.231	97.8184	8
-1	-10	8	1003.59	139.421	8
1	-10	-7	2520.57	223.693	15
-1	-10	7	2504.83	240.084	8

1	-10	-7	2193.40	203.510	3
1	-10	-6	145.614	88.3526	15
-1	-10	6	119.514	69.9494	4
1	-10	-6	143.757	86.2133	3
-1	-10	6	32.7949	80.0707	8
1	-10	-5	381.578	118.518	9
-1	-10	5	546.080	136.630	8
1	-10	-5	506.293	114.739	15
-1	-10	5	563.951	129.928	27
1	-10	-5	359.016	101.688	3
-1	-10	5	340.779	94.0573	4
-1	-10	4	5399.54	420.911	4
1	-10	-4	6911.99	462.944	3
1	-10	-4	6711.70	465.639	9
-1	-10	4	6073.60	462.040	8
-1	-10	4	6727.61	462.925	27
1	-10	-4	5931.18	409.199	28
1	-10	-3	56.7118	90.1052	16
1	-10	-3	34.0765	86.5734	3
-1	-10	3	112.274	91.1963	8
-1	-10	3	-15.5880	74.3030	4
1	-10	-3	-16.7847	80.0061	9
-1	-10	3	99.6888	87.7335	27
1	-10	-3	13.1216	54.9870	28
1	-10	-2	75.3605	87.7289	16
1	-10	-2	122.764	90.8679	3
-1	-10	2	-51.9690	88.0120	3
-1	-10	2	-16.1824	63.6850	4
1	-10	-2	-19.0780	82.2465	4

-1	-10	2	94.3700	89.9548	8
1	-10	-2	19.6674	87.1108	8
1	-10	-2	-16.3723	74.4003	9
-1	-10	2	-64.6673	78.7840	27
1	-10	-2	69.2951	59.7472	28
-1	-10	2	-53.7851	87.3682	28
1	-10	-1	7555.90	544.704	16
1	-10	-1	8318.46	562.324	4
-1	-10	1	7749.95	534.999	3
-1	-10	1	7560.96	527.762	4
1	-10	-1	8504.32	552.352	3
-1	-10	1	8102.05	557.447	8
1	-10	-1	7246.12	543.475	8
1	-10	-1	6995.81	512.869	9
1	-10	-1	7875.60	514.709	28
-1	-10	1	8618.78	537.368	27
-1	-10	1	8324.56	552.103	28
1	-10	0	118.784	96.6555	8
-1	-10	0	190.222	96.8240	8
1	-10	0	150.194	97.4518	4
-1	-10	0	119.206	83.1956	4
-1	-10	0	152.403	99.0201	3
1	-10	0	142.840	92.6985	3
-1	-10	0	149.086	78.9364	27
-1	-10	0	121.532	86.6267	28
1	-10	0	180.184	87.8448	28
1	-10	0	18.3449	89.5914	16
1	-10	1	812.272	161.365	8
-1	-10	-1	876.546	163.929	8

1	-10	1	1055.28	170.367	4
-1	-10	-1	904.168	159.815	4
-1	-10	-1	1023.37	162.237	3
-1	-10	-1	939.760	143.574	27
-1	-10	-1	1024.64	160.987	28
1	-10	1	1006.88	143.575	27
1	-10	1	1259.70	183.194	16
1	-10	2	1258.55	188.303	16
-1	-10	-2	1329.00	198.204	16
-1	-10	-2	1174.31	170.354	3
1	-10	2	1520.61	199.670	3
1	-10	2	1095.41	177.480	4
1	-10	2	1069.34	180.087	8
-1	-10	-2	1178.77	189.076	8
1	-10	2	1190.44	160.881	27
-1	-10	-2	1239.58	179.587	28
-1	-10	-3	2599.37	284.904	16
1	-10	3	2485.33	268.236	4
1	-10	3	2988.69	281.944	3
-1	-10	-3	2297.63	245.282	3
1	-10	3	3126.20	303.206	8
1	-10	3	2880.52	259.641	27
-1	-10	-3	2967.67	273.805	28
1	-10	4	-19.2191	85.1256	8
-1	-10	-4	13.5377	64.5219	3
1	-10	4	-15.8959	83.9300	3
1	-10	4	17.1838	74.0807	4
-1	-10	-4	-15.5351	70.5955	28
-1	-10	-4	50.1688	79.7027	9

1	-10	5	2021.32	211.207	3
1	-10	5	1460.56	199.017	4
-1	-10	-5	1523.77	172.457	3
-1	-10	-5	1904.61	216.661	9
1	-10	5	2144.91	239.353	8
-1	-10	-5	1646.06	192.033	28
1	-10	6	185.591	97.1347	8
1	-10	6	181.666	78.0235	3
1	-10	6	28.7326	67.1186	4
-1	-10	-6	86.8023	91.9660	9
-1	-10	-6	137.526	68.6304	28
1	-10	7	2809.04	238.806	4
-1	-10	-7	2043.31	200.801	28
1	-10	7	2847.73	257.437	8
1	-10	7	2971.94	258.422	10
-1	-10	-7	2766.33	239.539	9
-1	-10	-8	101.698	67.2173	15
1	-10	8	71.9684	67.9872	8
1	-10	8	69.8133	71.0601	10
1	-10	8	100.369	55.7837	4
-1	-10	-9	295.334	64.4643	15
1	-10	9	344.983	75.5988	8
1	-10	9	153.172	43.1062	4
1	-10	10	21.6570	24.5264	8
1	-11	-8	13.2892	42.7022	15
-1	-11	8	-7.10765	43.3340	8
1	-11	-7	1311.39	134.853	15
-1	-11	7	1391.54	147.883	8
-1	-11	7	973.405	125.999	3

1	-11	-6	594.536	100.266	15
-1	-11	6	532.755	108.381	3
1	-11	-6	529.819	96.5951	3
-1	-11	6	670.402	115.358	8
-1	-11	5	2700.22	240.484	3
-1	-11	5	2063.57	192.400	4
1	-11	-5	2437.04	222.308	3
1	-11	-5	2441.06	210.411	15
-1	-11	5	2361.13	233.716	8
1	-11	-5	2439.28	232.505	9
1	-11	-4	42.7884	73.9114	9
1	-11	-4	27.3518	76.1259	3
1	-11	-4	10.6440	61.1977	15
-1	-11	4	23.3564	56.9257	4
-1	-11	4	31.1288	82.1903	8
-1	-11	4	41.6596	64.6764	27
1	-11	-3	2400.37	220.042	15
-1	-11	3	2646.57	272.701	16
-1	-11	3	3036.57	256.609	4
-1	-11	3	3255.02	282.095	3
1	-11	-3	2990.57	269.500	3
-1	-11	3	3051.77	281.778	8
1	-11	-3	2965.64	265.553	9
-1	-11	3	2971.50	263.613	27
1	-11	-2	152.077	94.7041	16
-1	-11	2	242.072	110.290	16
-1	-11	2	206.250	97.3699	3
1	-11	-2	285.163	106.150	3
-1	-11	2	358.321	111.578	8

-1	-11	2	323.066	96.3941	4
1	-11	-2	196.403	104.818	8
1	-11	-2	244.089	89.4454	9
-1	-11	2	181.987	83.6917	27
1	-11	-1	-16.9096	82.4043	16
-1	-11	1	35.3669	91.6230	16
1	-11	-1	16.3353	79.6055	3
-1	-11	1	15.9124	77.5446	3
-1	-11	1	17.3649	72.7528	8
1	-11	-1	-36.3785	90.5464	8
1	-11	-1	51.9288	89.6860	4
-1	-11	1	29.4990	63.5868	4
1	-11	-1	-27.4188	65.3406	9
-1	-11	1	13.4623	59.6280	27
1	-11	0	166.634	88.4779	16
-1	-11	0	218.746	100.848	3
1	-11	0	148.707	102.554	3
-1	-11	0	279.127	99.1407	8
1	-11	0	219.587	111.955	8
1	-11	0	206.428	105.295	4
1	-11	0	227.255	82.6758	9
-1	-11	0	138.334	67.0551	27
-1	-11	0	252.130	93.8340	28
1	-11	1	80.5045	89.6759	16
-1	-11	-1	60.0764	76.3832	3
1	-11	1	72.7892	90.6506	8
1	-11	1	134.892	90.7260	4
-1	-11	-1	51.9194	82.5546	8
-1	-11	-1	45.6215	69.1899	28

-1	-11	-1	79.4993	55.4503	27
-1	-11	-2	17.2707	97.9138	16
1	-11	2	167.252	88.9773	16
1	-11	2	284.118	104.135	3
-1	-11	-2	70.0912	75.6375	3
1	-11	2	227.493	93.6166	4
1	-11	2	160.645	101.181	8
-1	-11	-2	137.484	88.0341	9
-1	-11	-2	130.180	78.0283	28
1	-11	3	15868.6	879.753	16
1	-11	3	17128.8	901.065	3
1	-11	3	15623.7	891.437	4
-1	-11	-3	14562.8	857.229	3
-1	-11	-3	15770.3	888.311	9
1	-11	3	17120.1	925.958	8
-1	-11	-3	13690.7	859.000	28
1	-11	4	999.427	163.878	8
-1	-11	-4	1197.19	142.020	3
1	-11	4	1372.36	168.981	3
1	-11	4	1251.18	163.047	4
1	-11	4	792.546	129.737	16
-1	-11	-4	1204.51	146.916	28
-1	-11	-5	713.032	128.396	9
1	-11	5	599.523	126.426	8
1	-11	5	484.238	100.887	3
-1	-11	-5	442.151	83.0030	3
-1	-11	-5	699.570	114.730	15
1	-11	5	900.999	130.800	4
-1	-11	-5	547.204	93.8529	28

-1	-11	-6	56.5444	63.6368	15
1	-11	6	80.6797	61.6059	4
1	-11	6	100.994	79.1694	8
-1	-11	-6	98.3057	69.4200	9
1	-11	7	4008.79	262.409	4
1	-11	7	4057.45	292.016	8
1	-11	8	6.99006	40.8038	8
-1	-12	6	154.020	54.2030	8
-1	-12	6	156.638	53.6550	3
-1	-12	5	-26.0616	45.0165	16
1	-12	-5	21.9884	44.6920	3
-1	-12	5	108.094	61.4052	3
-1	-12	5	9.22953	53.0572	8
-1	-12	4	181.353	75.6731	16
1	-12	-4	176.194	70.9424	9
1	-12	-4	226.664	73.0461	3
-1	-12	4	207.219	79.6857	8
-1	-12	3	72.7691	76.0020	16
1	-12	-3	51.1289	62.3844	9
-1	-12	3	69.9745	70.1444	3
1	-12	-3	11.0270	64.4061	3
-1	-12	3	48.5514	68.7071	8
-1	-12	2	223.436	87.9689	16
1	-12	-2	171.588	74.4229	9
1	-12	-2	206.152	83.0756	3
-1	-12	2	245.447	85.8941	3
-1	-12	2	77.7764	72.6209	8
-1	-12	1	1803.49	184.457	3
1	-12	-1	2222.50	206.587	3

-1	-12	1	2012.52	200.138	8
-1	-12	1	1951.75	201.044	16
1	-12	0	687.916	122.369	16
-1	-12	0	570.522	111.690	3
1	-12	0	582.802	120.891	3
-1	-12	0	472.953	101.872	9
1	-12	0	691.787	133.855	8
1	-12	1	753.543	133.395	8
-1	-12	-1	774.882	122.397	9
1	-12	1	683.494	118.603	16
1	-12	1	780.891	126.595	4
-1	-12	-1	856.801	125.395	3
-1	-12	-2	975.579	120.901	28
1	-12	2	846.638	140.957	8
-1	-12	-2	986.797	137.017	9
1	-12	2	797.347	122.301	16
1	-12	2	971.791	138.416	4
1	-12	2	1010.21	140.324	3
-1	-12	-2	747.102	115.508	3
1	-12	3	63.5570	75.3208	8
1	-12	3	10.7844	59.0199	4
1	-12	3	10.8293	62.2548	3
-1	-12	-3	34.1087	49.0237	3
-1	-12	-3-10.8075		65.8972	9
1	-12	4	851.850	126.073	8
1	-12	4	939.807	115.803	4
-1	-12	-4	729.020	86.4465	3
1	-12	4	717.367	107.272	3
-1	-12	-5	1353.18	124.467	15

1	-12	5	1634.93	154.566	8
1	-12	5	1301.95	126.445	4
1	-12	5	1419.82	130.686	3
-1	-12	-6	664.049	78.5579	15
1	-12	6	933.833	101.532	8
1	-12	7	371.493	49.6627	8
1	-13	-4	122.853	32.9377	3
1	-13	-3	22.9328	40.7946	3
-1	-13	3	52.4328	44.6890	3
-1	-13	3	6.03207	35.2278	16
-1	-13	2	1053.22	111.804	3
1	-13	-2	995.215	107.738	3
-1	-13	2	699.254	97.6933	16
1	-13	-1	1067.01	119.783	3
-1	-13	1	1216.75	124.827	3
-1	-13	0	250.195	61.9482	3
1	-13	0	262.397	72.3614	3
-1	-13	-1	1128.57	110.905	3
-1	-13	-2	1746.03	124.621	3
1	-13	2	2200.82	152.783	3
1	-13	3	13.6182	40.3533	8
1	-13	3	21.4610	34.0573	3
1	-13	4	393.887	56.6862	8
2	0	-15	-37.3982	38.0421	20
2	0	-14	788.508	142.873	21
2	0	-14	659.351	104.915	20
-2	0	14	356.469	82.3074	29
2	0	-13	12.9054	71.8713	20
-2	0	13	-88.4152	79.1582	29

-2	0	12	3386.03	284.813	29
2	0	-12	2907.09	270.195	20
2	0	-11	29.6919	76.9212	20
2	0	-11	-86.0935	360.698	17
2	0	-10	416.776	104.374	20
2	0	-10	321.919	156.054	24
2	0	-9	75.4617	202.875	17
2	0	-9	-36.9141	70.7350	20
2	0	-9	42.6482	86.6775	24
2	0	-8	12524.4	1110.10	17
2	0	-8	9536.57	805.932	24
2	0	-7	-55.5879	149.453	17
2	0	-7	-31.1376	70.7686	24
2	0	-6	79729.1	3452.90	17
2	0	-6	53115.9	3084.49	24
2	0	-5	70.1941	112.797	17
2	0	-5	38.5484	61.9709	24
2	0	-4	579210.	21630.8	17
2	0	-4	402171.	21396.9	24
-2	0	3	47.5108	80.4802	30
2	0	-3	32.5726	49.3696	24
2	0	-2	314727.	14917.9	25
2	0	-2	314482.	14896.4	24
2	0	-1	19.5831	44.5050	25
2	0	-1	19.6588	33.3060	2
2	0	0	109315.	5312.25	25
2	0	0	111151.	5271.42	2
2	0	1	-12.2751	52.9244	25
2	0	1	39.4721	44.6701	1

2 0 2 104170. 5160.58 1

2 0 2 118972. 5287.21 30

-2 0 -3 39.5977 69.7164 23

-2 0 -4 16843.3 1039.44 23

-2 0 -5-30.9438 104.295 23

-2 0 -6 36941.4 2119.03 23

-2 0 -7-44.7831 101.775 23

-2 0 -8 23009.7 1580.32 23

-2 0 -9 181.390 173.984 23

-2 0 -10 13432.2 732.818 20

-2 0 -10 9196.25 1022.76 23

-2 0 -11-38.8718 64.5065 20

2 0 11 15.7363 73.2804 29

-2 0 -12 842.508 122.872 20

2 0 12 872.318 138.555 29

-2 0 -13-17.9789 53.2633 20

2 0 13 33.0307 57.0477 29

-2 0 -14 2936.75 195.542 20

2 1 -15 755.377 84.8643 20

-2 -1 14 949.026 115.475 29

2 1 -14 1122.06 127.175 20

-2 -1 13 2970.17 254.904 29

-2 1 13 2944.12 259.432 29

2 1 -13 3203.36 262.794 20

-2 -1 12 4036.14 319.981 29

-2 1 12 3864.34 320.939 29

2 1 -12 3970.14 319.971 20

2 -1 -11 5877.65 831.652 17

2 1 -11 5757.62 777.680 17

2	1	-11	4040.43	552.103	24
-2	1	11	4483.64	599.024	30
-2	-1	11	4142.25	584.788	30
-2	1	11	5058.76	373.329	29
-2	-1	11	5054.14	369.939	29
2	1	-11	4089.38	346.983	20
-2	-1	10	2682.44	257.188	29
-2	1	10	2427.70	254.921	29
-2	1	10	2619.00	439.274	30
-2	-1	10	2374.20	420.732	30
2	1	-10	3922.95	656.974	17
2	1	-10	2553.45	421.073	24
2	1	-10	2519.50	242.083	20
-2	-1	9	5439.73	608.456	30
-2	1	9	5456.69	615.116	30
-2	-1	9	4978.51	402.300	29
-2	1	9	5026.12	397.908	29
2	-1	-9	5309.61	540.404	24
2	1	-9	6564.13	654.082	24
2	1	-9	5218.69	373.056	20
2	1	-8	1004.36	319.675	17
2	-1	-8	1349.42	356.916	17
-2	1	8	328.361	187.801	30
-2	-1	8	183.081	100.081	29
-2	-1	8	478.922	191.471	30
2	-1	-8	579.764	161.825	24
2	1	-7	17407.9	1271.20	17
2	-1	-7	18783.5	1303.17	17
-2	-1	7	14484.6	1035.51	30

-2	1	7	15362.5	1052.24	30
2	1	-7	14140.5	1040.81	24
2	-1	-7	14489.2	970.940	24
-2	-1	6	16417.9	1069.55	30
-2	1	6	16919.3	1086.43	30
2	1	-6	15882.3	1073.70	24
2	-1	-6	16499.0	1011.90	24
2	1	-5	30964.6	1755.56	24
2	-1	-5	30369.7	1668.64	24
-2	-1	5	31873.7	1743.14	30
-2	1	5	29580.4	1741.42	30
2	-1	-4	50536.3	2476.59	24
2	1	-4	49932.5	2555.93	24
-2	-1	4	50227.6	2551.15	30
-2	1	4	48990.6	2549.41	30
2	1	-3	129064.	6395.16	24
2	-1	-3	127268.	6310.30	24
-2	1	3	144358.	6442.03	30
-2	-1	3	118205.	6436.53	30
2	1	-2	10312.1	574.770	17
2	1	-2	9146.09	538.363	25
2	-1	-2	8783.25	484.672	24
2	1	-1	17191.3	942.769	25
2	1	-1	17764.2	955.072	17
2	-1	-1	18682.8	965.241	2
2	1	0	398763.	19191.2	25
2	-1	0	388859.	19185.7	25
2	-1	0	421451.	19193.0	17
2	1	0	434235.	19203.8	17

2	-1	0	392664.	19195.0	2
2	-1	0	399515.	19196.4	1
2	-1	1	20289.7	1095.91	1
2	1	1	20918.3	1117.66	25
2	-1	1	21509.2	1115.94	25
2	-1	2	15.3209	73.0412	25
2	1	2	34.5444	74.4820	30
-2	1	-2	34.6780	70.4960	23
2	1	2	50.8825	25.3887	1
2	-1	2	10.9656	41.7274	1
2	1	3	177069.	8461.09	30
2	-1	3	173044.	8454.17	30
-2	1	-3	173810.	8433.50	23
2	1	4	86611.6	4504.21	30
2	-1	4	93573.7	4513.82	30
-2	1	-4	88218.1	4465.41	23
2	1	5	11007.1	784.028	30
2	-1	5	11047.0	774.479	30
2	1	5	8886.06	521.723	29
2	-1	5	8675.28	516.237	29
-2	1	-5	10346.3	730.050	23
-2	-1	-5	10791.4	758.221	23
2	-1	6	18030.1	1177.29	30
2	1	6	15472.4	862.628	29
2	-1	6	15800.5	859.465	29
2	1	6	19157.6	1200.20	30
-2	1	-6	16340.4	1109.19	23
-2	-1	-6	17508.3	1147.05	23
2	-1	7	-48.4211	120.525	30

2	1	7	148.775	133.328	30
2	1	7	11.3006	55.0814	29
2	-1	7	-44.1190	56.0494	29
-2	-1	-7	-137.749	139.975	23
-2	1	-7	131.026	140.346	23
-2	1	-8	7931.22	772.473	23
-2	-1	-8	8415.67	808.039	23
2	1	8	9098.94	851.583	30
2	1	8	7905.41	506.415	29
2	-1	8	7722.02	502.968	29
2	-1	8	8620.97	833.434	30
-2	1	-9	2787.27	484.604	23
-2	-1	-9	4496.95	609.755	23
2	1	9	4321.13	609.806	30
2	-1	9	4335.99	336.117	29
2	-1	9	3901.86	579.377	30
2	1	9	4551.41	339.522	29
2	-1	10	2594.08	467.134	25
-2	-1	-10	2468.20	460.366	23
-2	1	-10	2334.79	216.917	20
2	1	10	2154.09	229.392	29
2	-1	10	2185.21	231.653	29
-2	1	-11	816.649	125.054	20
2	1	11	737.821	144.129	29
2	-1	11	633.338	128.281	29
-2	1	-12	6155.82	394.901	20
-2	-1	-12	5353.66	383.708	20
2	1	12	6186.66	417.769	29
2	-1	12	5810.19	406.050	29

-2	1	-13	34.5399	53.4358	20
-2	-1	-13	44.2388	59.6933	20
2	-1	13	94.9042	61.6085	29
-2	1	-14	871.767	86.4807	20
-2	-1	-14	757.868	85.7967	20
2	-2	-15	310.835	52.4885	20
2	2	-15	229.029	46.1804	20
2	-2	-14	442.869	86.3689	20
-2	2	14	324.329	76.6829	29
2	-2	-13	701.701	121.633	20
2	2	-13	913.758	130.523	20
-2	-2	13	597.038	104.253	29
-2	2	13	604.178	119.619	29
-2	2	12	1415.25	179.242	29
-2	-2	12	1412.00	172.354	29
2	2	-12	1440.32	176.880	20
2	-2	-12	1342.95	176.139	20
2	2	-11	5010.04	689.093	17
2	2	-11	4612.60	597.065	24
-2	2	11	5040.42	635.907	30
-2	2	11	5514.76	380.296	29
-2	-2	11	5809.41	377.244	29
-2	-2	11	3472.83	551.583	30
2	2	-11	4537.89	357.821	20
2	-2	-11	3952.89	344.248	20
2	2	-10	2196.62	452.758	17
-2	-2	10	1699.69	195.716	29
-2	2	10	1686.64	207.779	29
-2	2	10	1714.63	371.988	30

-2	-2	10	1553.71	339.656	30
2	2	-10	1610.49	344.137	24
2	2	-10	1428.29	178.333	20
2	-2	-10	1564.01	182.753	20
2	-2	-9	25596.8	1356.21	15
-2	2	9	27287.8	1703.12	30
-2	-2	9	25658.3	1661.99	30
-2	-2	9	25664.0	1369.00	29
-2	2	9	26298.9	1387.12	29
2	2	-9	24661.3	1349.12	20
2	-2	-9	25251.5	1352.59	20
2	2	-9	29200.1	1783.40	24
2	2	-8	29609.3	1425.07	24
-2	-2	8	8917.88	828.596	29
-2	2	8	29300.3	1348.84	30
-2	-2	8	29389.5	1314.57	30
-2	2	8	12835.7	901.054	29
2	2	-7	60103.3	3215.47	24
2	-2	-7	53549.8	2983.26	24
-2	-2	7	55422.7	3107.74	30
-2	2	7	61066.7	3167.23	30
-2	2	6	53234.2	2601.51	30
-2	-2	6	51011.2	2571.30	30
-2	-2	6	44203.5	2328.94	27
2	2	-6	48734.8	2619.62	24
2	-2	-6	45541.7	2415.23	24
2	-2	-5	89203.9	4733.68	24
2	2	-5	95933.5	4922.51	24
-2	-2	5	101323.	4873.35	30

-2	2	5	93624.6	4875.67	30
2	-2	-4	10975.2	620.654	24
-2	-2	4	11032.7	727.357	30
-2	2	4	11434.9	741.877	30
2	-2	-3	62346.4	2105.75	17
2	2	-3	45401.5	1967.13	17
-2	2	3	37898.1	1921.76	30
-2	-2	3	21836.0	1848.52	30
2	-2	-2	5673.72	413.140	1
2	2	-2	4887.28	366.892	17
2	2	-2	5456.02	364.906	25
-2	-2	1	2720.37	153.909	2
2	-2	-1	2454.92	224.463	17
2	2	-1	2928.56	229.237	17
2	-2	-1	2772.14	216.289	25
2	2	-1	2890.63	221.044	25
2	-2	0	104701.	5084.35	17
2	2	0	107554.	5095.27	25
2	-2	0	104826.	5081.49	25
2	-2	1	272855.	13166.5	25
2	2	1	270914.	13178.2	25
2	-2	1	264295.	13192.1	1
-2	-2	-1	312715.	13265.4	23
2	-2	2	518528.	24581.8	25
2	2	2	514324.	24598.0	25
2	-2	2	492558.	24582.8	1
-2	2	-2	489308.	24608.2	23
2	2	3	435638.	20842.2	30
-2	2	-3	439127.	20791.8	23

-2	-2	-3	437241.	20819.5	23
2	2	4	6770.78	562.137	30
2	-2	4	6293.28	546.866	30
-2	2	-4	7302.38	540.386	23
-2	-2	-4	6456.94	547.147	23
2	2	5	4608.78	478.194	30
2	-2	5	4277.21	454.122	30
2	2	5	4045.73	279.269	29
2	-2	5	4292.07	279.080	29
-2	-2	-5	5262.04	499.783	23
-2	2	-5	4028.70	431.752	23
2	2	6	50679.8	2614.81	30
2	2	6	44025.4	2231.86	29
2	-2	6	45119.7	2227.68	29
2	-2	6	46534.7	2560.85	30
-2	2	-6	42471.7	2477.79	23
-2	-2	-6	47433.7	2560.20	23
2	-2	7	5515.42	609.142	30
2	2	7	5850.32	384.534	29
2	-2	7	5980.90	379.953	29
2	-2	8	6509.95	397.241	29
2	2	8	5012.66	643.794	30
2	-2	8	4437.67	604.945	30
2	2	8	5806.49	392.329	29
2	-2	9	115.636	73.5023	29
2	2	9	75.0409	79.3021	29
-2	2	-9	70.9485	53.8225	20
-2	2	-9	-57.6188	117.137	23
2	2	10	5612.65	685.282	25

2	-2	10	5550.42	694.646	25
2	2	10	6784.15	407.734	29
2	-2	10	5941.14	378.755	29
-2	2	-10	5415.05	351.314	20
-2	-2	-10	5696.93	354.645	20
2	2	11	19265.1	1038.12	29
2	-2	11	17732.3	1017.58	29
-2	-2	-11	18169.0	1003.71	20
-2	2	-11	18748.6	1003.74	20
-2	2	-12	10.5444	54.6338	20
-2	-2	-12	10.8716	62.4911	20
2	-2	12	-38.3275	66.1954	29
2	2	12	40.0075	62.0985	29
-2	2	-13	77.1795	48.9764	20
-2	-2	-13	-24.6463	47.9557	20
2	2	13	-10.2431	57.9512	29
2	-2	13	-9.29283	49.0662	29
-2	2	-14	124.430	33.9030	20
-2	2	-14	124.096	42.0994	21
-2	-2	-14	221.185	45.6467	20
2	-3	-14	11.5263	36.5946	11
-2	3	14	73.6837	47.0782	29
2	3	-14	6.96380	38.7795	20
2	-3	-14	38.1676	44.5776	20
-2	3	13	4125.92	287.139	29
-2	-3	13	3039.68	251.228	29
2	-3	-13	5359.55	309.182	20
2	-3	-13	1977.57	268.448	21
2	3	-13	4613.03	290.939	20

2	3	-12	243.388	83.0796	20
2	-3	-12	445.324	101.973	20
-2	-3	12	177.374	80.4836	29
-2	3	12	145.101	90.6080	29
-2	3	11	-59.8743	149.091	30
-2	-3	11	224.925	151.236	30
2	-3	-11	-84.7261	192.558	17
2	3	-11	187.349	155.498	24
-2	3	11	185.995	83.6283	29
-2	-3	11	179.755	80.5432	29
2	-3	-11	69.8894	69.7180	20
2	3	-11	110.059	79.1987	20
2	3	-10	15662.1	1253.81	17
2	-3	-10	13604.2	799.565	15
2	-3	-10	18075.4	1451.79	17
2	3	-10	14366.3	1196.94	24
-2	3	10	12997.4	1106.76	30
-2	-3	10	9114.57	982.521	30
-2	3	10	15191.2	824.315	29
-2	-3	10	14543.2	799.587	29
2	-3	-10	13336.4	788.105	20
2	3	-10	13639.1	793.015	20
2	3	-9	73957.9	3481.30	17
2	-3	-9	59467.1	2853.16	15
2	3	-9	67164.6	3396.01	24
-2	3	9	51756.9	3161.59	30
-2	-3	9	44573.0	3072.77	30
-2	3	9	63706.9	2868.49	29
-2	-3	9	59128.6	2838.69	29

2	3	-9	53862.5	2828.13	20
2	-3	-9	54026.4	2829.80	20
2	3	-8	8075.80	795.211	17
2	3	-8	8219.67	755.405	24
-2	3	8	8809.73	702.484	30
-2	-3	8	6882.36	611.384	30
-2	3	8	6447.41	408.897	29
-2	-3	7	4702.23	432.864	30
-2	3	7	4733.61	458.404	30
2	-3	-7	3917.51	315.115	1
2	3	-7	5137.90	529.678	24
-2	-3	7	4828.17	261.672	27
2	-3	-6	700.806	264.929	17
2	3	-6	217.066	157.024	17
2	-3	-6	452.271	121.374	1
-2	3	6	248.148	145.581	30
-2	-3	6	697.361	175.673	30
-2	-3	6	357.090	76.0587	27
2	3	-5	2131.00	289.694	17
2	-3	-5	2734.72	454.473	17
2	-3	-5	1843.13	242.912	1
-2	-3	5	2085.51	171.389	27
-2	-3	5	2687.94	297.088	30
-2	3	5	2223.96	282.472	30
2	-3	-4	16273.4	977.198	17
2	3	-4	15272.7	864.763	17
2	-3	-4	14263.0	852.737	1
2	3	-4	13469.4	915.514	24
-2	-3	4	12844.1	818.939	30

-2	3	4	12093.6	852.944	30
2	3	-3	32807.0	1442.64	17
2	-3	-3	32640.4	1485.80	1
2	3	-3	33617.4	1546.43	24
2	-3	-2	320174.	10725.5	17
-2	-3	2	297534.	10627.8	2
2	-3	-2	334791.	10779.6	1
2	-3	-2	304701.	10842.2	2
2	3	-2	329210.	10806.1	24
2	3	-1	117320.	5318.38	25
-2	3	1	111741.	5252.52	24
2	3	-1	119719.	5440.25	24
2	-3	-1	120207.	5312.05	25
2	3	-1	96788.2	5315.87	17
2	-3	-1	96387.3	5321.55	17
2	-3	-1	116723.	5457.35	2
-2	-3	1	111738.	5282.82	2
2	-3	0	16528.3	879.548	25
2	3	0	17328.2	907.098	25
2	-3	0	15442.9	884.472	17
2	-3	0	18110.6	982.585	1
2	-3	0	16665.4	1002.29	2
2	-3	1	46473.1	2315.89	1
2	-3	1	42606.1	2346.04	2
2	-3	1	42623.6	2221.18	25
2	3	1	43722.3	2250.57	25
-2	-3	-1	48510.2	2282.42	23
2	-3	2	33258.7	1799.21	1
-2	-3	-2	35395.2	1781.98	23

-2	3	-2	35330.5	1735.73	23
2	3	2	32422.7	1758.61	25
2	-3	2	31283.8	1727.49	25
-2	3	-3	63614.6	3113.29	23
-2	-3	-3	65841.4	3176.29	23
2	-3	3	57257.8	3109.48	25
2	3	4	28704.4	1709.76	30
-2	3	-4	26690.5	1625.68	23
-2	-3	-4	34029.5	1738.69	23
2	-3	4	31603.5	1682.94	25
-2	3	-5	3269.87	392.166	23
-2	-3	-5	3925.40	447.422	23
2	-3	5	4081.83	266.884	29
2	3	5	4099.07	273.629	29
2	3	5	4577.54	478.305	30
-2	-3	-6	2018.53	341.919	23
2	3	6	1433.28	315.805	30
2	3	6	2093.45	181.388	29
2	-3	6	1949.14	168.088	29
2	3	7	837.221	126.237	29
2	-3	7	1092.93	134.206	29
2	3	7	901.154	261.983	30
-2	-3	-7	1119.68	283.210	23
-2	3	-7	669.009	212.670	23
2	3	8	181.532	185.859	30
2	-3	8	568.909	91.5976	7
2	-3	8	585.664	108.314	29
2	3	8	506.573	112.744	29
2	-3	8	526.279	201.382	25

-2	3	-8	196.627	124.450	23
-2	-3	-8	621.707	230.847	23
2	-3	8	502.488	91.3010	5
-2	3	-9	7374.61	403.939	20
2	-3	9	6042.19	373.818	5
2	-3	9	6645.58	376.823	7
2	-3	9	6602.10	415.166	29
2	3	9	7915.01	446.780	29
-2	-3	-9	8610.04	835.037	23
2	-3	9	7536.46	771.826	25
2	-3	10	3319.40	284.465	29
2	3	10	3490.59	297.320	29
-2	-3	-10	4070.24	607.567	23
-2	3	-10	3090.82	516.488	24
2	-3	10	3375.59	516.698	25
2	3	10	2859.71	491.111	25
-2	-3	-10	3293.68	271.670	20
-2	3	-10	3245.32	265.627	20
2	-3	11	27.1887	63.5058	29
-2	-3	-11	47.0418	65.6509	20
2	-3	12	981.741	132.759	29
2	3	12	1059.69	147.653	29
-2	3	-12	1424.79	141.611	20
-2	-3	-12	1479.58	148.284	20
2	3	13	44.4800	50.3319	29
-2	3	-13	6.00602	33.9828	20
-2	-3	-13	-7.03118	41.0453	20
2	-3	14	760.495	63.3437	10
-2	-3	-14	827.507	64.5870	20

2	-4	-14	180.904	50.4683	11
2	4	-14	180.192	52.7770	19
2	-4	-13	3130.24	223.349	20
2	4	-13	3488.04	223.073	20
-2	4	12	59.3538	68.3191	29
2	-4	-12	57.8444	57.6903	20
2	4	-12	99.0005	61.3161	20
-2	4	11	92.6241	78.4166	29
-2	-4	11	-33.1982	50.3135	29
2	4	-11	62.4465	64.7308	20
2	-4	-11	103.379	69.5010	20
-2	4	10	1650.82	192.739	29
-2	-4	10	1263.71	165.346	29
-2	4	10	841.648	269.417	30
-2	-4	10	698.344	230.507	30
2	-4	-10	1791.89	197.700	15
2	4	-10	1603.35	377.580	24
2	4	-10	1262.83	160.178	20
2	-4	-10	1651.97	175.402	20
2	-4	-9	246.115	79.7332	20
2	4	-9	309.833	85.3683	20
-2	4	9	150.865	198.168	30
-2	-4	9	44.8711	121.059	30
2	-4	-9	407.012	108.010	15
2	4	-9	320.384	206.147	24
-2	-4	9	209.495	90.2048	29
-2	4	9	279.447	101.695	29
2	-4	-8	20537.2	967.464	15
2	4	-8	20846.0	1396.53	24

2	-4	-8	18599.3	927.273	20
-2	4	8	18610.2	946.725	29
-2	4	8	16952.0	1209.75	30
-2	-4	8	14211.9	1110.45	30
-2	-4	8	12658.4	883.951	29
-2	-4	7	3861.08	242.427	27
-2	4	7	4051.77	425.798	30
-2	4	7	666.873	167.638	29
-2	-4	7	2806.01	337.987	30
2	4	-7	3697.13	422.734	17
2	4	-6	30.0417	86.3469	17
2	-4	-6	150.222	143.919	17
2	-4	-6	-40.5642	68.3604	1
-2	4	6	30.8719	76.8466	30
-2	-4	6	25.6631	82.4689	30
-2	-4	6	10.7111	47.4521	27
-2	-4	5	39011.3	2114.88	27
2	-4	-5	40728.9	2240.74	1
2	4	-5	43453.5	2263.92	17
2	4	-5	42341.4	2404.49	24
-2	4	5	45155.4	2306.49	30
-2	-4	5	47599.6	2265.80	30
-2	-4	4	637.319	91.1397	28
2	-4	-4	857.212	203.339	17
2	4	-4	326.040	126.110	17
2	-4	-4	439.581	131.002	1
-2	-4	4	635.398	123.028	30
-2	4	4	573.914	139.148	30
2	4	-4	578.591	174.851	24

2	-4	-3	116547.	4233.74	17
2	-4	-3	115604.	4240.23	1
-2	-4	3	97844.0	4153.54	2
2	4	-3	106082.	4304.71	24
-2	4	3	103452.	4136.64	30
2	4	-2	85321.7	4823.32	17
2	-4	-2	118833.	4924.49	17
-2	-4	2	117270.	4904.50	2
2	-4	-2	124872.	5112.21	2
2	-4	-2	128764.	5037.71	1
2	4	-2	122710.	5075.98	24
-2	4	2	76382.6	4825.08	30
2	4	-1	6308.67	341.796	25
-2	4	1	5233.34	314.128	24
2	4	-1	6348.84	482.948	24
2	-4	-1	4037.15	339.246	17
-2	-4	1	4923.44	355.486	2
2	-4	-1	6042.32	464.705	1
2	-4	-1	5679.71	495.935	2
-2	4	0	61474.3	2790.54	24
2	-4	0	57716.0	2721.02	25
2	4	0	58927.6	2759.49	25
2	-4	0	60287.8	2968.72	2
-2	-4	-1	18380.8	881.750	23
2	4	1	18212.5	874.180	25
2	-4	1	14788.3	798.834	25
2	-4	1	17905.5	995.485	1
2	-4	1	17126.4	1030.77	2
-2	4	-2	50840.4	2721.38	23

-2	-4	-2	58162.4	2798.46	23
2	-4	2	55329.0	2735.04	25
2	4	2	56100.7	2788.04	25
2	-4	2	57696.3	2967.98	2
2	-4	3	20805.4	1190.27	25
2	4	3	22338.9	1247.20	25
2	-4	3	21775.7	1392.72	2
2	-4	3	20415.2	1088.13	27
-2	-4	-3	22213.7	1253.74	23
-2	4	-3	21799.3	1174.73	23
2	4	4	40408.3	2018.11	25
2	-4	4	39090.1	1951.20	25
2	4	4	33631.1	1745.75	29
-2	4	-4	33870.4	1884.14	23
-2	-4	-4	37924.7	2000.83	23
2	4	5	455.954	78.5386	29
2	-4	5	244.315	125.694	25
-2	-4	-5	538.846	183.581	23
-2	4	-5	451.429	136.357	23
-2	-4	-6	4308.42	522.449	23
2	4	6	5169.39	310.959	29
2	-4	6	4666.29	287.739	29
2	-4	6	4395.05	494.110	25
2	-4	7	248.849	69.9151	29
2	4	7	172.957	74.6249	29
2	-4	7	153.870	64.6683	7
-2	-4	-7	150.733	153.338	23
2	-4	7	97.1688	57.4713	5
2	-4	7	88.7143	127.694	25

2	-4	8	19342.7	1065.12	5
2	-4	8	19092.9	1061.77	7
-2	-4	-8	19824.0	1466.37	23
2	-4	8	20920.5	1083.70	29
2	4	8	21778.9	1105.18	29
2	-4	8	19470.0	1433.19	25
2	4	8	20784.7	1490.20	25
2	4	9	300.539	194.655	25
-2	4	-9	549.419	232.714	24
-2	-4	-9	630.297	249.229	23
-2	-4	-9	556.749	105.562	20
-2	4	-9	500.318	94.9425	20
2	-4	9	504.557	101.368	7
2	4	9	515.401	118.406	29
2	-4	9	486.099	105.360	29
2	-4	9	561.677	103.048	5
2	4	10	11660.4	688.696	29
2	-4	10	10601.4	662.293	29
2	-4	10	13796.4	661.886	5
-2	-4	-10	14666.7	687.939	20
-2	4	-10	13895.2	671.872	20
-2	4	-11	7328.54	382.646	20
-2	-4	-11	8357.11	406.193	20
2	4	11	4569.11	367.995	29
2	-4	11	3768.32	340.213	29
2	4	12	116.231	64.4467	29
-2	4	-12	241.247	55.7755	20
2	-5	-14	84.9505	36.9273	11
2	5	-14	161.992	44.6656	19

-2	5	13	246.643	58.7287	29
-2	-5	12	410.954	85.7283	10
-2	5	12	327.291	78.6524	29
2	-5	-11	143.827	68.0499	20
2	-5	-11	193.694	82.4668	15
-2	5	11	174.327	73.3512	29
-2	-5	11	159.181	75.5856	10
2	-5	-10	24168.4	1726.18	17
2	-5	-10	21846.7	1181.19	15
2	5	-10	21888.5	1650.40	24
-2	5	10	22427.3	1155.11	29
2	-5	-10	20247.8	1136.17	20
2	-5	-9	7373.48	869.736	17
2	-5	-9	6798.45	445.514	15
-2	5	9	7367.42	426.130	29
2	-5	-9	6266.81	399.956	20
2	-5	-8	2041.73	208.954	15
2	-5	-8	3018.89	509.720	17
2	-5	-8	1965.58	208.387	3
-2	-5	8	2160.37	219.616	27
-2	5	8	1276.50	179.170	29
-2	5	8	799.064	243.379	30
2	5	-7	64068.9	3102.82	24
2	-5	-7	56041.2	2564.41	9
2	-5	-7	56763.5	2537.02	15
2	-5	-7	78596.3	3254.06	17
-2	-5	7	60957.6	2557.65	27
2	-5	-7	57864.9	2546.65	3
-2	5	7	35461.1	2463.22	29

-2	5	7	35907.3	2653.37	30
-2	-5	6	9516.96	383.228	27
2	-5	-6	10523.7	769.492	17
2	5	-6	10243.6	765.951	24
-2	-5	5	68.7178	104.765	2
2	-5	-5	23.7204	63.8123	1
-2	-5	5	33.4252	58.8734	27
2	-5	-5	37.7305	93.9397	17
-2	5	5	125.617	84.7003	30
2	5	-5	88.2097	134.469	24
2	-5	-4	35180.4	1783.71	1
-2	-5	4	30018.1	1768.57	2
-2	-5	4	29548.3	1601.13	28
2	-5	-4	36838.1	1819.71	17
-2	5	4	32578.9	1712.80	30
2	5	-4	30240.2	1875.59	24
2	-5	-3	574.498	146.853	1
-2	-5	3	484.557	124.377	2
-2	-5	3	392.344	83.7874	28
2	-5	-3	549.989	124.249	17
-2	5	3	385.495	97.5813	30
2	5	-3	446.289	153.504	24
-2	-5	2	6858.45	409.877	28
2	5	-2	8478.14	636.621	24
2	-5	-2	7552.63	638.715	2
2	-5	-2	9271.13	612.213	1
-2	-5	2	7506.89	508.387	2
2	5	-1	78157.5	3913.26	24
-2	-5	1	71842.9	3754.57	2

2	-5	-1	76612.9	3969.50	2
2	-5	-1	82984.4	3931.31	1
-2	5	0	85964.8	4325.53	24
-2	-5	0	87709.8	4247.50	23
2	-5	0	98265.5	4542.73	1
2	-5	0	90905.2	4563.39	2
-2	-5	-1	65207.2	3173.91	23
-2	5	-1	65429.1	3262.88	24
2	-5	1	60436.4	3417.54	2
2	5	2	4655.27	380.047	25
2	-5	2	4693.73	305.540	27
-2	5	-2	5188.36	452.659	24
-2	-5	-2	4547.65	382.137	23
2	-5	2	5368.70	551.587	2
2	5	3	321.936	110.435	25
2	-5	3	307.383	78.0454	27
-2	-5	-3	167.753	98.3469	23
-2	5	-3	259.361	124.007	24
2	-5	3	598.544	187.630	2
2	-5	4	3099.09	463.570	2
2	-5	4	2702.66	279.491	25
2	5	4	2164.63	303.557	25
-2	-5	-4	1975.89	295.958	23
2	5	5	13996.8	736.777	29
2	-5	5	16030.0	1211.04	2
2	5	5	15138.3	1009.04	25
-2	-5	-6	21170.6	1081.93	9
2	-5	6	19317.6	1057.09	5
2	-5	6	18919.3	1049.41	7

2	5	6	22012.1	1064.74	29
2	-5	6	20749.5	1556.46	2
2	5	6	19512.8	1346.93	25
-2	5	-6	23235.6	1504.34	24
-2	-5	-7	8175.96	512.782	9
2	-5	7	7456.57	486.984	5
2	-5	7	7846.41	488.651	7
2	5	7	8472.55	498.127	29
2	-5	7	8798.09	954.986	2
2	5	7	7398.02	747.238	25
-2	5	-7	9336.17	891.009	24
-2	5	-8	11333.2	1070.02	24
-2	-5	-8	10957.0	618.309	20
2	-5	8	11259.1	636.920	7
2	-5	8	11454.3	644.787	5
2	-5	8	13702.0	1240.55	2
2	5	8	11181.8	972.201	25
2	5	8	11438.6	649.936	29
2	5	9	610.267	117.201	29
-2	-5	-9	964.472	134.135	15
-2	5	-9	1492.42	376.068	24
-2	-5	-9	1152.61	131.630	20
2	-5	9	1329.20	368.808	2
2	-5	9	973.381	130.494	5
-2	-5	-10	3483.07	256.018	15
-2	-5	-10	3632.19	256.604	20
2	-5	10	3761.01	258.988	5
-2	-5	-11	928.617	105.343	20
2	5	11	209.907	73.9471	29

2	5	12	162.747	53.3779	29
2	-5	13	350.269	61.5429	10
2	-5	13	172.381	49.0921	4
-2	-6	13	43.9289	38.8434	8
-2	-6	12	237.633	64.3500	10
2	-6	-11	989.451	147.435	15
-2	-6	11	976.542	137.330	10
2	-6	-10	1385.40	179.988	15
-2	-6	10	1401.82	177.187	10
2	-6	-9	3911.33	333.056	15
-2	-6	9	4152.82	337.982	10
2	6	-9	3840.15	599.437	24
-2	-6	8	4297.35	348.722	27
2	6	-8	4069.12	596.176	24
2	-6	-8	4003.10	328.824	3
2	-6	-8	5079.06	608.134	17
2	-6	-8	4067.38	333.795	15
-2	-6	8	3626.61	327.124	10
2	6	-7	6588.96	756.298	24
-2	-6	7	5724.82	438.276	10
2	-6	-7	7512.40	482.215	9
-2	-6	7	6122.10	453.131	27
2	-6	-7	6114.12	443.146	3
2	-6	-7	6483.39	447.268	15
2	-6	-7	7329.14	714.636	17
2	-6	-6	19969.3	1126.12	3
-2	-6	6	19893.9	1125.82	10
-2	-6	6	21568.2	1140.04	27
-2	-6	6	20228.8	1109.26	5

2	-6	-6	24643.5	1454.22	17
2	6	-6	22589.8	1543.20	24
-2	-6	5	13175.1	762.438	27
2	-6	-5	13629.2	953.813	17
2	-6	-5	12872.9	884.690	1
2	6	-5	13458.6	1075.97	24
-2	-6	4	121.173	67.7733	27
-2	-6	4	201.929	75.0780	28
2	-6	-4	90.9074	97.6018	1
2	6	-4	137.795	132.181	24
2	-6	-3	3812.06	411.210	1
2	6	-3	3702.96	455.426	24
-2	-6	3	2983.82	254.177	28
2	-6	-2	20616.4	1384.40	2
2	6	-2	21328.6	1349.62	24
-2	-6	2	20586.6	1094.75	28
2	-6	-2	19814.0	1054.15	28
-2	-6	1	1396.68	149.927	28
2	-6	-1	1160.91	120.764	28
2	6	-1	1218.57	243.020	24
2	-6	-1	1101.78	264.770	2
2	-6	0	24503.6	1235.45	27
2	-6	0	23988.3	1241.34	28
2	-6	0	28143.6	1624.71	2
2	-6	1	62.0387	49.4496	28
2	-6	1	85.2276	46.7676	27
2	-6	1	181.055	130.137	2
-2	6	-1	46.7348	67.2500	24
2	-6	2	62973.7	3334.98	2

-2	6	-2	59958.7	3116.37	24
2	-6	2	58345.1	2895.30	27
2	-6	3	12198.4	700.894	27
-2	6	-3	12150.6	914.278	24
2	-6	3	13347.8	1051.26	2
2	-6	4	3621.99	277.961	27
2	-6	4	4783.65	601.431	2
-2	6	-4	4895.67	525.998	24
-2	6	-5	8211.34	771.846	24
-2	-6	-5	7174.03	438.535	22
2	-6	5	7450.82	444.157	5
2	-6	5	7153.10	435.091	7
2	-6	5	8428.05	853.291	2
2	-6	6	297.193	213.873	2
-2	-6	-6	276.658	90.7950	9
-2	6	-6	120.970	137.952	24
-2	-6	-6	130.293	108.763	23
2	-6	6	319.982	88.3564	7
2	-6	6	301.633	90.1946	5
2	-6	7	159.759	198.820	2
-2	-6	-7	45.0683	68.2689	9
-2	6	-7	-66.0724	164.455	24
-2	-6	-7	-49.8664	124.118	23
2	-6	7	-12.9624	70.9321	7
2	-6	7	-13.6530	69.3676	5
-2	6	-8	8882.03	950.096	24
-2	-6	-8	9586.04	604.509	9
2	-6	8	8570.25	1017.34	2
2	-6	8	10178.4	601.972	5

-2	-6	-9	87.8988	74.4518	15
2	-6	9	37.2854	64.4548	5
-2	-6	-10	4220.21	309.075	15
2	-6	10	4799.60	318.770	5
2	-6	11	12.0663	58.8040	4
2	-6	12	160.699	58.7538	10
2	-6	12	152.884	58.5927	4
2	-6	13	132.666	31.5893	8
2	-7	-13	384.802	62.0861	11
2	7	-13	499.418	68.4175	19
-2	-7	12	270.593	65.9688	8
-2	-7	12	290.007	57.0696	10
2	7	-12	248.720	76.5601	19
-2	-7	11	244.484	75.3999	10
2	-7	-11	148.780	74.1264	15
-2	-7	10	8283.69	545.508	10
2	-7	-10	9145.49	558.550	15
2	-7	-9	403.592	122.822	15
-2	-7	9	449.124	112.368	10
2	-7	-8	5377.52	406.921	3
-2	-7	8	6489.65	451.555	27
2	-7	-8	5532.55	416.072	15
2	7	-8	5411.17	714.026	24
-2	-7	8	5396.67	418.839	10
-2	-7	7	4162.44	361.979	27
2	-7	-7	4600.28	372.688	9
-2	-7	7	4574.70	366.339	10
2	7	-7	4461.63	631.071	24
2	-7	-7	4334.07	350.830	3

-2	-7	6	9068.43	589.144	27
-2	-7	6	8404.60	579.785	10
2	7	-6	9886.61	946.752	24
2	-7	-6	9184.71	586.435	3
2	-7	-5	1453.45	188.090	9
-2	-7	5	1271.63	175.113	10
-2	-7	5	1498.61	185.796	27
2	-7	-5	1518.52	189.825	3
2	7	-5	1297.77	312.840	24
-2	-7	4	149.113	71.2560	10
2	-7	-4	202.818	87.2419	9
2	-7	-4	95.6948	65.0311	8
2	-7	-4	117.147	78.9048	3
-2	-7	4	73.2372	59.7586	8
-2	-7	4	30.0787	65.0414	28
2	-7	-4	222.237	91.3920	16
2	7	-4	107.409	109.263	24
2	-7	-3	2012.47	185.195	28
-2	-7	3	2489.20	229.666	28
-2	-7	3	1938.75	214.315	10
2	-7	-3	2645.48	240.422	8
2	7	-3	2453.16	396.639	24
2	-7	-3	2094.70	218.470	3
2	7	-2	33003.6	1976.83	24
2	-7	-2	31569.2	1601.33	28
2	-7	-1	35465.2	1800.31	28
2	-7	-1	39254.2	2243.57	2
2	-7	0	1241.31	139.517	28
2	-7	0	1169.66	128.831	27

2	-7	0	1403.39	313.231	2
2	-7	1	392.065	84.9294	28
2	-7	1	512.753	93.4346	27
2	-7	2	32760.9	1783.40	8
2	-7	2	40361.6	2275.20	2
2	-7	2	36529.4	1760.11	27
2	-7	2	33523.4	1749.42	5
-2	-7	-2	33644.0	1781.10	16
2	-7	2	34900.2	1786.85	4
-2	-7	-3	1522.92	162.357	22
2	-7	3	1401.61	183.008	10
2	-7	3	1728.09	367.793	2
2	-7	3	1706.12	185.427	27
2	-7	3	1384.06	158.249	7
-2	-7	-3	1437.17	184.631	16
2	-7	3	1947.23	206.928	4
2	-7	4	1698.72	205.943	10
2	-7	4	1203.04	333.871	2
-2	-7	-4	1735.66	204.092	16
-2	7	-4	1914.96	338.001	24
-2	-7	-4	1516.65	170.144	22
2	-7	4	1614.55	183.073	5
2	-7	4	1271.69	162.900	7
2	-7	4	1750.90	206.670	4
-2	-7	-5	3262.52	290.328	9
-2	7	-5	3681.90	511.157	24
2	-7	5	4555.57	642.668	2
-2	-7	-5	3250.34	275.696	22
2	-7	5	3339.65	310.342	4

2	-7	5	3155.55	277.534	5
-2	7	-6	11693.2	1052.10	24
2	-7	6	11764.0	1140.15	2
-2	-7	-6	12382.3	718.760	9
2	-7	6	11850.2	693.631	7
2	-7	6	11184.0	691.456	5
2	-7	6	11022.1	729.292	4
-2	7	-7	64.8478	114.236	24
-2	-7	-7	203.218	92.7495	9
2	-7	7	79.0219	113.662	2
2	-7	7	94.3295	87.9851	4
2	-7	7	73.3407	76.1568	5
2	-7	8	-27.7948	70.6214	5
2	-7	8	-72.6240	102.728	4
-2	-7	-8	14.3873	70.1257	9
2	-7	9	3060.33	286.346	4
-2	-7	-9	2726.53	243.720	15
2	-7	9	2671.77	242.335	5
2	-7	9	3425.82	294.443	10
-2	-7	-10	55.2737	56.1973	15
2	-7	10	39.3025	76.4959	4
2	-7	10	-9.00182	47.5672	5
2	-7	10	166.286	84.5056	10
-2	-7	-11	46.5405	37.5881	15
2	-7	11	-20.3330	57.5588	10
2	-7	11	136.974	60.8844	4
2	-7	12	11.4538	37.2616	10
2	-7	12	-12.3544	24.7564	4
2	-7	12	31.0501	38.3835	8

2	8	-12	285.544	56.3041	19
2	-8	-12	250.894	56.7234	11
2	8	-11	1005.12	120.739	19
-2	-8	11	853.205	111.202	8
2	8	-10	23.7550	59.1285	19
2	-8	-10	-60.6635	77.9735	15
-2	-8	10	23.7154	56.5172	10
-2	-8	10	-24.8690	65.6559	8
2	-8	-9	2796.82	255.750	15
-2	-8	9	2486.99	244.285	10
-2	-8	9	2401.62	245.892	8
2	-8	-8	4009.53	330.192	3
-2	-8	8	4043.47	348.010	8
-2	-8	8	4542.34	357.258	10
2	-8	-7	585.379	131.328	15
2	8	-7	490.019	237.954	24
2	-8	-7	733.632	140.040	3
-2	-8	7	830.319	151.254	10
-2	-8	7	654.782	146.071	8
-2	-8	7	595.757	134.638	27
2	-8	-6	46.6497	75.7802	15
2	8	-6	67.9330	119.563	24
2	-8	-6	72.0042	101.844	9
-2	-8	6	17.8961	77.1539	10
2	-8	-6	16.6580	79.3958	3
-2	-8	6	-53.6312	94.3922	27
-2	-8	6	17.7227	80.5390	8
2	-8	-5	224.356	100.863	9
-2	-8	5	70.6607	71.9731	10

2	8	-5	89.8869	61.4035	19
-2	-8	5	86.5497	82.6507	8
2	8	-5	64.2060	113.112	24
2	-8	-5	150.929	88.6847	3
-2	-8	4	9715.77	633.428	28
2	-8	-4	9996.75	639.716	16
-2	-8	4	9472.08	628.340	10
2	-8	-4	9728.96	638.564	8
2	-8	-4	9927.47	626.516	9
-2	-8	4	9802.03	628.005	8
2	-8	-4	9468.47	623.821	3
2	8	-4	10002.1	940.180	24
2	8	-3	3164.48	483.495	24
2	-8	-3	3872.55	316.704	3
-2	-8	3	3774.93	313.469	27
2	-8	-3	3268.32	263.621	28
2	-8	-3	3653.72	318.374	16
-2	-8	3	3388.44	302.372	8
2	-8	-3	3521.83	300.454	9
2	-8	-3	3235.17	310.448	8
2	-8	-2	1741.32	204.171	4
2	-8	-2	1310.19	181.116	3
2	-8	-2	1307.01	151.419	28
2	-8	-2	1562.66	192.858	16
2	-8	-2	1393.81	177.916	9
-2	-8	2	1321.88	179.925	8
2	-8	-2	1531.81	198.740	8
2	-8	-1	779.959	116.390	28
2	-8	-1	653.915	130.441	8

-2	-8	1	701.970	134.665	8
2	-8	-1	773.304	136.327	16
2	-8	-1	726.181	141.042	4
-2	-8	1	555.914	108.072	4
2	-8	0	14714.6	825.819	3
-2	-8	0	14204.3	804.426	4
2	-8	0	13017.7	816.812	4
2	-8	0	14224.3	1167.45	2
-2	-8	0	14959.7	828.745	28
2	-8	0	13842.7	764.355	27
2	-8	0	13591.9	778.496	28
2	-8	0	13128.0	779.722	5
2	-8	0	13389.2	807.677	16
2	-8	0	14010.9	830.532	8
-2	-8	0	14274.2	820.854	8
2	-8	1	-16.6173	77.3846	4
-2	-8	-1	44.4330	67.3126	4
2	-8	1	13.2229	55.4080	5
2	-8	1	-62.1300	126.268	2
2	-8	1	45.6274	67.3718	3
-2	-8	-1	-13.4591	59.6215	3
2	-8	1	35.4366	52.3282	27
-2	-8	-1	64.7453	71.6995	8
2	-8	1	103.310	78.2514	8
-2	-8	-1	50.0356	77.6698	16
2	-8	1	-15.3225	92.1164	10
-2	-8	-2	252.365	98.5101	8
2	-8	2	193.526	94.4537	10
-2	-8	-2	164.759	66.4793	22

-2	-8	-2	132.913	65.2290	3
2	-8	2	91.0758	86.2238	3
2	-8	2	65.4940	163.197	2
-2	-8	-2	136.561	85.3248	16
2	-8	2	249.817	107.547	8
2	-8	2	383.064	95.2220	27
2	-8	2	51.1789	67.5792	4
2	-8	2	195.108	76.6490	5
-2	-8	-3	18563.7	975.212	22
2	-8	3	18740.9	1031.45	10
2	-8	3	20127.1	1514.81	2
-2	-8	-3	17989.3	1032.64	16
2	-8	3	17308.8	997.458	5
2	-8	3	18729.0	1005.35	27
2	-8	3	17201.2	1026.24	4
-2	-8	-3	17012.1	978.901	3
2	-8	4	19548.4	1102.28	10
-2	-8	-4	18693.9	1040.26	22
2	-8	4	20244.3	1110.50	4
2	-8	4	17605.2	1523.74	2
-2	-8	-4	19722.6	1107.72	16
2	-8	5	561.277	139.312	4
2	-8	5	676.820	133.240	5
-2	-8	-5	415.980	119.731	9
2	-8	5	503.090	140.047	10
2	-8	5	448.757	253.276	2
2	-8	6	6211.24	775.662	2
-2	-8	-6	5311.28	401.521	9
2	-8	6	5245.46	397.481	5

2	-8	6	5213.29	417.937	4
-2	-8	-7	4594.79	352.086	9
2	-8	7	4996.43	360.835	5
2	-8	7	5281.62	392.006	4
2	-8	8	1856.52	223.304	10
2	-8	8	2056.55	210.079	5
2	-8	8	2020.69	228.321	4
2	-8	9	7623.28	473.583	10
2	-8	9	6372.52	462.574	8
-2	-8	-9	6490.95	420.855	15
2	-8	9	6414.84	422.187	5
2	-8	9	6938.27	454.785	4
2	-8	10	176.651	75.6040	10
-2	-8	-10	148.083	55.7570	15
2	-8	10	137.608	65.1298	4
2	-8	10	162.966	76.0816	8
2	-8	11	96.7128	41.2788	4
2	-8	11	133.092	58.0106	8
2	-8	11	169.308	54.4394	10
-2	-9	11	98.1748	30.3355	8
2	9	-11	82.2280	36.8578	19
2	-9	-11	128.495	42.4033	11
-2	-9	10	180.110	61.6978	8
2	9	-10	274.699	67.3936	19
2	-9	-10	335.510	83.5270	15
2	-9	-10	347.252	75.2748	11
2	-9	-9	619.198	107.044	11
-2	-9	9	768.207	122.712	8
2	9	-9	870.859	116.528	19

2	-9	-8	3731.20	289.348	3
-2	-9	8	3450.63	299.441	10
-2	-9	8	3447.47	295.851	8
2	9	-8	3412.91	271.291	19
-2	-9	7	179.233	89.3126	8
-2	-9	7	67.2315	83.8313	10
2	9	-7	159.881	75.1619	19
2	-9	-7	117.277	90.7589	15
-2	-9	7	171.015	99.9607	27
2	-9	-7	14.6127	72.9283	3
2	9	-6	2536.27	230.549	19
2	-9	-6	2665.54	257.040	15
2	-9	-6	2494.05	258.353	3
-2	-9	6	2562.06	265.346	8
-2	-9	6	2512.05	266.837	10
2	-9	-6	2947.45	282.258	9
2	-9	-5	12809.2	788.482	3
2	-9	-5	12818.7	768.958	15
2	9	-5	12058.1	1121.76	24
2	-9	-5	13588.9	801.395	9
-2	-9	5	13534.2	805.129	8
2	-9	-5	13044.2	742.618	11
-2	-9	4	2203.64	246.560	8
2	-9	-4	2334.44	249.060	9
-2	-9	4	2561.07	257.502	27
2	-9	-4	2298.01	257.001	4
2	-9	-4	2278.79	244.474	3
2	-9	-4	1972.42	220.566	15
2	-9	-3	147.903	80.4288	9

-2	-9	3	194.189	84.4661	8
2	-9	-3	249.268	103.366	8
2	-9	-3	88.0987	96.7019	3
2	-9	-3	115.246	91.8466	4
2	-9	-3	165.052	93.4709	16
-2	-9	3	155.432	89.7900	27
2	-9	-2	3484.46	302.876	9
2	-9	-2	3554.67	328.990	8
-2	-9	2	3718.24	321.222	8
2	-9	-2	3213.16	269.277	28
-2	-9	2	3792.38	319.365	27
-2	-9	2	3355.99	293.323	4
2	-9	-2	4035.95	337.742	3
2	-9	-2	3439.46	328.680	4
2	-9	-2	3350.08	311.623	16
2	-9	-1	-14.5917	59.3087	9
-2	-9	1	105.321	79.7679	8
2	-9	-1	37.8723	90.2513	8
2	-9	-1	12.7059	53.2335	28
-2	-9	1	-17.7219	86.3624	28
-2	-9	1	-15.0887	66.8312	4
2	-9	-1	37.2724	78.0787	4
2	-9	-1	-17.2966	74.5675	16
2	-9	0	469.678	107.500	9
-2	-9	0	724.877	143.856	8
2	-9	0	778.348	157.395	8
-2	-9	0	931.632	148.295	3
2	-9	0	796.243	146.419	3
-2	-9	0	811.988	145.865	28

-2	-9	0	874.290	145.643	4
2	-9	0	926.360	164.932	4
2	-9	0	1061.61	160.202	16
-2	-9	-1	4467.50	370.378	8
2	-9	1	4593.36	383.407	8
2	-9	1	4191.64	319.967	27
-2	-9	-1	4711.75	376.400	28
2	-9	1	3845.29	584.511	2
-2	-9	-1	4598.01	347.529	3
2	-9	1	4555.33	367.126	3
2	-9	1	3924.14	360.518	4
-2	-9	-1	3882.64	362.909	16
2	-9	1	4644.02	365.249	16
2	-9	2	4716.64	384.430	10
-2	-9	-2	4985.48	394.309	8
2	-9	2	4765.16	370.384	5
2	-9	2	4931.34	359.200	27
-2	-9	-2	4924.00	390.090	28
2	-9	2	4163.94	618.683	2
-2	-9	-2	4646.32	362.597	3
2	-9	2	4485.26	376.963	3
2	-9	2	5035.72	401.450	4
-2	-9	-2	4896.25	397.027	16
2	-9	3	8901.10	588.033	10
2	-9	3	8922.97	568.126	5
2	-9	3	9966.05	977.656	2
-2	-9	-3	8065.33	537.696	3
2	-9	3	8932.04	577.038	3
2	-9	3	8741.65	591.339	4

-2	-9	-3	7960.81	512.768	22
-2	-9	-3	8819.54	591.990	16
-2	-9	-4	56.0037	84.8325	16
2	-9	4	46.6643	80.5939	3
2	-9	4	215.793	163.437	2
-2	-9	-4	-37.9953	64.3476	3
2	-9	4	-18.8063	74.0119	4
2	-9	4	-18.5495	77.7157	10
-2	-9	-4	51.1125	83.0274	9
2	-9	5	14023.2	834.377	5
2	-9	5	14294.1	861.465	4
2	-9	5	13550.4	812.451	3
2	-9	5	14682.2	866.091	10
-2	-9	-5	14859.6	848.110	9
2	-9	6	4423.17	342.200	5
2	-9	6	3568.90	295.125	3
2	-9	6	4508.73	358.291	4
-2	-9	-6	4012.50	332.542	9
2	-9	6	4053.82	350.217	10
2	-9	7	4566.07	347.461	5
-2	-9	-7	4460.09	342.489	9
2	-9	7	4769.30	365.522	4
-2	-9	-8	21.5500	61.9378	15
2	-9	8	-39.1371	70.1466	4
2	-9	8	-14.7886	78.0849	8
-2	-9	-9	884.592	113.133	15
2	-9	9	1052.16	127.450	4
2	-9	9	906.343	132.842	10
2	-9	9	1141.76	141.853	8

-2	-9	-10	1155.14	103.499	15
2	-9	10	1218.05	109.471	4
2	-9	10	1395.55	131.644	8
2	-9	10	1306.71	128.746	10
-2	-10	9	317.410	66.8497	8
2	-10	-8	63.2069	63.4483	15
-2	-10	8	107.669	64.8428	8
2	-10	-7	324.454	95.7740	15
-2	-10	7	317.315	92.3544	8
2	-10	-7	318.646	96.3674	3
2	-10	-6	295.612	103.149	3
2	-10	-6	515.921	111.506	15
-2	-10	6	570.983	122.867	8
2	-10	-6	325.958	113.348	9
-2	-10	6	492.360	115.900	27
2	-10	-5	13408.5	777.832	15
2	-10	-5	13162.9	805.570	9
-2	-10	5	13215.9	807.923	8
-2	-10	5	13839.8	818.682	27
2	-10	-5	14553.8	813.866	3
2	-10	-4	6931.56	448.496	15
2	-10	-4	6242.91	471.143	3
2	-10	-4	6962.21	478.096	9
-2	-10	4	6340.51	473.225	8
-2	-10	4	7150.53	486.705	27
2	-10	-3	1439.63	164.868	15
2	-10	-3	1312.31	192.415	3
2	-10	-3	1283.84	203.225	4
-2	-10	3	1434.54	195.643	8

2	-10	-3	1261.91	199.068	8
2	-10	-3	1266.03	178.859	9
-2	-10	3	1565.32	197.028	27
2	-10	-2	12442.8	776.605	16
-2	-10	2	12009.8	740.483	4
2	-10	-2	12701.9	792.403	4
2	-10	-2	13269.3	786.571	3
-2	-10	2	12351.0	773.277	8
2	-10	-2	12952.7	759.214	9
2	-10	-2	13189.1	800.614	8
-2	-10	2	12710.9	770.819	27
-2	-10	2	12880.0	785.057	28
-2	-10	1	710.500	152.138	16
2	-10	-1	631.142	139.989	16
-2	-10	1	906.096	159.262	3
-2	-10	1	932.044	164.424	8
2	-10	-1	1228.59	190.735	8
-2	-10	1	854.308	141.944	4
2	-10	-1	961.309	175.589	4
2	-10	-1	887.253	139.195	9
-2	-10	1	1064.42	162.688	27
-2	-10	1	1272.58	187.795	28
2	-10	0	699.193	138.143	16
-2	-10	0	536.608	137.728	16
-2	-10	0	768.962	136.090	3
-2	-10	0	595.048	134.995	8
2	-10	0	610.605	145.145	4
2	-10	0	387.921	100.444	9
-2	-10	0	737.287	145.374	28

-2	-10	-1	646.098	151.079	16
2	-10	1	1182.06	169.018	16
-2	-10	-1	797.759	141.568	3
2	-10	1	1015.85	167.864	3
2	-10	1	738.316	161.230	4
-2	-10	-1	740.803	146.493	8
-2	-10	-1	887.042	158.020	28
2	-10	2	321.311	117.470	3
-2	-10	-2	475.820	110.986	3
2	-10	2	635.397	134.837	4
-2	-10	-2	402.047	126.376	28
-2	-10	-2	636.781	146.689	16
2	-10	2	498.871	118.585	16
2	-10	3	990.684	160.009	3
-2	-10	-3	902.298	142.257	3
2	-10	3	1094.89	178.810	4
-2	-10	-3	1289.16	182.486	28
2	-10	3	866.232	143.865	16
-2	-10	-3	1401.44	193.110	16
2	-10	4	64.0458	67.6332	16
2	-10	4	43.5298	72.2317	3
-2	-10	-4	56.0071	59.1447	3
2	-10	4	17.4402	81.2112	4
2	-10	4	-17.2189	89.2165	10
-2	-10	-4	15.9465	82.6244	9
-2	-10	-4	-80.5022	74.9728	28
2	-10	5	9342.50	600.056	4
2	-10	5	8939.03	561.417	3
2	-10	5	9674.50	604.861	10

-2	-10	-5	9404.04	585.719	9
-2	-10	-5	8939.10	580.296	28
2	-10	6	14.3494	77.2011	4
-2	-10	-6	140.513	71.1445	28
2	-10	6	14.9280	75.8908	10
-2	-10	-6	51.9263	68.5944	9
2	-10	7	54.1800	72.8308	8
-2	-10	-7	30.7243	48.8134	28
-2	-10	-7	30.4787	57.4802	15
-2	-10	-7	-31.7536	65.4231	9
2	-10	7	-11.9188	67.4327	4
2	-10	8	551.549	102.750	8
2	-10	8	380.210	82.6369	4
-2	-10	-8	461.073	83.5261	15
2	-10	9	316.844	63.6053	8
2	-10	9	309.505	51.4342	4
-2	-10	-9	292.513	55.0864	15
-2	-11	8	405.216	62.7364	8
-2	-11	8	509.241	76.2478	3
2	-11	-7	128.727	57.3104	15
-2	-11	7	37.6106	54.1361	3
-2	-11	7	51.8167	53.4686	8
2	-11	-7	54.3662	50.0087	3
2	-11	-6	18.2698	53.3287	15
2	-11	-6	10.4613	58.2286	3
-2	-11	6	22.0083	57.0213	8
2	-11	-5	48.0603	54.3809	15
-2	-11	5	-53.8266	62.6608	27
2	-11	-5	25.0872	69.8124	3

-2	-11	5	-25.6208	73.6356	8
2	-11	-4	622.042	125.993	3
-2	-11	4	697.530	138.634	3
-2	-11	4	594.100	119.323	8
2	-11	-4	724.172	126.717	9
-2	-11	4	772.355	131.455	27
2	-11	-3	2106.29	213.025	9
-2	-11	3	2019.52	222.110	8
-2	-11	3	2215.56	221.777	27
2	-11	-3	1979.43	225.017	3
-2	-11	3	2140.70	223.802	3
2	-11	-2	2966.79	289.955	8
2	-11	-2	3173.70	265.796	9
-2	-11	2	3194.30	284.367	8
-2	-11	2	2883.57	267.440	27
-2	-11	2	2759.18	269.200	3
2	-11	-2	3331.61	304.407	4
2	-11	-2	3009.88	282.957	3
2	-11	-1	3330.22	305.736	16
-2	-11	1	3983.21	333.555	16
2	-11	-1	3104.84	268.176	9
-2	-11	1	3626.74	314.057	8
-2	-11	1	4126.93	312.106	27
-2	-11	1	3357.85	307.315	28
2	-11	-1	4055.88	337.009	4
-2	-11	1	3754.71	307.902	3
-2	-11	0	1509.61	202.461	16
2	-11	0	1615.64	190.573	16
2	-11	0	2028.60	226.912	4

-2	-11	0	1524.44	187.449	3
-2	-11	0	1171.81	177.704	8
-2	-11	0	1471.54	177.422	27
-2	-11	0	1709.86	202.415	28
2	-11	1	814.774	147.911	3
-2	-11	-1	581.067	121.880	3
2	-11	1	781.284	148.124	4
-2	-11	-1	642.789	111.582	27
-2	-11	-1	685.764	134.707	28
2	-11	1	684.723	124.931	16
-2	-11	-1	869.005	155.480	16
-2	-11	-2	-12.1823	65.6451	3
2	-11	2	162.941	99.9357	3
-2	-11	-2	87.1425	60.7788	27
-2	-11	-2	104.032	74.1080	28
-2	-11	-2	147.686	100.139	16
2	-11	2	165.321	76.5704	16
2	-11	2	114.417	99.7473	4
-2	-11	-3	4591.94	345.602	9
-2	-11	-3	3946.29	329.034	28
-2	-11	-3	4311.32	310.138	3
2	-11	3	4879.89	363.542	4
2	-11	3	4670.67	343.376	3
2	-11	4	265.336	84.3250	3
2	-11	4	407.223	104.052	4
-2	-11	-4	308.682	86.4928	28
-2	-11	-4	377.739	100.250	9
2	-11	5	171.514	78.8977	4
-2	-11	-5	312.281	80.2891	28

-2	-11	-5	423.656	97.5789	9
2	-11	6	367.730	87.2152	4
-2	-11	-6	440.396	89.7752	9
-2	-11	-6	288.483	64.1004	28
2	-11	7	131.440	57.9807	8
2	-11	7	111.701	50.4790	4
2	-11	8	997.288	89.8550	8
-2	-11	-8	650.686	72.0650	15
-2	-12	7	365.837	53.1416	3
-2	-12	6	-14.0669	41.6756	16
-2	-12	6	25.3060	30.8604	8
-2	-12	5	134.358	63.9681	3
2	-12	-5	112.901	57.0604	3
-2	-12	5	113.483	58.3210	8
-2	-12	5	130.262	65.7519	16
-2	-12	4	3470.02	266.843	16
-2	-12	4	3608.33	263.456	3
2	-12	-4	3440.07	259.308	3
-2	-12	4	3238.25	248.898	8
-2	-12	3	-59.6980	50.5818	27
2	-12	-3	-22.3390	69.0600	3
-2	-12	3	-22.1046	66.4647	3
-2	-12	3	-21.0816	62.4793	8
-2	-12	2	1613.12	173.097	8
-2	-12	2	1731.55	167.050	27
-2	-12	2	1620.04	177.889	3
2	-12	-2	1755.61	184.832	3
-2	-12	1	374.566	107.203	16
2	-12	-1	633.169	119.939	3

-2	-12	1	497.248	101.859	3
-2	-12	0	293.678	95.1052	16
-2	-12	0	200.034	85.0104	9
2	-12	0	411.164	104.547	4
-2	-12	0	375.596	89.7843	3
-2	-12	-1	77.5865	63.8661	3
2	-12	1	11.8227	65.7999	3
-2	-12	-1	-11.2097	63.4199	9
-2	-12	-1	12.9669	73.3609	16
-2	-12	-1	-45.6723	58.0113	28
2	-12	1	12.7988	65.0264	4
-2	-12	-2	184.266	77.2555	9
2	-12	2	143.737	72.1746	4
-2	-12	-2	89.4004	55.0013	3
2	-12	2	32.3641	63.1648	3
-2	-12	-2	114.804	62.9335	28
2	-12	3	946.305	129.788	4
2	-12	3	1039.91	123.914	3
-2	-12	-3	806.944	91.0163	3
-2	-12	-3	930.122	113.273	28
-2	-12	-3	896.129	105.548	15
-2	-12	-4	1662.73	158.334	9
2	-12	4	1734.81	159.413	4
-2	-12	-4	1469.23	135.161	15
-2	-12	-5	112.701	53.5383	9
-2	-12	-5	124.492	47.2320	15
2	-12	5	218.599	55.1008	4
-2	-12	-6	117.181	35.0333	15
-2	-13	4	723.895	73.7607	3

2	-13	-4	636.077	64.6166	3
-2	-13	3	467.698	77.3015	16
-2	-13	3	763.356	83.9989	3
2	-13	-3	669.997	81.2598	3
-2	-13	2	358.631	65.8698	3
2	-13	-2	368.983	70.1453	3
2	-13	-1	166.773	57.4558	3
-2	-13	1	123.339	47.7726	3
-2	-13	0	87.1430	40.4454	9
-2	-13	0	224.126	51.0300	3
-2	-13	-1	11.4622	35.9029	9
-2	-13	-1	-23.8718	27.7215	3
2	-13	1	-6.53821	41.4933	3
2	-13	2	1180.16	95.0202	3
-2	-13	-2	526.462	74.3773	9
3	0	-15	6.50889	39.1294	20
3	0	-14	10.3561	57.6475	20
-3	0	14	-48.8112	55.2410	29
-3	0	13	1371.57	164.892	29
3	0	-13	1305.48	171.589	20
-3	0	12	-14.6575	101.021	29
3	0	-12	-30.1896	88.1178	20
-3	0	11	17425.7	1330.29	30
3	0	-11	15990.1	967.020	20
3	0	-10	157.636	196.197	17
3	0	-10	43.8409	72.7969	20
3	0	-9	121.350	108.865	24
3	0	-9	73.3321	197.213	17
3	0	-9	105.977	68.8229	20

3	0	-8	144.371	103.942	24
3	0	-8	130.475	162.510	17
3	0	-7	1622.04	257.594	24
3	0	-7	2556.27	419.108	17
3	0	-6	-48.2562	69.3673	24
3	0	-6	131.946	189.617	17
3	0	-5	105349.	5682.34	24
3	0	-5	143141.	5935.59	17
-3	0	4	23.2062	91.5294	30
3	0	-4	95.0283	66.6334	24
3	0	-3	45273.5	2334.46	24
3	0	-3	45953.7	2363.77	25
3	0	-3	49111.3	2423.11	17
3	0	-2	15.0725	59.3213	25
3	0	-1	174266.	8362.25	2
3	0	-1	177725.	8433.38	25
3	0	0	-20.3721	46.3007	2
3	0	0	-32.8803	76.5609	25
3	0	1	196814.	9467.72	1
3	0	1	200333.	9537.44	25
3	0	2	44.0976	46.2226	1
3	0	2	21.5050	75.7181	25
3	0	3	28993.4	1500.49	1
3	0	3	26552.6	1638.46	25
-3	0	-4	-29.9039	91.1696	23
-3	0	-5	40985.4	2307.69	23
-3	0	-6	129.845	107.901	23
-3	0	-7	1354.39	308.209	23
-3	0	-8	-113.019	151.931	23

3	0	8	82.3850	56.3772	18
-3	0	-9	36100.2	2313.57	23
3	0	10	-67.6118	153.624	25
-3	0	-10	64.8901	131.874	23
-3	0	-10	12.7462	59.3546	20
-3	0	-11	6135.88	418.876	20
3	0	11	6694.76	457.466	29
-3	0	-12	-40.5230	60.9236	20
3	0	12	-26.3364	61.3398	29
-3	0	-13	364.197	70.1777	20
3	0	13	327.264	81.1642	29
-3	0	-14	3.49838	22.7625	20
3	1	-15	387.944	67.6915	20
3	1	-14	129.843	68.1031	20
-3	-1	14	-9.25912	49.8142	29
3	1	-13	1638.09	216.316	21
3	1	-13	2406.65	205.786	20
-3	1	13	2157.45	196.110	29
-3	-1	13	2125.65	192.209	29
-3	1	12	762.563	146.397	29
-3	-1	12	686.724	137.762	29
3	1	-12	787.385	140.302	20
3	1	-11	6040.61	791.825	17
3	-1	-11	6623.25	842.371	17
-3	1	11	6074.15	424.683	29
-3	-1	11	5827.90	418.577	29
-3	1	11	5228.43	654.191	30
-3	-1	11	4484.33	613.292	30
3	1	-11	4756.70	387.355	20

3	-1	-10	6460.59	806.165	17
3	1	-10	6553.29	788.785	17
-3	1	10	4797.64	594.047	30
-3	-1	10	4014.83	566.102	30
-3	1	10	4352.94	385.693	29
-3	-1	10	3635.45	373.232	29
3	1	-10	4740.14	564.720	24
3	1	-10	4594.10	348.949	20
3	1	-9	206.352	221.304	17
3	-1	-9	226.708	217.477	17
3	1	-9	282.572	166.150	24
3	1	-9	250.447	82.2334	20
3	-1	-9	292.481	86.1641	20
-3	1	9	97.9488	149.709	30
-3	-1	9	143.884	176.070	30
3	1	-8	83.8829	95.7655	24
3	-1	-8	377.233	125.456	24
-3	-1	8	214.028	137.872	30
-3	1	8	87.4525	117.935	30
3	1	-8	488.288	232.252	17
3	-1	-8	340.281	195.826	17
3	1	-7	14425.3	1105.27	17
3	-1	-7	15405.3	1126.73	17
-3	1	7	13085.0	936.731	30
-3	-1	7	12673.7	915.060	30
3	1	-7	11838.0	887.321	24
3	-1	-7	10030.0	767.716	24
3	1	-6	36227.5	2141.19	17
-3	-1	6	37291.3	1994.98	30

-3	1	6	35067.6	1987.74	30
3	-1	-6	31990.7	1858.70	24
3	1	-6	34651.6	1970.29	24
-3	1	5	146507.	7114.99	30
-3	-1	5	149333.	7125.33	30
3	-1	-5	134916.	6988.20	24
3	1	-5	139493.	7092.07	24
3	1	-4	210590.	9189.42	17
-3	1	4	193755.	9176.20	30
-3	-1	4	212090.	9181.44	30
3	-1	-4	177515.	9003.64	24
3	1	-4	174217.	9091.01	24
3	1	-3	145734.	7276.50	24
3	1	-3	149015.	7265.09	25
3	1	-3	158658.	7322.69	17
3	1	-2	70938.4	3621.08	25
3	1	-2	72203.9	3638.84	24
3	-1	-2	72641.0	3632.12	2
3	1	-2	79291.5	3665.17	17
3	1	-1	586567.	28066.2	25
3	-1	-1	578564.	28053.2	2
3	1	-1	616714.	28081.5	17
3	-1	0	1966.82	220.050	25
3	1	0	1975.75	220.389	25
3	1	0	1774.02	137.836	2
3	-1	0	1805.36	202.480	2
3	-1	0	2013.77	222.000	1
3	1	0	2265.05	234.015	17
3	-1	0	1991.14	219.154	17

3	-1	1	75252.4	3764.94	25
3	1	1	78168.3	3778.16	25
3	-1	1	74356.6	3726.30	1
3	-1	2	69405.6	3298.57	25
3	1	2	70153.6	3306.91	25
3	-1	2	65788.7	3205.97	1
3	1	2	61117.5	3126.87	1
3	-1	3	148453.	7581.38	1
3	-1	3	156879.	7710.92	25
3	1	3	150747.	7715.57	25
3	1	4	12156.9	637.702	29
3	-1	4	10580.5	625.975	29
3	1	4	11878.5	863.230	25
3	-1	4	12085.4	849.653	25
-3	1	-4	13370.5	849.322	23
3	1	5	25087.2	1286.27	29
3	-1	5	23440.4	1275.09	29
3	-1	5	24850.3	1559.25	25
-3	1	-5	28285.0	1568.39	23
-3	-1	-5	28613.5	1588.59	23
3	-1	6	12645.9	733.379	29
3	1	6	13609.4	743.420	29
-3	-1	-6	14567.7	1048.43	23
-3	1	-6	15141.0	1059.13	23
-3	1	-7	342.642	179.347	23
-3	-1	-7	51.2467	127.571	23
3	1	7	106.201	70.1245	29
3	-1	7	-12.9933	60.5408	29
3	-1	7	53.0686	142.687	25

-3	1	-8	2602.46	446.381	23
-3	-1	-8	3052.48	491.093	23
3	1	8	2976.82	238.522	18
3	1	8	3177.81	507.146	25
3	-1	8	3254.78	511.902	25
3	-1	8	3065.20	273.668	29
3	1	8	3839.85	302.380	29
3	1	9	54967.2	3123.99	25
3	-1	9	51969.5	3114.89	25
-3	-1	-9	60508.1	3152.45	23
3	1	9	55795.4	2610.55	29
3	-1	9	54388.8	2603.21	29
-3	-1	-10	-65.7537	133.630	23
-3	1	-10	62.8863	127.803	23
3	-1	10	-66.3280	150.707	25
3	1	10	67.5849	137.351	25
3	1	10	83.1006	97.0172	29
3	-1	10	16.4503	83.5805	29
-3	1	-10	37.6976	66.3502	20
3	1	11	279.856	97.7756	29
3	-1	11	107.619	83.1212	29
-3	1	-11	153.652	76.4549	20
-3	-1	-11	239.179	89.7202	20
3	-1	12	789.297	130.373	29
3	1	12	938.342	138.919	29
-3	-1	-12	1000.45	129.686	20
-3	1	-12	1010.19	126.649	20
3	-1	13	78.9331	62.2233	29
-3	1	-13	227.095	58.5960	20

-3	-1	-13	179.005	59.6097	20
-3	-1	-14	16.9470	21.2457	20
3	2	-15	81.9541	36.1731	20
3	-2	-15	69.1450	37.2233	20
3	2	-14	2438.26	183.543	20
3	-2	-14	2247.46	181.139	20
-3	-2	13	55.4007	61.6807	29
-3	2	13	11.9668	68.7985	29
3	2	-13	24.5227	68.2535	20
3	-2	-13	12.4519	66.9645	20
3	2	-12	1269.07	167.258	20
3	-2	-12	1252.94	169.334	20
-3	2	12	1288.76	175.877	29
-3	-2	12	1459.97	173.800	29
3	2	-11	14732.8	1270.40	17
3	2	-11	12820.8	821.923	20
3	-2	-11	13299.9	825.352	20
3	2	-11	13801.1	1156.21	24
-3	2	11	17404.7	875.929	29
-3	2	11	12773.9	1139.07	30
-3	-2	11	12496.0	1129.88	30
-3	-2	11	16681.4	854.246	29
-3	2	10	95.3532	106.409	29
-3	2	10	378.169	205.667	30
-3	-2	10	155.162	149.016	30
3	2	-10	407.913	218.707	17
3	2	-10	273.088	88.2523	20
3	-2	-10	304.414	93.7451	20
-3	-2	10	162.959	99.2651	29

3	2	-10	168.462	128.018	24
3	-2	-9	20155.9	991.473	15
3	2	-9	28054.9	1618.98	17
3	-2	-9	19156.2	982.372	20
3	2	-9	19458.5	981.643	20
-3	-2	9	19199.0	1300.25	30
-3	-2	9	10397.8	929.243	29
-3	2	9	18658.1	1002.92	29
-3	2	9	21511.5	1359.61	30
3	2	-9	21881.4	1392.26	24
3	2	-8	10683.7	938.688	17
-3	2	8	9547.69	830.597	30
-3	-2	8	9746.53	819.602	30
3	2	-8	9782.47	850.579	24
3	-2	-7	1281.31	328.221	17
-3	-2	7	1096.50	243.334	30
-3	2	7	1036.94	230.034	30
3	2	-7	893.407	231.822	24
-3	-2	7	1007.98	121.938	27
3	-2	-6	150.357	52.9094	24
3	2	-6	102.113	109.542	24
3	-2	-6	138.836	133.147	17
3	2	-6	149.707	126.279	17
-3	-2	6	29.2269	94.1112	30
-3	2	6	215.044	149.830	30
3	2	-5	35531.5	1677.45	17
3	-2	-5	38518.9	1788.14	17
3	-2	-5	24202.9	1376.33	24
3	2	-5	28899.8	1611.60	24

-3	-2	5	26693.2	1553.88	30
-3	2	5	29810.8	1584.11	30
3	2	-4	159263.	6760.98	17
3	2	-4	141596.	6738.41	24
-3	2	4	139959.	6716.36	30
3	2	-3	2544.15	288.528	24
3	2	-3	2949.43	295.659	17
3	-2	-3	3075.20	316.268	17
3	-2	-2	150425.	7411.31	2
3	-2	-2	151064.	7345.50	25
3	2	-2	155178.	7353.29	25
3	-2	-2	153692.	7400.10	17
3	2	-2	152033.	7384.95	17
3	-2	-1	2981.65	289.744	1
3	-2	-1	2877.12	293.406	2
3	-2	-1	2738.53	254.202	25
3	2	-1	2655.93	253.676	25
3	-2	-1	2449.92	254.045	17
3	2	-1	2934.49	276.373	17
3	-2	0	193612.	9678.87	2
3	-2	0	205794.	9673.41	1
3	2	0	204009.	9655.63	25
3	-2	0	196984.	9633.57	25
3	-2	0	205269.	9665.96	17
3	-2	1	5661.76	482.418	2
3	-2	1	5661.89	443.388	1
3	-2	1	6162.36	452.291	25
3	2	1	6516.99	468.425	25
3	-2	2	3037.24	304.050	25

3	2	2	3407.25	327.051	25
3	2	2	2394.62	187.657	1
3	-2	2	2470.58	264.437	1
-3	-2	-2	3240.13	353.493	23
-3	2	-2	3393.70	352.715	23
3	-2	3	111897.	5675.73	25
3	2	3	110616.	5691.73	25
-3	-2	-3	127874.	5727.09	23
-3	2	-3	124464.	5695.36	23
3	-2	3	104606.	5605.47	1
-3	-2	-4	7480.60	594.826	23
-3	2	-4	6580.10	553.086	23
3	-2	4	7128.38	587.070	25
3	2	4	5877.21	559.847	25
3	-2	4	5770.76	374.858	29
3	2	4	6602.22	386.535	29
-3	2	-5	1547.26	289.215	23
-3	-2	-5	1809.40	309.507	23
3	-2	5	2006.38	327.037	25
3	2	5	2187.69	349.679	25
3	2	5	1652.60	171.869	29
3	-2	5	1848.93	172.165	29
-3	-2	-6	2780.57	423.019	23
-3	2	-6	2289.31	383.663	23
3	2	6	2384.99	424.189	25
3	-2	6	2875.81	438.525	25
3	2	6	3262.23	256.252	29
3	-2	6	3304.74	251.408	29
3	2	7	-54.7669	136.315	25

3	-2	7	158.627	161.186	25
3	2	7	-27.0952	56.7591	29
3	-2	7	51.8134	65.8114	29
3	2	8	272.994	100.290	29
3	-2	8	130.986	78.7836	29
-3	2	-8	161.865	173.612	23
3	-2	8	238.150	160.321	25
3	2	8	246.123	177.077	25
3	-2	9	8119.20	466.674	5
3	2	9	6946.72	817.137	25
3	-2	9	7981.07	845.202	25
3	2	9	8643.38	529.443	29
3	-2	9	8981.08	529.041	29
3	2	10	508.353	122.695	29
3	-2	10	365.108	103.662	29
-3	-2	-10	472.415	100.362	20
-3	2	-10	292.110	86.7595	20
3	-2	11	101.468	72.3100	29
3	2	11	105.546	81.2072	29
-3	2	-11	100.531	69.1997	20
-3	-2	-11	92.1942	70.4169	20
-3	-2	-12	3941.42	264.377	20
-3	2	-12	4099.15	262.315	20
3	2	12	3748.40	282.326	29
-3	-2	-13	13.0412	39.7584	20
-3	2	-13	-29.2330	40.7324	20
3	2	13	-8.49277	43.1522	29
-3	3	14	8.07150	43.4191	29
3	3	-14	21.7455	40.3633	20

3	-3	-14	31.1972	47.5687	20
3	-3	-14	46.5599	38.3486	11
3	3	-13	-54.0047	73.6504	20
3	-3	-13	22.2984	59.9805	20
-3	3	13	-21.9052	65.8716	29
-3	-3	13	-84.4507	60.3358	29
-3	3	12	1971.78	205.723	29
-3	-3	12	1893.54	190.135	29
3	3	-12	1936.41	200.786	20
3	-3	-12	2017.26	206.390	20
3	-3	-11	28.9034	67.3079	20
3	3	-11	42.7967	69.5321	20
3	-3	-11	-79.6538	140.196	17
-3	3	11	57.6634	131.026	30
-3	-3	11	215.811	155.068	30
3	3	-11	121.607	138.160	24
-3	-3	11	13.0863	71.6216	29
-3	3	11	-14.9351	87.1901	29
3	3	-10	154.529	77.0546	20
3	-3	-10	198.832	79.2217	20
-3	3	10	-53.7668	122.281	30
-3	-3	10	150.579	144.354	30
3	-3	-10	80.1970	215.669	17
3	3	-10	247.492	177.905	17
3	3	-10	185.252	153.789	24
-3	-3	10	81.7067	85.4645	29
-3	3	10	61.6783	90.1637	29
3	3	-9	11873.4	964.275	17
3	-3	-9	11564.9	1087.01	17

3	-3	-9	9842.10	570.939	15
3	3	-9	9784.76	906.215	24
3	3	-9	9904.38	558.071	20
3	-3	-9	10638.6	567.537	20
-3	3	9	8477.17	805.940	30
-3	-3	9	8605.36	782.547	30
-3	3	9	9327.35	574.136	29
3	3	-8	50.0579	113.826	17
3	-3	-8	139.141	199.999	17
3	3	-8	211.904	161.553	24
-3	3	8	88.2765	126.934	30
-3	-3	8	80.9615	116.429	30
3	-3	-8	91.9485	73.2431	15
-3	-3	7	24425.9	1330.57	27
3	-3	-7	24435.9	1515.34	1
3	3	-7	29535.9	1692.89	17
3	-3	-7	31997.2	1875.30	17
3	3	-7	25392.6	1659.29	24
-3	-3	7	27547.5	1613.60	30
-3	3	7	27188.5	1639.25	30
-3	-3	6	6159.84	396.903	27
3	-3	-6	6937.79	572.162	1
3	3	-6	7512.53	624.462	17
3	3	-6	6519.81	620.265	24
-3	-3	6	6103.39	555.454	30
-3	3	6	6737.93	587.350	30
-3	-3	5	11667.9	652.021	27
3	-3	-5	15468.4	1002.22	17
3	3	-5	13862.9	863.502	17

-3	3	5	12105.3	821.482	30
-3	-3	5	9983.51	788.328	30
3	3	-5	12069.8	882.467	24
3	-3	-5	13208.7	862.195	1
3	3	-4	43693.4	2298.68	17
3	-3	-4	48225.8	2389.36	17
3	-3	-4	47197.0	2351.68	1
3	-3	-3	58353.7	2259.26	17
3	-3	-3	55804.8	2267.67	1
3	3	-2	87214.7	4298.75	25
3	3	-2	87432.7	4404.74	24
3	-3	-2	90728.5	4398.10	1
3	-3	-2	92844.3	4366.39	17
3	3	-2	82319.2	4332.52	17
3	-3	-1	3929.14	320.898	25
3	3	-1	4542.01	340.417	25
3	3	-1	4241.40	357.758	17
3	-3	-1	3814.54	335.820	17
3	-3	0	26302.2	1469.69	25
3	3	0	27607.5	1497.36	25
3	-3	0	27775.2	1555.51	1
3	-3	0	30434.4	1508.27	17
3	-3	1	84716.3	4248.99	1
-3	-3	-1	81897.2	4270.49	23
3	3	1	88306.2	4235.63	25
3	-3	1	84674.3	4197.80	25
3	-3	2	797.855	166.609	1
-3	-3	-2	851.782	181.685	23
3	-3	2	555.772	127.151	25

3	3	2	686.663	161.459	25
-3	-3	-3	74362.5	3553.36	23
-3	3	-3	69304.3	3488.71	23
3	-3	3	67359.2	3502.26	25
3	3	3	64011.7	3524.54	25
-3	3	-4	39372.1	2103.03	23
-3	-3	-4	42768.0	2184.12	23
3	3	4	37637.7	1892.41	29
3	-3	4	36154.1	1882.81	29
3	3	4	40307.3	2176.50	25
3	-3	4	42767.4	2159.44	25
-3	-3	-5	17638.8	1154.29	23
3	-3	5	15820.9	855.467	29
3	3	5	16386.1	866.034	29
3	3	5	17840.0	1167.91	25
3	-3	5	16135.0	1118.78	25
-3	-3	-6	11555.9	925.926	23
3	3	6	12239.1	947.791	25
3	-3	6	10892.0	892.658	25
3	-3	6	10995.4	610.649	29
3	3	6	10903.4	617.682	29
3	-3	7	986.479	130.971	5
-3	-3	-7	743.990	264.254	23
-3	3	-7	457.337	187.504	23
3	-3	7	1542.77	147.671	7
3	3	7	1114.31	298.836	25
3	-3	7	832.591	264.321	25
3	-3	7	1277.52	159.019	29
3	3	7	1356.39	165.796	29

3	-3	8	2881.83	240.099	5
-3	-3	-8	3919.44	539.033	23
3	-3	8	3200.01	237.002	7
3	-3	8	3575.52	514.492	25
3	3	8	2650.03	457.839	25
3	3	8	3735.78	291.404	29
3	-3	8	3138.06	274.270	29
-3	3	-9	3031.85	232.848	20
3	-3	9	2862.77	225.476	5
-3	-3	-9	4017.49	561.489	23
3	-3	9	2681.37	212.049	7
3	3	9	3317.44	279.632	29
3	-3	9	2723.59	252.257	29
3	3	9	3435.56	518.584	25
3	-3	9	3877.60	541.694	25
-3	-3	-10	462.343	99.0971	20
-3	3	-10	658.559	108.236	20
3	-3	10	639.612	96.7346	5
3	3	10	283.568	106.282	29
3	-3	10	384.537	105.586	29
3	3	11	1360.52	163.850	29
3	-3	11	1189.24	147.216	29
-3	-3	-11	2013.58	169.849	20
-3	-3	-11	1134.69	165.391	21
-3	3	-11	1898.46	160.565	20
3	3	12	1189.82	150.541	29
-3	3	-12	1616.13	141.045	20
-3	-3	-12	1432.38	140.376	20
-3	3	-13	44.6071	28.4105	20

-3	-3	-13	94.9024	40.5485	20
3	-4	-14	68.8692	42.1733	11
3	4	-14	66.5408	42.8814	19
-3	4	13	56.4153	53.2129	29
3	-4	-13	54.4536	58.3407	20
3	4	-13	-8.37068	51.0523	20
-3	4	12	327.741	88.3790	29
3	4	-12	327.388	83.7531	20
3	-4	-12	200.592	76.9552	20
-3	4	11	526.160	118.419	29
-3	-4	11	557.211	100.526	29
3	4	-11	477.841	103.209	20
3	-4	-11	465.040	103.438	20
3	-4	-11	257.667	129.141	21
3	-4	-10	1259.10	156.593	20
3	4	-10	254.658	125.965	21
3	4	-10	1233.72	154.875	20
-3	4	10	1037.50	170.961	29
-3	-4	10	965.072	144.860	29
-3	4	10	858.356	253.779	30
3	-4	-10	824.059	334.811	17
3	4	-10	2061.13	402.187	24
3	-4	-10	1456.70	181.398	15
3	-4	-9	14933.9	1219.41	17
3	-4	-9	12610.5	665.998	15
3	4	-9	13052.1	1070.40	24
-3	-4	9	7664.23	579.592	29
-3	4	9	8680.64	848.038	30
-3	-4	9	7367.15	769.530	30

-3	4	9	10112.4	630.024	29
3	4	-9	10942.7	615.824	20
3	-4	-9	11957.9	632.501	20
-3	-4	8	5230.39	306.583	27
3	-4	-8	4609.25	301.357	15
3	-4	-8	5795.31	699.585	17
3	4	-8	6354.39	656.610	24
-3	4	8	4201.09	475.909	30
-3	-4	8	4249.57	454.066	30
-3	-4	8	768.700	163.680	29
-3	4	8	1943.97	229.055	29
3	-4	-8	4531.04	271.611	20
-3	-4	7	1126.25	148.842	27
3	-4	-7	1158.50	213.475	1
3	-4	-7	1198.46	317.887	17
3	4	-7	1349.02	266.496	17
3	4	-7	2042.66	361.924	24
-3	4	7	1431.11	270.258	30
-3	-4	7	1308.42	250.596	30
3	4	-6	48439.5	2543.15	17
3	-4	-6	45007.3	2492.33	1
-3	4	6	47084.9	2556.12	30
-3	-4	6	50440.5	2521.35	30
-3	-4	6	43291.3	2300.25	27
3	4	-5	50863.9	2638.99	17
3	-4	-5	52089.4	2676.17	1
-3	4	5	49834.8	2653.83	30
-3	-4	5	51368.6	2622.33	30
-3	-4	5	46443.2	2451.65	27

-3	-4	4	8.68480	40.4508	28
-3	-4	4	17.8523	54.4281	2
3	-4	-4	114.046	86.9187	1
3	-4	-4	59.9236	86.1163	17
3	4	-4	-43.6635	70.1574	17
-3	-4	4	19.2384	61.8250	30
-3	4	4	180.187	97.1041	30
3	4	-4	70.7820	101.719	24
-3	-4	3	53651.0	2520.25	28
3	4	-3	43089.3	2647.04	17
3	-4	-3	65806.7	2754.33	17
3	-4	-3	66298.3	2821.65	1
3	4	-3	59849.2	2824.98	24
-3	4	3	37855.7	2596.81	30
3	4	-2	271.673	119.180	24
3	4	-2	82.6489	90.9568	17
3	-4	-2	364.387	116.756	17
3	-4	-2	220.996	116.131	1
3	-4	-2	163.005	124.319	2
3	-4	-1	102833.	5114.62	25
3	4	-1	107706.	5142.38	25
3	-4	-1	106747.	5167.00	17
3	-4	-1	107093.	5342.28	2
3	-4	0	56447.7	2880.15	1
3	-4	0	56045.2	2935.36	2
3	-4	0	51997.0	2707.72	25
3	4	0	55993.1	2755.12	25
-3	-4	-1	54421.0	1882.70	23
3	-4	1	54199.0	2004.43	1

3	-4	1	52194.0	2072.31	2
3	4	1	53148.0	1865.51	25
3	-4	1	50551.5	1783.48	25
-3	4	-2	13217.8	926.475	23
-3	-4	-2	17274.7	993.140	23
3	-4	2	16078.2	1117.01	2
3	-4	2	18420.7	1088.77	1
3	-4	2	14603.9	921.809	25
3	4	2	15600.6	970.666	25
3	4	3	1716.14	152.145	29
-3	-4	-3	1758.26	270.639	23
-3	4	-3	1672.19	236.684	23
3	-4	3	2162.92	366.923	2
3	4	3	2028.32	281.096	25
3	-4	3	1552.60	237.912	25
3	4	4	3648.07	247.264	29
3	-4	4	2608.37	219.688	29
-3	-4	-4	3289.30	398.949	23
-3	4	-4	3378.76	354.918	23
3	-4	4	3558.94	508.772	2
3	-4	4	3576.14	390.947	25
3	4	4	4100.30	439.210	25
-3	-4	-5	5827.88	622.711	23
3	4	5	7055.77	409.385	29
3	-4	5	6086.38	383.785	29
3	-4	5	5937.43	589.926	25
3	4	5	7475.99	674.310	25
3	-4	6	9171.36	544.226	5
-3	-4	-6	7471.73	788.494	23

-3	-4	-6	10026.7	569.768	22
3	-4	6	9122.56	536.365	7
3	-4	6	8658.85	793.924	25
3	4	6	9918.22	857.786	25
3	4	6	11056.9	575.219	29
3	-4	6	10115.0	552.355	29
3	-4	7	109.666	60.1742	7
3	-4	7	188.021	71.0554	29
3	4	7	196.132	76.7889	29
-3	-4	-7	-53.6344	121.946	23
3	4	7	-55.6618	113.206	25
3	-4	7	148.701	142.527	25
3	-4	7	83.1630	62.8856	5
-3	4	-8	4344.26	307.220	20
3	-4	8	4394.22	322.831	5
3	-4	8	4393.32	318.664	7
-3	-4	-8	3490.74	540.188	23
3	4	8	5432.13	644.046	25
3	-4	8	3926.21	541.573	25
3	4	8	4447.71	346.376	29
3	-4	8	4503.56	335.565	29
-3	4	-9	14512.4	760.529	20
-3	-4	-9	14038.5	766.156	20
3	-4	9	14523.1	773.760	5
3	-4	9	14707.0	766.718	7
-3	-4	-9	11189.8	1112.08	23
-3	4	-9	16500.2	1293.82	24
3	4	9	12860.3	792.040	29
3	-4	9	11113.0	760.014	29

3	4	9	14336.6	1181.72	25
-3	4	-10	4993.77	308.513	20
-3	-4	-10	5241.49	321.208	20
3	4	10	3443.95	310.234	29
3	-4	10	2841.64	283.070	29
3	-4	10	4700.32	303.098	5
-3	-4	-11	989.290	119.858	20
-3	4	-11	1058.27	111.591	20
-3	-4	-12	1485.04	116.109	20
3	4	12	268.185	76.5178	29
3	-4	13	1524.47	125.650	4
3	-5	-14	77.2952	33.9860	11
3	5	-14	140.067	43.6157	19
3	-5	-13	100.769	52.7057	11
-3	5	13	14.2912	41.1354	29
3	5	-13	67.8595	54.8921	19
-3	5	12	485.163	87.2790	29
3	-5	-11	190.053	67.4574	20
-3	5	11	68.9813	60.9812	29
-3	5	10	1849.50	207.685	29
3	-5	-10	2260.23	444.097	17
3	-5	-10	2180.68	228.050	15
3	-5	-10	1751.54	190.241	20
3	5	-10	2347.05	447.447	24
3	5	-9	964.510	292.811	24
3	-5	-9	1237.32	147.372	20
3	-5	-9	801.381	166.456	21
-3	5	9	769.990	234.595	30
-3	-5	9	1077.36	148.439	10

-3	5	9	897.198	163.912	29
3	-5	-9	1137.22	166.108	15
-3	-5	8	4281.09	277.979	10
-3	-5	8	4441.95	297.955	27
3	-5	-8	4270.49	563.717	17
3	-5	-8	4392.40	301.558	15
3	-5	-8	4255.18	304.416	3
-3	-5	7	18465.0	680.371	10
-3	-5	7	21581.1	732.061	27
3	-5	-7	19169.9	710.092	15
3	-5	-7	20517.0	732.585	3
3	-5	-6	22633.2	1358.85	1
-3	5	6	20025.6	1357.90	30
3	-5	-6	26402.9	1558.63	17
3	5	-6	22620.9	1549.14	24
-3	-5	6	22393.6	1193.92	27
3	-5	-5	93.6121	89.7895	1
-3	5	5	84.5142	70.2562	30
-3	-5	5	124.273	76.0106	30
3	-5	-5	74.2229	92.4749	17
3	5	-5	140.986	158.441	24
-3	-5	5	79.7025	64.5673	27
3	-5	-4	190.379	111.722	1
-3	-5	4	16.9651	54.5829	30
-3	5	4	140.452	85.8065	30
3	-5	-4	59.9732	68.1890	17
-3	-5	4	42.6401	50.8807	28
3	5	-4	42.4501	96.4915	24
3	-5	-3	82378.8	3243.65	1

-3	5	3	71046.7	3008.77	30
3	-5	-3	62789.0	3059.47	17
3	5	-3	76120.1	3298.97	24
-3	-5	3	68206.7	2904.73	28
3	-5	-2	5596.75	505.985	2
3	-5	-2	5280.40	444.078	1
-3	5	2	1440.45	174.628	30
3	-5	-2	2739.07	252.806	17
3	5	-2	4929.93	450.179	24
-3	-5	2	4260.43	214.844	28
3	-5	-1	54266.2	1680.71	2
3	-5	-1	62425.5	1639.72	1
-3	-5	0	16917.5	966.160	23
3	-5	0	16676.0	1175.41	2
3	-5	1	2946.97	384.256	1
3	-5	1	3175.88	418.769	2
-3	-5	-1	3056.26	300.956	23
-3	5	-1	2668.09	287.317	24
3	5	1	2447.42	261.931	25
3	-5	1	2025.59	205.211	25
3	-5	2	-46.6111	105.923	2
-3	-5	-2	-24.5383	70.5487	23
3	5	2	-48.2395	73.5497	25
3	-5	3	9874.22	870.281	2
-3	-5	-3	9508.17	708.814	23
3	5	3	8932.60	693.662	25
3	-5	3	8786.16	629.360	25
3	-5	4	19936.2	1397.54	2
3	-5	4	15806.6	1061.68	25

3	5	4	18052.3	1165.76	25
-3	-5	-4	16296.7	1131.77	23
3	-5	5	9386.25	575.795	5
3	-5	5	10935.0	1012.56	2
3	5	5	11065.4	591.836	29
3	-5	5	9822.66	573.275	7
3	5	5	10502.2	847.960	25
-3	-5	-5	10222.7	594.351	22
-3	-5	-6	19028.4	1071.63	22
-3	-5	-6	20511.5	1067.88	9
3	-5	6	23668.6	1698.90	2
3	5	6	21987.3	1075.31	29
3	-5	6	20459.5	1057.39	7
3	5	6	19846.8	1384.25	25
3	-5	6	19423.8	1060.69	5
3	-5	7	17822.9	1466.29	2
3	5	7	14778.5	861.686	29
3	-5	7	16273.4	870.253	5
3	-5	7	17261.2	867.485	7
3	5	7	15260.1	1196.52	25
-3	-5	-8	721.572	108.415	20
3	5	8	595.502	124.007	29
3	-5	8	597.769	115.678	5
3	-5	8	845.522	323.023	2
-3	-5	-8	402.674	196.432	23
3	-5	8	769.412	125.643	7
3	-5	9	319.017	87.0482	7
3	5	9	70.6796	70.8753	29
3	-5	9	240.678	80.6294	5

-3	-5	-9	217.480	76.2218	15
-3	-5	-9	122.503	68.5462	20
3	-5	10	1496.46	146.730	5
-3	-5	-10	1387.72	137.488	20
3	5	11	86.6860	52.8628	29
3	-5	12	162.641	62.5866	4
3	-5	13	1376.01	85.7340	10
3	-5	13	531.721	62.0378	8
3	-6	-14	47.7110	22.2558	11
3	6	-14	84.5572	26.5800	19
3	-6	-13	462.938	80.5019	11
3	6	-12	-34.5727	59.7322	19
-3	-6	10	503.777	106.731	10
3	-6	-10	901.123	155.065	15
-3	-6	9	559.419	114.894	10
3	-6	-9	537.951	127.707	15
3	6	-8	139.878	174.339	24
-3	-6	8	316.376	92.3446	10
3	-6	-8	225.258	102.546	15
-3	-6	8	165.631	84.6894	27
3	-6	-8	56.1342	114.477	17
3	-6	-8	228.446	92.8086	3
-3	-6	7	88.1805	73.1823	10
3	-6	-7	-32.2395	83.5399	3
-3	-6	7	63.0114	83.1937	27
3	6	-7	65.7067	133.545	24
3	-6	-7	100.083	124.566	17
3	-6	-6	5829.29	429.824	3
-3	-6	6	5391.90	408.546	10

3	-6	-6	6370.82	443.931	9
3	6	-6	6517.21	731.039	24
-3	-6	6	6539.09	433.295	27
3	-6	-6	6065.64	422.737	15
3	-6	-6	5561.42	614.848	17
3	-6	-5	2912.52	276.230	4
3	-6	-5	3787.00	299.586	3
3	-6	-5	3134.16	278.438	9
3	-6	-5	3441.38	404.859	1
3	-6	-5	2553.98	366.233	17
-3	-6	5	3621.70	286.891	27
3	-6	-5	3404.26	276.965	15
3	6	-5	3847.46	517.180	24
3	6	-4	2905.84	435.342	24
3	-6	-4	2931.44	385.496	1
-3	-6	4	3193.69	262.772	28
3	6	-3	11648.6	950.125	24
3	-6	-3	12725.8	915.491	1
-3	-6	3	11220.3	669.617	28
3	6	-2	13359.6	1030.24	24
3	-6	-2	13834.4	1071.56	2
3	-6	-2	15198.9	1036.37	1
-3	-6	2	13606.2	776.428	28
3	-6	-1	123.187	118.132	1
3	-6	-1	96.1267	138.236	2
3	-6	0	993.186	266.330	2
3	-6	1	2900.16	436.870	2
3	-6	1	2695.03	209.005	27
-3	6	-2	94.0678	84.3873	24

3	-6	2	55.5885	126.364	2
-3	6	-3	37241.2	2119.80	24
3	-6	3	35022.6	2283.97	2
3	-6	4	52826.8	2430.91	5
-3	-6	-4	55069.4	2440.87	22
-3	-6	-4	52554.8	2456.97	16
3	-6	4	59393.3	3075.41	2
-3	-6	-5	6466.28	409.017	22
-3	6	-5	6997.02	710.548	24
3	-6	5	5980.19	394.517	5
3	-6	5	5686.72	382.290	7
3	-6	5	6571.90	783.294	2
-3	6	-6	6771.32	721.143	24
-3	-6	-6	5835.17	386.754	9
3	-6	6	6212.75	381.654	7
3	-6	6	6488.40	395.054	5
-3	6	-7	2668.49	470.054	24
-3	-6	-7	2404.27	224.008	9
3	-6	7	2040.74	207.280	7
3	-6	7	2009.31	211.067	5
3	-6	8	1499.11	171.843	7
3	-6	8	1172.92	159.525	5
3	-6	9	1644.27	176.233	5
-3	-6	-9	1684.90	170.517	15
-3	-6	-10	1070.17	115.619	15
3	-6	10	980.018	122.942	5
3	-6	11	6689.24	405.066	4
3	-6	11	7602.52	420.064	10
3	-6	12	507.429	74.8409	10

3	-6	12	370.411	62.7621	4
3	7	-13	129.813	43.8579	19
3	-7	-13	52.6803	36.9134	11
3	7	-12	436.535	85.5934	19
3	-7	-12	485.667	90.2855	11
3	7	-11	36.8468	68.3821	19
-3	-7	11	39.8865	52.6814	8
3	-7	-10	629.437	128.556	15
-3	-7	10	472.589	105.723	8
-3	-7	10	587.918	108.060	10
-3	-7	9	2426.67	248.630	22
-3	-7	9	3083.21	268.682	10
3	-7	-9	3186.74	282.187	15
-3	-7	8	1537.54	197.352	22
3	7	-8	1523.99	379.143	24
-3	-7	8	2135.23	230.048	27
3	-7	-8	2158.77	233.400	3
-3	-7	8	1960.46	215.681	10
3	7	-7	1132.38	324.341	24
-3	-7	7	882.109	155.397	22
3	-7	-7	795.518	147.902	3
3	-7	-7	1055.49	163.850	15
-3	-7	7	792.938	146.273	27
-3	-7	7	920.438	150.868	10
-3	-7	6	3706.43	313.913	22
3	-7	-6	4029.02	339.147	9
-3	-7	6	3438.59	311.156	10
-3	-7	6	3758.29	315.357	8
-3	-7	6	4465.40	343.631	27

3	-7	-6	3821.74	317.555	15
3	7	-6	3213.99	535.041	24
3	-7	-6	3619.02	325.367	3
-3	-7	5	4513.84	352.070	10
3	-7	-5	4664.73	357.981	9
-3	-7	5	4162.64	335.960	8
-3	-7	5	3796.38	323.336	22
3	7	-5	4797.58	617.926	24
-3	-7	5	5228.63	372.765	27
3	-7	-5	3808.30	342.182	4
3	-7	-5	4349.73	356.476	3
3	-7	-5	4273.87	337.923	15
3	-7	-4	3131.81	272.291	15
3	7	-4	3530.30	522.356	24
-3	-7	4	2816.56	260.401	22
3	7	-4	3281.78	266.473	19
-3	-7	4	3362.88	287.821	10
-3	-7	4	3138.28	275.487	8
3	-7	-4	3717.90	294.511	9
3	-7	-4	3514.12	297.872	8
3	-7	-4	3155.00	290.522	4
3	-7	-4	3770.89	310.182	3
-3	-7	4	3247.58	290.721	28
3	7	-3	809.414	252.584	24
3	-7	-3	510.310	107.932	9
-3	-7	3	467.909	106.111	28
3	-7	-3	525.484	114.011	8
3	-7	-3	628.934	119.453	4
3	-7	-3	578.717	116.751	16

3	-7	-2	255.912	92.0735	3
3	-7	-2	207.012	87.0582	4
-3	-7	2	270.347	97.6536	28
3	-7	-1	2798.33	447.281	2
3	-7	-1	2295.16	226.131	4
3	-7	-1	2005.97	213.978	3
-3	-7	1	2490.50	232.685	28
3	-7	0	175.339	168.180	2
3	-7	1	1288.85	334.102	2
3	-7	1	1094.64	129.739	27
3	-7	1	1195.75	153.306	3
3	-7	1	1247.81	165.010	8
3	-7	1	966.041	129.094	5
3	-7	2	1525.11	199.358	8
3	-7	2	1616.67	188.802	10
-3	-7	-2	1839.19	203.027	8
-3	-7	-2	1983.92	210.285	16
3	-7	2	1802.30	177.049	7
3	-7	2	1986.22	191.616	5
3	-7	2	2100.71	208.272	3
-3	-7	-2	2003.61	190.475	22
3	-7	3	9938.50	600.439	10
3	-7	3	9369.72	964.584	2
-3	-7	-3	9828.76	602.492	16
3	-7	3	9461.19	559.405	7
3	-7	3	8735.88	563.142	5
3	-7	3	8420.99	593.432	4
3	-7	3	8709.69	567.248	3
-3	-7	-3	9982.90	574.338	22

3	-7	4	2249.58	236.112	10
3	-7	4	2653.48	489.582	2
-3	-7	-4	1884.41	225.489	16
3	-7	4	2030.84	204.553	7
3	-7	4	2069.24	207.134	3
3	-7	4	2048.13	233.601	4
-3	7	-4	1919.01	341.529	24
-3	-7	-4	2265.61	215.468	22
3	-7	5	623.652	264.113	2
-3	7	-5	274.873	150.252	24
3	-7	5	693.560	143.826	4
3	-7	5	595.720	123.842	5
-3	-7	-5	560.328	126.981	9
-3	-7	-5	681.696	126.175	22
3	-7	6	80.7123	183.389	2
-3	-7	-6	-14.9722	71.3588	9
-3	7	-6	60.1313	86.4102	24
3	-7	6	-38.5638	83.1258	4
3	-7	6	14.9047	66.0160	5
3	-7	6	-27.2476	70.5885	7
3	-7	7	3123.18	303.448	4
3	-7	7	3177.65	270.826	7
3	-7	7	2880.33	268.794	5
-3	-7	-7	2845.41	258.991	9
3	-7	8	53.1939	82.6420	4
3	-7	8	93.6001	74.5045	5
3	-7	9	1910.68	187.018	5
-3	-7	-9	1562.18	165.938	15
3	-7	9	1933.70	211.976	4

3	-7	10	2216.15	202.762	10
3	-7	10	1743.22	187.184	4
-3	-7	-10	1665.93	144.221	15
3	-7	10	1707.83	155.649	5
3	-7	11	4022.03	284.076	8
3	-7	11	4494.42	290.258	10
3	-7	11	4375.46	280.145	4
3	8	-12	271.701	58.9111	19
3	8	-11	56.2168	57.1261	19
3	-8	-11	-18.4585	57.0474	11
3	8	-10	191.093	76.2879	19
3	-8	-10	182.271	84.1957	15
-3	-8	10	163.408	66.2099	8
3	-8	-10	214.293	80.7150	11
-3	-8	9	52.1409	66.2372	8
-3	-8	9	13.3228	64.9347	10
3	8	-9	13.7256	66.8972	19
3	8	-8	6943.22	496.677	19
3	-8	-8	7485.74	512.128	3
-3	-8	8	8919.43	544.669	27
-3	-8	8	7289.75	502.772	8
-3	-8	8	8123.51	520.981	10
-3	-8	7	4763.83	366.408	27
3	8	-7	4996.99	664.228	24
-3	-8	7	3956.41	307.752	22
-3	-8	7	3441.98	322.490	10
-3	-8	7	3900.26	330.410	8
3	8	-7	4181.72	329.022	19
3	-8	-7	4267.14	344.118	15

3	-8	-7	3844.21	336.395	3
-3	-8	6	17.7753	80.7764	27
3	-8	-6	46.8822	77.7936	15
3	8	-6	-70.2646	142.796	24
-3	-8	6	12.9525	52.6465	22
3	8	-6	28.7144	63.5915	19
3	-8	-6	-34.8406	91.9790	3
-3	-8	6	-16.0429	63.1368	8
3	-8	-6	-17.9097	83.3970	9
-3	-8	6	51.4217	73.8947	10
-3	-8	5	12777.6	737.463	27
3	8	-5	13737.6	1153.35	24
3	-8	-5	12702.3	734.762	9
-3	-8	5	10463.3	698.037	8
3	8	-5	12211.3	694.144	19
3	-8	-5	11761.5	704.523	15
3	-8	-5	11443.3	721.882	3
-3	-8	5	10529.8	674.315	22
3	-8	-4	241.710	101.093	3
3	-8	-4	208.247	83.8370	15
3	8	-4	382.203	214.826	24
-3	-8	4	127.586	65.3085	22
-3	-8	4	124.214	86.7805	8
-3	-8	4	84.7211	91.4534	27
-3	-8	4	105.496	80.2958	28
3	-8	-4	195.137	92.2581	8
3	-8	-4	234.921	72.6101	11
3	-8	-4	177.698	93.1969	9
3	-8	-4	182.915	93.2728	4

-3	-8	3	2475.75	219.308	22
3	-8	-3	1919.01	221.337	16
-3	-8	3	2007.06	219.853	8
3	-8	-3	2057.80	233.521	8
3	-8	-3	2406.04	236.947	9
-3	-8	3	2609.96	251.335	28
3	-8	-3	2180.43	243.670	4
3	-8	-3	1898.06	200.635	15
-3	-8	2	18968.5	1018.69	8
3	-8	-2	16291.2	981.934	9
3	-8	-2	16983.9	1022.52	8
3	-8	-2	19031.2	1022.61	16
3	-8	-2	18222.8	1035.63	4
-3	-8	2	19407.9	1032.81	28
-3	-8	2	18059.1	980.122	22
-3	-8	1	-23.8822	51.4810	22
3	-8	-1	14.8224	60.2478	16
3	-8	-1	33.7201	80.3575	8
-3	-8	1	-30.2088	73.6081	8
3	-8	-1	11.6641	48.8703	5
3	-8	-1	-64.9510	263.995	2
3	-8	-1	-32.1348	76.5797	3
-3	-8	1	-33.1791	79.0682	28
-3	-8	0	13163.7	724.002	22
-3	-8	0	12369.5	749.807	8
3	-8	0	13254.5	773.919	8
3	-8	0	11724.2	732.415	16
3	-8	0	12121.4	724.086	5
3	-8	0	13012.3	763.891	3

3	-8	0	11897.0	1127.89	2
-3	-8	0	12951.1	769.282	28
-3	-8	-1	8367.27	535.629	8
3	-8	1	7926.25	541.326	8
3	-8	1	7511.42	514.883	10
-3	-8	-1	8200.03	539.232	16
3	-8	1	7396.78	503.055	16
3	-8	1	7760.75	502.401	5
3	-8	1	7627.14	476.779	7
-3	-8	-1	8027.24	495.263	22
3	-8	1	7904.50	524.992	3
-3	-8	-1	7813.90	499.800	3
3	-8	1	8055.52	889.688	2
3	-8	2	36329.4	2039.75	10
3	-8	2	40649.6	2080.00	8
-3	-8	-2	41792.8	2072.40	16
3	-8	2	38420.0	2003.26	7
3	-8	2	39939.6	2035.43	5
-3	-8	-2	41421.5	2021.67	22
3	-8	2	40384.0	2050.73	3
3	-8	2	40738.4	2082.21	4
3	-8	2	42945.5	2621.32	2
3	-8	3	3353.61	312.225	8
3	-8	3	3418.27	302.063	10
-3	-8	-3	3017.50	292.211	16
3	-8	3	3184.12	279.276	5
3	-8	3	3339.28	268.503	7
-3	-8	-3	2887.86	260.769	22
3	-8	3	2812.52	267.717	3

3	-8	3	3668.53	325.577	4
3	-8	3	3696.35	612.281	2
3	-8	4	974.995	165.124	10
-3	-8	-4	787.379	152.080	16
-3	-8	-4	907.526	138.880	22
3	-8	4	779.695	159.783	4
3	-8	4	832.564	135.397	3
3	-8	4	700.613	298.716	2
3	-8	4	798.587	132.461	7
3	-8	4	1051.64	176.075	8
3	-8	5	6174.89	789.235	2
3	-8	5	6064.73	410.834	7
3	-8	5	6314.86	430.136	5
3	-8	5	5791.79	448.576	4
3	-8	5	5082.55	382.525	3
-3	-8	-5	5948.45	424.694	9
3	-8	5	5408.66	430.719	10
3	-8	5	5794.18	453.102	8
3	-8	6	5751.45	420.458	5
3	-8	6	5948.95	451.451	4
3	-8	6	5349.93	443.156	8
-3	-8	-6	5633.65	411.094	9
3	-8	6	6066.78	448.600	10
3	-8	7	1187.59	184.908	8
3	-8	7	1086.06	181.451	4
3	-8	7	1208.39	166.308	5
-3	-8	-7	1118.92	158.150	9
3	-8	7	1047.72	173.018	10
3	-8	8	16.0869	80.1505	8

3	-8	8	159.802	79.0692	5
3	-8	8	45.9049	80.8616	4
-3	-8	-9	2840.95	217.951	15
3	-8	9	2976.81	228.860	5
3	-8	9	3019.04	253.675	4
3	-8	9	2803.50	251.779	8
-3	-8	-10	1888.87	141.947	15
3	-8	10	1986.52	181.477	8
3	-8	11	57.7269	28.5145	8
3	-8	11	87.2019	28.4544	4
3	-9	-11	471.417	65.9700	11
3	9	-11	478.469	65.9751	19
3	-9	-10	48.8963	49.8586	11
3	9	-10	139.113	56.5489	19
-3	-9	9	213.124	66.2180	8
3	-9	-9	196.777	73.7376	11
3	9	-9	309.223	79.8379	19
3	9	-8	706.047	116.021	19
3	-9	-8	571.357	110.846	11
-3	-9	8	666.598	115.302	8
3	-9	-8	774.406	136.046	15
3	-9	-8	731.393	131.246	3
3	-9	-7	152.239	76.3022	11
3	-9	-7	-15.2873	80.7512	3
3	9	-7	62.5086	64.8183	19
-3	-9	7	28.3859	66.1321	8
-3	-9	7	50.3416	81.8092	27
3	-9	-7	43.0813	81.2799	15
-3	-9	6	5863.51	423.986	27

3	9	-6	5084.06	364.885	19
3	-9	-6	5492.84	409.150	3
-3	-9	6	5000.36	387.305	8
3	-9	-6	4847.33	360.861	11
3	-9	-6	5149.39	382.247	15
3	-9	-5	52.8231	85.8130	3
-3	-9	5	-15.9572	68.8021	8
3	-9	-5	43.3022	68.8031	15
3	-9	-5	36.0323	53.2102	11
3	-9	-5	17.1315	81.6578	9
3	-9	-4	14497.8	839.492	15
3	-9	-4	14066.9	892.938	4
3	-9	-4	15417.3	890.802	3
3	-9	-4	15665.1	877.689	9
-3	-9	4	14472.9	863.582	8
3	-9	-3	1008.64	166.062	3
-3	-9	3	668.641	136.710	8
3	-9	-3	839.548	157.589	8
-3	-9	3	684.441	148.190	28
-3	-9	3	583.574	135.011	27
3	-9	-3	674.691	117.047	15
3	-9	-3	660.950	132.211	9
-3	-9	2	180.504	88.8444	8
3	-9	-2	111.790	93.3317	8
-3	-9	2	201.028	98.8143	28
3	-9	-2	205.035	80.8143	9
3	-9	-1	-16.1057	71.3379	16
-3	-9	1	33.0875	80.6232	8
3	-9	-1	37.3335	88.9692	8

3	-9	-1	105.911	98.2443	3
-3	-9	1	56.7438	52.8489	22
3	-9	-1	-39.7895	66.0268	9
3	-9	0	31435.8	1672.81	8
-3	-9	0	30508.2	1647.33	8
-3	-9	0	31739.8	1677.36	16
3	-9	0	31895.1	1641.97	16
-3	-9	0	31241.2	1596.10	22
-3	-9	0	31113.6	1631.08	3
3	-9	0	31484.5	1662.23	3
3	-9	0	30934.6	1671.99	4
3	-9	0	28955.1	2098.00	2
-3	-9	-1	4428.58	374.436	8
3	-9	1	4945.13	399.343	8
3	-9	1	5072.08	370.309	16
-3	-9	-1	4689.20	391.056	16
3	-9	1	4839.10	364.406	5
-3	-9	-1	4396.03	333.665	22
-3	-9	-1	4368.23	349.783	3
3	-9	1	5060.83	405.926	4
3	-9	1	5230.83	392.770	3
3	-9	1	5693.10	726.134	2
3	-9	2	8565.69	583.291	8
3	-9	2	9200.55	577.578	10
-3	-9	-2	9418.35	586.317	16
3	-9	2	7004.11	517.621	16
3	-9	2	7888.53	541.369	5
-3	-9	-2	8812.53	522.931	22
-3	-9	-2	8274.35	525.978	3

3	-9	2	8534.74	585.264	4
3	-9	2	8655.68	559.229	3
3	-9	2	7326.46	881.274	2
3	-9	3	6660.63	486.983	8
3	-9	3	6090.52	457.272	10
-3	-9	-3	6762.62	477.768	16
3	-9	3	6471.52	450.342	5
-3	-9	-3	6074.62	416.894	22
3	-9	3	5332.08	740.790	2
3	-9	3	7157.76	498.032	4
3	-9	3	5880.57	439.896	3
-3	-9	-4	2618.93	258.476	9
3	-9	4	2374.89	256.678	10
3	-9	4	2303.51	263.299	8
3	-9	4	2803.93	280.728	4
3	-9	4	2138.40	224.206	3
3	-9	5	581.245	145.497	8
3	-9	5	397.973	93.1884	3
-3	-9	-5	561.176	126.165	9
3	-9	5	587.206	137.688	10
3	-9	5	414.533	111.377	5
3	-9	5	382.600	121.958	4
3	-9	6	11210.5	703.259	8
-3	-9	-6	10867.0	662.239	9
3	-9	6	11386.0	696.599	10
3	-9	6	11014.8	694.387	4
3	-9	6	11357.0	676.433	5
3	-9	7	363.871	105.573	10
-3	-9	-7	456.081	97.4632	9

3	-9	7	546.636	122.787	4
3	-9	7	464.606	106.469	5
3	-9	7	386.076	112.871	8
3	-9	8	1182.05	147.354	4
3	-9	8	966.010	128.792	5
3	-9	8	1181.60	147.617	10
3	-9	8	1046.47	145.343	8
-3	-9	-8	1067.45	129.184	15
3	-9	9	841.680	112.401	8
-3	-9	-9	680.239	86.7022	15
3	-9	9	857.204	108.627	4
3	-9	10	157.689	35.3338	4
3	-9	10	140.928	39.5194	8
3	10	-9	261.839	48.9347	19
-3	-10	8	24.8663	44.5771	8
3	10	-8	20.5448	39.3755	19
3	-10	-8	9.97937	55.5487	15
-3	-10	7	76.2794	59.6426	8
3	-10	-7	34.2209	62.4323	15
3	-10	-7	24.7084	72.1253	3
-3	-10	7	-14.1119	71.7088	27
-3	-10	6	2728.58	245.468	8
3	-10	-6	2644.88	240.798	15
3	-10	-6	3161.46	267.515	3
3	-10	-5	1354.24	162.128	15
3	-10	-5	1130.29	175.060	3
-3	-10	5	1374.09	173.226	8
3	-10	-4	608.991	126.581	9
-3	-10	4	693.954	130.976	8

3	-10	-4	488.356	101.674	15
-3	-10	4	542.796	128.260	27
3	-10	-4	541.996	129.230	3
3	-10	-3	2709.26	236.482	15
-3	-10	3	3507.89	307.078	27
3	-10	-3	3221.79	303.725	8
3	-10	-3	3408.50	286.841	9
-3	-10	3	3041.97	280.729	8
3	-10	-3	3213.99	299.661	3
-3	-10	2	1278.92	179.504	27
3	-10	-2	926.649	171.613	8
-3	-10	2	1027.28	159.300	8
3	-10	-2	1049.61	150.476	9
3	-10	-1	3274.50	298.667	16
-3	-10	1	3720.58	330.169	16
-3	-10	1	4125.69	324.684	27
3	-10	-1	3257.73	317.887	8
-3	-10	1	3194.24	294.848	8
3	-10	-1	3832.30	333.748	4
-3	-10	1	3693.38	301.876	3
3	-10	-1	3842.18	323.336	3
3	-10	0	2701.62	280.995	4
3	-10	0	2653.90	271.817	3
-3	-10	0	2621.00	246.695	3
3	-10	0	2449.76	242.884	16
-3	-10	0	2670.63	270.409	16
-3	-10	0	2119.65	239.032	8
3	-10	0	2503.37	271.206	8
3	-10	1	-27.6675	61.2774	16

-3	-10	-1	-18.0944	86.2424	16
3	-10	1	-56.3723	85.3938	8
3	-10	1	75.4915	95.8895	4
-3	-10	-1	89.4743	87.2085	28
-3	-10	-1	-27.3008	67.9562	3
3	-10	1	-50.2051	88.3637	3
3	-10	2	4021.46	336.681	8
-3	-10	-2	3335.03	312.290	16
3	-10	2	2815.90	259.748	16
-3	-10	-2	3410.20	276.843	3
3	-10	2	3336.72	299.614	3
3	-10	2	3879.85	333.798	4
-3	-10	-2	4018.00	327.600	28
-3	-10	-2	3165.68	250.189	22
-3	-10	-3	371.892	113.457	28
-3	-10	-3	435.462	116.227	16
3	-10	3	327.030	116.093	8
3	-10	3	233.797	108.968	4
3	-10	3	185.842	83.2932	3
3	-10	4	9013.56	563.814	3
3	-10	4	10345.1	624.160	4
3	-10	4	9688.34	623.045	8
-3	-10	-4	9662.10	595.285	9
-3	-10	-4	8575.76	589.963	28
-3	-10	-5	-12.9808	65.9517	9
3	-10	5	58.0679	76.6497	10
3	-10	5	15.9293	77.6271	8
3	-10	5	46.4748	78.7079	4
-3	-10	-5	-57.0070	72.4087	28

-3	-10	-6	542.408	103.199	9
3	-10	6	555.031	116.785	10
3	-10	6	525.223	109.489	4
-3	-10	-6	387.993	91.9665	28
3	-10	6	586.578	121.089	8
3	-10	7	2088.76	196.313	8
3	-10	7	1871.31	185.554	10
-3	-10	-7	2072.86	180.156	15
3	-10	7	2296.47	200.913	4
-3	-10	-7	1763.25	173.911	28
3	-10	8	1165.71	123.814	4
3	-10	8	1237.61	129.606	8
-3	-10	-8	1062.96	107.683	15
3	-10	9	554.471	61.0007	8
-3	-11	8	271.087	62.9009	3
3	-11	-6	2312.11	200.845	3
-3	-11	6	1855.05	169.430	8
3	-11	-5	25.1285	68.7583	3
-3	-11	5	-10.3813	48.3497	8
-3	-11	5	93.1807	77.7080	27
-3	-11	4	133.084	83.5068	3
3	-11	-4	83.5447	81.4269	3
-3	-11	4	94.4068	68.0106	8
-3	-11	4	112.420	77.0526	27
-3	-11	3	1126.75	154.255	8
3	-11	-3	1681.41	196.238	3
-3	-11	3	1551.52	183.239	3
-3	-11	3	1461.67	194.605	16
-3	-11	3	1139.16	161.563	27

-3	-11	2	67.1880	68.7827	8
3	-11	-2	161.168	93.0183	8
-3	-11	2	173.363	84.7442	27
-3	-11	2	219.671	86.4052	3
3	-11	-2	216.957	94.7892	4
-3	-11	2	375.099	108.990	16
-3	-11	1	427.203	104.159	8
-3	-11	1	369.846	111.673	28
-3	-11	1	567.463	116.397	27
3	-11	-1	308.425	120.220	3
-3	-11	1	412.848	102.609	3
3	-11	-1	608.331	131.161	8
3	-11	-1	523.655	134.662	4
-3	-11	1	491.088	127.386	16
-3	-11	0	122.219	75.8927	27
-3	-11	0	205.307	91.0397	28
-3	-11	0	150.390	75.6532	3
3	-11	0	30.1851	78.5011	3
3	-11	0	230.953	100.812	8
3	-11	0	184.583	103.945	4
-3	-11	0	177.889	94.6488	16
-3	-11	-1	946.146	135.637	27
-3	-11	-1	993.173	153.820	28
3	-11	1	658.475	135.117	3
3	-11	1	943.918	158.778	8
-3	-11	-1	1107.50	140.127	3
3	-11	1	1019.75	165.480	4
-3	-11	-1	1001.06	165.770	16
-3	-11	-2	109.976	82.7898	9

-3	-11	-2	123.414	61.5946	27
-3	-11	-2	87.0547	79.5500	28
-3	-11	-2	114.389	59.9165	3
3	-11	2	156.668	86.1187	3
3	-11	2	110.162	90.5968	8
3	-11	2	62.9530	86.2638	4
-3	-11	-2	59.2420	83.9391	16
3	-11	3	1630.70	193.371	4
3	-11	3	1602.21	167.073	3
-3	-11	-3	1643.25	184.240	28
-3	-11	-3	1587.59	182.119	9
3	-11	3	1598.54	192.849	8
-3	-11	-4	541.885	99.7015	28
3	-11	4	608.447	115.367	4
-3	-11	-4	433.176	98.0168	9
3	-11	4	322.669	100.010	8
3	-11	5	4858.44	329.917	4
-3	-11	-5	3931.14	297.842	28
-3	-11	-5	4465.40	306.724	9
3	-11	5	4942.24	333.937	8
3	-11	6	8.91189	50.4407	4
3	-11	6	55.8923	53.5655	8
3	-11	7	1714.94	138.373	4
3	-11	7	1950.73	146.876	8
-3	-11	-7	1609.09	130.981	15
-3	-12	7	121.448	33.4007	16
-3	-12	6	788.489	93.6207	3
-3	-12	5	826.720	105.588	3
3	-12	-5	584.363	93.0462	3

-3	-12	4	1574.41	158.404	27
-3	-12	4	1633.52	169.791	16
-3	-12	4	1705.86	163.735	3
3	-12	-4	1633.00	164.626	3
-3	-12	3	1469.83	158.359	27
-3	-12	3	1666.54	175.381	16
3	-12	-3	1907.29	181.908	3
-3	-12	3	1934.03	174.590	3
-3	-12	2	4214.42	309.622	27
-3	-12	2	4566.17	313.227	3
-3	-12	2	5152.37	342.181	16
-3	-12	1	783.365	105.289	27
3	-12	-1	510.487	110.468	3
-3	-12	1	596.702	102.221	3
-3	-12	1	585.009	111.407	16
3	12	-1	677.846	82.4012	18
3	-12	0	-32.9297	71.4269	3
-3	-12	0	17.1000	52.1396	3
-3	-12	0	41.9205	64.7861	9
3	-12	0	-24.2581	65.2257	8
-3	-12	0	143.578	73.9619	16
3	-12	0	75.1658	73.1352	4
3	-12	1	4005.12	288.222	4
3	-12	1	4034.70	271.399	3
-3	-12	-1	2978.34	227.412	3
3	-12	1	3650.77	279.057	8
-3	-12	-1	3205.43	260.740	28
3	-12	2	551.808	103.819	8
3	-12	2	597.234	96.8589	3

3	-12	2	636.201	109.714	4
-3	-12	-2	576.307	95.8071	28
-3	-12	-2	493.616	100.017	9
3	-12	3	1017.79	124.429	4
3	-12	3	720.869	111.098	8
-3	-12	-3	807.223	103.629	28
-3	-12	-3	871.457	113.442	9
3	-12	4	536.782	90.2421	8
3	-12	4	529.234	89.4521	4
-3	-12	-4	529.943	74.9437	15
-3	-12	-4	504.920	81.3287	9
-3	-12	-5	13.5858	29.4796	15
3	-12	5	51.9321	33.6950	4
3	-12	5	-6.05661	35.8974	8
3	-13	-4	192.872	37.1730	3
-3	-13	3	121.239	39.6325	16
3	-13	-3	154.219	44.0935	3
-3	-13	3	109.792	33.4742	3
-3	-13	2	157.693	49.9921	16
-3	-13	2	107.583	36.2686	3
-3	-13	1	119.973	31.4241	3
-3	-13	1	8.68170	29.9007	9
3	-13	0	326.934	57.5320	3
-3	-13	0	93.4009	38.8275	9
3	-13	1	-4.21593	26.4155	3
-3	-13	-1	-24.2016	31.8811	9
4	0	-15	-6.37862	39.9558	20
-4	0	14	1156.19	137.181	29
4	0	-14	2054.92	175.556	20

-4 0 13-12.4140 67.9295 29

4 0 -13 13.4518 72.3284 20

-4 0 12 13592.9 833.178 29

4 0 -12 11609.9 812.793 20

-4 0 11-57.2220 153.841 30

4 0 -11-77.3472 175.746 17

4 0 -11 48.2489 74.8976 20

4 0 -10 8179.17 901.975 17

4 0 -10 6807.95 482.454 20

4 0 -9 140.966 175.460 17

4 0 -9 38.5835 67.9797 24

4 0 -9 86.2407 70.1154 20

4 0 -8 3494.90 416.838 24

4 0 -8 4715.00 604.785 17

4 0 -7-29.6947 60.3487 24

4 0 -7 153.878 164.819 17

4 0 -6 29208.6 1700.68 24

4 0 -6 33558.3 1899.58 17

4 0 -5 21.9738 66.9952 24

4 0 -5 34.8241 100.093 17

4 0 -4 71553.9 3587.01 24

4 0 -4 71489.6 3704.22 17

4 0 -3 21.1502 74.4484 25

4 0 -3-25.9303 79.0459 17

4 0 -2 12240.4 815.248 25

4 0 -2 14731.8 880.634 17

4 0 -1 36.9724 51.6426 2

4 0 -1 64.0361 75.1416 25

4 0 -1-22.5106 72.3396 17

4	0	0	5988.47	412.631	2
4	0	0	6045.23	488.999	25
4	0	0	5997.49	499.643	17
4	0	1	-50.9445	73.2101	25
4	0	1	-17.3442	58.4541	2
4	0	1	17.5018	58.9854	1
4	0	2	5505.80	504.964	25
4	0	2	5109.05	399.976	1
-4	0	-3	67.7453	114.232	23
4	0	3	94.1246	56.4907	1
-4	0	-4	13062.0	952.456	23
-4	0	-5	-42.8239	123.095	23
-4	0	-6	17730.3	1280.76	23
-4	0	-7	-56.1151	150.861	23
4	0	7	22.3117	54.3649	18
-4	0	-8	3217.23	519.903	23
4	0	8	3224.85	260.789	18
-4	0	-9	-63.0171	192.125	23
4	0	9	-69.4116	172.792	25
-4	0	-11	32.1772	65.4160	20
4	0	11	44.2675	74.9898	29
4	0	12	3792.22	280.084	29
-4	0	-12	3478.70	252.491	20
-4	0	-13	-15.6528	36.3545	20
4	1	-15	5.94973	38.7124	20
-4	-1	14	3034.55	249.000	29
4	1	-14	4962.99	295.133	20
4	1	-13	551.231	120.470	20
-4	-1	13	383.144	96.8174	29

-4	1	12	24168.9	1012.30	29
-4	-1	12	22822.1	989.679	29
4	-1	-12	16308.5	908.289	11
4	1	-12	18031.4	962.122	20
4	1	-11	4340.78	648.226	17
4	-1	-11	2997.79	568.233	17
-4	1	11	4299.84	576.205	30
-4	1	11	5038.38	378.338	29
-4	-1	11	3540.99	524.408	30
-4	-1	11	4246.54	373.975	29
4	1	-11	4254.60	346.195	20
4	1	-10	-144.555	164.224	17
4	-1	-10	395.313	241.014	17
4	1	-10	-48.6560	110.553	24
-4	1	10	55.7340	138.722	30
-4	-1	10	54.4492	123.716	30
-4	-1	10	16.4319	81.7981	29
4	1	-10	124.291	80.4981	20
-4	1	9	7101.15	713.324	30
-4	-1	9	6712.22	696.523	30
4	1	-9	6717.98	773.072	17
4	1	-9	5305.20	612.414	24
4	-1	-9	5641.15	425.520	20
4	1	-9	6341.68	435.330	20
4	-1	-8	-130.527	148.293	17
4	1	-8	57.9830	117.845	17
4	1	-8	42.1520	95.7850	24
-4	-1	8	45.5908	122.575	30
-4	1	8	139.426	124.953	30

4	1	-7	36451.1	2103.30	17
4	-1	-7	39671.8	2180.19	17
4	1	-7	32020.9	1938.22	24
4	-1	-7	32180.2	1811.10	24
-4	-1	7	32451.7	1960.19	30
-4	1	7	33016.5	1972.71	30
4	1	-6	40.7857	109.660	17
4	-1	-6	19.1140	58.2875	24
4	1	-6	30.7951	62.6028	24
-4	-1	6	32.4938	80.8920	30
-4	1	6	98.2472	124.522	30
4	1	-5	13174.0	897.090	17
-4	-1	5	13035.4	872.712	30
4	1	-5	11787.7	811.987	24
4	-1	-5	10936.5	719.585	24
4	1	-4	109887.	5611.05	24
4	1	-4	118778.	5686.10	17
4	1	-3	38893.8	2144.47	25
4	1	-3	41258.5	2170.78	24
4	1	-3	42740.6	2205.41	17
4	1	-2	19287.7	1115.13	25
4	1	-2	19098.5	1145.68	17
4	-1	-2	18940.7	1095.31	2
4	1	-1	1902.55	244.402	25
4	1	-1	2160.71	274.599	17
4	-1	-1	2327.18	244.648	2
4	1	0	2147.50	262.345	25
4	-1	0	1615.80	235.980	25
4	-1	0	2499.54	282.174	17

4	-1	0	1990.74	246.660	1
4	1	0	1927.08	161.143	2
4	-1	0	1968.80	236.984	2
4	-1	1	19879.6	1145.42	2
4	-1	1	18378.2	1109.59	1
4	1	1	19786.8	1182.57	25
4	-1	1	19795.6	1172.91	25
4	-1	2	15268.6	950.055	1
4	1	2	15093.1	886.304	1
4	1	2	16475.1	1054.49	25
4	-1	2	16859.0	1058.14	25
4	-1	3	77594.5	3689.00	1
4	-1	3	74103.0	3844.56	25
4	1	3	75046.3	3868.91	25
4	1	3	73775.8	3576.32	29
4	-1	3	68597.2	3570.47	29
4	-1	4	-39.6655	98.7413	25
4	1	4	40.8129	92.7475	25
4	1	4	63.0659	61.3775	29
4	-1	4	30.6455	49.8083	29
-4	1	-4	108.755	116.499	23
-4	1	-5	1515.77	289.804	23
-4	-1	-5	2182.68	348.375	23
4	1	5	1754.37	340.030	25
4	-1	5	1327.90	301.994	25
4	1	5	1464.04	172.492	29
4	-1	5	1516.29	169.124	29
4	1	6	336.391	213.512	25
4	-1	6	-54.4206	166.347	25

4	-1	6	437.607	99.9899	29
4	1	6	396.491	104.481	29
-4	1	-6	195.376	157.427	23
-4	-1	-6	454.037	205.415	23
4	1	7	12885.6	778.004	29
4	-1	7	14122.6	789.104	29
4	-1	7	14811.8	1174.18	25
4	1	7	11928.8	1101.94	25
-4	1	-7	11598.8	1048.28	23
-4	-1	-7	14389.0	1135.87	23
4	1	7	12671.4	732.688	18
4	-1	7	13203.0	744.328	18
4	1	8	9767.92	601.446	29
4	-1	8	9741.24	598.718	29
4	1	8	8684.49	919.232	25
4	-1	8	9794.60	950.725	25
-4	1	-8	7134.77	810.468	23
-4	-1	-8	9186.27	903.066	23
4	1	8	8252.46	526.921	18
4	1	9	5692.51	415.256	29
4	-1	9	5715.07	410.953	29
-4	-1	-9	5554.48	683.996	23
4	1	9	5000.27	684.076	25
4	-1	9	5459.20	708.489	25
4	-1	10	5530.38	351.556	5
-4	1	-10	5192.15	370.654	20
4	1	10	6354.61	433.684	29
4	-1	10	5308.85	409.108	29
-4	-1	-11	488.420	99.0808	20

-4	1	-11	343.999	85.7000	20
4	1	11	511.312	109.851	29
4	-1	11	373.636	102.030	29
-4	-1	-12	1373.78	139.224	20
-4	1	-12	1651.00	146.013	20
4	1	12	1664.62	170.080	29
4	-1	12	1144.31	145.520	29
-4	-1	-13	189.445	46.7121	20
-4	1	-13	230.790	46.7136	20
4	2	-15	32.7900	30.8834	20
4	-2	-15	30.5438	32.7494	20
-4	2	14	343.281	73.9573	29
-4	-2	14	254.265	66.7597	29
4	2	-14	481.657	87.9947	20
4	2	-13	2003.29	199.048	20
4	-2	-13	2359.40	210.788	20
4	-2	-13	1570.13	160.626	11
4	2	-13	1726.53	170.809	19
-4	2	13	1914.19	190.036	29
-4	-2	13	1807.58	178.875	29
-4	2	12	5862.60	403.611	29
-4	-2	12	6072.41	396.053	29
4	-2	-12	5197.45	393.231	20
4	2	-12	4837.40	383.042	20
4	2	-11	412.815	225.286	17
-4	2	11	924.113	163.156	29
-4	-2	11	1011.86	163.933	29
4	2	-11	822.059	141.456	20
4	-2	-11	888.863	152.377	20

-4	2	11	397.608	196.267	30
-4	-2	11	815.818	256.935	30
4	2	-10	11018.1	1037.08	17
4	-2	-10	9293.87	580.947	20
4	2	-10	9751.22	586.486	20
-4	2	10	8832.26	867.983	30
-4	-2	10	7476.58	804.938	30
-4	2	10	9527.59	612.653	29
-4	-2	10	7625.31	575.636	29
4	2	-10	8951.48	862.083	24
4	2	-9	12136.0	1034.60	17
4	2	-9	9678.91	613.409	20
4	-2	-9	9735.46	621.673	20
-4	2	9	9455.31	892.950	30
-4	-2	9	11882.4	943.177	30
4	2	-9	9927.75	905.932	24
4	2	-8	1368.04	325.235	17
4	-2	-8	1102.11	314.990	17
4	-2	-8	1246.92	157.205	20
-4	-2	8	1373.04	300.434	30
-4	2	8	1381.91	292.928	30
4	2	-8	1515.33	312.246	24
4	2	-7	4516.87	514.826	24
4	2	-7	4496.60	548.321	17
4	-2	-7	5146.57	601.008	17
-4	2	7	4145.88	500.068	30
-4	-2	7	3913.88	487.561	30
4	2	-6	157.681	132.960	17
4	-2	-6	497.459	172.025	17

-4	-2	6	64.4178	118.127	30
-4	2	6	168.189	145.115	30
4	2	-6	36.3997	128.239	24
4	2	-5	3392.74	398.077	24
4	2	-5	3808.55	434.001	17
4	-2	-5	4038.17	452.925	17
4	2	-4	2165.96	307.382	24
4	2	-4	2360.72	305.152	17
4	-2	-4	2731.70	344.869	17
4	-2	-3	223156.	10924.6	25
4	2	-3	221546.	10990.3	24
4	2	-3	223273.	10924.6	25
4	2	-3	235140.	10992.8	17
4	-2	-3	235895.	11016.5	17
4	2	-2	189.967	105.356	25
4	-2	-2	163.356	85.8971	25
4	2	-2	210.325	114.145	17
4	-2	-2	225.499	105.256	17
4	-2	-2	139.958	102.843	1
4	-2	-2	97.2121	85.8932	2
4	-2	-1	1526.59	213.342	25
4	2	-1	1559.78	230.679	25
4	2	-1	1298.84	228.227	17
4	-2	-1	1619.85	225.316	17
4	-2	-1	1296.24	217.066	2
4	-2	-1	1631.43	237.776	1
4	-2	0	78599.9	4038.23	25
4	2	0	82812.5	4059.68	25
4	-2	0	80582.0	4088.89	17

4	-2	0	79999.1	4064.30	2
4	-2	0	81273.7	4059.41	1
4	-2	1	67829.7	3491.89	25
4	2	1	69109.5	3517.51	25
4	-2	1	67286.5	3519.03	2
4	-2	1	69209.8	3477.44	1
4	2	2	60843.3	3058.39	25
4	-2	2	58654.4	3028.19	25
4	2	2	50935.4	2810.03	1
4	-2	2	59762.4	2966.04	1
4	-2	2	60569.0	3073.43	2
4	-2	3	31382.2	1694.72	1
4	2	3	28886.4	1530.45	29
4	-2	3	28957.4	1529.05	29
-4	2	-3	34808.0	1800.53	23
4	-2	3	31255.5	1786.15	25
4	2	3	30934.7	1805.51	25
4	2	4	1146.82	139.479	29
4	-2	4	1100.91	134.035	29
-4	-2	-4	1190.18	272.511	23
-4	2	-4	1384.48	272.573	23
4	2	4	869.450	232.503	25
4	-2	4	1025.37	225.626	25
4	2	5	18404.5	990.460	29
4	-2	5	18326.5	986.141	29
4	2	5	18858.1	1320.38	25
4	-2	5	17409.9	1288.11	25
-4	2	-5	17490.1	1235.47	23
-4	-2	-5	18254.8	1272.80	23

-4	2	-6	335.037	201.150	23
-4	-2	-6	412.719	216.722	23
4	-2	6	401.201	89.8544	18
4	-2	6	320.993	78.9263	6
4	2	6	56.1285	171.834	25
4	-2	6	214.995	173.436	25
4	-2	6	368.952	99.4554	29
4	2	6	370.624	105.647	29
-4	2	-7	317.474	179.192	23
4	2	7	426.945	89.2049	18
4	-2	7	319.731	102.973	29
4	2	7	394.134	112.382	29
4	2	7	562.172	254.497	25
4	-2	7	359.775	236.639	25
4	-2	7	352.451	79.0538	7
4	2	8	10687.3	645.309	29
4	-2	8	11447.4	646.582	29
4	2	8	10734.3	581.876	18
4	2	8	8980.06	951.498	25
4	-2	8	10988.4	1012.64	25
4	-2	8	9298.58	561.364	7
-4	2	-9	58.5837	134.842	23
4	-2	9	565.730	102.474	5
4	2	9	690.938	138.768	29
4	-2	9	437.984	119.311	29
4	2	9	546.399	241.377	25
4	-2	9	461.119	201.962	25
-4	2	-10	689.811	113.869	20
4	-2	10	600.284	95.1437	5

4	2	10	654.739	137.595	29
4	-2	10	726.158	134.870	29
-4	2	-11	397.159	82.3794	20
-4	-2	-11	363.678	92.3177	20
4	2	11	362.220	99.0823	29
4	-2	11	146.394	74.1726	29
-4	-2	-12	1259.89	120.613	20
-4	2	-12	1214.12	113.943	20
-4	-2	-13	279.185	47.0464	20
-4	2	-13	214.978	35.7331	20
-4	-3	15	70.0514	32.0581	22
-4	3	14	455.437	78.1603	29
4	3	-14	577.223	85.9143	20
4	-3	-14	679.761	78.1077	11
4	3	-13	1245.80	142.940	20
4	-3	-13	1257.69	148.059	20
-4	-3	13	787.262	109.493	29
4	3	-13	1182.28	139.575	19
4	-3	-13	1194.64	136.507	11
-4	3	13	1066.50	134.863	29
4	3	-12	186.414	76.7606	20
4	-3	-12	67.7832	73.1262	20
-4	-3	12	103.539	62.1328	29
-4	3	12	161.393	82.2062	29
4	-3	-11	76.0500	155.724	17
4	3	-11	59.5054	122.423	24
4	3	-11	469.768	108.220	20
4	-3	-11	592.900	121.991	20
-4	3	11	490.332	117.033	29

-4	-3	11	600.477	120.006	29
4	3	-10	2597.24	451.841	24
4	-3	-10	2157.70	491.426	17
4	3	-10	2172.60	435.536	17
-4	3	10	2533.44	420.283	30
-4	-3	10	1899.70	361.605	30
-4	3	10	3073.52	281.444	29
-4	-3	10	2085.77	237.582	29
4	-3	-10	2960.50	328.119	21
4	3	-10	2856.31	258.432	20
4	-3	-10	2800.61	256.385	20
4	3	-9	9188.20	894.694	17
4	-3	-9	9763.22	1004.68	17
-4	3	9	8143.24	810.969	30
-4	-3	9	8290.20	794.517	30
4	3	-9	9887.85	927.779	24
4	-3	-9	8238.59	563.849	20
4	3	-9	8357.05	560.814	20
4	-3	-8	11162.7	689.498	20
4	3	-8	12246.1	699.548	20
-4	-3	8	11630.3	952.569	30
-4	3	8	11613.3	972.626	30
4	3	-8	14224.2	1071.01	17
4	-3	-8	13750.4	1143.09	17
4	-3	-8	11983.3	716.679	15
4	-3	-8	12862.1	928.265	1
-4	-3	8	12174.1	689.748	27
4	3	-8	11567.0	1020.06	24
4	3	-7	1064.06	271.866	24

4	3	-7	1258.18	277.187	17
4	-3	-7	2055.38	391.942	17
4	-3	-7	1232.06	249.053	1
-4	-3	7	1362.16	147.428	27
-4	-3	7	1187.60	250.925	30
-4	3	7	1411.42	286.627	30
-4	-3	6	2255.56	336.539	30
-4	3	6	2530.16	355.099	30
4	-3	-6	3124.40	444.494	17
4	3	-6	2825.18	378.979	17
4	-3	-6	2374.70	354.583	1
-4	-3	6	2665.60	210.372	27
4	3	-6	3350.81	423.283	24
4	-3	-5	739.427	220.220	17
4	3	-5	950.708	216.979	17
4	-3	-5	860.824	213.114	1
-4	3	5	688.334	189.209	30
4	3	-5	1056.32	227.039	24
4	3	-4	7276.19	579.051	17
4	-3	-4	7219.64	605.300	17
4	-3	-4	7126.13	612.049	1
4	3	-4	7061.70	607.119	24
-4	3	4	5485.59	534.924	30
4	-3	-3	14208.3	934.663	1
4	3	-3	12959.8	866.488	17
4	-3	-3	13666.1	904.527	17
4	3	-3	12373.8	826.293	25
4	-3	-2	66212.1	3527.53	2
4	3	-2	66693.6	3474.69	17

4	-3	-2	73048.8	3500.69	17
4	-3	-2	67314.0	3417.96	25
4	3	-2	67225.9	3430.87	25
4	-3	-1	8022.63	648.380	2
4	-3	-1	8460.63	652.037	1
4	-3	-1	8548.98	609.374	17
4	3	-1	8574.36	596.142	25
4	-3	-1	7559.87	564.721	25
4	3	0	154837.	7641.55	25
4	-3	0	156347.	7618.38	25
4	-3	0	151174.	7712.04	2
4	-3	0	164868.	7702.47	1
4	3	1	4806.13	450.112	25
4	-3	1	5006.68	443.369	25
4	-3	1	5037.84	496.643	2
4	-3	1	4758.89	458.054	1
4	-3	2	77120.5	3947.92	25
4	3	2	76804.5	3978.72	25
4	3	2	78243.4	3732.12	29
4	-3	2	77544.8	3949.73	1
4	-3	2	81625.0	4074.83	2
-4	-3	-3	23034.3	1308.26	23
-4	3	-3	21580.2	1258.66	23
4	-3	3	20488.8	1265.27	25
4	3	3	22475.3	1313.49	25
4	-3	3	18075.0	1013.35	29
4	3	3	19918.1	1030.13	29
4	-3	3	18843.2	1224.90	1
4	-3	3	18201.2	1337.34	2

4	-3	4	26265.9	1636.17	25
4	3	4	26367.5	1669.51	25
-4	-3	-4	28573.0	1669.35	23
-4	3	-4	27374.8	1610.38	23
4	3	4	26702.6	1372.53	29
4	-3	4	25914.7	1360.99	29
4	3	5	990.698	141.647	29
4	-3	5	925.927	135.601	29
4	3	5	987.747	254.768	25
4	-3	5	916.693	241.544	25
-4	3	-5	650.218	215.131	23
-4	-3	-5	1248.82	285.422	23
4	3	6	13345.2	763.932	29
4	-3	6	14321.9	761.899	29
4	3	6	12252.0	712.653	18
-4	-3	-6	14098.1	1101.22	23
-4	-3	-6	12673.1	744.204	22
4	3	6	14247.5	1121.33	25
4	-3	6	12804.6	1069.24	25
4	-3	6	12858.4	714.782	7
4	3	7	313.146	79.3911	18
4	3	7	304.295	103.484	29
4	-3	7	194.319	90.4983	29
-4	3	-7	99.9028	134.720	23
-4	-3	-7	231.371	186.176	23
4	-3	7	257.501	79.3485	7
4	3	7	312.185	210.695	25
4	-3	7	-58.3283	213.968	25
4	-3	8	3094.07	239.012	5

-4 -3 -8 2950.46 499.647 23

4 -3 8 2419.58 207.923 7

4 3 8 2176.76 432.302 25

4 -3 8 2163.02 425.732 25

4 3 8 3191.60 273.675 29

4 -3 8 2189.22 237.971 29

4 -3 9-102.524 66.5198 5

4 -3 9-9.79252 49.7853 7

4 3 9 16.6926 79.5789 29

4 -3 9-15.7673 67.9981 29

-4 -3 -9 125.345 168.500 23

-4 -3 -10 283.891 78.6739 20

-4 3 -10 462.857 88.9580 20

4 -3 10 170.035 63.4622 5

4 3 10 291.208 94.2839 29

4 -3 10 228.538 84.3172 29

4 -3 11 112.981 61.5490 29

4 3 11 251.672 79.4405 29

-4 -3 -11 274.647 74.4968 20

-4 -3 -11 130.572 93.4000 21

-4 3 -11 291.630 68.4524 20

4 3 12-17.8247 43.4381 29

-4 3 -12 47.9872 37.9329 20

-4 -3 -12 19.5995 40.3878 20

4 -3 13 4.49975 25.0437 4

-4 -3 -13-2.86788 19.3302 20

4 -4 -14 10.7910 32.8954 11

4 4 -14-6.18499 36.1033 19

4 4 -13 205.946 72.1743 19

-4	4	13	126.226	57.0344	29
4	-4	-13	149.225	56.9714	20
4	4	-13	214.295	63.7297	20
4	-4	-13	175.934	66.3594	11
-4	4	12	11.6003	58.9376	29
4	-4	-12	-11.6686	59.2846	20
4	4	-12	-11.2621	54.8828	20
-4	4	11	68.5766	76.4477	29
-4	-4	11	77.0178	54.9295	29
4	-4	-11	107.346	68.3011	20
4	4	-11	105.103	69.4932	20
4	-4	-10	1844.22	436.700	17
-4	4	10	1785.02	206.222	29
-4	-4	10	1089.06	172.828	29
-4	4	10	831.266	252.616	30
4	4	-10	1794.66	388.928	24
4	4	-10	1906.71	192.744	20
4	-4	-10	1887.69	197.998	20
-4	4	9	8018.12	764.368	30
-4	-4	9	6849.59	699.046	30
-4	4	9	7308.60	513.734	29
-4	-4	9	3605.68	433.512	29
4	-4	-9	12787.9	596.595	15
4	-4	-9	13146.7	1090.15	17
4	-4	-9	11777.3	554.518	20
4	4	-9	11367.6	546.802	20
4	4	-9	10598.3	957.913	24
4	-4	-8	1205.87	149.007	20
4	4	-8	1023.08	140.792	20

-4	4	8	928.486	255.505	30
-4	-4	8	1231.75	269.440	30
4	-4	-8	1682.75	381.707	17
4	-4	-8	1139.33	164.438	15
-4	-4	8	1206.11	155.216	27
4	4	-8	1588.90	355.483	24
4	4	-7	441.333	218.288	24
4	-4	-7	635.055	122.181	3
-4	-4	7	802.899	203.141	30
4	4	-7	378.179	155.612	17
4	-4	-7	445.713	197.421	17
-4	-4	7	550.622	103.551	27
4	-4	-7	317.987	92.7723	15
4	-4	-7	331.460	136.825	1
-4	-4	6	9050.13	551.370	27
4	-4	-6	9862.25	781.468	1
4	-4	-6	10356.3	858.243	17
4	4	-6	9360.63	763.219	17
4	4	-6	8519.96	824.193	24
-4	-4	6	9883.51	741.971	30
-4	4	6	9852.34	765.478	30
4	-4	-5	47174.2	2348.46	17
4	4	-5	45588.4	2235.02	17
4	-4	-5	46386.0	2307.84	1
-4	-4	5	39660.8	1981.94	28
-4	4	5	37893.9	2177.30	30
4	-4	-4	27896.6	1390.84	17
4	4	-4	26406.0	1295.03	17
4	-4	-4	30475.0	1444.74	1

-4	-4	4	25550.6	1079.36	28
-4	-4	3	15362.3	745.636	28
4	-4	-3	17779.2	1050.43	1
4	4	-3	17019.5	941.041	17
4	-4	-3	18914.9	1017.07	17
4	4	-3	15302.2	1030.86	24
4	4	-2	758.464	147.465	25
4	-4	-2	617.030	153.107	17
-4	-4	2	615.387	89.5008	28
4	-4	-1	77.2026	68.1403	25
4	4	-1	172.891	87.9962	25
4	-4	-1	95.0153	93.6292	17
4	-4	-1	132.573	121.541	1
4	4	0	9988.16	621.744	25
4	-4	0	9114.12	570.560	25
4	-4	0	10247.6	704.492	1
4	4	1	27.6648	74.3746	25
4	-4	1	-23.4886	75.4754	25
-4	-4	-1	-30.2449	86.9254	23
4	-4	1	33.4450	101.953	1
4	4	2	24691.5	1357.87	25
4	-4	2	22968.9	1298.13	25
4	-4	2	22854.5	1380.90	1
-4	-4	-2	25307.7	1361.48	23
-4	4	-2	23079.2	1295.33	23
4	-4	3	26.2792	40.7901	29
4	4	3	-10.0661	46.8731	29
4	-4	3	32.4998	87.3734	25
4	4	3	-37.1234	92.4002	25

-4	-4	-3	-71.4461	102.670	23
-4	4	-3	87.4008	98.1839	23
4	4	4	196.012	70.4183	29
4	-4	4	91.0689	59.2104	29
4	4	4	43.0902	107.331	25
4	-4	4	76.2461	102.575	25
-4	4	-4	-33.5831	83.6908	23
-4	-4	-4	82.6096	111.122	23
4	4	5	99.5500	123.964	25
4	-4	5	44.1702	127.020	25
-4	4	-5	76.5198	116.710	23
-4	-4	-5	47.4035	118.065	23
-4	-4	-5	100.198	65.0352	22
4	-4	5	116.640	62.0438	7
4	4	5	26.3436	65.7090	29
4	-4	5	253.674	72.3531	29
4	-4	5	57.4910	53.7125	5
4	-4	6	3185.60	266.263	5
4	4	6	2978.04	245.028	18
4	-4	6	3066.82	252.853	7
4	4	6	3248.23	485.726	25
4	-4	6	3653.12	489.279	25
4	4	6	3137.30	281.310	29
4	-4	6	3370.45	272.900	29
-4	-4	-6	2427.87	434.762	23
-4	-4	-6	3489.73	284.483	22
-4	-4	-7	-56.9550	208.732	23
4	-4	7	80.3437	163.363	2
4	-4	7	116.571	68.2468	7

4	4	7	-61.0773	240.424	25
4	-4	7	54.5157	259.879	25
4	-4	7	180.403	81.9383	5
4	4	7	238.127	92.8146	29
4	-4	7	126.592	75.8113	29
4	-4	8	30401.4	2032.26	2
4	-4	8	27927.2	1309.61	5
-4	-4	-8	24023.8	1705.77	23
4	-4	8	28021.9	1296.31	7
4	4	8	25455.5	1762.81	25
4	-4	8	19128.4	1277.20	29
4	4	8	23321.9	1317.42	29
-4	4	-9	128.065	60.2467	20
4	-4	9	145.555	67.8114	7
4	-4	9	92.6814	68.8055	5
4	4	9	108.896	85.2574	29
4	-4	9	13.6947	72.4627	29
4	4	10	13.6183	69.1920	29
4	-4	10	9.42274	50.6679	5
-4	4	-10	8.50219	44.0558	20
-4	-4	-10	19.4099	51.2449	20
4	4	11	21.0446	51.2941	29
-4	4	-11	41.9830	35.5600	20
-4	-4	-11	7.62777	45.2137	20
4	-4	11	35.6602	38.2181	5
4	-5	-14	547.628	61.9776	11
4	5	-14	449.130	64.0615	19
4	-5	-13	672.914	96.9067	11
4	5	-13	625.853	97.1271	19

-4	5	12	102.546	55.7375	29
4	5	-12	127.252	79.0064	19
-4	5	11	3779.47	273.543	29
4	-5	-11	3016.81	253.381	20
-4	5	10	3302.62	282.958	29
4	-5	-10	3488.11	273.064	20
4	5	-10	2837.04	507.043	24
4	-5	-9	48.8865	52.6886	20
4	-5	-9	66.6859	95.8294	17
4	-5	-9	33.2631	81.0487	15
-4	5	9	-27.5353	64.1093	29
-4	5	9	-46.8402	116.585	30
4	5	-9	-70.1145	159.310	24
4	-5	-8	-49.9861	86.3328	3
-4	-5	8	29.6532	62.1211	27
4	-5	-8	186.101	166.774	17
4	-5	-8	-32.1214	74.7893	15
4	5	-8	67.3209	152.964	24
-4	5	8	26.9690	71.2006	29
-4	5	8	-43.9495	118.157	30
-4	-5	7	3067.31	261.631	27
-4	5	7	2665.21	383.585	30
4	-5	-7	2597.18	442.419	17
4	-5	-7	2968.56	283.179	3
4	-5	-7	3104.01	386.841	1
4	-5	-6	110.061	74.1603	15
4	-5	-6	47.5705	118.520	17
4	-5	-6	38.5437	87.7352	1
4	-5	-6	193.609	83.0556	3

-4	-5	6	127.931	74.8623	27
-4	5	6	110.143	105.650	30
4	5	-5	2274.11	410.221	24
4	-5	-5	2565.81	378.075	17
4	-5	-5	2591.87	372.095	1
-4	-5	5	2483.63	214.385	28
-4	-5	5	1710.76	278.793	30
-4	5	5	1822.36	320.633	30
4	-5	-4	7208.04	635.878	17
4	-5	-4	8666.06	688.768	1
-4	-5	4	8034.07	429.279	28
4	5	-4	8234.77	720.396	24
4	5	-3	44.7543	120.517	24
4	-5	-3	116.444	93.7970	17
4	-5	-3	388.438	153.024	1
-4	5	3	67.8511	69.2908	30
-4	-5	3	219.788	65.9211	28
4	-5	-2	9547.76	751.874	1
4	-5	-2	8458.27	621.861	17
-4	-5	2	8070.72	497.256	28
4	5	-1	9268.17	626.532	25
-4	-5	0	15497.9	1026.03	23
4	5	0	16602.7	1004.97	25
4	-5	0	16207.0	954.757	25
-4	-5	-1	5952.23	513.975	23
4	5	1	5204.08	481.147	25
4	-5	1	5499.67	443.424	25
4	-5	2	128.099	130.165	1
4	5	2	62.9045	84.5571	25

4	-5	2	-23.2627	52.8559	25
-4	-5	-2	-31.2015	77.6606	23
4	-5	3	4774.93	446.611	25
4	5	3	5280.02	516.030	25
4	5	4	2899.83	231.498	29
4	5	4	2575.09	389.352	25
4	5	5	14300.6	778.330	29
4	-5	5	13725.0	760.188	7
4	5	5	13745.0	749.937	18
4	5	5	13851.9	1081.11	25
4	-5	5	13074.4	763.123	5
4	-5	6	2164.74	207.311	5
4	5	6	1566.97	352.571	25
4	-5	6	1863.76	192.377	7
-4	-5	-6	2096.23	211.165	22
4	5	6	1756.47	194.138	29
4	-5	7	10668.1	577.879	7
4	-5	7	10256.9	579.927	5
4	-5	8	287.631	88.1665	7
4	5	8	59.1939	69.3547	29
4	-5	8	221.536	87.1860	5
-4	-5	-9	5649.41	286.985	20
4	-5	9	5673.71	302.171	7
4	-5	9	5919.19	314.485	5
-4	-5	-10	5543.74	249.149	20
4	-5	10	5689.62	266.614	5
4	-5	11	86.8764	37.6682	5
4	-5	12	172.520	49.0059	10
4	-5	12	132.880	42.6629	4

4	6	-14	-9.40738	19.1234	19
4	-6	-13	2173.55	171.853	11
4	-6	-12	1250.58	146.322	11
4	6	-11	266.955	97.6415	19
4	-6	-9	4240.17	350.687	15
4	6	-9	4118.58	619.949	24
-4	-6	8	517.044	122.356	27
4	-6	-8	600.859	130.831	3
-4	-6	7	5238.78	376.312	27
4	6	-7	4673.21	650.367	24
4	-6	-7	5060.74	383.814	3
4	-6	-7	4675.46	367.828	15
4	6	-6	1410.12	339.658	24
-4	-6	6	894.754	178.044	22
4	-6	-6	1585.53	200.281	3
4	-6	-6	1860.69	311.959	1
4	-6	-6	1844.09	204.888	15
-4	-6	6	1880.27	205.413	27
-4	-6	5	5299.31	357.333	27
-4	-6	5	4340.85	341.851	28
4	-6	-5	4320.19	348.103	9
-4	-6	5	4156.21	328.461	10
-4	-6	5	3995.34	340.813	22
4	6	-5	4439.42	333.834	19
4	-6	-5	3883.24	336.349	4
4	-6	-5	4985.63	363.756	3
4	-6	-5	4585.57	342.609	15
4	-6	-5	4628.53	521.324	1
4	6	-5	4572.40	596.977	24

4	-6	-4	11103.1	869.073	1
4	6	-4	9982.78	915.989	24
-4	-6	4	9454.25	593.501	28
-4	-6	3	1902.31	195.120	28
4	-6	-3	2162.63	356.534	1
-4	-6	2	2745.74	246.598	28
4	-6	-2	3740.03	464.527	1
4	-6	-2	2847.02	447.725	2
4	-6	-1	654.310	225.367	1
4	-6	-1	814.119	242.821	2
4	-6	0	112.651	128.089	2
4	-6	1	9599.49	911.388	2
4	-6	2	1503.39	343.366	2
4	-6	2	1348.15	148.887	7
4	-6	2	1298.90	152.340	5
4	-6	2	1518.90	182.503	4
-4	-6	-2	1575.39	171.473	22
4	-6	3	1553.81	373.777	2
4	6	3	1131.63	144.860	18
4	-6	3	1154.54	141.521	7
4	-6	3	1070.25	144.947	5
4	-6	3	1108.05	166.994	4
-4	6	-3	1423.11	276.501	24
-4	-6	-3	1293.94	158.904	22
-4	-6	-3	1183.66	153.011	16
4	6	4	978.544	128.615	18
-4	6	-4	530.646	188.155	24
4	-6	4	1173.23	335.851	2
-4	-6	-4	936.081	142.112	22

-4	6	-5	2546.72	425.789	24
-4	-6	-5	2498.02	236.065	22
-4	-6	-5	1259.13	276.726	23
4	-6	5	2320.66	224.299	5
4	-6	5	2164.12	215.138	7
4	-6	5	2596.74	503.333	2
-4	-6	-5	2138.09	215.952	9
4	-6	6	26.1212	67.6783	7
4	-6	6	82.3522	167.364	2
-4	6	-6	61.3002	124.582	24
4	-6	6	-70.9576	76.3126	5
4	-6	7	25.8971	68.3737	7
4	-6	7	83.0036	146.088	2
-4	6	-7	129.219	131.307	24
4	-6	7	-126.750	94.9291	5
-4	-6	-8	7226.67	453.091	20
4	-6	8	8468.54	512.909	5
4	-6	8	8848.64	509.262	7
-4	-6	-9	457.595	85.5923	15
4	-6	9	505.738	99.3239	5
4	-6	10	895.456	112.335	5
4	-6	10	1245.44	152.627	4
4	-6	11	172.938	61.4604	4
4	7	-13	572.731	72.1728	19
4	-7	-13	686.600	72.7762	11
4	7	-12	1090.05	125.235	19
4	-7	-12	938.678	116.538	11
4	7	-11	1809.41	190.536	19
4	7	-10	-57.3525	68.3405	19

-4	-7	10	37.4351	62.1216	22
4	7	-9	-47.1960	71.4934	19
-4	-7	9	14.7061	66.8318	22
4	7	-8	73.2357	128.904	24
-4	-7	8	-63.4649	80.6272	22
4	7	-8	-32.4453	67.9862	19
4	-7	-8	-32.8458	86.7277	15
-4	-7	8	50.3966	72.4394	27
4	-7	-8	-101.849	96.0503	3
-4	-7	8	14.1897	57.6972	10
4	-7	-7	620.259	132.426	15
4	7	-7	871.528	296.473	24
-4	-7	7	501.193	120.500	22
4	7	-7	637.732	122.183	19
-4	-7	7	506.308	125.518	27
-4	-7	7	554.760	114.272	10
4	-7	-7	669.178	139.023	3
-4	-7	6	4560.65	359.819	22
4	7	-6	4543.26	352.808	19
-4	-7	6	4371.96	359.234	27
4	-7	-6	4165.12	349.015	15
4	-7	-6	4467.78	371.083	3
4	7	-6	4480.63	634.479	24
4	-7	-5	340.533	110.141	3
4	-7	-5	368.486	169.220	1
4	-7	-5	487.182	115.909	15
4	7	-5	132.769	135.494	24
-4	-7	5	167.132	81.2002	22
4	7	-5	240.015	90.4683	19

-4	-7	5	225.228	97.5234	27
4	-7	-5	272.201	100.097	4
4	-7	-5	269.078	90.7416	11
4	-7	-5	308.024	108.750	9
4	-7	-4	20119.0	1115.01	4
4	-7	-4	19358.6	1093.67	9
4	-7	-4	18926.7	1097.45	8
4	-7	-4	19717.3	1078.11	11
4	7	-4	20186.8	1083.41	19
-4	-7	4	20593.1	1101.34	22
4	-7	-4	19019.1	1077.39	15
4	-7	-4	24112.9	1474.41	1
-4	-7	4	21082.0	1117.77	28
4	-7	-3	25703.9	1375.76	4
4	-7	-3	24960.5	1375.38	3
-4	-7	3	27049.0	1361.33	22
-4	-7	3	27421.9	1379.91	28
4	7	-3	25525.6	1334.21	19
4	-7	-3	25712.9	1330.27	15
4	-7	-3	21017.5	1324.35	9
4	-7	-3	26085.6	1366.19	8
4	-7	-3	26100.2	1332.16	11
4	-7	-2	58.1419	70.9192	8
-4	-7	2	150.124	79.3410	28
4	-7	-2	45.9850	69.7427	4
4	-7	-2	263.693	151.613	1
4	-7	-2	62.7219	211.409	2
4	-7	-2	30.0648	73.3379	3
-4	-7	2	66.8913	68.0589	22

4	-7	-1	665.923	122.002	8
-4	-7	1	712.231	130.164	28
4	-7	-1	486.499	214.650	1
4	-7	-1	507.010	251.051	2
4	-7	-1	653.244	125.828	3
4	-7	-1	503.610	114.382	4
-4	-7	1	601.188	111.621	22
-4	-7	0	10472.6	602.066	22
4	-7	0	9380.02	551.058	7
4	-7	0	9736.95	572.410	5
4	-7	0	9028.13	601.339	8
4	-7	0	10221.0	612.648	3
4	-7	0	9801.69	615.944	4
4	-7	0	11590.1	1036.18	2
-4	-7	-1	13097.4	716.841	22
-4	-7	-1	11884.5	721.864	16
4	-7	1	11702.6	674.770	7
4	-7	1	12091.2	692.956	5
4	-7	1	11738.7	702.379	10
4	-7	1	13142.5	739.189	8
4	-7	1	11320.7	709.192	3
4	-7	1	12282.6	735.920	4
4	-7	1	12505.9	1137.40	2
-4	-7	-2	5579.04	384.507	22
4	-7	2	5566.26	406.592	8
4	-7	2	4647.31	374.681	10
-4	-7	-2	5061.98	388.087	16
4	-7	2	5584.87	365.774	7
4	-7	2	5767.64	383.675	5

4	-7	2	5157.71	382.370	3
4	-7	2	5384.64	408.829	4
4	-7	2	5658.13	745.456	2
-4	-7	-3	1965.43	203.366	22
4	-7	3	2186.58	461.164	2
4	7	3	1616.84	178.658	18
4	-7	3	2089.38	195.426	7
4	-7	3	2001.03	200.638	5
4	-7	3	1822.11	221.152	4
4	-7	3	1566.63	194.091	10
4	-7	3	1785.23	217.680	8
-4	-7	-3	1636.26	197.118	16
4	-7	4	810.011	130.032	7
4	-7	4	693.831	133.529	10
-4	-7	-4	780.785	132.829	22
4	-7	4	475.007	243.818	2
4	-7	5	4316.23	345.529	5
-4	-7	-5	4922.89	356.313	9
-4	-7	-5	4273.64	343.944	22
4	-7	5	4350.68	679.668	2
4	-7	6	3780.70	624.034	2
-4	-7	-6	3562.49	289.653	9
4	-7	6	3413.87	298.662	5
4	-7	6	3398.23	283.843	7
4	-7	7	3433.37	305.204	4
-4	-7	-7	2593.32	232.182	9
4	-7	7	2634.19	242.390	7
4	-7	7	2886.75	258.432	5
4	-7	8	1159.97	171.501	10

4	-7	8	1089.51	172.535	4
4	-7	8	1094.80	146.050	5
4	-7	9	128.222	83.5322	10
4	-7	9	66.4188	67.6208	4
4	-7	9	127.795	65.6367	5
-4	-7	-9	67.8537	48.0265	15
4	-7	10	44.6229	37.9507	5
4	-7	10	124.638	63.9905	4
4	-7	10	84.8566	60.7057	10
4	-7	10	118.878	60.3323	8
4	-7	11	130.506	39.1504	4
4	-7	11	47.6992	29.7830	8
4	8	-12	3034.45	201.728	19
4	8	-11	18.0672	49.4420	19
4	-8	-11	-52.6353	55.6769	11
4	8	-10	6729.46	432.108	19
4	-8	-10	6527.67	427.692	11
4	8	-9	3022.30	259.525	19
4	-8	-9	2793.54	254.074	11
4	-8	-8	2180.84	223.694	11
4	8	-8	1958.39	213.626	19
-4	-8	8	2120.55	229.469	27
4	-8	-8	2323.00	231.742	15
4	-8	-8	2244.88	239.774	3
-4	-8	8	1975.61	200.794	22
4	-8	-7	165.812	93.2003	11
4	8	-7	89.8459	74.5809	19
-4	-8	7	69.2977	67.5828	22
-4	-8	7	51.5558	83.7815	27

4	-8	-7	-62.7979	98.3685	15
4	-8	-7	34.3105	90.6110	3
4	-8	-6	781.303	134.628	11
-4	-8	6	1125.63	153.907	22
4	8	-6	854.208	144.934	19
4	-8	-6	1026.74	161.888	15
-4	-8	6	996.731	165.006	27
4	-8	-6	962.743	167.023	3
-4	-8	5	5138.17	394.042	22
4	8	-5	5843.41	403.222	19
4	-8	-5	5381.20	394.360	11
4	-8	-5	5413.20	401.599	15
4	-8	-5	5894.92	436.053	3
-4	-8	5	6125.43	438.293	27
-4	-8	4	1982.22	209.396	22
-4	-8	4	2212.85	237.463	27
-4	-8	4	2015.63	229.124	28
4	8	-4	1826.36	193.851	19
4	-8	-4	1708.35	191.285	15
4	-8	-4	2201.21	223.823	9
4	-8	-4	1887.26	222.973	8
4	-8	-4	1477.64	176.315	11
4	-8	-4	1846.58	224.210	4
4	-8	-3	683.513	118.006	15
4	-8	-3	776.166	135.490	9
-4	-8	3	773.898	124.146	22
-4	-8	3	847.322	149.337	28
4	-8	-3	747.500	147.987	3
4	-8	-3	738.943	137.039	8

4	-8	-3	891.552	158.970	4
-4	-8	2	27.5239	59.3299	22
-4	-8	2	68.6072	85.3824	28
4	-8	-2	-16.9195	74.9408	3
4	-8	-2	16.6575	61.0293	8
4	-8	-2	-88.0780	83.9580	4
-4	-8	1	217.199	80.3113	22
-4	-8	1	189.660	96.8472	28
4	-8	-1	346.048	104.043	3
4	-8	-1	423.269	215.445	2
4	-8	-1	400.006	110.532	8
4	-8	-1	456.406	114.960	4
4	-8	-1	206.635	73.0860	5
-4	-8	0	296.368	94.1792	22
4	-8	0	236.685	93.8540	8
4	-8	0	313.668	76.4690	7
-4	-8	0	509.015	127.104	28
4	-8	0	425.646	112.212	4
4	-8	0	274.344	89.2043	5
4	-8	0	650.944	255.081	2
4	-8	0	417.132	116.356	3
4	-8	1	7761.03	542.884	8
4	-8	1	7614.45	520.348	10
-4	-8	-1	8237.62	542.285	16
4	-8	1	8524.18	521.588	5
4	-8	1	7478.86	484.567	7
4	-8	1	7921.58	897.791	2
4	-8	1	8410.55	540.988	3
4	-8	1	8323.77	556.640	4

-4	-8	-1	8298.02	518.551	22
-4	-8	-2	54.6711	69.4420	22
-4	-8	-2	33.1373	75.2931	16
4	8	2	-13.1663	58.3170	18
4	-8	2	-35.4386	66.8329	7
4	-8	2	-17.8836	77.0983	8
4	-8	2	59.4819	79.9571	3
4	-8	2	-77.8764	158.265	2
4	-8	2	14.1628	64.3607	5
4	-8	2	92.6411	80.0003	10
-4	-8	-3	609.179	119.986	22
-4	-8	-3	575.836	129.881	16
4	8	3	726.694	114.920	18
4	-8	3	846.965	149.300	8
4	-8	3	798.995	131.832	7
4	-8	3	766.365	140.020	10
4	-8	3	480.012	271.351	2
4	-8	3	584.516	114.668	3
4	-8	3	473.081	113.572	5
4	-8	4	7943.44	548.167	8
4	-8	4	7680.44	528.676	10
4	-8	4	7785.03	498.818	7
4	-8	4	7608.64	515.635	5
-4	-8	-4	7563.65	501.921	22
4	-8	4	8280.52	980.931	2
-4	-8	-5	4767.75	379.857	9
4	-8	5	5532.65	428.625	8
4	-8	5	5810.27	422.419	10
4	-8	5	5171.50	423.067	4

4	-8	5	5823.90	395.234	7
-4	-8	-6	11816.8	708.861	9
4	-8	6	11805.7	751.413	8
4	-8	6	11915.6	743.126	10
4	-8	6	13215.2	774.836	4
4	-8	6	13078.0	737.970	5
-4	-8	-7	820.934	123.067	9
4	-8	7	1403.11	174.859	10
4	-8	7	1485.79	188.223	4
4	-8	7	1124.17	167.185	8
4	-8	7	1271.99	162.786	5
4	-8	8	51.1437	66.3097	10
4	-8	8	54.0008	80.0403	4
4	-8	8	32.3703	66.7542	5
4	-8	8	118.364	75.6508	8
4	-8	9	78.2496	51.7480	5
4	-8	9	132.247	67.1592	4
4	-8	9	136.786	69.6584	10
-4	-8	-9	87.3159	43.7246	15
4	-8	9	106.780	60.9899	8
4	-8	10	822.970	88.5408	10
4	-8	10	639.292	77.4856	8
4	-8	10	652.964	83.7934	4
4	9	-11	705.880	73.5460	19
4	-9	-11	518.740	66.3363	11
4	9	-10	1998.42	169.169	19
4	-9	-10	1955.29	163.916	11
4	9	-9	607.053	103.137	19
4	-9	-9	685.645	104.537	11

4	-9	-8	125.772	83.9753	15
4	9	-8	34.4721	59.7763	19
-4	-9	8	113.919	80.7404	27
4	-9	-8	145.360	80.7434	3
4	-9	-8	204.373	75.8433	11
4	9	-7	6745.61	448.310	19
-4	-9	7	6623.27	472.480	27
4	-9	-7	6875.61	461.136	15
4	-9	-7	7026.24	448.528	11
4	-9	-7	7158.64	479.116	3
4	-9	-6	344.984	89.1420	11
4	9	-6	188.778	77.4378	19
-4	-9	6	166.173	65.5053	22
-4	-9	6	220.999	96.7446	27
4	-9	-6	181.932	87.1291	15
4	-9	-6	335.275	110.888	3
4	-9	-5	248.311	75.3147	11
-4	-9	5	83.9982	61.3117	22
4	9	-5	183.617	73.8738	19
-4	-9	5	175.413	102.599	27
4	-9	-5	67.9126	60.5096	15
4	-9	-5	158.312	91.3706	3
-4	-9	4	4044.84	345.421	27
-4	-9	4	3890.57	343.041	28
4	-9	-4	3409.04	286.951	15
-4	-9	4	3975.97	301.700	22
4	-9	-4	3827.23	340.055	4
4	-9	-3	13698.2	836.837	9
4	-9	-3	15789.1	888.826	8

-4	-9	3	14269.3	824.714	22
4	-9	-3	15216.1	890.843	4
4	-9	-3	15281.1	887.930	3
-4	-9	3	14978.8	887.617	28
-4	-9	2	2717.65	260.687	8
4	-9	-2	3122.02	295.187	8
-4	-9	2	3040.22	294.047	28
-4	-9	2	2961.57	253.668	22
4	-9	-2	3242.04	303.233	4
4	-9	-2	2462.29	273.483	3
-4	-9	1	22858.6	1276.82	8
4	-9	-1	24701.5	1322.32	8
4	-9	-1	21278.2	1266.53	16
-4	-9	1	24823.9	1329.62	28
4	-9	-1	24632.1	1315.51	3
4	-9	-1	26214.4	1342.45	4
-4	-9	1	24322.4	1264.62	22
-4	-9	0	9324.24	571.671	22
4	-9	0	8915.72	609.512	8
-4	-9	0	8674.43	602.102	16
4	-9	0	9233.59	574.340	5
-4	-9	0	9839.87	630.974	28
4	-9	0	10682.6	1042.16	2
4	-9	0	10166.0	638.476	4
-4	-9	0	9260.45	575.296	3
4	-9	0	9327.64	610.733	3
-4	-9	-1	5665.91	396.292	22
4	-9	1	6373.31	791.831	2
-4	-9	-1	6469.35	450.413	16

4	-9	1	5404.82	406.324	5
4	-9	1	5704.64	424.629	3
-4	-9	-1	5532.15	389.864	3
-4	-9	-1	5722.17	447.235	28
4	-9	1	6346.13	453.868	8
4	-9	2	573.382	121.230	10
-4	-9	-2	576.876	112.296	22
-4	-9	-2	526.344	126.653	16
4	9	2	491.077	102.581	18
4	-9	2	640.263	142.330	8
4	-9	2	338.505	106.293	5
-4	-9	-2	464.465	127.452	28
4	-9	2	538.587	260.384	2
4	-9	2	498.101	111.017	3
-4	-9	-3	356.691	94.2899	22
-4	-9	-3	195.765	96.1549	16
4	-9	3	382.465	117.801	8
4	-9	3	294.011	92.0649	3
4	-9	3	222.120	90.7451	10
4	-9	3	286.075	100.043	5
4	-9	4	109.670	98.5923	4
4	-9	4	148.845	83.2111	5
-4	-9	-4	111.466	70.5066	22
4	-9	4	221.387	92.6384	10
-4	-9	-4	175.873	85.9094	9
4	-9	4	219.203	70.8709	3
4	-9	4	177.107	98.8812	8
4	-9	5	666.991	134.078	8
4	-9	5	480.730	127.568	4

-4	-9	-5	759.245	127.061	9
4	-9	5	456.015	116.765	10
4	-9	6	538.472	121.652	8
4	-9	6	401.119	112.981	4
4	-9	6	503.329	111.101	5
-4	-9	-6	500.479	95.6811	9
4	-9	6	375.430	110.704	10
4	-9	7	2252.53	204.571	5
4	-9	7	2105.53	207.489	10
4	-9	7	2067.70	208.641	8
4	-9	7	2356.78	220.857	4
4	-9	8	1775.58	156.602	5
-4	-9	-8	1396.67	134.183	15
4	-9	8	1553.85	158.142	8
4	-9	8	1735.77	161.721	10
4	-9	8	1686.01	166.063	4
4	-9	9	-27.6119	35.9422	8
4	-9	9	-11.5440	36.6386	10
-4	-9	-9	-15.2629	22.1700	15
4	-9	9	-31.6249	38.0335	4
-4	-10	10	-26.3219	27.8662	3
4	10	-9	921.762	85.3044	19
4	10	-8	504.738	74.1124	19
4	-10	-7	3738.78	284.317	3
4	10	-7	3128.37	231.029	19
-4	-10	7	3436.23	280.095	27
-4	-10	6	14.8012	65.6105	27
4	-10	-6	74.3996	61.1506	15
4	-10	-6	84.7260	78.6356	3

-4	-10	5	221.425	88.2012	27
4	-10	-5	42.0004	69.3695	15
4	-10	-5	154.918	87.8222	3
4	-10	-3	3385.69	315.610	4
-4	-10	3	3727.51	320.830	28
-4	-10	3	3087.52	269.502	8
-4	-10	2	335.826	94.5425	8
-4	-10	2	303.058	74.1561	22
4	-10	-2	474.849	120.920	8
4	-10	-2	528.690	129.768	4
4	-10	-2	407.068	119.702	3
-4	-10	2	521.782	124.815	27
-4	-10	2	585.545	132.654	28
4	-10	-1	2734.15	264.234	8
-4	-10	1	2166.83	251.004	16
-4	-10	1	2291.41	201.167	22
-4	-10	1	2247.63	245.601	28
-4	-10	1	2094.36	218.867	3
4	-10	-1	2489.77	252.967	3
-4	-10	0	186.609	97.9005	16
4	-10	0	120.930	101.216	8
-4	-10	0	186.182	66.2104	22
-4	-10	0	193.413	96.6068	28
-4	-10	0	188.840	76.1790	3
4	-10	0	225.506	98.5888	3
4	-10	1	921.361	151.631	3
-4	-10	-1	872.507	122.350	3
-4	-10	-1	722.413	142.968	16
4	10	1	793.972	123.799	18

4	-10	1	874.992	155.827	8
-4	-10	-1	1056.40	173.123	28
-4	-10	-1	1122.91	134.077	22
4	-10	2	2015.74	224.878	8
4	10	2	1653.23	163.843	18
4	-10	2	1871.71	198.588	3
-4	-10	-2	1559.83	165.035	22
-4	-10	-2	1742.38	206.304	16
4	-10	3	79.8543	60.4914	3
-4	-10	-3	153.641	93.2350	9
4	-10	3	84.4563	95.7268	4
4	-10	3	134.492	83.3152	5
4	-10	3	193.164	94.1280	8
-4	-10	-4	237.745	89.0354	9
4	-10	4	104.672	88.2384	8
4	-10	4	469.796	115.474	4
4	-10	4	399.415	100.543	5
-4	-10	-5	6281.91	424.185	28
4	-10	5	6659.78	443.255	4
-4	-10	-5	5677.21	394.394	9
4	-10	5	7013.97	440.720	8
-4	-10	-6	15.8566	46.9754	9
4	-10	6	23.4655	63.0854	4
4	-10	6	-11.0656	57.3343	8
-4	-10	-6	-10.6142	49.4253	28
-4	-10	-7	2375.85	177.658	15
4	-10	7	2335.05	189.053	8
4	-10	7	2330.67	196.052	4
-4	-10	-8	106.682	34.5770	15

4	-10	8	196.278	40.8212	8
4	-10	8	269.391	50.9082	4
-4	-11	7	234.184	68.8758	3
4	-11	-6	10.2042	60.4617	3
4	-11	-5	3680.70	287.023	3
-4	-11	4	2740.52	238.156	3
-4	-11	4	2644.44	243.797	27
-4	-11	4	2760.77	257.595	16
-4	-11	3	1177.35	157.128	27
-4	-11	3	993.067	141.830	3
-4	-11	3	1281.59	172.002	16
4	-11	-2	3668.59	300.788	3
-4	-11	2	3397.55	274.307	3
-4	-11	2	3767.18	305.411	16
-4	-11	2	3606.67	293.288	27
-4	-11	2	3423.25	299.165	28
-4	-11	1	7960.10	517.349	16
-4	-11	1	7402.71	499.966	27
-4	-11	1	7505.73	514.301	28
4	-11	-1	8402.74	518.866	3
-4	-11	1	7960.49	484.259	3
4	-11	-1	7844.45	512.345	8
-4	-11	0	1459.88	182.420	16
4	11	0	1559.38	152.694	18
-4	-11	0	1319.67	169.284	27
4	-11	0	1761.94	195.795	8
-4	-11	0	1360.17	151.046	3
4	-11	0	1533.44	178.171	3
4	-11	1	196.026	89.0555	8

-4	-11	-1	96.9969	74.1653	27
-4	-11	-1	161.066	84.2890	16
4	11	1	113.137	48.8438	18
4	-11	1	120.414	80.4596	3
4	-11	2	479.467	109.315	8
4	-11	2	357.754	110.688	4
4	-11	2	562.501	100.115	3
-4	-11	-2	325.353	96.2127	9
-4	-11	-3	1172.17	148.943	28
4	-11	3	1209.62	159.852	4
4	-11	3	1271.78	155.041	8
-4	-11	-3	972.651	136.241	9
-4	-11	-4	731.384	107.699	28
4	-11	4	648.968	115.031	4
4	-11	4	641.579	106.207	8
-4	-11	-4	547.195	93.7636	9
-4	-11	-5	789.335	101.264	28
4	-11	5	994.327	121.039	4
4	-11	5	891.686	108.064	8
4	-11	6	1962.50	157.429	4
4	-11	6	1745.55	142.739	8
-4	-11	-6	2007.14	145.045	15
-4	-12	6	399.995	57.3553	3
-4	-12	6	128.763	45.0309	16
4	-12	-5	121.979	55.1045	3
-4	-12	5	53.5054	45.1521	3
-4	-12	5	15.3805	43.5828	16
-4	-12	4	102.424	50.8435	27
-4	-12	4	215.287	66.5656	16

-4	-12	4	89.5649	51.1461	3
4	-12	-4	76.3800	56.1331	3
-4	-12	3	1244.02	136.690	16
-4	-12	3	1045.43	123.199	27
-4	-12	3	1029.76	115.511	3
-4	-12	2	523.231	91.1709	27
4	-12	-2	653.288	102.291	3
-4	-12	2	607.488	86.1493	3
-4	-12	2	672.363	106.038	16
4	12	-2	512.831	76.9452	18
-4	-12	1	8.85836	44.1125	27
4	12	-1	38.1302	35.0262	18
-4	-12	1	10.1591	53.6523	16
4	-12	-1	113.037	65.6308	3
-4	-12	1	-6.68754	42.4535	3
-4	-12	0	1717.25	166.086	16
4	-12	0	1790.62	176.807	4
4	-12	0	1871.53	161.140	3
-4	-12	0	1323.24	119.276	3
-4	-12	0	1567.82	145.070	27
4	-12	1	532.368	97.3399	4
4	-12	1	591.016	93.5663	8
-4	-12	-1	341.105	82.4210	9
-4	-12	-1	436.401	85.2749	28
4	-12	2	313.727	78.0611	4
-4	-12	-2	285.149	66.7421	28
4	-12	2	336.389	71.2720	8
-4	-12	-2	314.655	71.2053	9
4	-12	3	16.2968	49.6795	4

-4	-12	-3	19.5593	41.9003	9
4	-12	3	-13.6500	36.0364	8
-4	-12	-4	192.799	41.6348	15
4	-12	4	189.718	42.5870	8
4	-13	-2	65.0174	28.8290	3
5	0	-15	2337.64	167.864	20
-5	0	14	-35.3225	52.3294	29
5	0	-14	9.79538	58.8919	20
-5	0	13	-49.0450	69.3747	29
5	0	-13	65.6911	81.2108	20
-5	0	12	15.3705	79.6421	29
5	0	-12	15.5447	77.3848	20
5	0	-11	77.3856	136.360	17
5	0	-11	217.843	95.0371	20
-5	0	11	57.7082	131.289	30
5	0	-10	12.1800	52.5202	19
5	0	-10	16.8151	70.4571	20
5	0	-10	154.384	156.878	17
5	0	-9	31568.5	1660.80	19
5	0	-9	32538.2	1704.85	20
5	0	-8	-62.9611	143.058	17
5	0	-8	13.7340	63.9573	20
5	0	-8	69.2661	86.2053	24
5	0	-7	44904.5	2514.12	24
5	0	-7	49550.3	2766.52	17
5	0	-6	57.1013	82.0933	24
5	0	-6	46.5351	94.6038	17
5	0	-5	17926.9	1236.63	17
5	0	-4	72.0909	115.874	17

5	0	-3	38917.8	1948.06	26
5	0	-3	40183.9	2172.16	25
5	0	-3	44376.2	2246.61	17
5	0	-2	-27.2299	87.4973	25
5	0	-2	30.3418	97.4967	17
5	0	-1	53554.3	2851.56	25
5	0	-1	58152.9	2888.46	17
5	0	-1	52359.1	2702.13	2
5	0	0	-79.0917	110.458	1
5	0	0	17.1082	57.6636	2
5	0	0	30.5106	116.005	25
5	0	1	3819.28	361.707	1
5	0	1	3895.58	355.893	2
5	0	1	3739.51	423.010	25
5	0	2	-20.1974	58.0483	1
5	0	2	52.6183	75.6138	2
5	0	2	-37.6167	108.112	25
-5	0	-3	11911.1	965.664	23
-5	0	-4	-44.3041	135.072	23
-5	0	-5	23873.2	1595.96	23
5	0	6	72.4088	61.3170	18
5	0	6	-18.7412	45.6689	7
5	0	6	40.7044	48.5040	6
-5	0	-6	-56.4868	140.597	23
-5	0	-7	921.354	298.317	23
5	0	7	1293.38	157.129	18
-5	0	-8	-125.778	169.101	23
5	0	8	-36.6430	66.9095	18
5	0	10	15.8129	44.0103	5

-5	0	-11	356.326	80.2289	20
5	0	11	371.565	91.8016	29
-5	0	-12	-24.7287	43.0683	20
5	0	12	8.61111	43.7518	29
5	1	-15	2463.66	170.264	20
-5	-1	14	1111.97	123.483	29
5	1	-14	1530.30	151.927	20
5	1	-13	179.110	85.1247	20
-5	1	13	317.900	92.9411	29
-5	-1	13	288.353	84.5802	29
-5	1	12	107.521	92.4849	29
-5	-1	12	205.134	91.9289	29
5	1	-12	61.0942	80.8126	20
5	1	-12	100.713	58.2145	19
5	1	-11	131.930	72.1990	19
5	1	-11	16.4847	78.5735	20
5	1	-11	-73.9849	168.106	17
5	-1	-11	-78.9833	160.518	17
5	-1	-11	-12.8647	62.6997	11
-5	1	11	51.9714	96.4218	29
-5	-1	11	101.254	99.9928	29
-5	1	11	115.634	131.371	30
-5	-1	11	56.6708	115.174	30
5	1	-10	223.179	83.9881	19
5	1	-10	115.495	80.5454	20
5	1	-10	-73.4971	167.063	17
5	-1	-10	318.688	229.031	17
5	-1	-10	95.8850	66.9224	11
-5	1	10	233.776	197.021	30

-5	-1	10	229.535	154.346	30
5	1	-9	8493.96	555.384	20
5	-1	-9	8248.90	553.537	20
5	1	-9	9098.00	931.775	17
5	-1	-9	9075.12	972.829	17
5	1	-9	8274.19	776.509	24
5	-1	-9	8008.73	527.848	11
-5	-1	9	8954.98	850.124	30
-5	1	9	10105.7	890.576	30
5	-1	-8	34043.1	1816.18	20
5	1	-8	34547.8	1817.34	20
5	1	-8	40808.5	2345.93	17
5	-1	-8	41358.8	2383.90	17
-5	-1	8	40988.2	2207.48	30
-5	1	8	35973.5	2171.90	30
5	-1	-8	34430.4	1804.34	11
5	1	-8	36020.0	2114.15	24
-5	-1	7	16831.0	1165.19	30
-5	1	7	16659.3	1173.53	30
5	1	-7	18464.8	1282.03	17
5	-1	-7	17615.0	1292.16	17
5	1	-7	13910.5	1089.66	24
-5	-1	6	5796.93	568.956	30
5	1	-6	4761.12	565.251	17
5	1	-6	4930.98	506.311	24
-5	1	5	24825.5	1458.96	30
5	1	-5	20358.1	1340.38	24
5	1	-5	22333.3	1430.74	17
5	1	-4	60870.7	3258.84	17

5	-1	-3	8596.44	488.845	26
5	1	-3	9443.86	684.223	25
5	1	-3	8857.30	491.401	26
5	1	-3	9751.93	725.425	17
5	-1	-2	50744.7	2699.61	2
5	1	-2	51681.9	2758.62	25
5	1	-2	55629.3	2814.65	17
5	-1	-1	26102.2	1485.10	2
5	-1	-1	27735.4	1587.21	1
5	1	-1	25564.4	1590.99	17
5	1	-1	27494.8	1559.23	25
5	1	0	256128.	11510.6	29
5	1	0	248280.	11766.3	25
5	-1	0	235650.	11739.1	25
5	-1	0	247685.	11721.9	1
5	1	0	226716.	11503.8	2
5	-1	0	248937.	11679.0	2
5	-1	1	26152.5	1282.05	29
5	1	1	25862.4	1284.10	29
5	1	1	26437.1	1543.02	25
5	-1	1	24221.4	1505.45	25
5	1	1	23272.5	1393.62	1
5	1	1	23680.8	1325.71	2
5	-1	1	24562.7	1450.25	2
5	-1	1	24792.6	1436.89	1
5	-1	2	373.705	81.3744	29
5	1	2	370.139	82.7707	29
5	1	2	343.251	151.097	25
5	-1	2	335.929	152.702	25

5	-1	2	287.221	126.791	2
5	-1	2	543.189	132.585	1
5	1	2	344.161	92.8528	1
-5	1	-3	1076.68	271.080	23
5	1	3	1127.78	265.745	25
5	-1	3	1233.16	264.268	25
5	-1	3	972.814	133.491	29
5	1	3	1145.08	142.019	29
5	-1	3	1152.17	183.624	1
5	1	4	71756.4	3514.86	29
5	-1	4	70360.9	3510.65	29
5	-1	4	70211.6	3878.40	25
5	1	4	68621.5	3894.36	25
-5	-1	-4	69600.2	3835.40	23
-5	1	-4	82931.0	3900.66	23
5	1	5	69103.0	3483.76	29
5	-1	5	72809.7	3477.79	29
-5	1	-5	75613.7	3863.16	23
-5	-1	-5	75868.1	3887.59	23
5	1	5	67434.7	3903.36	25
5	-1	5	73260.4	3927.25	25
5	-1	5	67266.5	3431.52	18
-5	1	-6	55.4176	138.146	23
-5	-1	-6	171.893	142.818	23
5	1	6	191.458	75.3427	18
5	-1	6	195.682	73.9178	18
5	1	6	136.137	88.5006	29
5	-1	6	197.780	87.3576	29
5	1	6	-65.4209	199.575	25

5	-1	6	190.487	204.173	25
5	-1	6	141.908	66.9897	6
5	-1	6	160.785	61.7416	7
5	1	6	217.126	64.0870	6
5	1	7	3426.02	300.666	29
5	-1	7	2612.17	267.556	29
5	-1	7	2704.98	221.758	7
-5	1	-7	2757.71	466.839	23
-5	-1	-7	3111.88	503.507	23
5	1	7	2777.66	242.747	18
5	-1	7	2790.84	245.235	18
5	1	7	2577.97	487.952	25
5	-1	7	2999.21	521.356	25
5	1	8	23.4382	58.3900	18
-5	1	-8	-122.485	215.586	23
-5	-1	-8	-127.266	233.146	23
5	1	8	-71.5195	251.759	25
5	-1	8	-70.2124	257.249	25
5	1	8	-55.4157	101.109	29
5	-1	8	-18.2122	86.8360	29
5	1	9	463.679	126.975	29
5	-1	9	668.343	137.455	29
5	-1	10	3609.64	306.587	29
5	1	10	4337.42	329.589	29
-5	1	-10	3757.16	274.229	20
5	-1	10	3285.96	248.106	5
5	1	11	4700.16	329.843	29
5	-1	11	3524.01	303.151	29
-5	1	-11	4424.87	289.476	20

-5	-1	-11	4427.34	290.892	20
5	1	12	25.4310	48.7361	29
-5	1	-12	5.75252	36.9816	20
-5	-1	-12	36.5350	40.1105	20
5	2	-15	209.615	40.0404	20
5	-2	-15	240.298	45.1832	20
-5	2	14	106.657	54.9985	29
-5	-2	14	76.8162	42.9995	29
5	-2	-14	188.743	63.1513	20
5	2	-14	210.046	62.4441	20
-5	2	13	-11.4477	58.1642	29
-5	-2	13	20.5368	51.1190	29
5	-2	-13	34.5224	46.4088	11
5	2	-13	23.8055	70.5261	20
5	-2	-13	-24.0414	61.0754	20
5	2	-12	20789.2	1188.01	19
5	2	-12	21336.7	1207.82	20
5	-2	-12	22569.3	1216.61	20
-5	-2	12	24985.0	1221.01	29
5	-2	-12	20147.9	1179.87	11
5	2	-11	8118.20	538.947	19
-5	2	11	10061.0	592.618	29
-5	-2	11	9274.75	566.949	29
5	-2	-11	7701.90	526.488	11
5	2	-11	7986.51	542.866	20
5	-2	-11	8550.02	552.013	20
5	-2	-10	1220.94	174.538	20
5	2	-10	1151.74	168.769	20
-5	2	10	1493.26	340.646	30

-5	-2	10	1103.09	292.083	30
5	2	-10	894.265	294.462	17
5	2	-10	1279.40	305.129	24
5	-2	-10	1692.97	189.030	11
-5	-2	9	2897.16	448.307	30
-5	2	9	3346.36	481.664	30
5	2	-9	2715.10	478.042	17
5	2	-9	2555.24	433.599	24
5	-2	-9	2317.53	394.809	1
5	2	-9	2135.87	231.642	20
5	-2	-9	2312.48	237.927	20
5	2	-8	22180.7	1429.77	24
5	2	-8	20508.6	1465.87	17
5	-2	-8	21312.6	1526.67	17
5	-2	-8	19948.3	1398.35	1
-5	2	8	21022.7	1391.37	30
-5	-2	8	18548.5	1339.80	30
5	-2	-8	18381.1	1039.97	20
5	2	-8	17955.8	1036.63	20
5	2	-7	10735.8	930.563	17
5	-2	-7	12855.2	1049.47	17
5	2	-7	10174.6	866.258	24
-5	-2	7	10196.5	870.953	30
-5	2	7	9723.05	848.081	30
5	2	-6	670.232	211.185	17
5	-2	-6	938.056	253.435	17
-5	2	6	786.899	225.519	30
5	2	-6	736.081	214.947	24
-5	2	5	21652.8	1256.99	30

5	2	-5	18638.9	1206.32	17
5	-2	-5	19437.4	1243.09	17
5	2	-5	18505.6	1182.55	24
5	2	-4	200465.	9483.25	17
5	-2	-4	191333.	9511.32	17
5	2	-4	187143.	9458.23	24
5	2	-3	181299.	8175.90	17
5	-2	-3	189514.	8219.59	17
5	2	-3	166683.	8103.77	25
5	-2	-3	168163.	8096.19	25
5	2	-3	161892.	7864.10	26
5	-2	-3	161473.	7862.06	26
5	-2	-3	164890.	8212.26	1
5	2	-2	5054.23	476.592	17
5	-2	-2	5160.19	482.795	17
5	2	-2	4841.39	449.974	25
5	-2	-2	4429.31	281.579	26
5	2	-2	4292.93	282.343	26
5	-2	-2	4630.19	438.641	25
5	-2	-2	4155.80	430.752	2
5	-2	-2	4906.72	485.893	1
5	2	-1	15928.9	1084.89	17
5	-2	-1	14699.2	1099.77	17
5	-2	-1	17291.1	1082.66	1
5	-2	-1	16559.1	1052.82	2
5	2	-1	15607.2	840.872	26
5	2	-1	17336.5	1069.43	25
5	-2	-1	17949.0	1057.16	25
5	-2	0	91177.8	4449.89	1

5	-2	0	89444.2	4438.31	2
5	2	0	91617.9	4467.16	25
5	-2	0	89237.6	4435.66	25
5	-2	0	84390.7	4182.05	26
5	2	1	1043.26	232.031	25
5	-2	1	1026.04	223.942	25
5	-2	1	1169.42	214.211	1
5	-2	1	724.952	190.470	2
5	-2	1	1232.19	133.351	29
5	2	1	1123.38	132.978	29
-5	-2	-2	-130.173	146.238	23
-5	2	-2	42.7575	137.397	23
5	-2	2	-38.0512	102.304	2
5	-2	2	140.220	102.752	1
5	2	2	77.9674	112.047	25
5	-2	2	37.3106	119.895	25
5	-2	2	50.7179	51.5493	29
5	2	2	53.0532	60.0419	29
5	-2	3	3028.41	240.309	29
5	2	3	2858.19	242.708	29
5	-2	3	2711.75	328.483	1
5	-2	3	2969.16	418.163	2
5	-2	3	2931.74	402.805	25
5	2	3	2783.53	404.780	25
-5	-2	-3	3647.11	452.125	23
-5	2	-3	2370.89	427.865	23
5	2	4	97410.2	4764.20	29
5	-2	4	98734.4	4759.56	29
5	-2	4	95464.7	5157.20	2

-5	-2	-4	106165.	5149.84	23
-5	2	-4	98238.5	5080.72	23
5	2	4	97055.9	5152.42	25
5	-2	4	94368.8	5122.01	25
5	-2	5	27898.6	1448.29	14
5	2	5	30114.3	1510.70	29
5	-2	5	30311.9	1494.62	29
5	-2	5	28232.7	1444.24	6
5	-2	5	28015.9	1440.47	7
5	-2	5	26468.0	1448.16	18
5	2	5	27437.5	1451.69	18
-5	2	-5	27168.2	1784.78	23
-5	-2	-5	31036.3	1873.01	23
-5	-2	-5	27725.1	1454.86	22
5	2	5	30426.3	1929.69	25
5	-2	5	27881.3	1858.34	25
5	2	6	35388.6	1762.81	29
5	-2	6	36118.7	1757.29	29
5	-2	6	33714.1	1702.12	6
5	-2	6	32260.9	1688.16	7
5	-2	6	33713.0	1712.97	18
5	2	6	32585.6	1703.41	18
-5	2	-6	27404.5	2020.67	23
-5	-2	-6	33403.2	2132.94	23
5	2	6	32493.8	2182.31	25
5	-2	6	33973.6	2185.25	25
5	2	7	5001.83	381.813	29
5	-2	7	4447.19	363.925	29
5	-2	7	4407.86	312.634	7

5	2	7	4722.07	330.225	18
5	2	7	4292.78	639.888	25
5	-2	7	4315.84	620.135	25
5	2	8	13741.4	756.637	29
5	-2	8	13656.9	747.983	29
5	2	8	12555.4	673.592	18
5	-2	8	11596.5	653.844	7
5	2	8	12108.4	1120.03	25
5	-2	8	12324.1	1115.56	25
5	2	9	1047.34	128.746	18
5	-2	9	1407.72	153.954	5
5	-2	9	1131.03	124.402	7
5	2	9	1104.95	172.471	29
5	-2	9	1172.36	177.069	29
5	2	10	2216.88	224.933	29
5	-2	10	1776.03	205.270	29
5	-2	10	2237.38	182.439	5
-5	2	-10	2219.29	193.222	20
5	2	11	82.3723	58.6396	29
5	-2	11	10.9812	60.1489	29
-5	2	-11	69.4273	47.7553	20
-5	2	-12	1649.72	126.391	20
-5	-2	-12	1812.11	136.268	20
-5	-3	15	742.966	70.7639	22
5	3	-14	332.904	65.2243	20
5	-3	-14	370.499	70.2189	20
5	-3	-14	245.165	46.0362	11
-5	3	13	214.225	67.8307	29
-5	-3	13	164.657	56.2800	29

5	3	-13	234.233	71.4421	19
5	-3	-13	209.392	71.9798	20
5	3	-13	215.501	72.8513	20
5	-3	-13	184.979	65.7856	11
-5	3	12	295.090	95.0161	29
-5	-3	12	215.409	75.4014	29
5	3	-12	261.300	93.5275	19
5	-3	-12	241.438	84.3436	11
5	3	-12	222.677	82.7237	20
5	-3	-12	145.579	79.1211	20
5	3	-11	4419.10	367.710	19
-5	-3	11	5389.20	376.282	29
-5	3	11	5887.92	398.338	29
5	-3	-11	4949.01	375.188	11
5	3	-11	4865.09	372.486	20
5	-3	-11	4368.91	361.375	20
5	3	-10	1134.73	307.443	17
5	-3	-10	1478.46	408.408	17
5	3	-10	1926.46	384.443	24
-5	-3	10	744.332	171.068	29
-5	3	10	2047.65	378.669	30
-5	-3	10	1382.84	301.524	30
5	3	-10	1857.32	199.760	20
5	-3	-10	1565.62	185.784	20
5	3	-9	363.058	194.852	17
5	-3	-9	444.980	226.438	17
5	3	-9	186.677	155.404	24
5	-3	-9	313.064	95.4560	20
5	3	-9	307.172	92.5053	20

-5	3	9	495.200	216.912	30
-5	-3	9	51.5198	139.093	30
5	3	-8	9589.18	920.179	24
5	3	-8	9515.82	903.037	17
5	-3	-8	11275.1	1052.12	17
5	-3	-8	10090.1	862.576	1
-5	-3	8	10226.1	877.371	30
-5	3	8	10011.7	882.095	30
5	3	-8	9356.15	584.389	20
5	-3	-8	9053.28	583.924	20
-5	-3	7	13947.4	1041.98	30
-5	3	7	13780.9	1052.29	30
5	3	-7	14781.9	1128.83	17
5	-3	-7	15904.8	1206.89	17
5	-3	-7	14781.5	1094.08	1
-5	-3	7	13533.9	762.195	27
5	3	-7	13682.3	1110.39	24
-5	-3	6	34640.6	2326.77	30
-5	3	6	40679.8	2324.84	30
5	3	-6	44309.6	2415.45	17
5	-3	-6	41943.2	2451.66	17
5	-3	-6	44804.1	2431.11	1
-5	-3	6	40173.9	2019.30	27
5	3	-6	43407.3	2426.14	24
5	3	-5	9048.87	766.439	17
5	-3	-5	9460.60	800.193	17
5	-3	-5	8674.17	770.548	1
5	3	-5	10116.3	818.699	24
-5	3	5	7730.80	727.748	30

5	3	-4	20898.5	1242.51	17
5	-3	-4	20506.5	1264.37	17
-5	-3	4	16849.2	934.092	28
5	3	-4	18999.6	1261.11	24
5	-3	-4	20952.7	1293.12	1
5	3	-3	14680.5	992.403	17
5	-3	-3	15701.8	1024.50	17
5	3	-3	14841.6	952.559	25
5	3	-3	13776.5	746.021	26
5	3	-2	375.704	152.805	17
5	-3	-2	161.572	123.148	17
5	3	-2	195.902	102.877	25
5	3	-2	175.576	51.6542	26
5	-3	-2	159.330	46.0288	26
5	-3	-2	186.367	101.573	25
5	-3	-2	182.355	133.884	1
5	-3	-2	211.165	147.690	2
5	-3	-1	12368.5	848.060	17
5	-3	-1	11969.9	875.544	2
5	-3	-1	12761.8	890.303	1
5	3	-1	11405.9	619.442	26
5	3	-1	12306.6	833.271	25
5	-3	-1	11678.0	800.883	25
5	-3	-1	10843.3	610.505	26
5	-3	0	32748.3	1911.05	2
5	-3	0	34427.2	1899.00	1
5	3	0	35611.9	1884.03	25
5	-3	0	32722.2	1840.25	25
5	-3	0	31878.8	1608.32	26

5	3	0	31894.2	1616.38	26
5	3	1	6516.07	384.794	29
5	-3	1	6396.29	601.598	2
5	-3	1	6225.35	563.301	1
5	3	1	6321.31	573.890	25
5	-3	1	4923.06	510.989	25
5	-3	1	5460.75	357.299	26
5	-3	2	18658.0	1175.96	1
5	-3	2	16360.3	1229.14	2
-5	-3	-2	19279.7	1276.08	23
-5	3	-2	19066.8	1269.13	23
5	-3	2	16860.6	1181.79	25
5	3	2	18675.3	1225.93	25
5	-3	2	17048.8	942.980	29
5	3	2	18264.5	958.571	29
5	-3	3	1379.74	301.957	2
5	-3	3	1160.26	244.503	1
-5	-3	-3	1698.30	312.985	23
-5	3	-3	1092.13	263.252	23
5	3	3	1354.22	283.970	25
5	-3	3	2187.11	335.655	25
5	3	3	1645.63	172.042	29
5	-3	3	1485.67	161.695	29
5	-3	4	24947.3	1712.53	2
5	-3	4	24293.8	1306.61	29
5	3	4	25421.5	1320.77	29
-5	-3	-4	27774.7	1669.26	23
-5	3	-4	25064.6	1584.38	23
5	3	4	24523.7	1659.12	25

5	-3	4	23013.7	1613.55	25
5	-3	5	29312.9	1933.31	2
5	3	5	26920.0	1407.31	18
5	-3	5	30245.0	1449.44	29
5	3	5	27268.6	1453.39	29
5	-3	5	26191.0	1398.65	7
5	-3	5	26214.7	1401.12	6
-5	3	-5	24169.5	1694.08	23
-5	-3	-5	31880.1	1858.75	23
-5	-3	-5	26451.2	1416.77	22
5	3	5	29217.1	1873.81	25
5	-3	5	27268.5	1810.56	25
5	-3	6	22890.9	1704.72	2
5	3	6	22370.7	1165.13	18
5	-3	6	23863.5	1213.21	29
5	3	6	23191.5	1218.43	29
5	-3	6	21127.1	1153.46	7
-5	-3	-6	23862.7	1609.25	23
5	3	6	24017.1	1661.90	25
5	-3	6	19624.8	1550.13	25
5	-3	7	8199.93	924.042	2
5	3	7	7501.64	443.910	18
5	-3	7	6662.99	471.685	29
5	3	7	7471.36	496.963	29
5	-3	7	7601.62	445.953	7
-5	-3	-7	6915.89	783.851	23
5	3	7	7805.35	839.113	25
5	-3	7	7595.16	806.326	25
5	3	8	67.9321	267.475	25

-5	-3	-8	61.5726	177.162	23
5	3	8	245.280	108.615	29
5	-3	8	32.9418	82.4216	29
5	3	8	307.993	77.8209	18
5	-3	8	160.215	70.0010	7
5	-3	8	264.638	90.0284	5
5	-3	8	77.9864	158.711	2
5	-3	9	2456.26	186.168	7
5	-3	9	2576.80	202.471	5
5	-3	10	101.641	58.4097	5
-5	-3	-10	56.7269	56.8730	20
-5	3	-10	8.66717	48.2816	20
5	3	10	109.677	75.0479	29
5	-3	10	-12.5425	74.3430	29
5	-3	11	635.075	72.0077	5
5	3	11	174.289	80.2527	29
-5	3	-11	626.413	79.3488	20
-5	-3	-11	665.670	87.2220	20
-5	3	-11	249.094	76.3984	21
-5	-3	-12	732.809	73.9030	20
-5	-4	14	511.639	84.1102	22
5	-4	-14	601.557	67.7860	11
-5	4	13	1491.74	150.269	29
5	4	-13	1740.63	166.044	19
5	4	-13	1849.13	155.246	20
5	-4	-13	1974.18	169.574	11
-5	4	12	456.776	95.5208	29
5	4	-12	284.624	92.3749	19
5	-4	-12	288.632	81.2403	20

5	4	-12	271.540	77.2197	20
5	-4	-12	390.373	97.8289	11
5	4	-11	2727.56	263.057	19
5	-4	-11	2554.45	249.580	11
-5	4	11	3207.00	266.721	29
-5	-4	11	2184.74	216.611	29
5	4	-11	2638.57	239.516	20
5	-4	-11	2901.55	246.135	20
5	4	-10	1634.16	199.600	19
-5	4	10	1032.56	264.285	30
-5	-4	10	666.637	138.085	29
-5	4	10	1942.23	213.521	29
5	-4	-10	1685.34	412.447	17
5	4	-10	1628.18	179.968	20
5	-4	-10	1439.94	175.945	20
5	4	-10	2059.60	409.719	24
5	-4	-9	9594.91	1033.51	17
5	4	-9	10844.1	1039.31	24
-5	4	9	8465.09	868.888	30
-5	-4	9	9125.96	847.977	30
5	4	-9	10542.9	632.668	20
5	-4	-9	11288.6	642.187	20
5	4	-8	91.3366	64.9568	20
5	-4	-8	39.1977	56.3320	20
-5	4	8	-101.039	125.745	30
-5	-4	8	45.3940	103.144	30
5	-4	-8	65.9680	134.066	17
5	-4	-8	-43.7867	108.987	1
-5	-4	8	12.7001	56.2552	27

5	4	-8	64.4653	131.012	24
5	4	-7	7827.43	796.562	24
5	-4	-7	6724.92	471.218	3
5	4	-7	6749.82	439.671	20
5	-4	-7	6816.78	437.247	20
-5	-4	7	6738.37	437.144	27
5	4	-7	6086.35	670.324	17
5	-4	-7	7313.46	779.250	17
5	-4	-7	6688.77	667.066	1
-5	-4	7	6023.87	676.977	30
-5	4	7	6954.46	682.339	30
5	4	-6	54.2660	123.325	24
5	-4	-6	182.514	131.163	1
5	4	-6	66.7522	62.2163	19
5	-4	-6	81.9395	63.6408	15
5	-4	-6	312.062	158.568	17
5	4	-6	83.2089	94.5639	17
-5	-4	6	44.4326	49.2637	27
-5	-4	6	35.8445	96.3975	30
-5	4	6	-118.243	98.1339	30
5	4	-5	3057.89	472.180	24
5	4	-5	3581.38	424.954	17
5	-4	-5	4546.28	527.578	17
5	-4	-5	4062.47	487.554	1
-5	4	5	2962.96	384.450	30
-5	-4	5	3288.69	255.193	28
5	-4	-4	81.5309	101.541	17
5	4	-4	302.446	132.310	17
5	-4	-4	173.237	164.709	1

-5	-4	4	-34.6116	70.4490	30
-5	-4	4	104.548	51.2542	28
5	-4	-3	32264.7	1818.34	1
-5	-4	3	27818.2	1436.54	28
5	4	-3	25396.9	1647.84	17
5	-4	-3	32512.1	1759.21	17
5	-4	-2	3848.37	467.868	2
-5	-4	2	3414.79	248.587	28
5	4	-2	3435.86	232.669	26
5	-4	-2	3643.35	368.474	25
5	-4	-2	3511.99	228.840	26
5	4	-2	2975.37	355.878	25
5	-4	-2	3446.35	420.125	17
5	-4	-1	1472.21	226.680	25
5	4	-1	1550.39	136.928	26
5	-4	-1	1555.07	131.746	26
5	4	-1	1658.65	255.588	25
5	-4	-1	1356.97	275.173	1
5	-4	-1	1002.43	260.312	2
5	-4	0	20059.1	1158.40	25
5	4	0	21817.1	1215.43	25
5	-4	0	19438.6	938.311	26
5	4	0	19668.9	950.047	26
5	-4	0	21031.1	1327.57	2
5	-4	0	23192.2	1322.99	1
5	4	1	28292.1	1557.47	25
5	-4	1	23597.9	1462.63	25
5	-4	1	23206.7	1237.68	26
5	-4	1	27745.8	1583.66	1

5	-4	1	25606.3	1642.86	2
-5	-4	-1	27177.4	1626.73	23
-5	-4	-2	78438.5	3730.03	23
-5	4	-2	71166.0	3667.35	23
5	4	2	67671.0	3681.86	25
5	-4	2	65812.2	3620.81	25
5	-4	2	64808.5	3349.56	26
5	-4	2	68859.3	3692.58	1
5	-4	2	74332.1	3841.64	2
5	4	2	73429.5	3389.60	29
5	-4	3	232.904	157.329	2
5	4	3	455.750	185.911	25
5	-4	3	281.174	142.266	25
5	4	3	450.153	96.3820	29
5	-4	3	315.791	77.4805	29
-5	-4	-3	214.461	145.410	23
-5	4	-3	329.980	149.856	23
5	4	4	20645.1	1388.49	25
5	-4	4	18762.5	1315.96	25
-5	-4	-4	18059.4	1015.97	22
-5	4	-4	16956.5	1241.30	23
-5	-4	-4	18347.1	1323.89	23
5	-4	4	21871.9	1530.25	2
5	4	4	18772.3	1011.54	18
5	-4	4	19307.1	1023.33	29
5	4	4	20596.2	1045.40	29
5	-4	4	18156.9	1000.67	7
5	-4	4	18624.0	1004.34	6
5	-4	4	18092.7	1005.54	14

5	4	5	24.1474	56.2264	18
5	4	5	61.4156	71.4995	29
5	-4	5	54.4267	63.3641	29
5	4	5	-57.6404	130.969	25
5	-4	5	51.1699	103.993	25
-5	4	-5	132.844	119.049	23
-5	-4	-5	-53.3632	143.465	23
-5	-4	-5	95.8920	72.3336	22
5	-4	5	11.8416	53.8172	7
5	-4	5	-72.4723	127.553	2
5	-4	5	-12.8569	64.0063	5
-5	-4	-6	19768.8	1523.30	23
-5	-4	-6	23174.5	1200.64	22
5	-4	6	26188.0	1816.38	2
5	4	6	21998.7	1162.02	18
5	-4	6	18827.3	1169.33	29
5	4	6	20776.8	1198.95	29
5	-4	6	22728.0	1168.78	7
5	4	6	24153.8	1657.15	25
5	-4	6	25396.3	1613.99	25
5	-4	6	23208.1	1186.58	5
-5	-4	-7	3230.47	511.960	23
5	-4	7	2534.07	270.278	29
5	4	7	3412.32	304.486	29
5	-4	7	3817.15	285.928	7
5	4	7	3760.51	272.356	18
5	-4	7	3314.50	586.648	2
5	4	7	3944.26	569.974	25
5	-4	7	4215.50	305.766	5

5	-4	8	15797.3	628.453	7
5	4	8	7662.12	579.779	29
5	-4	8	5262.43	532.152	29
5	-4	8	16323.3	651.083	5
-5	4	-9	4502.08	238.145	20
5	4	9	1965.62	219.295	29
5	-4	9	4626.01	248.065	7
5	-4	9	5191.98	275.371	5
-5	4	-10	1009.63	99.6356	20
-5	-4	-10	992.366	109.988	20
5	-4	10	1133.14	115.859	5
5	-4	11	5393.33	314.843	5
-5	-4	-11	6324.59	336.056	20
5	-5	-14	147.714	32.6660	11
5	5	-14	148.465	35.2510	19
-5	-5	13	3628.25	266.981	22
5	-5	-13	3988.55	261.665	11
5	5	-13	3685.40	262.807	19
5	5	-12	180.910	76.9121	19
-5	5	12	164.874	58.3205	29
5	-5	-12	140.854	71.9673	11
-5	5	11	5846.89	377.818	29
5	-5	-11	5915.58	412.052	11
5	-5	-11	5176.00	361.256	20
5	-5	-10	70.6940	63.4550	20
-5	5	10	106.010	79.7419	29
5	-5	-10	49.7710	93.9306	11
5	-5	-9	3414.18	545.155	17
5	5	-9	3899.50	584.252	24

5	-5	-9	3807.91	273.522	20
5	5	-8	-33.6164	70.4243	19
-5	5	8	-92.6357	105.243	30
5	-5	-8	63.5548	111.859	17
-5	-5	8	-14.2126	59.5506	27
5	5	-8	69.7914	141.837	24
-5	-5	7	27888.3	1455.75	27
5	-5	-7	29275.4	1771.66	1
5	5	-7	26752.9	1477.42	19
-5	5	7	28348.6	1755.15	30
5	-5	-7	24429.3	1807.64	17
5	-5	-7	27658.9	1483.76	15
5	-5	-7	29014.1	1497.93	3
5	5	-7	28562.6	1944.69	24
5	-5	-6	17772.8	1249.63	1
5	5	-6	16816.3	950.940	19
-5	5	6	16396.5	1187.99	30
5	-5	-6	16445.4	1272.68	17
5	-5	-6	16829.1	954.893	15
-5	-5	6	17110.4	934.863	27
5	-5	-6	16854.2	952.835	11
5	-5	-5	3125.86	269.320	3
5	-5	-5	3443.12	268.405	11
-5	-5	5	2377.92	262.795	22
5	5	-5	3078.21	259.183	19
5	-5	-5	3136.57	449.191	17
5	-5	-5	3058.39	259.135	15
5	-5	-5	3530.01	463.254	1
-5	5	5	2012.15	369.512	30

-5	-5	5	3383.55	256.540	28
5	-5	-4	39.9904	107.647	17
5	-5	-4	282.636	165.900	1
-5	5	4	30.8351	121.471	30
-5	-5	4	139.281	62.6419	28
5	-5	-3	7722.51	573.093	17
5	-5	-3	8395.79	697.237	1
-5	-5	3	7467.46	341.710	28
5	-5	-2	2088.83	379.855	2
-5	-5	2	1415.47	155.874	28
5	5	-2	2058.61	262.592	25
5	-5	-2	1058.29	229.596	17
5	-5	-1	1902.95	337.130	1
5	-5	-1	2171.47	385.273	2
5	5	-1	1716.33	256.744	25
-5	-5	0	316.502	135.224	23
5	-5	0	539.527	190.985	2
5	-5	0	501.655	203.002	1
5	-5	0	617.380	141.169	25
5	5	0	301.266	127.827	25
-5	-5	-1	77162.2	3834.97	23
5	-5	1	82831.8	4001.73	1
5	-5	1	73954.6	4054.01	2
5	-5	1	68499.8	3706.93	25
5	5	1	68273.9	3775.12	25
5	-5	2	20882.9	1408.97	1
5	-5	2	20522.7	1505.24	2
5	5	2	21018.2	1327.20	25
5	-5	2	19584.5	1237.65	25

5	-5	2	18989.2	1042.27	7
-5	-5	-2	20980.5	1323.19	23
5	-5	3	3396.42	277.200	14
5	5	3	4436.63	515.260	25
5	-5	3	3407.94	414.686	25
-5	-5	-3	3655.57	297.683	22
-5	-5	-3	3351.53	448.813	23
5	-5	3	3853.17	289.879	5
5	5	3	3664.78	291.468	18
5	-5	3	3941.17	285.195	6
5	-5	3	4176.03	292.244	7
5	-5	3	5136.19	655.916	2
5	5	3	3853.56	295.708	29
5	5	4	12.5180	52.4541	18
5	5	4	27.2976	57.1909	29
5	5	4	100.037	134.474	25
-5	-5	-4	-47.7309	118.806	23
-5	-5	-4	55.0397	59.3275	22
5	-5	4	144.174	163.794	2
5	-5	4	12.1059	53.6277	14
-5	-5	-5	9836.44	1036.74	23
-5	-5	-5	17016.7	865.553	22
5	-5	5	16954.3	1405.67	2
5	5	5	16023.7	834.333	18
5	5	5	11019.9	810.212	29
5	-5	5	16747.6	841.437	7
5	5	5	14129.6	1163.39	25
5	-5	5	15688.5	845.129	5
5	-5	6	1100.38	153.601	5

-5	-5	-6	755.927	243.626	23
-5	-5	-6	1262.98	166.838	22
5	5	6	1197.39	144.911	18
5	-5	6	1463.59	398.955	2
5	5	6	491.991	119.843	29
5	-5	6	1314.21	155.497	7
5	-5	7	8131.32	888.608	2
5	-5	7	7069.36	370.845	7
5	5	7	1917.97	273.926	29
-5	-5	-7	3948.86	538.741	23
5	-5	7	7564.23	392.147	5
5	5	8	-42.1498	74.1957	29
5	-5	8	12.4613	72.7522	5
5	-5	8	-33.6666	67.4761	7
5	-5	9	1536.65	141.500	7
-5	-5	-9	1446.90	126.601	20
5	5	10	116.242	59.0300	29
5	-5	10	2486.65	150.258	5
5	6	-13	4379.81	273.225	19
5	-6	-13	4178.78	267.446	11
-5	-6	12	1952.54	180.025	22
5	-6	-12	1953.37	183.051	11
5	6	-11	6595.57	452.528	19
-5	-6	11	6624.60	458.353	22
5	-6	-11	7339.36	463.467	11
5	6	-10	203.771	93.2135	19
-5	-6	10	97.2084	85.9127	22
5	-6	-10	218.421	91.4170	11
-5	-6	9	5601.22	520.964	22

5	6	-9	9006.47	535.195	19
5	6	-8	1542.94	204.678	19
-5	-6	8	1535.40	189.875	27
5	-6	-8	2031.18	225.041	15
-5	-6	8	1275.36	211.656	22
5	6	-8	1318.46	364.412	24
-5	-6	7	3558.12	351.388	22
5	6	-7	4595.44	360.296	19
5	-6	-7	3926.68	352.930	3
5	-6	-7	4271.54	352.917	15
5	-6	-7	4165.25	351.024	11
-5	-6	7	4459.66	344.001	27
-5	-6	6	6616.45	494.511	22
5	6	-6	6754.20	481.326	19
5	-6	-6	6491.91	684.904	1
5	-6	-6	7601.78	499.460	15
-5	-6	6	7556.00	491.236	27
5	-6	-6	7336.01	496.153	11
5	-6	-5	10554.7	628.689	11
-5	-6	5	10051.3	637.414	22
5	6	-5	10014.3	621.556	19
5	-6	-5	10562.6	649.884	3
5	-6	-5	8186.03	601.243	4
5	-6	-5	10391.1	624.776	15
5	-6	-5	12475.8	949.811	1
-5	-6	5	10168.4	625.294	28
5	-6	-4	145871.	6812.71	11
-5	-6	4	145053.	6836.06	22
5	6	-4	138861.	6811.47	19

5	-6	-4	135719.	6815.05	4
5	-6	-4	143924.	6839.00	3
5	-6	-4	131031.	6791.69	15
-5	-6	4	147370.	6821.23	28
5	-6	-3	81710.7	3951.66	11
5	6	-3	79130.6	3954.64	19
-5	-6	3	83056.5	3979.94	22
5	-6	-3	82026.2	3969.58	4
5	-6	-3	79837.7	3980.69	3
5	-6	-3	92057.7	4434.72	1
-5	-6	3	78608.7	3960.60	28
5	-6	-2	103.395	148.581	1
5	-6	-2	27.6369	61.2051	3
-5	-6	2	-41.6375	67.6361	22
-5	-6	2	-13.4132	65.3656	28
5	-6	-1	796.501	132.399	4
-5	-6	1	662.649	120.686	22
-5	-6	0	29804.0	1511.15	22
5	-6	0	26445.3	1500.42	4
5	-6	1	950.644	144.109	4
-5	-6	-1	1487.11	168.365	22
5	-6	1	1146.44	136.282	5
5	-6	1	912.619	120.104	7
5	-6	1	869.084	133.385	8
5	-6	1	891.926	122.117	14
5	-6	1	991.360	283.751	1
-5	-6	-2	2614.13	239.848	22
5	-6	2	2545.19	252.616	4
5	-6	2	2885.47	229.742	7

5	-6	2	2603.21	226.954	5
5	6	2	2458.67	241.072	18
5	-6	2	2923.67	230.019	14
5	-6	2	2584.31	242.561	8
5	-6	2	2230.82	219.117	10
5	6	3	6821.81	445.968	18
5	-6	3	6719.23	433.194	14
5	-6	3	6406.60	434.966	7
5	-6	3	6952.19	480.351	4
5	-6	3	6567.14	441.497	5
5	6	4	2461.59	227.091	18
5	-6	4	2409.13	255.191	4
5	-6	5	13569.7	779.881	7
5	-6	5	14012.4	794.127	5
5	-6	5	14176.2	837.090	4
5	6	5	12873.0	758.536	18
5	-6	6	456.017	107.234	5
5	-6	6	469.323	129.463	4
5	6	6	409.155	86.0702	18
5	-6	6	364.160	101.691	7
5	-6	7	638.506	118.138	7
5	-6	7	736.436	150.626	4
5	-6	7	699.876	124.248	5
5	-6	8	1842.55	183.725	7
5	-6	8	1932.20	192.414	5
5	-6	9	160.301	65.4216	5
5	-6	10	1102.01	125.483	10
5	-6	10	1207.81	113.949	5
5	-6	11	707.713	72.9927	10

5	7	-13	30.2070	25.6103	19
5	-7	-13	32.3039	26.3138	11
5	7	-12	610.494	90.1422	19
5	-7	-12	547.393	85.4605	11
-5	-7	11	1253.19	141.574	22
5	7	-11	1418.65	158.786	19
-5	-7	10	3297.45	272.227	22
5	7	-10	3313.16	281.427	19
5	7	-9	2290.51	237.208	19
5	-7	-9	2430.73	245.945	11
-5	-7	9	2441.83	243.717	22
-5	-7	8	343.345	108.282	22
5	7	-8	404.598	119.167	19
5	-7	-8	477.664	133.555	3
5	-7	-8	423.390	119.885	11
-5	-7	8	533.999	122.173	27
-5	-7	7	22838.1	1282.55	22
5	7	-7	23269.6	1281.62	19
5	-7	-7	23706.6	1302.70	3
5	-7	-7	22648.2	1279.94	15
-5	-7	7	25063.6	1294.25	27
5	-7	-7	23959.1	1287.20	11
-5	-7	6	8851.10	579.174	22
5	7	-6	8610.90	569.220	19
-5	-7	6	9063.45	579.875	27
5	-7	-6	8396.85	567.506	15
5	-7	-6	8518.10	803.434	1
5	-7	-6	9251.33	580.677	11
5	-7	-5	9953.51	623.262	11

5	7	-5	10029.3	625.377	19
-5	-7	5	10672.6	646.739	22
5	-7	-5	9174.28	613.621	15
5	-7	-5	11461.6	957.176	1
-5	-7	5	10160.8	639.403	27
5	-7	-4	12780.4	732.579	4
5	-7	-4	11655.4	730.907	3
-5	-7	4	12038.8	720.379	22
-5	-7	4	11103.8	715.051	28
5	7	-4	11362.2	696.334	19
5	-7	-4	10976.6	684.880	15
5	-7	-4	14110.2	1091.54	1
5	-7	-4	12591.0	707.520	11
5	-7	-3	550.807	102.961	11
-5	-7	3	368.191	108.760	22
5	7	-3	593.088	112.265	19
5	-7	-3	365.186	100.192	4
5	-7	-3	484.516	103.352	8
5	-7	-3	376.960	108.530	3
5	-7	-3	639.230	237.057	1
-5	-7	3	266.307	94.9849	28
-5	-7	2	8246.59	518.380	22
-5	-7	2	7779.16	515.856	28
5	7	-2	7597.95	482.716	19
5	-7	-2	9478.82	921.151	2
5	-7	-2	9724.54	884.562	1
5	-7	-2	6723.55	465.254	11
5	-7	-2	7434.16	497.084	8
5	-7	-2	7651.64	513.253	4

5	-7	-2	7679.68	517.787	3
5	-7	-1	412.084	104.017	8
5	-7	-1	458.543	103.782	4
5	-7	-1	273.044	71.7618	5
-5	-7	1	445.165	118.404	28
5	-7	-1	-60.2431	137.561	1
5	-7	-1	275.817	221.991	2
5	-7	-1	378.173	111.681	3
-5	-7	1	203.463	89.6533	22
5	-7	0	1513.96	186.833	8
5	-7	0	1796.06	167.579	7
5	-7	0	1689.58	174.288	5
-5	-7	0	1591.28	192.402	22
5	-7	0	1988.26	422.218	2
5	-7	0	1759.15	206.153	4
5	-7	0	1862.84	203.059	3
5	-7	1	2056.81	192.216	14
-5	-7	-1	2563.96	238.559	22
5	-7	1	2270.04	234.203	8
-5	-7	-1	2338.63	229.803	16
5	-7	1	2205.98	210.344	5
5	-7	1	2151.28	198.522	7
5	-7	1	2307.78	246.302	4
5	-7	1	2560.11	233.417	3
5	-7	1	3052.09	524.190	2
5	-7	2	14356.4	725.619	14
-5	-7	-2	13721.9	757.763	22
5	-7	2	10646.2	720.420	10
5	-7	2	12328.9	755.318	8

5	7	2	13161.1	748.659	18
-5	-7	-2	12648.4	743.989	16
5	-7	2	12543.7	720.800	7
5	-7	2	11993.2	727.481	5
5	-7	2	11230.0	1164.92	2
5	-7	2	13170.1	783.265	4
5	-7	3	1452.62	178.367	10
5	7	3	1182.80	160.181	18
5	-7	3	1647.99	208.374	4
5	-7	3	1143.99	173.108	8
5	-7	3	1233.46	157.316	7
5	-7	3	1501.36	177.412	5
5	-7	3	1281.54	369.620	2
-5	-7	-3	1655.13	185.706	22
-5	-7	-4	916.048	152.160	22
5	7	4	1194.62	148.266	18
5	-7	4	893.518	165.032	4
5	-7	4	867.376	146.271	5
5	-7	4	958.836	163.430	8
5	-7	4	1099.98	148.395	7
5	-7	4	990.315	155.345	10
5	-7	4	1065.69	356.490	2
-5	-7	-5	1188.02	165.784	22
5	7	5	1192.53	138.029	18
5	-7	5	1100.34	182.026	4
5	-7	5	1440.26	191.323	8
-5	-7	-5	876.499	132.902	9
5	-7	5	1114.95	165.561	10
5	-7	6	871.631	156.037	8

5	-7	6	980.930	141.591	7
5	-7	6	1031.02	159.393	10
5	-7	6	1202.29	181.311	4
5	-7	6	1057.99	155.790	5
5	-7	7	947.516	149.570	8
5	-7	7	914.627	135.403	7
5	-7	7	996.610	152.624	10
5	-7	7	956.605	141.337	5
5	-7	8	572.893	117.931	8
5	-7	8	661.788	116.558	10
5	-7	8	640.799	108.181	5
5	-7	9	26.1392	48.4993	8
5	-7	9	-7.91912	46.2319	5
5	-7	9	38.1216	55.6363	10
5	-7	10	61.1991	32.2757	5
5	-7	10	81.7447	41.6576	10
5	8	-12	82.7027	35.8586	19
5	-8	-11	1732.20	150.157	11
5	8	-11	1498.04	147.428	19
5	-8	-10	4795.93	329.479	11
5	8	-10	4709.80	330.477	19
-5	-8	9	65.6037	53.4085	22
5	8	-9	77.1830	75.1780	19
5	-8	-9	101.301	72.8778	11
-5	-8	8	104.085	80.3376	27
-5	-8	8	157.112	76.8302	22
5	8	-8	212.899	85.6865	19
5	-8	-8	210.625	88.3074	11
5	-8	-7	5093.52	382.241	11

5	8	-7	5352.32	392.776	19
-5	-8	7	5147.13	399.937	27
-5	-8	7	5260.46	386.330	22
5	-8	-7	5482.16	409.430	3
5	-8	-6	6208.90	429.959	11
5	8	-6	5820.40	423.372	19
-5	-8	6	6586.92	458.485	27
5	-8	-6	5609.44	422.072	15
5	-8	-6	6083.49	455.120	3
-5	-8	6	6404.52	438.506	22
5	8	-5	158.041	74.6197	19
5	-8	-5	209.303	87.5549	15
-5	-8	5	34.5551	74.6631	27
-5	-8	5	93.1480	79.0438	22
5	-8	-5	83.5500	66.5676	11
5	-8	-4	883.870	131.565	11
-5	-8	4	988.332	151.525	22
5	8	-4	818.414	130.232	19
5	-8	-4	910.559	131.782	15
5	-8	-4	1087.09	166.772	4
5	-8	-4	893.291	161.777	3
-5	-8	4	766.171	152.924	28
5	-8	-3	2343.82	241.421	8
5	-8	-3	2537.08	220.206	11
5	8	-3	2256.29	217.873	19
5	-8	-3	1997.02	236.243	4
5	-8	-3	2261.77	252.002	3
-5	-8	3	2681.42	247.576	22
-5	-8	3	2538.27	260.414	28

5	-8	-2	125.559	80.0461	8
-5	-8	2	207.613	89.9140	28
5	-8	-2	105.146	75.8717	4
5	-8	-2	204.630	95.1642	3
-5	-8	2	135.019	80.9666	22
-5	-8	1	1309.82	168.291	22
5	-8	-1	1378.69	177.059	8
5	-8	-1	1000.43	135.148	5
5	-8	-1	1199.28	174.491	4
5	-8	-1	1006.17	164.276	3
5	-8	-1	673.395	268.685	2
-5	-8	1	1189.55	176.819	28
-5	-8	0	6029.32	413.011	22
-5	-8	0	6381.74	448.351	28
-5	-8	0	5495.73	414.603	16
5	-8	0	5664.62	367.122	7
5	-8	0	4969.56	377.810	5
5	-8	0	5717.70	754.214	2
5	-8	0	5926.52	436.114	4
5	-8	0	6027.82	425.579	3
5	-8	0	5274.79	409.504	8
5	-8	1	484.209	101.140	10
-5	-8	-1	404.869	103.198	22
-5	-8	-1	718.044	131.756	16
5	8	1	525.156	120.688	18
5	-8	1	493.886	96.6128	7
5	-8	1	427.267	111.774	8
5	-8	1	599.181	133.656	4
5	-8	1	478.192	105.214	5

5	-8	1	310.256	92.8275	3
-5	-8	-2	6855.90	455.699	22
5	-8	2	6943.50	478.102	8
5	-8	2	6241.84	444.149	10
5	8	2	7080.39	454.131	18
-5	-8	-2	5896.25	445.936	16
5	-8	2	6763.12	434.219	7
5	-8	2	6895.53	453.756	5
5	-8	2	7128.41	492.994	4
5	-8	2	5803.56	429.894	3
5	-8	2	7154.55	865.879	2
-5	-8	-3	3378.39	283.523	22
5	-8	3	3180.96	571.120	2
5	8	3	2909.34	259.231	18
5	-8	3	2993.74	302.359	4
5	-8	3	3090.85	280.348	10
5	-8	3	3746.31	314.663	8
5	-8	3	3683.54	292.605	5
5	-8	3	3456.33	274.454	7
5	8	4	489.497	91.7509	18
5	-8	4	448.535	103.415	7
5	-8	4	418.453	110.504	8
5	-8	4	513.691	137.003	4
5	-8	4	423.592	111.655	5
-5	-8	-4	380.223	104.032	9
5	-8	4	256.424	91.3134	10
-5	-8	-4	335.230	98.0684	22
-5	-8	-5	3037.50	258.985	9
5	-8	5	3151.74	284.177	10

5	-8	5	3416.55	298.924	8
5	-8	5	3299.72	308.431	4
5	-8	5	3488.29	273.951	7
5	-8	6	757.676	132.218	10
-5	-8	-6	629.045	102.408	9
5	-8	6	769.306	126.960	5
5	-8	6	606.965	122.631	8
5	-8	7	5493.46	363.029	10
5	-8	7	4797.70	353.790	8
5	-8	7	5047.28	350.955	5
5	-8	8	819.809	108.135	5
5	-8	8	645.089	101.576	10
-5	-8	-8	629.608	82.4610	15
5	-8	8	674.288	100.593	8
5	-8	9	979.815	98.6249	10
5	-8	9	926.122	94.5123	5
5	9	-11	3.81108	23.2701	19
5	-9	-11	58.1159	26.8997	11
5	9	-10	54.9686	43.1139	19
5	-9	-10	52.8417	41.4515	11
5	9	-9	-36.3576	49.7377	19
5	-9	-9	35.2181	48.1789	11
5	9	-8	3887.97	294.344	19
5	-9	-8	4100.73	296.567	11
-5	-9	8	4544.24	324.101	27
5	9	-7	691.335	113.930	19
5	-9	-7	645.472	129.253	3
-5	-9	7	616.022	123.968	27
5	-9	-7	635.086	109.931	11

5	9	-6	8698.21	537.939	19
-5	-9	6	8504.57	532.117	22
5	-9	-6	9070.47	580.822	3
5	-9	-6	8685.87	532.089	11
-5	-9	6	9308.17	580.231	27
-5	-9	5	133.925	88.7850	27
5	-9	-5	248.128	76.4790	11
5	9	-5	168.284	67.4210	19
-5	-9	5	153.593	72.8952	22
5	9	-4	13239.1	773.803	19
5	-9	-4	13475.7	835.893	4
5	-9	-4	14627.5	844.587	3
5	-9	-4	13757.1	767.997	11
-5	-9	4	15081.9	848.414	27
-5	-9	4	13811.4	836.934	28
-5	-9	4	14360.9	800.805	22
5	-9	-3	47.0109	73.1724	8
-5	-9	3	149.100	82.8192	22
5	-9	-3	142.371	90.5819	4
5	-9	-3	139.202	104.744	3
-5	-9	3	141.378	93.4682	28
-5	-9	2	13.6050	61.8247	22
5	-9	-2	-31.9516	79.5275	8
5	-9	-2	18.0235	73.2568	4
5	-9	-2	-17.1301	81.6434	3
-5	-9	2	35.5054	76.5332	28
-5	-9	1	243.388	85.9669	22
-5	-9	1	265.935	102.398	16
5	-9	-1	437.018	118.374	8

5	-9	-1	454.783	122.103	4
5	-9	-1	365.597	115.469	3
-5	-9	1	232.126	98.8107	28
5	-9	0	16592.6	960.570	8
-5	-9	0	16797.3	960.528	16
5	-9	0	16302.0	919.327	5
-5	-9	0	16586.1	932.681	22
5	-9	0	17019.1	983.938	4
5	-9	0	17643.2	965.788	3
-5	-9	0	16918.4	980.058	28
-5	-9	-1	538.088	133.518	28
-5	-9	-1	186.024	85.6240	22
5	9	1	364.626	98.1693	18
-5	-9	-1	373.607	108.312	16
5	-9	1	343.412	103.510	8
5	-9	1	258.967	93.6514	3
5	-9	1	438.473	125.421	4
5	-9	1	250.004	94.7704	5
-5	-9	-2	4494.49	319.901	22
5	-9	2	4261.22	352.319	4
5	-9	2	3792.70	302.605	3
5	9	2	4058.28	304.947	18
5	-9	2	3552.92	309.334	5
-5	-9	-2	4234.54	349.466	28
-5	-9	-2	4013.44	324.891	16
5	-9	2	3712.48	327.786	8
-5	-9	-3	716.880	119.470	22
5	9	3	531.995	96.2029	18
5	-9	3	520.212	124.897	8

5	-9	3	581.664	109.109	10
5	-9	3	386.250	121.331	4
5	-9	3	690.919	126.869	5
-5	-9	-3	582.803	126.041	28
5	-9	4	5297.31	385.260	5
5	-9	4	5833.89	405.441	8
-5	-9	-4	5354.96	375.751	9
5	-9	4	5374.87	382.126	10
-5	-9	-4	5354.71	374.685	22
5	-9	5	144.166	73.6127	10
-5	-9	-5	131.576	62.1419	9
5	-9	5	213.604	81.7237	8
5	-9	6	2019.63	186.612	8
5	-9	6	2380.86	190.678	10
5	-9	7	79.0199	65.6564	5
5	-9	7	73.3893	52.5250	10
5	-9	8	85.3748	49.5624	4
-5	-9	-8	-11.1206	25.2689	15
5	10	-9	40.5813	22.8738	19
5	10	-8	530.182	70.4842	19
-5	-10	7	258.150	80.7724	27
5	10	-7	230.325	56.6267	19
-5	-10	6	950.689	142.503	27
5	-10	-6	940.855	144.988	3
-5	-10	5	116.365	79.7820	27
-5	-10	4	11745.8	684.897	27
5	-10	-4	10376.7	666.041	3
-5	-10	3	97.1863	80.6637	28
5	-10	-3	64.8217	87.1633	4

5	-10	-3	30.9303	81.6865	3
-5	-10	3	29.7266	51.3899	22
-5	-10	2	946.452	129.052	22
-5	-10	2	1117.95	168.429	28
5	-10	-2	1264.22	180.017	4
-5	-10	2	1277.51	151.952	3
5	-10	-2	925.004	153.468	3
-5	-10	2	1114.46	165.623	16
5	-10	-2	1219.69	163.452	8
5	-10	-1	4181.46	333.369	8
-5	-10	1	3961.00	342.072	28
-5	-10	1	4420.73	305.338	22
-5	-10	1	3992.97	299.996	3
5	-10	-1	4464.89	357.040	4
5	-10	-1	4085.30	331.201	3
-5	-10	1	3955.69	329.423	16
-5	-10	0	1066.16	154.961	16
5	10	0	768.850	129.091	18
5	-10	0	922.581	147.290	8
5	-10	0	1125.95	168.680	4
-5	-10	0	1174.27	168.227	28
5	-10	0	1071.15	154.235	3
-5	-10	0	1028.49	130.642	22
-5	-10	-1	1648.87	165.975	22
-5	-10	-1	1524.06	190.100	28
5	10	1	1557.27	164.030	18
5	-10	1	1455.47	181.782	8
-5	-10	-1	1372.13	173.358	16
5	-10	1	1533.48	199.952	4

5	-10	1	1584.70	177.052	3
5	-10	2	79.3353	80.6150	4
5	-10	2	96.9301	79.5949	8
-5	-10	-2	-106.596	72.5776	28
5	10	2	44.7851	49.8511	18
-5	-10	-2	-10.0469	56.8415	22
5	-10	3	115.074	74.1517	5
-5	-10	-3	99.1724	72.1842	28
-5	-10	-3	185.309	79.1327	9
5	-10	3	77.1444	69.3449	8
-5	-10	-4	9577.33	565.064	28
-5	-10	-4	8424.06	525.094	9
5	-10	4	9133.29	548.852	8
5	-10	4	9724.17	551.095	5
-5	-10	-5	151.123	55.1021	9
5	-10	5	321.119	77.3942	8
-5	-10	-7	1305.05	107.114	15
5	-10	7	1928.26	140.020	4
-5	-11	8	165.419	44.0488	16
-5	-11	8	789.355	72.9318	3
-5	-11	7	6.80376	41.4810	3
-5	-11	7	-7.41680	43.9446	16
-5	-11	6	1519.51	144.910	27
-5	-11	6	1279.74	131.855	3
5	-11	-6	1238.96	139.623	3
-5	-11	5	446.103	88.2651	3
-5	-11	5	578.866	100.002	27
-5	-11	5	436.097	98.1218	16
-5	-11	4	427.662	99.3217	16

-5	-11	4	418.960	88.5204	3
5	-11	-4	342.109	93.1217	3
5	-11	-3	1963.22	205.451	3
-5	-11	3	2157.90	189.925	3
-5	-11	3	2507.77	217.390	16
5	-11	-2	6014.49	398.085	3
5	-11	-2	6155.65	414.310	4
-5	-11	2	5647.80	367.175	3
-5	-11	2	6541.48	409.190	16
-5	-11	2	6441.98	411.172	27
5	11	-2	5140.86	370.701	18
5	-11	-1	3784.05	283.671	3
5	-11	-1	4058.51	309.478	4
-5	-11	1	3299.46	246.456	3
-5	-11	1	3534.38	283.991	16
-5	-11	1	3913.45	292.897	27
-5	-11	1	3881.45	301.893	28
5	11	-1	3781.93	269.903	18
5	-11	0	197.736	71.9677	8
5	-11	0	160.946	79.9108	4
5	-11	0	215.469	72.7206	3
-5	-11	0	130.001	69.4961	27
-5	-11	0	208.197	77.5550	28
-5	-11	0	134.969	68.1346	16
5	11	0	206.557	64.4889	18
5	-11	1	253.194	78.0877	8
5	11	1	285.703	58.1984	18
5	-11	1	245.834	87.9065	4
-5	-11	-1	141.410	69.0257	27

-5	-11	-1	234.411	80.0532	28
-5	-11	-2	1197.07	141.802	28
-5	-11	-2	919.201	125.816	9
-5	-11	-3	479.480	86.0012	9
-5	-11	-4	360.131	62.7088	9
5	12	-6	243.420	38.4256	18
5	12	-5	299.561	50.1193	18
-5	-12	5	227.102	42.1200	3
-5	-12	4	4918.52	328.501	16
-5	-12	4	5295.76	313.875	3
5	-12	-4	6417.34	344.934	3
5	12	-4	6331.88	332.351	18
5	-12	-3	14.1774	44.9849	3
-5	-12	3	-9.07035	28.4108	3
-5	-12	3	-21.6440	41.4729	16
5	12	-3	-11.1371	38.7942	18
5	-12	-2	261.400	64.0241	3
-5	-12	2	133.186	52.8462	16
5	12	-2	195.407	48.1473	18
-5	-12	1	2052.37	158.609	16
5	12	-1	1629.67	122.296	18
-5	-12	0	381.115	74.8801	9
-5	-12	-1	291.250	64.2157	9
-5	-12	-2	36.7271	35.0027	9
6	0	-15	-18.8816	30.3489	20
6	0	-14	8715.72	430.557	20
-6	0	14	5593.43	386.618	29
6	0	-13	-12.3585	68.7824	20
-6	0	13	11.5276	64.1576	29

6	0	-12	9553.21	625.790	20
-6	0	12	10889.6	642.387	29
6	0	-11	16.9594	77.0723	20
6	0	-10	2202.00	474.697	17
6	0	-10	1870.37	228.884	20
6	0	-9	-74.6701	151.758	17
6	0	-9	16.5678	71.4434	20
6	0	-9	62.5415	59.6375	19
6	0	-8	4873.42	363.960	20
6	0	-8	4229.76	324.871	19
6	0	-8	4526.57	329.883	11
6	0	-7	-59.8699	172.092	17
6	0	-6	66667.7	3694.82	17
6	0	-5	-48.3039	109.777	17
6	0	-4	9019.99	724.370	25
6	0	-4	8501.10	504.678	26
6	0	-4	10329.5	813.573	17
6	0	-3	25.6022	42.4896	26
6	0	-3	34.4067	104.888	25
6	0	-3	-40.1303	122.335	17
6	0	-2	41179.1	2081.70	26
6	0	-2	42167.4	2357.33	25
6	0	-2	47614.8	2427.75	17
6	0	-1	-17.1995	46.2408	2
6	0	-1	-36.4636	117.168	25
6	0	-1	9.03037	51.9085	26
6	0	-1	38.8575	118.453	17
6	0	0	26320.7	1351.67	26
6	0	0	25879.9	1451.62	2

6	0	0	27251.1	1580.93	1
6	0	0	27490.9	1656.27	25
6	0	1	23.7095	53.9053	2
6	0	1	111.196	84.7636	1
6	0	1	42.6504	122.594	25
6	0	2	61754.6	3335.76	2
6	0	2	60727.0	3286.86	1
6	0	2	61199.7	3509.38	25
-6	0	-3	-51.5909	128.422	23
6	0	3	-36.2187	82.3126	2
6	0	4	134.753	70.5576	18
6	0	4	71.6622	56.1978	6
-6	0	-4	52.7791	151.756	23
6	0	4	131.691	109.351	2
6	0	5	25.8708	69.5518	18
-6	0	-5	57.8756	155.594	23
6	0	5	-10.8298	49.2137	6
6	0	5	-51.8347	91.2284	2
6	0	6	15499.8	842.885	18
-6	0	-6	14259.5	1213.52	23
6	0	6	14178.5	809.694	6
6	0	6	14762.5	805.250	7
6	0	7	104.892	75.3746	18
-6	0	-7	64.2285	159.869	23
6	0	7	-9.61936	49.8536	7
6	0	8	24078.4	1267.30	18
6	0	10	4274.28	278.921	5
6	0	10	5167.47	354.798	29
-6	0	-11	-6.65929	40.6071	20

6	0	11	48.2185	49.0020	29
-6	0	-12	3198.60	187.749	20
6	1	-15	2223.13	148.721	20
-6	-1	14	1143.63	124.618	29
6	1	-14	2219.04	166.310	20
-6	1	13	1126.67	143.653	29
-6	-1	13	1045.04	133.150	29
6	1	-13	1009.67	144.341	20
-6	1	12	9674.27	578.573	29
-6	-1	12	9197.16	566.553	29
6	1	-12	7788.08	550.341	20
6	1	-11	215.466	75.8404	19
6	-1	-11	187.370	74.7659	11
6	1	-11	349.821	118.961	20
6	1	-10	3186.28	301.369	20
6	-1	-10	3825.02	319.774	20
6	1	-10	3612.05	294.383	19
6	-1	-10	1840.94	402.696	1
6	1	-10	2849.98	540.958	17
6	-1	-10	3249.60	588.012	17
6	-1	-10	3628.27	290.317	11
-6	1	10	3985.39	559.208	30
-6	-1	10	3977.54	560.561	30
6	1	-9	314.506	90.1641	19
6	1	-9	472.894	120.826	20
6	-1	-9	395.182	113.664	20
6	1	-9	429.638	241.988	17
6	-1	-9	538.638	259.945	17
6	-1	-9	308.513	91.8913	11

-6	-1	9	573.997	248.104	30
-6	1	9	583.279	222.488	30
6	1	-8	26401.2	1397.40	19
6	1	-8	26483.3	1412.21	20
6	-1	-8	27044.9	1414.11	20
6	-1	-8	24331.0	1385.88	11
-6	-1	8	30298.8	1811.24	30
-6	1	8	27804.4	1770.72	30
6	1	-8	30034.3	1915.45	17
6	-1	-8	27893.0	1927.81	17
6	1	-7	1232.39	318.728	17
6	-1	-7	1041.12	301.811	17
-6	-1	7	1135.39	275.622	30
-6	1	7	909.137	261.176	30
6	1	-7	950.684	136.680	20
6	-1	-7	870.054	137.987	20
6	1	-6	8314.95	813.641	17
6	1	-5	50984.6	2884.32	17
6	1	-4	43529.0	2292.35	17
6	1	-4	39401.9	2194.10	25
6	1	-4	37368.1	1907.77	26
6	1	-3	7431.78	648.521	17
6	1	-3	6493.35	391.632	26
6	1	-3	6824.84	595.699	25
6	-1	-3	6062.62	386.426	26
6	1	-2	38015.6	2069.41	17
6	-1	-2	37368.1	2084.61	1
6	1	-2	37133.1	2021.06	25
6	1	-2	33371.9	1730.59	26

6	-1	-2	34568.4	1732.89	26
6	-1	-1	553.519	180.576	1
6	-1	-1	206.065	109.160	2
6	1	-1	429.005	82.5104	26
6	1	-1	331.503	141.003	25
6	-1	-1	321.909	74.5235	26
6	-1	0	22329.0	1354.18	1
6	-1	0	22670.0	1310.35	2
6	1	0	21545.4	1408.64	25
6	-1	0	22765.7	1412.92	25
6	1	0	21235.7	1120.87	26
6	-1	0	21056.5	1117.41	26
6	1	1	2932.87	411.280	25
6	-1	1	3507.79	439.138	25
6	1	1	3115.13	343.648	1
6	-1	1	3232.84	376.699	2
6	-1	1	2985.20	365.081	1
6	1	1	3420.23	262.211	29
-6	1	-2	482.337	218.657	23
6	-1	2	428.043	131.062	1
6	-1	2	325.759	138.922	2
6	1	2	336.560	183.786	25
6	-1	2	282.100	144.580	25
6	1	2	535.347	108.644	29
6	-1	2	382.519	94.4586	29
6	1	3	7600.58	510.519	29
6	-1	3	8097.71	514.083	29
6	-1	3	7506.63	702.494	2
6	-1	3	7998.23	792.504	25

6	1	3	6716.83	748.116	25
-6	1	-3	9773.20	839.070	23
-6	-1	-4	8575.26	810.351	23
-6	1	-4	8151.69	795.865	23
6	1	4	8771.11	532.393	29
6	-1	4	8955.36	534.490	29
6	-1	4	7750.04	469.684	6
6	1	4	6824.68	452.055	6
6	-1	4	8354.36	777.633	2
6	1	4	7223.00	479.861	18
6	1	4	8163.10	827.254	25
6	-1	4	8486.87	836.494	25
6	-1	4	6902.11	460.787	14
6	-1	5	11939.8	1065.37	2
6	1	5	12461.0	723.474	18
6	-1	5	11661.5	717.085	18
6	1	5	13640.7	780.695	29
6	-1	5	13206.7	772.688	29
6	1	5	12371.1	694.231	6
6	-1	5	11990.7	702.763	7
6	-1	5	12426.8	707.621	6
6	1	5	12772.7	1169.03	25
6	-1	5	12670.5	1149.87	25
-6	1	-5	11980.5	1047.34	23
-6	-1	-5	15298.3	1174.72	23
6	-1	6	44301.9	2871.96	2
6	-1	6	48504.2	2416.04	18
6	1	6	49720.3	2414.76	18
6	1	6	52250.9	2487.75	29

6	-1	6	54354.4	2489.72	29
6	1	6	46867.9	2377.17	6
6	-1	6	47635.2	2395.81	6
6	-1	6	46506.7	2382.38	7
6	1	6	45569.7	2368.21	7
6	1	6	47541.8	2992.16	25
6	-1	6	45967.7	2945.80	25
-6	1	-6	50107.9	2900.56	23
-6	-1	-6	48546.7	2917.97	23
6	-1	7	11461.6	1128.62	2
6	1	7	14670.8	833.669	29
6	-1	7	13970.9	822.433	29
6	-1	7	12978.7	762.981	18
6	1	7	13227.9	756.540	18
6	-1	7	13059.6	729.842	7
-6	1	-7	9931.05	1049.07	23
-6	-1	-7	13394.8	1166.47	23
6	1	7	11949.3	1183.90	25
6	-1	7	14775.1	1257.19	25
6	1	8	952.445	131.941	18
6	-1	8	816.291	106.873	7
6	1	8	873.350	159.857	29
6	-1	8	863.593	163.604	29
6	-1	9	79.8888	62.0135	5
6	1	9	199.552	95.7751	29
6	-1	9	163.942	88.3531	29
6	-1	10	12034.6	622.063	5
6	1	10	10791.1	604.524	5
6	1	10	13489.7	704.850	29

6	-1	10	10047.4	666.279	29
-6	1	-10	12185.0	641.030	20
-6	1	-11	6.25185	37.0453	20
-6	-1	-11	6.57196	40.0706	20
6	1	11	18.9441	40.8375	29
6	-1	11	9.01483	45.8036	29
-6	1	-12	106.831	25.3435	20
-6	-1	-12	174.836	33.4357	20
-6	-2	15	17.5572	25.6347	22
6	2	-14	547.314	82.5963	20
6	-2	-14	427.980	77.6503	20
-6	2	14	328.656	65.9443	29
-6	2	13	874.186	126.035	29
-6	-2	13	737.037	110.109	29
6	-2	-13	829.002	101.144	11
6	2	-13	1245.45	143.687	20
6	-2	-13	887.535	128.250	20
6	2	-12	3509.26	290.215	20
6	-2	-12	2909.40	272.859	20
6	-2	-12	3157.90	259.612	11
-6	-2	12	3818.25	288.128	29
-6	2	12	4057.83	306.286	29
6	2	-12	3014.52	257.242	19
6	2	-11	6470.13	465.647	20
6	-2	-11	7107.87	474.151	20
6	2	-11	6122.28	445.355	19
6	-2	-11	6655.80	451.094	11
-6	2	11	7844.27	496.527	29
-6	-2	11	6313.12	461.543	29

6	2	-10	5880.63	429.323	20
6	-2	-10	6174.99	438.235	20
6	2	-10	6497.85	434.693	19
6	2	-10	4143.72	659.575	17
-6	2	10	5864.54	700.898	30
-6	-2	10	4435.42	623.399	30
6	-2	-10	5531.92	413.629	11
6	-2	-9	4477.48	360.063	11
6	2	-9	4915.74	371.677	20
6	-2	-9	4604.86	368.095	20
6	2	-9	4729.76	369.973	19
6	-2	-9	4359.11	591.749	1
6	2	-9	4364.74	639.698	17
6	-2	-9	5002.92	712.594	17
-6	-2	9	5158.96	619.841	30
-6	2	9	4813.96	616.428	30
6	2	-9	5119.45	620.276	24
6	-2	-8	6942.27	472.680	11
6	2	-8	6959.74	472.573	20
6	-2	-8	6463.26	463.688	20
6	2	-8	6601.84	470.625	19
6	-2	-8	7421.98	783.992	1
6	2	-8	7785.53	832.671	17
6	-2	-8	6929.71	829.273	17
-6	-2	8	7916.95	772.121	30
-6	2	8	7066.06	744.179	30
6	2	-8	7630.39	753.304	24
6	2	-7	1543.30	317.237	24
6	2	-7	1459.93	165.758	20

6	-2	-7	1150.98	153.475	20
6	-2	-7	1051.85	152.451	11
6	2	-7	1551.36	335.376	17
6	-2	-7	1501.47	369.805	17
-6	-2	7	1396.10	297.267	30
-6	2	7	1050.72	288.915	30
6	-2	-7	1050.50	313.606	1
6	2	-7	1221.00	162.258	19
-6	-2	6	3917.46	496.403	30
-6	2	6	3238.08	459.749	30
6	-2	-6	2633.50	461.437	17
6	2	-6	3733.95	499.841	17
6	-2	-6	3406.70	509.897	1
-6	2	5	19881.5	1567.29	30
6	2	-5	25101.9	1571.96	17
6	-2	-5	25007.9	1601.77	17
6	-2	-5	23010.6	1581.34	1
6	2	-4	127.114	130.048	17
6	-2	-4	1046.73	249.492	17
6	2	-3	17094.6	1191.31	17
6	-2	-3	18444.8	1229.70	17
6	2	-3	17907.4	1156.30	25
6	-2	-3	17313.5	1152.99	25
6	-2	-3	16935.9	904.223	26
6	2	-3	17145.0	907.526	26
6	-2	-3	18001.5	1244.45	1
6	2	-2	39.3347	113.054	17
6	-2	-2	39.7429	106.850	17
6	2	-2	17.6842	41.1844	26

6	-2	-2	34.4170	85.6693	25
6	-2	-2	17.0482	39.7041	26
6	2	-2	35.5497	88.4884	25
6	-2	-2	-41.3424	126.031	1
6	-2	-2	130.483	114.828	2
6	-2	-1	711.566	241.930	17
6	2	-1	1043.13	119.523	26
6	2	-1	818.406	208.549	25
6	-2	-1	937.913	112.092	26
6	-2	-1	893.793	200.820	25
6	-2	-1	922.146	215.083	1
6	-2	-1	1331.83	241.130	2
6	-2	0	43190.4	2183.95	26
6	2	0	43239.5	2188.67	26
6	2	0	46676.5	2514.85	25
6	-2	0	46938.3	2514.15	25
6	-2	0	43947.6	2449.31	2
6	-2	0	47801.9	2491.74	1
6	2	1	205.305	76.4908	29
6	2	1	437.020	172.360	25
6	-2	1	378.015	154.298	25
6	-2	1	267.410	69.4614	26
6	-2	1	315.675	138.500	1
6	2	1	169.628	92.8267	1
6	-2	1	153.905	111.162	2
-6	-2	-2	4822.47	594.017	23
-6	2	-2	5870.39	660.021	23
6	2	2	4272.88	532.528	25
6	-2	2	5104.69	562.594	25

6	-2	2	4417.41	333.136	29
6	2	2	4819.01	344.398	29
6	-2	2	4097.54	450.885	1
6	-2	2	4658.79	523.616	2
-6	-2	-3	33968.1	1978.13	23
-6	2	-3	33991.0	1949.36	23
6	-2	3	24977.7	1712.39	1
6	-2	3	27952.2	1868.30	2
6	2	3	30661.4	1578.45	29
6	-2	3	31327.8	1575.43	29
6	2	3	31800.0	1967.23	25
6	-2	3	27683.5	1899.65	25
6	2	4	496.899	100.263	18
6	-2	4	432.748	93.8134	7
6	2	4	410.085	80.1139	6
6	-2	4	441.398	95.3712	6
6	2	4	304.217	196.779	25
6	-2	4	233.092	188.574	25
-6	-2	-4	487.670	214.411	23
-6	2	-4	718.495	245.617	23
-6	-2	-4	551.661	99.4341	22
6	-2	4	695.825	116.523	18
6	-2	4	586.845	103.075	14
6	2	4	491.272	119.762	29
6	-2	4	343.972	104.151	29
6	-2	4	455.384	217.571	2
-6	2	-5	14088.9	1206.98	23
-6	-2	-5	17472.6	1311.32	23
-6	-2	-5	14788.5	890.808	22

6	-2	5	17219.7	1340.52	2
6	2	5	16255.4	904.947	18
6	-2	5	16582.7	894.632	6
6	2	5	15210.6	860.250	6
6	-2	5	16663.2	893.318	7
6	2	5	17451.3	958.761	29
6	-2	5	18332.1	958.890	29
6	2	5	16137.9	1344.42	25
6	-2	5	16954.1	1338.91	25
6	2	6	288.063	207.439	25
6	-2	6	137.101	197.502	25
6	-2	6	275.872	172.218	2
6	-2	6	325.943	96.6060	18
-6	2	-6	471.328	216.294	23
-6	-2	-6	189.149	181.735	23
6	-2	6	238.048	81.6984	6
6	-2	6	397.174	89.1118	7
6	2	6	246.858	118.182	29
6	-2	6	218.134	102.112	29
6	2	6	366.505	103.952	18
-6	2	-7	295.776	208.568	23
-6	-2	-7	192.432	195.901	23
6	-2	7	208.990	78.3540	7
6	2	7	298.253	90.9693	18
6	2	7	266.561	124.458	29
6	-2	7	329.892	115.421	29
6	-2	7	281.723	202.774	2
6	2	7	-72.7441	234.053	25
6	-2	7	346.349	233.754	25

6	-2	8	13977.9	743.498	7
6	2	8	13749.5	756.091	18
6	2	8	14565.0	831.623	29
6	-2	8	11751.9	793.588	29
6	-2	9	2007.40	158.458	7
6	2	9	2062.12	218.236	29
6	-2	9	2139.55	183.510	5
-6	2	-10	1022.08	110.750	20
6	-2	10	1178.69	112.084	5
6	2	10	943.151	140.570	29
-6	2	-11	37.7722	53.7258	21
6	2	11	179.305	71.8352	29
-6	-2	-12	785.082	62.3002	20
6	3	-14	156.948	45.7917	20
6	-3	-14	117.842	40.8174	20
-6	3	13	150.779	61.8560	29
6	3	-13	134.710	56.6917	19
6	-3	-13	243.773	64.4767	11
6	3	-13	148.523	63.0986	20
6	-3	-13	206.723	65.9660	20
6	3	-12	1571.24	175.938	19
-6	3	12	1930.74	194.336	29
-6	-3	12	1569.91	166.219	29
6	-3	-12	1722.44	179.943	11
6	3	-12	1668.42	181.280	20
6	-3	-12	1804.96	187.954	20
6	3	-11	4195.77	330.270	19
6	3	-11	3591.52	309.040	20
6	-3	-11	3344.20	308.193	20

-6	3	11	4853.51	354.290	29
-6	-3	11	4109.35	320.360	29
6	-3	-11	4174.34	325.026	11
6	3	-10	1821.69	380.355	24
6	3	-10	2106.95	226.867	20
6	-3	-10	2668.85	248.941	20
6	3	-10	2682.47	255.357	19
6	-3	-10	2262.96	494.009	17
-6	3	10	2083.94	390.207	30
-6	-3	10	1467.25	328.354	30
6	-3	-10	2379.75	242.642	11
6	-3	-9	6067.73	429.812	11
6	3	-9	5668.56	400.449	20
6	-3	-9	5731.61	409.715	20
6	3	-9	5160.09	406.972	19
6	-3	-9	4627.12	579.494	1
6	-3	-9	4545.58	717.337	17
6	3	-9	3616.70	581.843	17
-6	-3	9	5753.50	653.377	30
-6	3	9	6354.40	699.130	30
6	3	-9	4773.34	654.254	24
6	3	-8	367.290	186.668	24
6	3	-8	144.610	76.5035	20
6	-3	-8	259.880	90.5625	20
6	3	-8	274.089	94.2341	19
6	-3	-8	-53.8202	109.481	1
6	-3	-8	-70.1534	142.650	17
6	3	-8	-60.2042	122.444	17
-6	-3	8	-49.9005	113.481	30

-6	3	8	266.017	162.255	30
6	-3	-8	161.762	87.1086	11
6	-3	-7	15633.1	897.726	11
6	3	-7	15194.7	879.689	20
6	-3	-7	15816.7	883.741	20
6	3	-7	15093.9	896.933	19
6	3	-7	15455.6	1231.59	24
6	3	-7	15503.9	1229.31	17
6	-3	-7	18646.0	1380.30	17
6	-3	-7	19255.8	1309.45	1
-6	-3	7	16973.8	1203.56	30
-6	3	7	14425.8	1174.51	30
6	-3	-6	1573.38	181.230	11
6	-3	-6	2242.71	412.252	17
6	3	-6	1647.23	333.975	17
-6	3	6	1595.10	323.590	30
6	-3	-6	1834.09	355.307	1
6	3	-6	1619.37	186.135	19
-6	3	5	9483.11	802.951	30
6	-3	-5	9526.39	796.792	1
6	3	-5	9107.64	748.184	17
6	-3	-5	9955.20	818.608	17
6	3	-4	7445.59	701.542	17
6	-3	-4	8322.26	760.133	17
6	-3	-3	6589.38	636.815	1
6	3	-3	5414.00	535.249	25
6	-3	-3	5253.81	513.598	25
6	3	-3	5103.60	339.141	26
6	-3	-3	5620.26	340.366	26

6	3	-3	4492.71	532.368	17
6	-3	-3	5507.64	583.263	17
6	-3	-2	5472.29	562.738	2
6	-3	-2	5950.55	598.165	1
6	-3	-2	5408.48	525.307	25
6	-3	-2	5108.38	332.393	26
6	3	-2	5392.21	340.299	26
6	3	-2	4530.01	496.117	25
6	-3	-2	5241.95	577.660	17
6	3	-1	34259.7	1704.24	26
6	-3	-1	33431.7	1956.96	25
6	-3	-1	33114.6	1694.42	26
6	3	-1	33561.3	1976.71	25
6	-3	-1	35951.3	2036.29	2
6	-3	-1	36702.4	2058.58	1
6	3	0	1156.52	133.316	26
6	-3	0	1301.69	137.883	26
6	3	0	1052.79	239.740	25
6	-3	0	1317.78	254.512	25
6	-3	0	1569.95	293.487	1
6	-3	0	1037.47	243.424	2
6	3	1	7209.25	688.759	25
6	-3	1	6910.70	657.288	25
6	-3	1	6712.59	430.112	26
6	-3	1	6685.33	673.352	2
6	-3	1	7444.92	667.790	1
-6	-3	-1	9024.41	831.561	23
6	-3	2	35233.6	2133.31	1
6	-3	2	34117.9	2224.21	2

-6	3	-2	33912.2	2289.58	23
6	3	2	34782.8	2217.32	25
6	-3	2	34478.1	2183.00	25
6	3	2	39390.3	1899.49	29
6	3	3	5410.04	616.443	25
6	-3	3	4508.11	557.712	25
-6	-3	-3	4037.25	311.124	22
-6	-3	-3	5070.04	588.262	23
-6	3	-3	4664.72	549.043	23
6	-3	3	3231.95	453.915	1
6	-3	3	3499.00	526.880	2
6	3	3	4294.56	322.357	18
6	3	3	4240.58	304.333	19
6	-3	3	4144.05	310.308	18
6	-3	3	4404.23	310.377	6
6	-3	3	4025.76	306.826	7
6	-3	3	5204.93	347.377	29
6	3	3	4918.20	350.562	29
6	-3	3	4018.27	304.303	14
6	3	4	511.265	110.179	18
6	-3	4	527.676	107.257	7
6	3	4	544.252	215.106	25
6	-3	4	959.156	270.686	25
-6	-3	-4	931.038	262.856	23
-6	3	-4	648.839	211.390	23
-6	-3	-4	729.443	120.367	22
6	-3	4	619.202	113.300	18
6	-3	4	808.213	122.815	14
6	3	4	752.562	137.563	29

6	-3	4	710.607	133.110	29
6	-3	4	635.099	251.854	2
6	3	5	584.266	137.713	29
6	-3	5	622.890	127.787	29
6	3	5	198.522	191.539	25
6	-3	5	615.460	235.099	25
-6	3	-5	749.128	244.219	23
-6	-3	-5	829.433	263.391	23
-6	-3	-5	518.814	107.175	22
6	-3	5	481.985	100.749	6
6	-3	5	541.843	107.129	7
6	-3	5	489.958	226.055	2
6	3	5	277.847	90.0409	18
6	-3	6	10798.0	1065.71	2
6	3	6	11077.6	629.837	18
6	3	6	10933.9	679.225	29
6	-3	6	9667.11	649.116	29
6	-3	6	11079.5	623.548	7
6	3	6	11112.5	1053.26	25
6	-3	6	10660.8	1008.92	25
-6	-3	-6	11560.2	1033.12	23
-6	-3	-7	13691.4	1194.46	23
6	3	7	13826.1	837.035	29
6	-3	7	15235.1	788.586	7
6	3	7	14880.4	788.023	18
6	-3	7	15501.3	817.444	5
6	-3	7	15688.0	1310.85	2
6	3	7	14949.9	1259.93	25
6	-3	8	5582.36	334.171	7

6	3	8	5837.56	336.475	18
6	-3	8	6556.72	369.948	5
6	-3	9	3057.96	190.143	7
6	-3	9	3516.57	220.311	5
-6	3	-9	3337.55	204.090	20
6	3	10	21.8569	60.9467	29
-6	3	-10	83.7560	43.5989	20
-6	-3	-10	119.896	52.8966	20
6	-3	10	88.4265	48.5377	5
-6	3	-11	90.0769	26.4811	20
-6	-3	-11	180.905	43.3848	20
6	-3	11	141.755	48.4343	4
-6	-4	14	306.481	58.3155	22
6	-4	-14	234.574	36.4412	11
6	4	-13	717.154	87.2771	20
-6	4	13	442.438	77.4375	29
6	-4	-13	625.894	93.1546	11
6	4	-13	632.583	91.4701	19
6	4	-12	-9.92317	52.3947	20
6	-4	-12	40.0040	56.5820	20
6	4	-12	-23.8095	62.8573	19
-6	4	12	64.0845	58.4460	29
6	-4	-12	-11.5913	62.3254	11
6	4	-11	581.745	111.421	20
6	-4	-11	671.873	114.090	20
-6	4	11	600.337	137.029	29
6	4	-11	487.996	116.528	19
-6	-4	11	496.094	93.0043	29
6	-4	-11	610.250	121.076	11

6	4	-10	23030.1	1092.02	19
-6	4	10	22268.8	1089.26	29
6	-4	-10	22151.8	1078.59	11
6	4	-10	21365.0	1040.70	20
6	-4	-10	21245.3	1038.67	20
6	4	-9	310.790	92.6970	20
6	-4	-9	226.343	84.6748	20
6	4	-9	417.039	115.059	19
-6	4	9	217.747	156.915	30
-6	-4	9	384.206	176.362	30
6	-4	-9	72.9021	128.868	17
6	-4	-9	351.020	116.201	11
6	4	-9	68.3325	120.863	24
6	-4	-8	12871.6	769.727	11
6	4	-8	11985.3	727.304	20
6	-4	-8	12471.1	732.337	20
6	4	-8	12520.5	763.615	19
6	-4	-8	12176.5	1161.75	17
6	4	-8	12372.5	1133.48	24
-6	-4	8	13302.0	1041.94	30
-6	4	8	12812.0	1061.20	30
6	-4	-8	12992.6	1062.37	1
6	-4	-7	7155.97	487.822	11
6	4	-7	6872.36	456.020	20
6	-4	-7	7203.69	457.701	20
-6	-4	7	5904.16	689.917	30
-6	4	7	6713.32	721.297	30
6	4	-7	4690.04	643.735	17
6	-4	-7	7606.94	837.889	17

6	4	-7	7770.45	817.577	24
-6	-4	7	7155.85	448.522	27
6	-4	-7	8678.18	798.453	1
6	-4	-6	2869.31	267.233	3
6	-4	-6	2243.60	233.676	11
6	4	-6	2852.82	235.816	20
6	-4	-6	2698.13	227.080	20
-6	-4	6	2975.98	232.055	27
6	4	-6	1783.76	361.177	17
6	-4	-6	2562.04	477.457	17
6	-4	-6	3653.85	493.928	1
-6	-4	6	694.633	362.826	30
-6	4	6	2202.58	393.285	30
6	-4	-5	3962.38	302.074	3
6	4	-5	3405.21	282.016	19
6	-4	-5	3800.18	512.519	1
-6	-4	5	2887.05	281.189	22
6	-4	-5	3542.91	509.293	17
6	-4	-4	6412.30	645.340	17
6	-4	-4	7705.49	712.871	1
-6	-4	4	6822.63	369.518	28
-6	-4	3	12121.4	711.784	28
6	4	-3	12835.6	932.033	25
6	4	-3	14149.6	715.553	26
6	-4	-3	13424.1	1016.80	17
6	4	-2	594.847	95.6123	26
6	4	-2	1089.47	228.066	25
6	-4	-2	695.828	96.1765	26
6	-4	-2	750.408	184.327	25

-6	-4	2	750.792	103.546	28
6	-4	-2	690.989	237.359	1
6	-4	-2	869.808	266.313	2
6	-4	-2	288.044	165.010	17
6	-4	-1	20058.5	1380.89	2
6	-4	-1	20389.3	1369.30	1
6	4	-1	19319.6	1269.10	25
6	4	-1	19763.5	1013.67	26
6	-4	-1	18580.3	999.034	26
6	-4	-1	20029.8	1253.77	25
6	-4	0	6861.85	690.917	2
6	-4	0	6599.06	641.850	1
6	4	0	5949.76	590.166	25
6	-4	0	6392.18	581.662	25
6	-4	0	5423.51	357.689	26
6	4	0	5632.57	369.169	26
-6	-4	-1	-47.8978	119.237	23
-6	4	-1	-48.8521	131.353	23
6	-4	1	67.3724	49.9144	26
6	-4	1	117.259	105.103	25
6	4	1	-43.9365	109.379	25
6	4	1	97.6665	55.1765	26
6	-4	1	54.5178	135.712	2
6	-4	1	-45.6327	113.600	1
6	4	2	4796.93	552.254	25
6	-4	2	4665.98	523.405	25
-6	-4	-2	4653.41	331.194	22
-6	-4	-2	3805.40	532.659	23
-6	4	-2	4838.80	536.138	23

6	-4	2	4651.79	542.031	1
6	-4	2	4949.52	605.783	2
6	-4	2	4015.90	298.300	18
6	4	2	4190.48	306.339	19
6	4	2	4061.95	321.327	18
6	-4	2	3987.83	299.759	26
6	-4	2	4723.47	313.984	6
6	-4	2	4342.85	311.897	7
6	-4	2	4118.54	308.899	14
6	4	3	2205.00	404.379	25
6	-4	3	1953.98	347.558	25
-6	-4	-3	1882.20	201.295	22
-6	-4	-3	2211.91	386.178	23
-6	4	-3	2516.47	380.891	23
6	-4	3	1404.93	349.172	2
6	4	3	1998.41	184.425	19
6	4	3	1892.77	202.426	18
6	-4	3	1732.31	182.240	26
6	-4	3	2061.45	193.651	6
6	4	3	2000.46	213.394	29
6	-4	3	1854.62	191.341	14
6	-4	4	1531.30	374.087	2
6	4	4	1424.31	174.408	18
-6	4	-4	1311.85	282.263	23
-6	-4	-4	1583.18	340.087	23
-6	-4	-4	1357.98	171.069	22
6	-4	4	1724.38	181.021	6
6	-4	4	1503.95	169.945	7
6	4	4	1476.13	330.560	25

6	-4	4	1514.63	312.768	25
6	4	4	1456.63	186.475	29
6	4	5	9909.02	941.394	25
6	-4	5	11086.5	937.486	25
-6	-4	-5	9473.57	903.109	23
-6	-4	-5	10174.9	581.889	22
6	-4	5	9071.14	968.894	2
6	4	5	9449.87	558.208	18
6	-4	5	9928.95	578.596	5
6	-4	5	10451.9	569.817	7
6	4	5	7727.52	566.592	29
6	4	6	261.016	175.691	25
-6	-4	-6	59.5457	148.518	23
6	4	6	138.603	83.1264	29
6	-4	6	122.210	81.2232	29
6	-4	6	231.092	191.968	2
6	-4	6	287.946	86.8092	7
6	-4	6	334.042	98.4511	5
6	4	6	210.382	75.0076	18
6	-4	7	1734.63	178.129	5
6	-4	7	1879.48	171.983	7
6	4	7	667.907	140.382	29
6	-4	7	248.119	102.160	29
6	4	7	1608.75	155.326	18
6	-4	8	233.718	82.4817	5
6	-4	8	231.191	75.2908	7
6	4	8	-14.8937	99.4167	29
6	4	8	168.983	57.8975	18
-6	4	-9	729.552	83.8580	20

6	-4	9	745.590	105.795	5
6	-4	9	741.507	94.9790	7
-6	-4	-10	59.4694	43.2241	20
-6	-4	-10	91.9014	59.3163	21
6	-4	10	18.7091	48.5301	4
6	-4	10	49.9699	40.7142	5
6	-4	11	929.980	89.9220	4
6	-5	-13	668.279	88.2846	11
-6	-5	13	705.206	98.1707	22
6	5	-13	738.189	95.3588	19
-6	-5	12	36.6691	68.0337	22
-6	5	12	31.2985	43.5571	29
6	-5	-12	-10.6248	57.1348	11
6	5	-12	10.9288	59.8089	19
6	5	-11	4305.88	307.669	20
6	-5	-11	4550.00	348.132	11
-6	5	11	4773.52	331.376	29
6	5	-11	5571.32	373.228	19
6	5	-10	143.711	68.8364	20
6	-5	-10	207.549	68.9317	20
-6	5	10	103.611	77.1898	29
6	-5	-10	337.507	108.649	11
6	5	-9	5199.44	360.134	20
6	-5	-9	4833.84	351.240	20
6	-5	-9	5408.97	407.495	11
6	-5	-8	21372.8	1146.61	11
6	-5	-8	19676.4	1076.97	20
6	5	-8	19934.2	1138.75	19
-6	-5	8	21351.4	1099.44	27

6	-5	-8	23298.2	1464.74	1
6	5	-7	8269.02	562.440	19
-6	5	7	7041.56	731.968	30
6	-5	-7	8431.93	866.972	17
-6	-5	7	9059.30	541.865	27
6	-5	-7	8464.29	825.978	1
6	-5	-6	1202.49	178.177	3
6	-5	-6	1045.21	160.664	11
6	5	-6	945.461	157.286	19
-6	5	6	1152.49	276.252	30
6	-5	-6	1009.67	284.458	17
-6	-5	6	1498.67	170.052	27
6	-5	-6	1266.99	306.283	1
6	-5	-5	2091.75	218.924	11
6	5	-5	2151.64	224.189	19
-6	5	5	1416.80	339.952	30
6	-5	-5	2021.44	376.651	17
6	-5	-5	2313.72	241.585	3
6	-5	-5	2949.88	456.432	1
6	-5	-4	17699.2	930.953	14
6	-5	-4	17260.9	952.368	3
6	-5	-4	18532.6	948.599	11
6	5	-4	17277.8	947.636	19
6	-5	-4	15976.0	1222.92	17
6	-5	-4	18928.3	1330.74	1
-6	-5	4	17815.8	925.690	28
6	-5	-3	9934.86	419.622	14
6	-5	-3	10290.6	451.453	3
6	-5	-3	9904.40	430.059	11

6	5	-3	9043.10	427.129	19
-6	-5	3	9839.25	416.291	28
6	-5	-2	9992.83	571.373	11
6	5	-2	9214.43	568.928	19
6	-5	-2	9467.87	894.872	2
-6	-5	2	8897.69	578.077	22
6	5	-2	9056.50	725.667	25
-6	-5	2	9355.79	562.289	28
-6	-5	1	6628.61	464.724	22
6	5	-1	7297.04	460.095	19
6	-5	-1	7585.16	456.562	14
6	-5	-1	6952.02	757.165	2
6	-5	-1	8228.12	776.541	1
6	5	-1	6446.50	605.724	25
6	-5	-1	6904.58	595.043	25
-6	-5	0	33975.0	1805.17	22
-6	-5	0	32232.7	2106.61	23
6	-5	0	36519.8	1791.15	14
6	5	0	35513.2	1791.13	19
6	-5	0	34956.6	1772.81	7
6	-5	0	38278.5	2295.63	2
6	5	0	34270.4	2042.99	25
6	-5	0	32878.2	1976.57	25
6	-5	1	530.465	96.5301	14
6	5	1	431.105	83.1870	26
6	-5	1	447.364	145.532	25
6	5	1	124.621	105.277	25
-6	-5	-1	255.539	131.335	23
-6	-5	-1	381.488	98.9173	22

6	-5	1	456.042	87.1408	5
6	-5	1	399.203	92.8454	4
6	5	1	510.224	95.6750	19
6	-5	1	417.876	82.6344	6
6	-5	1	450.942	95.5836	7
6	-5	1	320.890	181.327	1
6	5	1	514.504	109.420	18
6	-5	2	6237.81	399.306	6
6	-5	2	6030.12	401.509	7
6	5	2	5754.16	623.602	25
-6	-5	-2	6014.12	419.032	22
-6	-5	-2	5998.84	630.963	23
6	-5	2	5894.35	423.681	4
6	-5	2	5546.94	394.227	5
6	5	2	6345.90	425.295	18
6	5	2	5991.10	392.047	19
6	5	2	6405.01	400.438	26
6	-5	2	5399.15	659.123	1
6	-5	2	5778.06	720.065	2
6	-5	2	6110.91	407.405	14
6	5	3	1116.14	156.615	18
6	-5	3	1464.39	161.523	6
6	-5	3	1422.17	164.582	7
-6	-5	-3	1484.66	174.212	22
-6	-5	-3	836.711	236.964	23
6	-5	3	1208.27	168.943	4
6	-5	3	1155.24	154.502	5
6	5	3	1213.99	140.072	19
6	5	3	1217.76	284.106	25

6	5	3	1355.97	157.005	26
6	-5	3	1443.25	165.438	14
6	-5	3	1077.14	331.832	2
6	5	4	18786.4	1310.93	25
-6	-5	-4	16427.6	1251.30	23
-6	-5	-4	20143.9	945.501	22
6	5	4	19425.9	930.812	18
6	-5	4	19565.4	929.999	5
6	-5	4	22181.3	1562.83	2
-6	-5	-5	54.4812	146.469	23
-6	-5	-5	89.3771	78.6526	22
6	5	5	-25.6137	70.0808	18
6	-5	5	13.0368	67.5485	7
6	-5	5	-78.1782	137.593	2
6	5	5	15.3890	71.6601	29
6	5	6	1579.69	158.166	18
6	-5	6	1464.97	162.572	7
6	-5	6	1686.60	176.249	5
6	5	7	690.284	98.8207	18
6	-5	7	795.043	121.893	7
6	-5	7	861.531	134.855	5
6	-5	8	478.602	90.4401	7
6	-5	8	529.320	101.353	5
6	-5	9	5129.43	337.231	4
6	-5	9	4248.95	286.898	7
6	-5	9	4524.14	299.756	5
6	-5	10	15.3094	42.6221	4
6	-5	10	36.7349	38.1031	5
6	6	-13	27.5433	33.1493	19

6	-6	-13	36.8743	35.1323	11
-6	-6	12	-8.96528	56.1640	22
6	-6	-12	-26.8663	55.3595	11
-6	-6	11	2879.68	249.981	22
6	-6	-11	2955.89	248.751	11
6	6	-10	2779.90	272.895	19
-6	-6	10	3253.19	287.608	22
6	-6	-10	3436.57	290.031	11
6	6	-9	2641.50	265.864	19
-6	-6	9	2581.50	268.560	22
-6	-6	8	1275.22	166.847	27
6	6	-8	1584.40	202.935	19
-6	-6	8	1586.01	209.139	22
-6	-6	7	969.518	169.265	22
6	6	-7	843.091	155.293	19
-6	-6	7	1235.42	165.548	27
6	-6	-7	1241.46	285.171	1
6	-6	-7	1069.67	167.517	11
6	-6	-6	5185.20	403.528	11
-6	-6	6	6268.57	444.430	22
6	6	-6	5198.83	409.173	19
6	-6	-6	5148.10	372.665	14
-6	-6	6	5766.37	404.653	27
6	-6	-6	5730.55	431.395	3
6	-6	-6	5617.38	642.801	1
-6	-6	5	257.314	99.1322	22
6	6	-5	108.839	69.6207	19
6	-6	-5	224.713	72.8267	14
6	-6	-5	169.438	140.947	1

6	-6	-5	271.349	102.462	3
6	-6	-5	364.262	101.646	11
6	-6	-4	42.7093	61.3746	11
-6	-6	4	49.2889	70.8295	22
6	6	-4	-58.9563	66.9786	19
-6	-6	4	84.2118	66.8935	28
6	-6	-4	-11.6490	51.5960	14
6	-6	-4	-13.9285	54.8153	4
6	-6	-4	16.1498	73.3894	3
6	-6	-4	-115.328	131.020	1
-6	-6	3	2659.25	242.966	28
6	-6	-3	2311.82	228.101	11
6	6	-3	2541.85	240.342	19
-6	-6	3	2517.69	249.600	22
6	-6	-3	2931.78	232.535	14
6	-6	-3	2541.64	251.630	3
6	-6	-3	2458.53	235.317	4
6	-6	-3	2855.19	459.331	1
-6	-6	2	13763.4	806.531	28
-6	-6	2	14040.4	822.638	22
6	-6	-2	14795.1	1236.82	2
6	6	-2	13588.0	798.250	19
6	-6	-2	15528.1	805.371	11
6	-6	-2	14880.1	790.524	14
6	-6	-2	13423.2	809.040	3
6	-6	-2	13027.3	797.297	4
6	-6	-1	283.181	74.0179	14
6	-6	-1	205.500	78.6582	3
6	-6	-1	237.158	159.958	1

6	6	-1	277.251	80.0677	19
6	-6	-1	310.565	78.6781	11
6	-6	-1	317.970	74.4866	7
6	-6	-1	265.503	88.5495	4
-6	-6	1	302.257	88.7691	28
6	-6	0	12734.9	754.394	4
6	6	0	12648.3	726.883	19
6	-6	0	11857.3	706.238	5
6	-6	0	13191.4	714.806	7
6	-6	0	13844.4	1201.45	2
6	-6	0	15780.2	1194.52	1
6	-6	0	12292.2	714.291	11
6	-6	0	13641.4	729.801	14
6	-6	1	184.241	66.8862	5
6	-6	1	349.773	100.607	4
6	6	1	145.987	62.1990	19
6	6	1	147.940	77.2540	18
6	-6	1	247.926	78.2597	7
6	-6	1	60.1882	137.094	1
6	-6	1	359.263	219.246	2
6	-6	1	282.553	79.0347	14
6	-6	2	19386.4	1066.61	4
6	-6	2	18536.1	1026.34	5
6	6	2	19390.0	1053.10	18
6	-6	2	19143.7	1028.82	14
6	-6	2	18495.5	1023.89	7
6	-6	2	20193.0	1585.70	2
6	-6	3	1081.35	143.949	14
6	-6	3	1056.31	145.787	10

-6	-6	-3	971.512	152.192	22
6	6	3	1072.11	150.730	18
6	-6	3	949.246	144.310	8
6	-6	3	908.053	138.063	7
6	-6	3	1245.51	356.803	2
6	-6	3	955.240	141.827	5
6	-6	3	897.654	153.290	4
-6	-6	-4	15255.6	804.362	22
6	-6	4	14612.3	1313.31	2
6	6	4	13862.1	771.656	18
6	-6	4	11310.7	766.569	8
6	-6	4	13722.5	812.883	4
6	-6	4	13074.0	772.862	5
6	6	5	462.766	97.0201	18
6	-6	5	443.664	113.865	8
6	-6	5	584.821	115.219	7
-6	-6	-5	488.056	118.725	22
6	-6	5	529.062	126.328	4
6	-6	5	478.579	244.267	2
6	-6	6	457.767	109.197	5
6	-6	6	457.491	121.391	4
6	6	6	495.109	90.5601	18
6	-6	6	490.834	108.169	7
6	-6	7	15.2542	71.0505	4
6	-6	7	24.2968	69.8503	5
6	-6	7	-86.2592	68.6035	8
6	-6	7	10.9601	65.9079	7
6	-6	8	505.916	109.030	4
6	-6	8	603.118	103.008	5

6 -6 8 366.967 83.3221 7

6 -6 9 41.0889 47.2647 10

6 -6 9 55.9368 55.2616 4

6 -6 9 14.5818 45.0936 5

6 -6 10-5.33115 29.6792 4

6 -6 10 35.0811 25.4667 5

6 7 -12-6.82856 39.2567 19

6 -7 -12-19.7349 40.1117 11

6 7 -11 160.313 67.0063 19

-6 -7 11 96.6906 53.8946 22

6 7 -10 151.884 83.6386 19

-6 -7 10 144.409 73.6974 22

6 7 -9 72.8248 76.9095 19

-6 -7 9 14.4732 68.9883 22

6 -7 -9-14.4036 73.1867 11

6 7 -8 2578.75 249.306 19

-6 -7 8 2559.75 252.037 22

6 -7 -8 2237.56 237.132 11

-6 -7 8 1952.20 216.179 27

6 7 -7 564.554 134.999 19

-6 -7 7 411.570 110.810 27

-6 -7 7 610.277 135.164 22

6 -7 -7 904.839 146.895 11

6 7 -6 527.417 121.892 19

6 -7 -6 622.426 137.256 3

6 -7 -6 533.172 204.870 1

-6 -7 6 667.080 132.474 27

-6 -7 6 619.880 133.104 22

6 -7 -6 782.007 135.302 11

6	-7	-5	4226.31	339.726	11
6	7	-5	4028.87	337.315	19
6	-7	-5	4500.49	364.859	3
-6	-7	5	4450.43	351.208	27
6	-7	-5	4360.61	574.512	1
-6	-7	5	4412.68	358.791	22
6	-7	-4	13143.6	768.958	11
6	7	-4	13548.2	778.876	19
6	-7	-4	14337.2	810.469	3
6	-7	-4	11818.0	769.198	4
-6	-7	4	13034.6	781.948	28
6	-7	-4	15127.6	1192.01	1
-6	-7	4	13392.2	799.153	22
-6	-7	3	147.218	94.1344	22
6	7	-3	84.3695	68.6752	19
6	-7	-3	-63.2432	157.478	1
6	-7	-3	66.3697	79.2111	3
-6	-7	3	188.073	82.8763	28
6	-7	-3	155.632	82.2989	4
6	-7	-3	92.4838	67.2791	11
6	-7	-2	30957.8	1540.10	14
6	-7	-2	29652.6	1552.39	11
6	7	-2	30172.6	1568.21	19
-6	-7	2	29664.2	1590.16	28
6	-7	-2	29193.2	1593.88	3
6	-7	-2	30193.6	1591.10	4
-6	-7	2	30332.2	1595.26	22
-6	-7	1	3205.37	282.283	22
6	-7	-1	2190.48	466.121	2

6	7	-1	3102.90	254.055	19
-6	-7	1	3129.94	281.129	28
6	-7	-1	2399.27	254.398	4
6	-7	-1	2838.37	268.157	3
6	-7	-1	3186.19	243.953	14
-6	-7	0	13763.4	782.813	22
6	-7	0	14045.3	1248.07	2
6	7	0	13095.4	734.143	19
-6	-7	0	14008.8	794.701	28
6	-7	0	12348.9	718.262	7
6	-7	0	11865.1	725.045	5
6	-7	0	14280.0	739.175	14
6	-7	0	13293.6	783.495	4
6	-7	0	12245.9	752.577	3
-6	-7	-1	8528.88	536.391	22
6	-7	1	7777.23	512.318	8
6	7	1	8157.79	533.285	18
6	-7	1	8016.50	540.380	4
6	-7	1	7252.06	490.367	5
6	-7	1	7376.16	485.432	7
6	-7	1	9182.17	507.072	14
6	-7	1	7337.79	894.199	2
-6	-7	-2	12565.2	801.332	22
6	-7	2	13085.2	796.015	8
6	7	2	13933.5	806.461	18
6	-7	2	14215.3	832.535	4
6	-7	2	14787.0	789.262	7
6	-7	2	13352.5	787.055	5
6	-7	2	14909.9	787.011	14

6	-7	2	14619.9	1317.80	2
6	7	3	10651.9	630.913	18
6	-7	3	10171.9	662.422	4
6	-7	3	10868.6	626.494	7
6	-7	3	10752.4	637.610	5
6	-7	3	10924.1	1119.14	2
6	-7	3	9627.64	616.509	10
6	-7	3	10187.8	635.827	8
6	-7	4	4612.95	350.930	5
6	-7	4	4801.82	343.635	7
6	-7	4	4949.12	384.853	4
6	-7	4	4471.26	344.232	10
6	-7	4	4184.88	346.613	8
6	7	4	4914.08	340.854	18
6	-7	5	1082.86	153.811	8
6	-7	5	1278.07	182.054	4
6	-7	5	1219.43	158.614	10
6	7	5	1346.08	144.992	18
6	-7	6	347.361	92.6931	8
6	-7	6	307.459	92.7625	7
6	-7	6	231.080	98.6232	4
6	-7	6	276.998	90.5007	5
6	-7	6	228.197	83.5090	10
6	-7	7	78.1114	61.2650	7
6	-7	7	131.434	80.2127	4
6	-7	7	65.2212	64.5060	5
6	-7	7	54.5871	58.8111	10
6	-7	8	2444.25	200.630	5
6	-7	8	2474.36	197.774	10

6	-7	8	2734.76	218.557	4
6	-7	9	226.462	54.5467	4
6	-7	9	267.515	53.8987	5
6	8	-12	2544.12	153.919	19
6	8	-11	253.153	61.2952	19
6	-8	-11	236.437	57.3946	11
6	8	-10	-9.55303	54.9135	19
6	-8	-10	-9.25598	54.0310	11
-6	-8	9	2434.60	213.883	22
6	8	-9	2589.99	226.316	19
6	-8	-9	2528.64	223.632	11
-6	-8	8	12.9678	67.1923	27
6	-8	-8	-25.7990	65.5407	11
6	8	-8	-26.3716	70.9006	19
-6	-8	8	-12.7505	66.0667	22
-6	-8	7	-29.2770	66.5394	27
6	-8	-7	54.7406	66.7084	11
6	8	-7	70.3443	70.0521	19
-6	-8	7	57.2377	68.2174	22
6	8	-6	11366.4	690.694	19
-6	-8	6	11109.3	695.480	22
6	-8	-6	11708.6	717.372	3
-6	-8	6	12505.9	719.117	27
6	-8	-6	11425.6	688.637	11
6	8	-5	14.1816	67.7229	19
6	-8	-5	148.907	75.4603	11
6	-8	-5	155.508	97.8470	3
-6	-8	5	49.4915	76.9355	27
-6	-8	5	204.228	95.8728	22

6	8	-4	11025.9	669.265	19
-6	-8	4	11969.4	699.380	22
6	-8	-4	11679.4	712.098	3
6	-8	-4	10841.9	691.370	4
6	-8	-4	11054.3	662.649	11
-6	-8	4	11305.9	699.331	28
-6	-8	3	299.593	102.417	22
6	8	-3	375.548	92.3693	19
6	-8	-3	235.890	73.4268	11
6	-8	-3	279.763	100.038	4
6	-8	-3	338.827	115.151	3
-6	-8	3	482.386	118.041	28
-6	-8	2	2105.03	223.913	22
-6	-8	2	2200.52	236.788	28
6	8	-2	1656.88	182.447	19
6	-8	-2	2095.31	208.533	8
6	-8	-2	2100.11	227.292	3
6	-8	-2	1886.02	222.958	4
-6	-8	1	305.842	110.977	28
-6	-8	1	503.263	116.618	22
-6	-8	1	487.047	109.944	16
6	-8	-1	386.001	97.0496	8
6	-8	-1	640.948	126.637	3
6	-8	-1	287.419	104.963	4
-6	-8	0	17075.6	918.999	22
6	-8	0	15870.8	900.470	8
6	8	0	16746.7	922.339	18
-6	-8	0	16345.2	904.734	16
6	-8	0	15352.5	855.342	7

6	-8	0	14940.3	868.506	5
-6	-8	0	16796.1	940.641	28
6	-8	0	15287.7	897.492	3
6	-8	0	16761.6	939.553	4
-6	-8	-1	7660.99	480.869	22
6	8	1	7131.80	473.929	18
6	-8	1	6531.41	459.095	8
6	-8	1	6695.31	431.561	7
6	-8	1	6452.73	445.427	5
-6	-8	-1	6941.41	488.910	28
6	-8	1	6871.02	493.113	4
6	-8	2	21360.3	1206.33	8
6	8	2	23506.2	1212.66	18
-6	-8	-2	22623.2	1215.14	22
6	-8	2	23977.3	1254.03	4
6	-8	2	21616.7	1182.82	7
6	-8	2	22690.0	1204.60	5
-6	-8	-3	-27.6768	74.4088	22
6	8	3	71.7959	73.9620	18
6	-8	3	11.8869	68.3287	7
6	-8	3	-41.8837	63.4457	8
6	-8	3	68.2572	84.9475	4
6	-8	3	80.9126	72.5110	5
6	-8	3	11.7978	52.2574	10
6	8	4	5982.98	376.310	18
6	-8	4	6131.96	433.834	4
6	-8	4	6682.70	405.762	7
6	-8	4	6640.89	419.609	5
-6	-8	-4	6298.89	411.833	22

6	-8	4	4959.81	388.892	8
-6	-8	-4	5162.31	370.273	9
6	-8	4	5300.34	383.269	10
6	-8	5	1341.73	152.901	7
6	-8	5	1409.17	168.103	5
6	-8	5	1452.45	181.153	4
6	-8	5	1439.65	160.141	8
6	8	5	1077.93	117.139	18
6	-8	6	90.8531	71.1499	4
6	-8	7	454.449	98.3812	4
6	-8	7	631.446	100.601	5
6	-8	8	2526.27	187.303	4
6	-8	8	2393.84	177.658	5
6	-9	-10	35.3565	32.4785	11
6	9	-10	10.8759	32.7085	19
6	-9	-9	138.811	53.1240	11
6	9	-9	30.9387	41.7263	19
-6	-9	8	956.510	128.196	27
6	9	-8	1017.34	126.650	19
6	-9	-8	1019.13	122.796	11
-6	-9	7	3299.39	265.552	27
-6	-9	7	3203.23	244.284	22
6	9	-7	2915.01	241.433	19
6	-9	-7	2988.70	239.509	11
-6	-9	6	1947.72	207.767	27
-6	-9	6	1793.14	185.358	22
6	-9	-6	1970.47	181.721	11
6	9	-6	1683.10	176.670	19
6	9	-5	-22.4217	59.1949	19

6	-9	-5	71.4009	61.3200	11
6	-9	-5	77.5441	77.2044	3
-6	-9	5	-15.2968	77.7192	27
-6	-9	5	-12.6523	58.9173	22
6	-9	-4	5468.59	424.574	3
-6	-9	4	6749.44	446.743	27
-6	-9	4	6111.72	411.352	22
6	9	-4	5601.85	383.611	19
6	-9	-3	429.237	112.269	4
-6	-9	3	404.837	117.802	28
6	-9	-3	443.772	117.584	3
-6	-9	3	324.564	94.0046	22
-6	-9	2	6201.81	411.088	22
-6	-9	2	5157.89	401.961	16
6	-9	-2	5637.77	396.555	8
-6	-9	2	5653.71	423.455	28
6	-9	-2	6269.97	430.153	3
6	-9	-2	5858.63	428.624	4
-6	-9	1	6423.79	433.365	22
6	-9	-1	5924.28	423.821	8
-6	-9	1	5724.30	429.428	16
-6	-9	1	6680.83	464.113	28
6	-9	-1	7143.24	456.421	3
6	-9	-1	6754.70	467.752	4
-6	-9	0	8333.77	500.499	22
6	-9	0	7471.24	489.355	8
6	9	0	7656.13	498.615	18
-6	-9	0	7461.62	488.710	16
6	-9	0	7180.05	460.964	5

-6	-9	0	7285.82	509.827	28
6	-9	0	7352.68	484.102	3
6	-9	0	8120.48	528.727	4
-6	-9	-1	2173.24	209.885	22
6	-9	1	1762.43	196.056	8
6	9	1	2274.43	211.480	18
-6	-9	-1	2137.31	206.058	16
6	-9	1	1742.25	187.161	5
-6	-9	-1	2259.97	236.058	28
6	-9	1	2020.64	225.496	4
-6	-9	-2	6107.38	410.917	22
-6	-9	-2	6119.52	439.604	28
6	9	2	6075.64	400.264	18
6	-9	2	5678.60	408.759	8
6	-9	2	6362.70	450.703	4
6	-9	2	6520.93	415.364	5
-6	-9	-3	833.524	125.324	22
6	9	3	694.798	102.871	18
-6	-9	-3	670.068	125.810	28
-6	-9	-3	737.897	118.113	9
6	-9	3	847.920	145.123	4
6	-9	3	654.119	115.949	5
-6	-9	-4	1011.91	145.521	28
-6	-9	-4	734.056	107.775	9
6	-9	4	1161.24	157.154	4
6	-9	4	1150.63	143.287	5
-6	-9	-5	2186.33	208.147	28
6	-9	5	2511.36	223.389	4
6	-9	5	2371.05	204.357	5

6	-9	6	1003.05	123.769	4
-6	-9	-6	857.180	110.363	28
6	-9	7	188.279	56.8451	4
6	-9	7	139.345	47.4855	5
-6	-9	-7	160.763	35.8256	15
6	10	-9	150.530	37.0541	18
6	10	-8	11.6321	22.2943	19
-6	-10	7	19.1664	46.7108	27
6	10	-7	21.2242	32.8102	19
-6	-10	6	364.513	89.0540	27
-6	-10	5	139.252	73.9244	27
6	-10	-5	-24.6981	69.8967	3
-6	-10	4	-24.8635	64.4168	16
6	-10	-4	64.9924	79.2526	3
-6	-10	4	27.1273	64.6499	27
-6	-10	3	9.76870	53.4567	3
6	-10	-3	13.1526	70.7214	3
-6	-10	3	-28.3283	70.5108	27
-6	-10	3	-38.6544	57.2589	22
-6	-10	3	25.2205	64.0699	16
-6	-10	2	371.299	83.6908	22
-6	-10	2	243.524	89.9165	28
-6	-10	2	273.440	97.1659	27
-6	-10	2	281.680	72.7034	3
6	-10	-2	421.563	103.404	3
6	10	-2	323.388	93.3354	18
-6	-10	2	446.792	98.1268	16
6	-10	-2	366.649	104.512	4
6	10	-1	9301.95	548.010	18

6	-10	-1	9084.46	544.971	3
6	-10	-1	9124.02	568.265	4
-6	-10	1	9084.08	569.468	28
-6	-10	1	8067.63	535.217	16
-6	-10	1	9176.52	529.285	22
6	10	0	1127.66	141.177	18
6	-10	0	1551.58	179.120	4
-6	-10	0	1268.23	164.315	28
-6	-10	0	1099.89	142.861	16
-6	-10	0	1308.76	143.996	22
-6	-10	-1	1545.39	177.104	28
6	-10	1	1367.39	174.964	4
6	10	1	1376.52	147.564	18
-6	-10	-1	1500.81	153.408	22
6	-10	2	547.487	113.717	4
-6	-10	-2	288.130	77.4577	22
-6	-10	-2	622.374	110.913	28
6	10	2	533.748	81.6096	18
6	-10	3	426.417	94.1191	4
-6	-10	-3	332.422	87.3402	28
-6	-10	-3	174.313	64.4235	9
6	-10	4	224.964	76.0401	4
-6	-10	-4	284.873	72.3944	28
-6	-10	-4	103.953	43.4187	9
6	-10	5	388.562	78.4408	4
-6	-10	-6	2140.41	146.628	15
6	-10	6	2759.28	170.688	4
-6	-11	7	114.747	38.4055	16
-6	-11	7	863.578	70.3005	3

6	11	-6	67.2014	47.0619	18
-6	-11	6	76.3300	42.1647	16
-6	-11	6	172.764	47.9598	3
-6	-11	5	-31.1859	39.5821	3
-6	-11	5	-35.4769	44.1534	27
6	11	-5	-15.7556	49.3474	18
-6	-11	5	61.0293	46.5125	16
6	11	-4	1008.51	118.732	18
-6	-11	4	721.831	104.022	16
-6	-11	4	903.546	115.673	27
6	-11	-4	1143.26	127.136	3
-6	-11	4	798.190	95.1952	3
-6	-11	3	104.582	41.3908	3
6	-11	-3	99.5004	63.3308	3
-6	-11	3	197.328	67.0444	27
6	11	-3	192.074	70.1408	18
-6	-11	3	139.536	58.4541	16
-6	-11	2	1349.81	141.235	16
6	-11	-2	1584.26	149.934	3
6	11	-2	1484.06	145.753	18
-6	-11	1	-16.0682	54.1521	16
6	11	-1	-7.77066	48.0300	18
6	-11	-1	-21.0113	57.4877	4
-6	-11	1	10.7682	47.6949	28
-6	-11	1	10.4445	53.0651	27
6	11	0	3439.96	234.183	18
6	-11	0	3693.07	271.966	4
-6	-11	0	3500.56	261.966	27
-6	-11	0	3824.46	272.217	28

-6	-11	-1	2581.77	192.428	27
-6	-11	-1	2824.23	204.069	28
6	-11	1	2987.59	210.407	4
6	11	1	2370.45	151.835	18
6	-11	2	909.298	108.580	4
-6	-11	-2	948.482	104.892	28
6	-11	3	922.723	94.9113	4
-6	-11	-3	124.946	38.9591	9
6	-11	4	583.861	71.0640	4
6	12	-4	92.5327	28.2316	18
6	12	-3	20.8787	23.4751	18
-6	-12	2	-4.87958	30.9654	9
-6	-12	1	-14.5455	31.5538	9
-6	-12	0	17.0110	27.6816	9
7	0	-14	7.35813	42.9539	20
-7	0	14	13.3607	38.9979	29
7	0	-13	197.981	79.7658	20
-7	0	13	260.563	78.5732	29
7	0	-12	-28.3057	81.3583	20
-7	0	12	85.9344	77.0160	29
7	0	-11	8006.44	540.585	20
7	0	-10	-79.7185	181.143	17
7	0	-10	-17.6164	96.4201	20
7	0	-9	68078.0	3442.98	20
7	0	-9	69559.7	3398.67	11
7	0	-9	70305.3	4076.61	17
7	0	-9	69474.2	3396.50	19
7	0	-8	-16.0589	83.2079	20
7	0	-8	63.7863	57.9764	19

7	0	-8	12.9256	57.2539	11
7	0	-8	-72.8333	128.188	17
7	0	-7	3166.91	261.468	19
7	0	-7	3086.98	262.703	11
7	0	-6	-60.9651	123.898	17
7	0	-6	24.0941	51.9393	11
7	0	-5	22330.1	1558.47	17
7	0	-4	51.7648	117.629	17
7	0	-4	52.2667	56.2316	26
7	0	-4	-42.2611	105.202	25
7	0	-3	147.857	123.587	17
7	0	-3	366.867	81.1062	26
7	0	-3	425.119	167.976	25
7	0	-2	48.4564	110.101	17
7	0	-2	21.3898	49.8037	26
7	0	-2	-43.3268	139.222	25
7	0	-1	12242.3	720.180	26
7	0	-1	14771.9	1043.77	25
7	0	-1	13840.7	1002.14	1
7	0	0	48.4132	110.002	25
7	0	0	47.4915	60.3224	26
7	0	0	-36.7784	91.5416	1
7	0	1	4886.31	354.786	26
7	0	1	4661.32	581.328	25
7	0	1	5430.49	507.100	1
-7	0	-2	-63.2588	157.452	23
7	0	2	24.7722	56.2892	18
7	0	2	-21.5074	54.6394	13
7	0	3	3468.69	255.360	14

7	0	3	2970.36	261.205	13
-7	0	-3	3533.40	543.632	23
-7	0	-4	60.2277	149.907	23
7	0	4	23.3502	59.3173	6
7	0	4	-33.0234	50.0228	14
7	0	4	13.7208	65.3945	18
-7	0	-5	30151.5	1922.10	23
7	0	5	25994.2	1409.50	18
7	0	5	26945.0	1392.16	6
-7	0	-6	-64.5879	185.632	23
7	0	6	54.4555	59.6083	7
7	0	6	-23.2541	63.6345	6
7	0	6	137.310	88.2514	18
7	0	7	46.2492	54.0008	7
7	0	7	-12.7428	66.0297	18
7	0	9	185.154	64.9959	5
7	0	10	-4.55966	31.4245	5
-7	0	-10	26.7996	44.1211	20
-7	0	-11	2421.62	154.186	20
7	1	-14	111.850	50.0014	20
7	1	-13	16286.9	847.525	20
-7	1	13	17024.6	847.292	29
7	1	-12	413.358	109.888	20
-7	-1	12	284.514	97.9826	29
-7	1	12	465.120	115.049	29
7	1	-11	2714.56	241.297	19
7	-1	-11	3063.28	249.356	11
7	1	-11	3556.45	298.500	20
7	1	-10	68.4754	85.2243	20

7	-1	-10	34.7697	84.7265	20
7	1	-10	67.8670	64.7007	19
-7	-1	10	122.341	138.991	30
-7	1	10	62.2465	126.506	30
7	-1	-10	-81.9092	166.464	17
7	1	-10	75.5131	132.906	17
7	-1	-10	27.2747	60.4121	11
7	-1	-10	-60.2021	136.791	1
7	1	-9	6181.10	458.885	20
7	-1	-9	6592.47	464.694	20
7	1	-9	6376.26	442.435	19
-7	-1	9	6892.42	739.132	30
-7	1	9	6525.92	741.548	30
7	1	-9	6514.07	809.712	17
7	-1	-9	6932.15	854.860	17
7	-1	-9	6034.60	432.547	11
7	-1	-9	5570.44	737.654	1
7	1	-8	14.3861	62.0247	19
-7	-1	8	-55.4884	126.080	30
-7	1	8	-56.2068	151.110	30
7	-1	-8	74.4550	131.042	17
7	1	-8	70.6189	160.458	17
7	1	-8	47.8088	66.7716	20
7	-1	-8	-15.9912	74.4673	20
7	-1	-8	57.3554	66.7736	11
7	1	-7	4018.93	319.448	19
-7	-1	7	4838.30	579.738	30
-7	1	7	5267.92	613.146	30
7	1	-7	5851.36	681.740	17

7	1	-7	4048.16	325.957	20
7	-1	-7	3886.95	326.029	20
7	-1	-7	3778.61	312.854	11
7	1	-6	6487.05	426.720	19
7	1	-6	7131.59	750.425	17
7	-1	-6	5695.36	411.730	11
7	1	-6	6190.04	426.340	20
7	-1	-6	5659.63	416.548	20
-7	-1	6	8660.05	808.417	30
-7	1	6	8197.96	776.905	30
7	1	-5	71996.6	3626.45	17
7	1	-5	61824.6	3122.58	20
7	1	-4	306.983	188.189	17
7	1	-4	367.498	81.5631	26
7	1	-4	339.332	143.917	25
7	1	-3	49.2111	111.886	17
7	-1	-3	52.1677	118.600	1
7	1	-3	84.3724	53.7329	26
7	-1	-3	104.003	58.9765	26
7	1	-3	85.4900	114.987	25
7	1	-2	2475.90	453.890	17
7	-1	-2	3181.46	447.472	1
7	-1	-2	2868.37	234.267	26
7	1	-2	3017.05	238.498	26
7	1	-2	2704.77	405.036	25
7	-1	-1	-42.7793	115.011	1
7	-1	-1	84.1090	75.3768	2
7	1	-1	11.2533	51.1426	26
7	-1	-1	-44.1833	56.1242	26

7	1	-1	-45.6666	103.763	25
7	-1	-1	-44.8784	101.972	25
7	1	0	7114.40	446.714	26
7	-1	0	6739.66	440.836	26
7	-1	0	6905.16	588.871	2
7	1	0	6087.29	590.903	1
7	-1	0	7403.12	654.085	1
7	1	0	6882.81	696.193	25
7	-1	0	7695.86	721.811	25
7	1	1	7412.61	460.059	26
7	-1	1	6557.61	444.880	26
7	1	1	7815.60	752.502	25
7	-1	1	6881.50	711.589	25
7	-1	1	6529.10	605.376	2
7	-1	1	6531.43	612.780	1
-7	1	-2	8550.97	906.527	23
7	-1	2	7616.57	690.594	2
7	-1	2	8015.82	658.179	1
7	1	2	7911.10	503.341	18
7	-1	2	7766.60	496.560	18
7	1	2	7969.10	495.814	19
7	1	2	6636.47	757.225	25
7	-1	2	9551.92	860.490	25
7	1	2	9678.33	552.137	29
7	1	2	7805.20	479.175	13
7	-1	2	7926.58	488.335	13
7	-1	2	7449.24	479.527	14
7	-1	3	1046.14	174.564	13
7	1	3	1441.10	153.665	14

7	-1	3	2040.85	185.840	14
7	1	3	1771.91	179.781	13
-7	1	-3	2042.60	396.962	23
7	1	3	2136.59	196.684	19
7	-1	3	1623.77	180.553	18
7	1	3	1882.46	215.403	29
7	-1	3	1955.70	213.754	29
7	-1	3	1940.17	185.177	6
7	1	3	1463.72	351.359	25
7	-1	3	1366.77	337.583	25
7	-1	3	1597.48	306.248	2
7	1	4	1753.80	405.802	25
7	-1	4	1089.75	320.856	25
-7	-1	-4	2557.22	454.085	23
-7	1	-4	1845.56	382.384	23
7	-1	4	1318.95	294.731	2
7	1	4	1755.32	195.994	18
7	-1	4	1691.31	180.782	6
7	1	4	1892.54	181.312	6
7	-1	4	2094.28	232.800	29
7	1	4	1796.19	226.003	29
7	-1	4	1921.28	188.564	14
7	1	4	1819.43	172.726	14
-7	1	-5	249.456	167.786	23
-7	-1	-5	127.928	183.949	23
7	-1	5	181.083	85.1720	18
7	1	5	140.436	83.4603	18
7	1	5	182.327	96.7710	29
7	-1	5	137.740	109.707	29

7	1	5	151.530	172.270	25
7	-1	5	146.734	166.826	25
7	1	5	55.7357	62.3779	6
7	-1	5	121.761	71.3698	7
7	-1	5	199.369	82.3119	6
7	-1	6	928.291	129.579	7
7	1	6	739.593	110.188	7
7	-1	6	736.236	125.035	6
7	1	6	709.623	113.092	6
7	1	6	916.104	171.970	29
7	-1	6	729.661	161.948	29
7	1	6	898.290	137.578	18
7	-1	6	715.786	136.189	18
-7	1	-6	569.467	234.671	23
-7	-1	-6	783.117	283.695	23
7	1	6	688.825	271.864	25
7	-1	6	810.780	282.601	25
7	-1	7	69.6626	61.5804	7
7	1	7	74.4411	76.7409	18
7	-1	7	38.6576	70.5725	18
7	1	7	76.4151	99.0249	29
7	-1	7	56.0898	87.1096	29
7	-1	8	38.5291	46.4365	7
7	1	8	52.2940	62.0529	18
7	1	8	84.3308	82.2556	29
7	-1	8	198.581	89.0374	29
7	1	9	5156.57	363.756	29
7	-1	9	5159.63	309.968	5
-7	1	-10	113.643	46.8714	20

-7	-1	-10	79.4479	46.2904	20
7	-1	10	72.8951	37.1641	5
7	1	10	82.0154	31.2463	5
7	-1	10	82.0377	50.8605	26
-7	1	-11	2309.32	150.315	20
-7	-1	-11	2765.73	162.093	20
-7	-2	14	173.020	61.6116	22
7	2	-14	234.819	54.7461	20
7	-2	-14	264.600	55.4787	20
-7	2	13	86.3814	58.6261	29
-7	-2	13	41.1561	47.4495	29
7	2	-13	86.2902	62.4850	20
7	-2	-13	115.118	60.9813	20
7	2	-12	225.057	66.8729	19
7	-2	-12	385.323	82.9041	11
7	2	-12	293.401	91.0274	20
7	-2	-12	267.905	83.3225	20
-7	-2	12	421.917	97.5889	29
-7	2	12	430.445	110.353	29
7	2	-11	-25.9061	74.5772	19
7	-2	-11	129.223	75.5506	11
7	2	-11	256.836	98.3910	20
7	-2	-11	106.475	86.1611	20
7	2	-10	952.838	152.481	19
7	-2	-10	1291.45	370.777	17
7	-2	-10	1302.89	173.296	11
7	2	-10	1139.20	169.246	20
7	-2	-10	1256.37	174.902	20
7	2	-9	4402.85	342.524	20

7	-2	-9	3568.04	321.390	20
7	2	-9	4165.48	337.020	19
-7	2	9	3029.77	499.336	30
-7	-2	9	4247.62	565.777	30
7	-2	-9	3326.38	593.036	17
7	2	-9	3573.82	586.193	17
7	-2	-9	4069.27	329.114	11
7	-2	-9	4385.95	580.537	1
7	2	-8	2191.12	231.662	20
7	-2	-8	2473.53	244.467	20
7	2	-8	2107.25	231.424	19
-7	-2	8	2833.28	449.427	30
-7	2	8	2192.90	406.651	30
7	2	-8	2148.68	429.162	17
7	-2	-8	2771.52	516.570	17
7	-2	-8	2311.01	238.748	11
7	-2	-8	2982.14	496.255	1
7	2	-7	18172.4	1063.51	20
7	-2	-7	19457.6	1074.56	20
7	2	-7	18958.4	1078.03	19
-7	-2	7	20656.1	1442.54	30
-7	2	7	22392.9	1471.61	30
7	2	-7	19477.9	1474.81	17
7	-2	-7	22184.2	1599.89	17
7	-2	-7	19332.0	1075.49	11
7	-2	-7	22308.2	1567.86	1
7	2	-6	7540.48	507.874	19
7	2	-6	7679.65	501.209	20
7	-2	-6	7737.77	500.639	20

7	2	-6	8334.03	827.262	17
7	-2	-6	8145.40	847.956	17
7	-2	-6	7831.43	507.317	11
7	-2	-6	8878.57	869.915	1
7	-2	-5	3806.83	530.559	17
7	2	-5	3287.10	486.555	17
7	2	-5	3239.54	269.724	20
7	-2	-5	3204.73	266.578	20
7	2	-5	3203.34	273.040	19
7	-2	-5	3397.18	275.194	11
7	2	-4	588.546	97.9963	26
7	2	-4	681.310	209.207	25
7	-2	-4	373.981	182.006	17
7	2	-4	662.411	233.075	17
7	-2	-4	505.840	222.784	1
7	2	-3	10.5815	50.4407	26
7	-2	-3	41.1747	46.7863	26
7	2	-3	42.8875	97.4505	25
7	-2	-3	41.6966	94.7446	25
7	2	-3	-49.5615	112.614	17
7	-2	-3	-50.5958	102.828	17
7	-2	-3	52.0646	149.639	1
7	2	-2	834.810	230.873	25
7	-2	-2	913.245	120.196	26
7	2	-2	824.995	119.383	26
7	-2	-2	554.469	187.847	25
7	-2	-2	629.820	206.584	1
7	-2	-2	1135.72	290.788	17
7	2	-1	4471.26	319.928	26

7	-2	-1	4341.57	312.948	26
7	2	-1	4040.70	513.177	25
7	-2	-1	5288.54	557.338	25
7	-2	-1	4134.04	474.730	2
7	-2	-1	4753.82	543.956	1
7	-2	0	475.451	92.0748	26
7	-2	0	708.275	226.294	25
7	2	0	443.401	188.811	25
7	2	0	556.100	106.194	26
7	2	0	456.365	161.283	1
7	-2	0	575.474	212.312	1
7	-2	0	435.972	152.546	2
7	-2	1	7544.99	726.015	2
7	-2	1	9410.88	757.625	1
7	2	1	7486.16	507.313	18
7	2	1	7961.00	505.989	19
7	2	1	8426.56	810.250	25
7	-2	1	8743.50	800.187	25
7	2	1	8351.73	519.365	26
7	-2	1	8887.65	521.630	26
7	-2	1	7888.81	498.216	14
7	2	2	68.8594	56.5450	13
7	-2	2	-69.3233	56.3049	14
7	2	2	57.9997	102.079	25
7	-2	2	-55.5958	112.985	25
7	-2	2	-13.5612	60.0660	26
-7	-2	-2	-60.0436	149.449	23
-7	2	-2	-60.2927	136.993	23
-7	-2	-2	46.0603	51.0033	22

7	2	2	11.9827	48.7045	19
7	-2	2	-47.3047	96.1358	2
7	-2	2	76.4228	95.1085	1
7	-2	2	47.0606	63.2601	18
7	2	3	10437.8	999.365	25
7	-2	3	9726.70	949.959	25
-7	-2	-3	10481.9	615.616	22
-7	-2	-3	11493.9	1003.07	23
-7	2	-3	11543.9	992.823	23
7	-2	3	9859.71	907.468	2
7	-2	3	10922.1	627.972	18
7	2	3	10911.5	636.681	18
7	2	3	10293.1	616.328	19
7	-2	3	10557.4	615.579	6
7	2	3	11616.6	677.944	29
7	2	3	9107.44	593.902	13
7	-2	3	9935.16	610.331	14
7	2	4	113.113	71.9758	18
7	-2	4	164.515	77.2845	6
7	-2	4	89.1506	69.8345	7
7	2	4	84.2460	56.7993	6
7	2	4	69.3735	157.690	25
7	-2	4	197.427	176.980	25
-7	2	-4	116.112	177.034	23
-7	-2	-4	163.130	69.9828	22
7	-2	4	40.3086	64.1945	18
7	2	4	56.0147	87.0593	29
7	-2	4	106.714	86.7886	29
7	-2	4	62.6021	68.6585	14

7	-2	4	58.1575	118.276	2
7	2	5	-73.4516	149.802	25
7	-2	5	278.612	187.679	25
7	-2	5	439.641	202.205	2
7	2	5	378.711	102.559	18
7	-2	5	340.472	92.3677	7
7	2	5	267.404	71.9883	6
7	-2	5	337.001	94.8955	6
7	2	5	275.486	113.807	29
7	-2	5	243.169	109.910	29
-7	2	-5	180.896	150.610	23
-7	-2	-5	446.009	205.122	23
-7	2	-6	120.594	150.080	23
-7	-2	-6	-192.608	145.879	23
7	2	6	98.1163	101.676	29
7	-2	6	-18.6942	87.0520	29
7	-2	6	-13.7182	68.2925	18
7	-2	6	-25.3497	76.1979	6
7	2	6	124.707	57.6717	6
7	-2	6	71.1699	62.6328	7
7	2	6	-74.1619	168.507	25
7	-2	6	-69.6715	122.623	25
7	2	6	39.9467	68.9949	18
7	-2	7	6480.04	388.204	7
7	2	7	6159.99	397.071	18
7	2	7	5552.78	441.059	29
7	-2	8	9580.97	497.215	7
7	-2	8	10582.0	534.217	5
7	2	8	10993.9	524.986	18

7	-2	9	1063.55	117.493	5
7	-2	9	1042.94	97.1435	7
7	2	10	1035.24	114.708	26
7	-2	10	987.361	92.7988	5
-7	2	-10	1123.96	99.5984	20
-7	-2	-11	44.0326	24.2174	20
-7	-3	14	25.4696	36.0439	22
7	3	-13	380.563	77.5048	20
7	-3	-13	456.216	78.3741	20
-7	3	13	331.179	72.2711	29
7	-3	-13	407.086	70.9822	11
7	3	-12	815.784	121.209	20
7	-3	-12	846.166	121.379	20
7	3	-12	959.746	125.196	19
-7	-3	12	576.530	100.006	29
7	-3	-12	789.610	115.937	11
7	3	-11	10776.0	640.840	19
7	-3	-11	11243.7	643.690	11
7	3	-11	10870.0	639.538	20
7	-3	-11	11096.4	642.006	20
-7	3	11	14226.4	691.138	29
-7	-3	11	7657.61	596.543	29
7	3	-10	1062.47	165.432	19
7	-3	-10	995.500	157.777	11
7	3	-10	837.701	139.671	20
7	-3	-10	903.786	151.767	20
7	3	-9	8119.40	525.812	20
7	-3	-9	7768.40	519.793	20
7	3	-9	8655.01	551.244	19

-7	-3	9	8686.12	836.829	30
-7	3	9	8296.79	829.624	30
7	-3	-9	7098.87	888.460	17
7	-3	-9	8319.37	540.721	11
7	-3	-9	7029.49	766.099	1
7	3	-8	211.326	82.9873	20
7	-3	-8	239.994	87.9229	20
7	3	-8	184.712	89.0232	19
-7	-3	8	157.928	151.510	30
-7	3	8	55.5492	149.555	30
7	3	-8	186.729	179.069	17
7	-3	-8	73.8999	150.398	17
7	-3	-8	233.295	89.9579	11
7	-3	-8	59.8749	121.945	1
7	3	-7	128.938	75.8785	20
7	-3	-7	184.198	82.9233	20
7	3	-7	112.323	88.0076	19
-7	-3	7	100.567	135.315	30
-7	3	7	105.359	151.521	30
7	3	-7	118.409	134.653	17
7	-3	-7	206.517	185.165	17
7	-3	-7	95.0190	72.2125	11
7	-3	-7	122.461	139.253	1
7	3	-6	23735.0	1293.08	20
7	-3	-6	26282.1	1302.96	20
7	3	-6	24559.0	1321.56	19
-7	-3	6	18515.5	1586.41	30
7	3	-6	21587.1	1628.77	17
7	-3	-6	29183.5	1832.70	17

7	-3	-6	24693.9	1315.10	11
7	-3	-6	26205.8	1757.61	1
-7	-3	5	4563.27	366.782	22
7	3	-5	5036.57	362.789	19
7	-3	-5	5698.44	654.592	17
7	3	-5	3329.85	508.461	17
-7	3	5	3136.77	506.306	30
7	-3	-5	4957.69	356.637	11
7	-3	-5	6063.41	672.530	1
7	-3	-4	22500.5	1500.02	17
-7	-3	4	21452.6	1124.54	22
7	-3	-4	21543.8	1110.16	11
7	3	-3	741.962	122.587	19
7	-3	-3	650.954	216.397	1
7	3	-3	704.375	111.517	26
7	-3	-3	807.106	112.729	26
7	3	-3	763.798	222.688	25
7	-3	-3	739.039	211.581	25
7	-3	-3	925.987	263.752	17
7	3	-2	1222.28	152.126	19
7	-3	-2	1118.71	278.219	2
7	-3	-2	1239.84	297.313	1
7	3	-2	1102.52	139.212	26
7	3	-2	1094.59	257.512	25
7	-3	-2	1591.46	290.636	25
7	-3	-2	1193.40	136.387	26
7	-3	-2	749.523	254.649	17
7	3	-1	15615.0	835.501	26
7	-3	-1	14783.3	823.398	26

7	3	-1	14409.7	1105.63	25
7	-3	-1	15701.8	1123.82	25
7	3	-1	14593.1	841.863	19
7	-3	-1	15413.1	1128.71	2
7	-3	-1	17305.0	1214.47	1
7	-3	0	3381.60	455.338	1
7	-3	0	2074.33	374.375	2
7	3	0	2947.43	253.861	18
7	3	0	2838.24	243.246	19
7	-3	0	2755.96	230.891	26
7	3	0	2592.28	410.229	25
7	-3	0	2855.07	416.016	25
7	3	0	2949.30	242.405	26
7	-3	0	2358.61	223.999	12
7	-3	0	3050.84	243.873	11
7	-3	0	3031.47	240.214	13
-7	-3	0	2978.56	249.818	22
-7	-3	-1	288.024	86.7452	22
7	-3	1	197.543	68.2094	18
7	3	1	196.742	74.4346	19
7	-3	1	67.3124	56.5268	6
7	-3	1	264.849	75.4306	13
7	-3	1	145.650	65.8445	26
7	3	1	285.288	84.8244	26
7	3	1	264.701	178.628	25
7	-3	1	148.834	123.792	25
7	-3	1	103.219	117.589	2
7	-3	1	182.101	122.703	1
7	3	2	6596.22	727.129	25

7	-3	2	6075.12	684.914	25
-7	-3	-2	5947.48	416.198	22
-7	-3	-2	6991.15	756.876	23
-7	3	-2	7144.51	745.630	23
7	-3	2	5501.23	614.588	1
7	-3	2	6495.86	718.873	2
7	-3	2	6297.66	413.440	18
7	3	2	6219.75	432.015	18
7	3	2	6149.00	414.849	19
7	3	2	6528.38	435.253	26
7	-3	2	6154.64	422.456	26
7	-3	2	5965.49	409.918	6
7	-3	2	6609.12	424.929	14
7	-3	2	6585.21	427.413	13
7	3	3	741.081	131.138	18
7	-3	3	614.175	114.043	6
7	-3	3	759.250	123.299	7
7	3	3	436.216	202.327	25
7	-3	3	407.113	189.115	25
7	-3	3	649.679	118.411	26
-7	-3	-3	693.050	243.697	23
-7	3	-3	869.806	266.501	23
-7	-3	-3	657.918	113.215	22
7	3	3	586.628	108.579	19
7	-3	3	678.864	119.933	18
7	-3	3	480.720	104.496	14
7	-3	3	664.344	247.181	2
7	3	3	509.605	123.048	29
7	3	4	22358.3	1624.28	25

7	-3	4	20598.7	1561.59	25
7	-3	4	19441.4	1559.18	2
7	3	4	21602.5	1155.62	18
7	-3	4	20750.7	1142.39	18
-7	-3	-4	22789.4	1586.43	23
-7	3	-4	20945.1	1506.96	23
-7	-3	-4	21813.4	1146.21	22
7	-3	4	22072.4	1145.60	7
7	3	4	20641.7	1102.02	6
7	-3	4	22297.6	1148.57	14
7	3	4	19383.7	1172.28	29
-7	3	-5	10614.7	1050.61	23
-7	-3	-5	14824.4	1209.39	23
-7	-3	-5	15245.1	813.173	22
7	-3	5	12339.5	1179.80	2
7	3	5	14115.4	802.020	18
7	3	5	11330.4	811.621	29
7	3	5	13065.7	1211.12	25
7	-3	5	13566.0	1187.48	25
7	-3	5	15292.3	807.693	6
7	-3	5	15089.9	804.358	7
7	3	6	1176.45	147.986	18
7	-3	6	1203.05	147.695	7
-7	-3	-6	372.738	193.655	23
7	3	6	531.872	139.112	29
7	-3	7	2835.84	234.458	5
7	-3	7	2673.36	213.051	7
7	3	7	2516.75	215.089	18
7	-3	8	2051.75	177.341	5

7	-3	8	1793.71	152.325	7
7	-3	8	1852.83	176.844	26
7	3	8	1574.58	146.158	18
7	-3	9	-5.59863	35.9840	7
7	-3	9	7.74350	45.2037	5
-7	3	-9	50.4200	42.9638	20
7	-3	10	194.693	41.3733	5
-7	3	-10	148.989	32.7223	20
-7	-3	-10	261.982	53.4069	20
7	-4	-13	357.339	64.3246	11
-7	4	12	105.160	63.0022	29
7	-4	-12	20.2019	57.1987	11
7	4	-12	40.8475	51.9400	19
7	4	-11	229.428	95.5912	19
7	-4	-11	133.024	80.6576	11
7	4	-11	267.360	79.9341	20
7	-4	-11	230.800	76.4716	20
-7	4	11	268.082	92.3262	29
7	4	-10	14816.3	678.076	19
7	-4	-10	13997.3	665.659	11
7	4	-10	13851.1	627.308	20
7	-4	-10	13636.9	621.890	20
7	4	-9	7267.28	501.955	19
7	-4	-9	6180.89	801.933	17
7	-4	-9	7002.12	498.024	11
7	4	-9	6831.92	465.653	20
7	-4	-9	7303.00	469.894	20
7	4	-8	7664.92	507.644	20
7	-4	-8	8362.81	520.038	20

7	4	-8	7633.24	540.113	19
7	-4	-8	8896.82	935.235	17
-7	-4	8	8160.75	779.525	30
-7	4	8	8009.99	796.971	30
7	-4	-8	7613.83	538.751	11
7	-4	-8	8037.81	815.665	1
7	4	-7	18308.7	967.624	20
7	-4	-7	18690.2	965.182	20
-7	-4	7	15039.3	1010.40	22
7	-4	-7	18085.0	1460.28	17
-7	4	7	18666.8	1346.31	30
7	-4	-7	17822.9	998.221	11
7	-4	-7	18017.1	1363.40	1
-7	-4	6	3217.24	284.955	22
-7	-4	6	948.112	286.509	30
-7	4	6	2476.98	430.583	30
7	4	-6	1526.31	339.766	17
7	-4	-6	3048.93	494.305	17
7	-4	-6	3046.80	263.821	11
7	-4	-6	2739.39	472.149	1
7	4	-5	4970.39	324.828	19
-7	-4	5	403.960	340.130	30
-7	4	5	997.328	351.494	30
7	-4	-5	3818.53	558.623	17
7	4	-5	1577.22	372.228	17
-7	-4	5	4605.03	327.045	22
7	-4	-5	6157.65	668.771	1
7	-4	-4	522.497	117.949	11
-7	-4	4	342.990	99.2934	22

7	4	-4	380.840	96.9806	19
7	-4	-4	346.152	97.3302	12
7	-4	-4	324.835	191.413	17
7	-4	-4	487.097	103.545	3
-7	4	4	51.1630	173.358	30
7	-4	-3	1493.27	176.115	14
7	-4	-3	1753.77	182.058	12
-7	-4	3	1542.12	182.345	22
7	-4	-3	1517.87	174.341	11
7	4	-3	1505.49	177.900	19
7	4	-3	1649.10	310.049	25
7	4	-3	1548.40	161.181	26
7	-4	-3	1632.10	357.289	1
7	-4	-3	568.445	239.293	17
7	-4	-3	1416.31	167.787	3
-7	-4	2	52830.8	2799.49	22
7	-4	-2	55580.0	2790.91	11
7	4	-2	55025.5	2799.57	19
7	4	-2	50205.6	3059.46	25
7	-4	-2	55697.8	3071.85	25
7	4	-2	59345.1	2773.06	26
7	-4	-2	58068.6	2762.21	26
7	-4	-2	56689.4	2794.06	14
7	-4	-2	56202.1	2774.85	13
7	-4	-2	56169.2	2781.11	12
7	-4	-2	57423.0	3202.72	2
7	-4	-2	63392.7	3296.95	1
-7	-4	1	1438.64	176.223	22
7	-4	-1	1397.29	165.927	11

7	4	-1	1520.22	179.320	19
7	4	-1	1622.89	187.317	18
7	-4	-1	1473.39	171.771	14
7	-4	-1	1796.89	173.847	13
7	-4	-1	1317.43	163.758	12
7	-4	-1	1406.84	324.084	1
7	-4	-1	1143.69	286.067	2
7	4	-1	1838.00	323.839	25
7	-4	-1	1282.32	281.910	25
7	4	-1	1792.24	172.226	26
7	-4	-1	1541.33	159.178	26
7	-4	-1	1581.07	159.794	7
7	-4	-1	1693.89	163.533	6
7	-4	0	13753.1	760.154	7
7	-4	0	14049.4	755.340	6
7	-4	0	13247.6	1111.76	2
7	-4	0	14310.5	1106.71	1
7	4	0	13387.0	769.532	19
-7	-4	0	12218.7	770.410	22
-7	-4	0	11534.9	1175.28	23
7	-4	0	13798.4	752.012	26
7	4	0	14296.9	767.084	26
7	4	0	13897.3	1074.12	25
7	-4	0	12846.7	1017.54	25
7	-4	0	13427.3	765.024	11
7	-4	0	13541.3	757.777	13
7	-4	0	13476.4	769.474	14
7	-4	1	12092.6	720.283	26
7	-4	1	13019.2	1019.82	25

7	4	1	13292.7	1059.08	25
-7	-4	-1	16732.1	1206.67	23
-7	-4	-1	12700.4	751.188	22
7	-4	1	14096.9	738.636	6
7	-4	1	13718.8	746.302	7
7	-4	1	12794.7	1107.96	2
7	-4	1	13954.6	1088.02	1
7	4	1	13233.2	763.624	18
7	4	1	12301.0	734.761	19
7	4	1	12893.2	739.492	26
7	-4	1	13287.2	743.860	13
7	-4	1	13598.7	749.689	14
7	-4	2	244.641	83.5878	14
7	-4	2	242.631	75.9099	26
7	4	2	111.195	149.936	25
7	-4	2	199.114	134.347	25
7	4	2	216.981	81.2160	26
-7	-4	-2	325.711	191.448	23
-7	4	-2	99.8716	124.851	23
-7	-4	-2	194.671	82.0234	22
7	4	2	330.578	89.6305	19
7	4	2	337.754	97.4244	18
7	-4	2	267.659	76.1221	6
7	-4	2	331.180	94.9662	7
7	-4	2	350.355	90.2058	4
7	-4	2	411.477	188.812	1
7	-4	2	63.5982	112.555	2
7	4	3	8413.69	850.335	25
7	-4	3	8573.22	832.268	25

-7	-4	-3	8540.44	541.995	22
-7	-4	-3	8054.36	825.218	23
-7	4	-3	8650.20	807.789	23
7	-4	3	8434.04	900.149	2
7	4	3	8143.82	541.074	18
7	4	3	8216.15	514.523	19
7	-4	3	7681.52	530.485	4
7	-4	3	8941.26	533.087	6
7	4	3	8696.51	544.395	26
7	-4	3	8790.64	531.147	26
7	4	4	189.770	143.956	25
7	-4	4	333.164	178.610	25
7	-4	4	120.402	72.3878	26
-7	-4	-4	234.792	158.014	23
-7	-4	-4	242.965	89.8903	22
7	4	4	99.5978	83.4491	18
7	-4	4	172.186	81.1592	6
7	-4	4	186.001	81.4061	7
7	-4	4	141.976	161.498	2
7	-4	4	194.074	92.7240	5
7	-4	5	1801.83	400.335	2
7	4	5	2132.13	202.774	18
7	4	5	1733.93	375.488	25
7	-4	5	1986.36	195.948	7
-7	-4	-5	1304.66	314.861	23
-7	-4	-5	2006.55	203.786	22
7	-4	5	1810.22	187.545	26
7	-4	6	2964.73	230.462	26
7	-4	6	3087.39	228.965	7

7	-4	6	3022.89	237.189	5
7	4	6	2681.91	215.642	18
7	-4	7	4288.62	305.564	26
7	-4	7	3768.74	293.699	7
7	-4	7	4323.30	316.639	5
7	4	7	4568.20	300.371	18
7	-4	8	82.2125	53.6742	7
7	-4	8	137.046	66.1291	5
7	-4	8	46.1960	58.8438	4
7	4	8	117.872	53.3767	18
-7	-4	-9	740.362	87.3165	20
7	-4	9	657.247	96.3496	4
7	4	9	710.607	92.3477	26
7	-4	9	692.792	78.8518	7
7	-4	9	791.084	96.2546	5
7	-4	10	142.712	34.6650	5
-7	-5	13	649.601	86.1361	22
7	-5	-13	801.123	83.6633	11
7	5	-13	638.307	76.3170	19
-7	-5	12	1222.04	139.185	22
7	5	-12	1111.92	124.286	19
7	-5	-12	1275.75	129.923	11
7	5	-11	724.116	123.141	19
-7	5	11	710.051	101.105	29
7	-5	-11	577.313	114.683	11
-7	5	10	49.3984	66.4028	29
7	-5	-10	14.9651	71.3254	11
7	5	-10	-45.4044	79.9118	19
7	-5	-9	16.6969	75.8792	11

7	-5	-8	4909.39	596.401	1
7	-5	-8	5965.69	420.826	11
7	-5	-7	4112.48	548.748	1
-7	-5	7	3510.43	280.619	27
7	-5	-7	2593.97	473.695	17
7	5	-7	3749.08	323.456	19
7	5	-6	615.145	131.070	19
-7	-5	6	818.512	127.224	27
7	-5	-6	1096.23	298.795	1
-7	-5	6	779.436	151.358	22
7	-5	-6	528.994	226.134	17
7	-5	-5	4237.98	338.895	11
-7	-5	5	3878.82	340.664	22
7	5	-5	4094.29	334.557	19
7	-5	-5	3668.85	312.626	12
7	-5	-5	4443.53	329.574	14
7	-5	-5	4031.55	571.274	1
7	-5	-5	2823.39	504.720	17
7	-5	-5	4576.83	348.426	3
-7	-5	4	9274.95	591.949	22
7	-5	-4	9540.05	580.308	11
7	5	-4	8988.51	577.393	19
7	-5	-4	9411.06	556.132	12
7	-5	-4	9134.95	559.895	14
7	-5	-4	9358.84	913.175	1
7	-5	-4	9415.58	580.622	3
-7	-5	3	21768.8	1214.73	22
7	-5	-3	22237.8	1196.22	11
7	5	-3	22001.2	1205.15	19

-7	-5	3	21229.8	1169.34	28
7	-5	-3	20900.1	1192.55	3
7	-5	-3	22612.2	1180.44	12
7	-5	-3	23001.1	1194.53	14
7	-5	-3	23733.7	1167.35	13
-7	-5	2	1502.51	185.328	22
7	-5	-2	1332.82	167.455	11
7	5	-2	1444.53	176.067	19
-7	-5	2	1624.72	172.349	28
7	-5	-2	1381.14	341.775	1
7	-5	-2	1473.70	354.682	2
7	-5	-2	1497.17	148.954	7
7	-5	-2	1431.42	163.449	12
7	-5	-2	1517.16	158.809	13
7	-5	-2	1979.48	190.483	14
7	-5	-1	11439.5	872.824	25
7	5	-1	12798.1	656.766	26
7	-5	-1	12235.8	639.075	26
-7	-5	1	11312.5	694.698	22
7	-5	-1	11449.0	672.313	11
7	5	-1	11408.9	678.046	19
7	-5	-1	13065.9	689.405	14
7	-5	-1	12474.8	664.583	13
7	-5	-1	11915.5	1070.09	2
7	-5	-1	14182.1	1113.26	1
7	-5	-1	12375.8	648.422	6
7	-5	-1	11756.6	652.079	7
-7	-5	1	11306.4	669.468	28
-7	-5	0	2335.68	232.642	22

-7	-5	0	2214.87	426.772	23
7	-5	0	2301.94	200.434	5
7	5	0	2424.41	239.678	18
7	5	0	2133.26	214.134	19
7	5	0	2431.71	208.579	26
7	-5	0	2447.54	197.525	26
7	-5	0	2519.80	449.137	2
7	-5	0	2562.76	444.658	1
7	-5	0	2527.92	225.850	14
7	-5	0	1967.44	206.245	11
7	-5	0	2408.18	220.170	13
7	-5	0	2240.78	202.838	6
7	-5	0	2512.91	215.367	7
-7	-5	-1	8635.55	904.675	23
-7	-5	-1	10321.1	628.577	22
7	-5	1	9314.53	608.486	4
7	-5	1	9644.10	593.634	5
7	5	1	10040.6	634.293	18
7	5	1	9546.33	599.456	19
7	5	1	10861.9	606.843	26
7	-5	1	10082.2	579.614	26
7	-5	1	10166.3	953.218	1
7	-5	1	10552.0	1025.02	2
7	-5	1	10392.0	616.144	14
7	-5	1	10840.3	603.623	6
7	-5	1	10837.7	614.247	7
7	5	2	4470.63	323.263	26
7	-5	2	4211.66	299.228	26
-7	-5	-2	4447.54	339.601	22

-7	-5	-2	4026.53	530.937	23
7	-5	2	3863.43	325.708	4
7	-5	2	4127.81	318.493	5
7	5	2	4351.97	316.358	19
7	5	2	3966.76	332.391	18
7	-5	2	4619.82	653.037	2
7	-5	2	4018.84	315.916	6
7	-5	2	4575.56	328.880	7
7	-5	2	4889.65	338.143	14
7	5	3	1111.55	146.891	26
7	-5	3	1276.45	158.373	14
-7	-5	-3	691.332	233.912	23
-7	-5	-3	1019.19	153.477	22
7	5	3	999.310	129.102	19
7	-5	3	1028.35	302.140	2
7	-5	3	889.889	136.654	6
7	-5	3	1041.22	144.249	7
7	-5	3	761.577	128.575	5
7	-5	3	1158.94	159.512	4
7	5	3	892.039	143.881	18
7	5	4	7469.55	479.046	26
7	5	4	7444.71	480.936	18
7	-5	4	5758.30	794.709	2
7	-5	4	7343.86	497.953	4
7	-5	4	7275.30	480.166	5
-7	-5	-4	6291.85	724.355	23
-7	-5	-4	7591.74	492.223	22
7	5	5	10878.8	661.872	18
7	5	5	12100.6	680.954	26

7	-5	5	11694.5	672.776	7
7	-5	5	11347.7	697.711	4
-7	-5	-5	11210.1	684.112	22
7	5	6	1978.60	205.341	26
7	-5	6	2649.05	225.385	7
7	5	6	2511.49	211.307	18
7	-5	6	2573.34	244.953	4
7	-5	6	2462.25	225.959	5
7	-5	7	926.223	125.245	7
7	5	7	894.732	121.778	26
7	5	7	941.084	111.486	18
7	-5	7	909.748	130.573	5
7	-5	7	1276.20	157.166	4
7	-5	8	1490.27	138.050	7
7	-5	8	1628.84	151.565	5
7	5	8	1186.73	129.891	26
7	-5	8	1442.76	154.970	4
7	-5	9	-46.9038	40.8553	4
7	-5	9	54.7136	41.4424	5
7	6	-13	3.40325	21.0375	19
7	-6	-13	10.1511	20.9166	11
7	-6	-12	7.30882	43.3049	11
7	6	-12	-7.46032	44.2025	19
-7	-6	12	-7.53522	43.3133	22
7	-6	-11	63.0736	57.5607	11
-7	-6	11	22.1774	61.7498	22
7	-6	-10	1352.02	169.446	11
-7	-6	10	1230.40	167.092	22
7	6	-10	1193.51	164.553	19

-7	-6	9	-32.1678	80.0660	22
7	6	-9	45.9659	82.3843	19
7	-6	-8	33983.5	1581.93	14
-7	-6	8	31855.6	1587.32	27
-7	-6	8	35099.1	1664.46	22
7	6	-8	34485.7	1647.63	19
7	6	-7	50.1165	73.9983	19
7	-6	-7	-16.4506	76.6081	11
7	-6	-7	-37.8765	82.1521	12
7	-6	-7	23.9159	60.7616	14
-7	-6	7	67.2744	66.9852	27
-7	-6	7	53.9241	94.9106	22
7	-6	-6	4667.18	336.337	12
7	-6	-6	4329.47	331.915	14
7	6	-6	4536.98	364.600	19
7	-6	-6	4619.67	603.619	1
7	-6	-6	4798.87	371.548	11
7	-6	-6	4834.82	383.606	3
-7	-6	6	4492.26	346.468	27
7	6	-5	129.170	86.9131	19
7	-6	-5	-61.5029	165.397	1
7	-6	-5	156.678	84.3434	11
7	-6	-5	156.340	67.0585	12
7	-6	-5	89.3146	67.4934	14
7	-6	-5	151.403	95.2636	3
7	6	-4	1481.47	186.880	19
7	-6	-4	1148.25	177.561	3
7	-6	-4	1496.14	188.499	11
7	-6	-4	1480.99	170.376	14

7	-6	-4	1585.00	167.583	12
-7	-6	4	1467.41	174.715	28
7	-6	-3	14180.8	774.907	12
7	-6	-3	14932.3	798.314	14
7	6	-3	13653.1	809.981	19
7	-6	-3	15097.8	1230.21	1
-7	-6	3	13144.0	789.552	28
7	-6	-3	13556.1	812.611	3
7	-6	-3	13680.5	802.082	11
7	-6	-2	17088.2	883.831	11
-7	-6	2	14609.7	871.081	28
7	6	-2	15058.7	879.010	19
7	-6	-2	14607.3	866.908	4
7	-6	-2	14884.2	876.271	3
7	-6	-2	17337.3	879.459	14
-7	-6	1	28.7462	58.4825	28
7	-6	-1	151.279	69.0367	14
7	-6	-1	99.2010	66.7287	11
-7	-6	1	111.258	88.5006	22
7	6	-1	81.4284	61.7316	19
7	6	-1	-48.0878	72.8917	18
7	-6	-1	99.1400	53.3749	7
7	-6	-1	69.3921	141.049	2
7	-6	-1	64.1566	172.502	1
7	-6	-1	13.6751	53.8861	4
7	-6	0	32.1351	47.4454	5
7	-6	0	-14.1785	57.6299	4
-7	-6	0	-15.5678	63.2765	22
7	6	0	12.7873	60.9461	19

7	6	0	-15.8581	82.1659	18
7	-6	0	-9.81895	49.8877	6
7	-6	0	22.1677	54.0147	7
7	-6	0	63.3380	64.3604	14
7	-6	0	-64.8449	131.782	1
7	-6	0	71.4716	205.413	2
7	-6	1	82.6777	66.9101	5
7	-6	1	14.8304	64.0269	4
-7	-6	-1	76.7595	82.6161	22
7	6	1	71.5935	52.9616	19
7	6	1	62.2718	80.7345	18
7	-6	1	119.631	74.0216	7
7	-6	1	102.370	75.8957	14
7	-6	1	73.5606	105.764	2
7	-6	2	216.907	82.7688	5
7	-6	2	370.254	103.411	4
-7	-6	-2	273.466	100.680	22
7	6	2	219.978	73.3023	19
7	6	2	240.626	97.3049	18
7	-6	2	151.001	80.6421	7
7	-6	2	456.403	232.156	2
7	-6	2	246.657	88.2242	14
-7	-6	-3	14416.3	822.840	22
7	6	3	13830.6	810.722	18
7	-6	3	13506.7	822.428	4
7	-6	3	13828.4	798.596	7
7	-6	3	13250.3	798.008	5
7	-6	3	13681.3	1278.93	2
7	-6	3	16061.1	818.170	14

7	6	4	10777.6	667.035	18
-7	-6	-4	11527.2	691.639	22
7	-6	4	11755.6	683.909	5
7	-6	4	12301.6	712.313	4
-7	-6	-5	162.003	83.8812	22
7	6	5	285.819	82.5301	18
7	-6	5	119.690	80.2100	7
7	-6	5	135.318	89.4949	4
7	-6	5	194.986	67.4966	10
7	-6	6	13930.4	796.619	4
7	-6	6	14168.7	772.957	7
7	6	6	13702.4	746.647	18
7	-6	7	33.5847	56.8867	4
7	-6	7	-29.8527	57.2081	5
7	-6	7	44.5247	56.5177	7
7	6	7	33.5025	36.7851	18
7	-6	8	425.870	78.9835	4
7	-6	8	546.685	84.9514	5
7	-6	8	453.335	74.6406	7
7	-6	9	63.3826	30.6606	5
7	7	-12	54.4379	34.4910	19
7	-7	-12	4.80228	31.2630	11
-7	-7	11	7.51091	41.1060	22
7	7	-11	-32.9863	50.9760	19
-7	-7	10	32.0425	58.5223	22
7	7	-10	109.424	70.3856	19
7	7	-9	300.139	95.3059	19
7	-7	-9	578.666	112.205	11
-7	-7	9	567.087	113.396	22

-7	-7	8	11.8812	53.9940	27
7	7	-8	58.0427	67.5710	19
7	-7	-8	14.2417	67.8789	11
-7	-7	8	-45.1090	74.8527	22
7	7	-7	11554.3	695.695	19
-7	-7	7	10758.7	668.224	27
7	-7	-7	11611.9	690.533	11
-7	-7	7	11599.3	703.056	22
7	7	-6	9230.94	594.675	19
7	-7	-6	8941.88	602.736	3
-7	-7	6	9604.39	589.064	27
7	-7	-6	9724.35	595.795	11
-7	-7	6	9091.83	605.406	22
-7	-7	5	1877.08	217.868	22
7	-7	-5	2186.88	217.153	11
7	7	-5	1722.37	203.519	19
7	-7	-5	1766.85	172.730	14
-7	-7	5	1673.25	201.418	27
7	-7	-5	1977.85	227.730	3
7	-7	-5	1684.76	371.906	1
-7	-7	4	11308.0	688.258	22
7	-7	-4	11391.5	1036.83	1
7	7	-4	10835.0	666.974	19
7	-7	-4	11362.3	663.070	11
7	-7	-4	11375.4	630.158	14
7	-7	-4	9637.21	640.891	4
7	-7	-4	10703.7	675.863	3
-7	-7	4	11064.6	665.659	28
-7	-7	3	399.043	105.620	28

-7	-7	3	449.952	118.718	22
7	7	-3	386.802	101.438	19
7	-7	-3	400.676	98.6063	11
7	-7	-3	440.331	94.0126	14
7	-7	-3	338.517	97.8458	4
7	-7	-3	398.534	224.587	1
7	-7	-3	302.817	98.8523	3
-7	-7	2	406.699	103.319	28
7	-7	-2	382.211	92.3514	11
-7	-7	2	441.458	122.460	22
7	7	-2	475.051	104.061	19
7	-7	-2	240.812	94.9790	3
7	-7	-2	416.406	93.4701	14
7	-7	-2	564.370	113.612	4
-7	-7	1	2112.84	230.068	28
7	-7	-1	1955.52	441.613	1
7	-7	-1	2288.70	457.054	2
7	7	-1	2316.94	247.490	18
7	7	-1	2183.03	213.212	19
7	-7	-1	2359.47	195.077	7
7	-7	-1	2548.50	218.728	14
7	-7	-1	2524.66	218.943	11
7	-7	-1	2538.04	240.081	4
7	-7	-1	2079.60	216.292	3
-7	-7	0	174.025	85.2331	28
7	-7	0	74.9332	170.343	2
7	7	0	69.5184	59.1104	19
7	7	0	126.349	85.0895	18
7	-7	0	114.204	60.8402	7

7	-7	0	76.1219	64.0095	4
7	-7	0	152.554	69.3553	5
7	-7	0	139.758	68.1943	14
7	-7	1	11483.2	1123.06	2
7	7	1	11542.8	688.901	18
7	7	1	11265.9	640.733	19
-7	-7	-1	10986.3	691.056	28
7	-7	1	10598.9	650.145	5
7	-7	1	11214.8	650.210	7
7	-7	1	11065.2	687.923	4
7	-7	1	12379.0	665.725	14
7	-7	2	16837.8	973.313	4
7	7	2	17415.5	965.074	18
7	-7	2	17432.0	941.784	7
7	-7	2	17245.5	946.108	5
7	7	3	5416.77	382.931	18
7	-7	3	5681.46	381.122	7
7	-7	3	5657.97	386.015	5
7	-7	3	5618.81	407.714	4
-7	-7	-3	5173.60	392.797	22
7	7	4	616.465	108.625	18
7	-7	4	718.951	121.645	7
-7	-7	-4	717.408	126.647	22
7	-7	4	533.984	114.807	4
7	-7	4	654.897	121.795	5
7	-7	5	473.886	108.453	4
7	-7	5	459.175	104.180	5
7	7	5	384.882	81.0395	18
7	-7	6	167.677	70.4019	7

7	-7	6	184.431	74.2709	4
7	7	6	76.7343	40.1423	18
7	-7	7	567.710	92.7998	4
7	-7	7	843.987	106.232	5
7	-7	7	555.002	89.8574	7
7	-7	8	649.309	76.6664	4
7	-7	8	798.252	85.8446	5
7	8	-11	739.698	78.3244	19
7	-8	-11	756.417	78.1079	11
7	8	-10	835.753	103.723	19
7	-8	-10	771.330	98.5604	11
7	8	-9	466.277	90.8524	19
7	-8	-9	449.935	87.8367	11
-7	-8	9	409.266	84.9612	22
-7	-8	8	513.955	94.5126	27
7	8	-8	763.075	116.685	19
7	-8	-8	402.272	93.6140	11
-7	-8	8	565.023	106.536	22
-7	-8	7	-24.7112	58.9299	27
7	8	-7	63.3386	73.9780	19
7	-8	-7	-24.2857	69.8339	11
-7	-8	7	-118.862	79.4247	22
7	8	-6	1914.96	194.207	19
-7	-8	6	1615.75	187.956	27
-7	-8	6	1833.40	200.156	22
7	-8	-6	1507.06	173.660	11
7	8	-5	677.746	122.822	19
7	-8	-5	450.143	119.288	3
-7	-8	5	745.741	131.728	27

-7	-8	5	735.107	140.953	22
7	-8	-5	885.684	130.812	11
7	8	-4	52.0063	67.4366	19
7	-8	-4	93.4599	85.2927	3
7	-8	-4	47.8403	53.0651	11
-7	-8	4	107.260	82.4479	22
7	8	-3	9111.69	564.945	19
7	-8	-3	9121.93	580.921	4
7	-8	-3	9166.73	593.253	3
-7	-8	3	8786.18	583.578	28
7	-8	-3	10016.6	561.787	11
-7	-8	3	9925.97	599.912	22
-7	-8	2	847.860	143.557	22
7	8	-2	1048.49	131.671	19
7	-8	-2	864.295	142.447	3
-7	-8	2	774.783	133.715	28
7	-8	-2	671.933	127.271	4
7	-8	-2	704.741	110.543	11
-7	-8	1	11053.0	670.084	22
7	-8	-1	11533.3	617.379	14
7	8	-1	10804.5	677.861	18
7	8	-1	10610.4	624.069	19
7	-8	-1	11337.9	675.217	4
7	-8	-1	9945.03	639.599	3
-7	-8	1	11949.7	685.725	28
-7	-8	0	418.888	108.850	28
7	8	0	424.798	112.256	18
7	-8	0	450.253	86.7938	7
-7	-8	0	376.030	107.920	22

7	-8	0	442.021	84.7013	14
7	-8	0	448.288	91.9491	5
7	-8	0	453.467	112.622	4
-7	-8	-1	27.9549	69.5797	22
7	-8	1	9.95794	55.4218	7
7	8	1	-81.9740	83.2964	18
-7	-8	-1	77.2824	78.5290	28
7	-8	1	45.3606	65.1844	4
7	-8	1	-11.1453	58.8468	5
-7	-8	-2	172.880	86.8819	22
7	8	2	123.143	67.8415	18
7	-8	2	229.548	81.1267	7
-7	-8	-2	271.889	93.6925	28
7	-8	2	133.221	79.9776	4
7	-8	2	127.686	74.0785	5
7	-8	3	1296.01	172.205	4
7	8	3	1357.10	154.973	18
7	-8	3	1511.40	156.691	7
7	-8	3	1636.27	169.879	5
-7	-8	-3	1517.57	181.157	28
-7	-8	-3	1164.28	135.435	9
7	-8	4	98.9390	65.4776	7
7	-8	4	115.778	71.8910	4
7	-8	4	132.295	69.3617	5
7	8	4	192.131	60.9660	18
7	-8	5	814.141	111.632	7
7	-8	5	1077.57	134.704	4
7	-8	5	876.282	120.497	5
7	8	5	840.768	90.1026	18

7	-8	6	2346.62	192.395	4
7	-8	7	34.2072	39.7485	4
7	9	-10	341.888	42.8677	19
7	9	-9	87.1254	43.5642	19
7	9	-8	1007.44	111.782	19
-7	-9	7	50.8582	56.6303	27
-7	-9	7	35.8499	49.9032	22
7	9	-7	17.5864	50.5659	19
-7	-9	6	-23.6487	62.4328	27
-7	-9	6	10.5016	57.4657	22
7	9	-6	38.0123	54.6254	19
-7	-9	5	6952.07	455.254	27
7	9	-5	6328.47	416.493	19
7	-9	-5	7243.06	461.585	3
-7	-9	5	7132.93	442.780	22
-7	-9	4	4860.97	362.182	27
7	9	-4	4769.38	324.516	19
7	-9	-4	4713.74	354.973	3
-7	-9	4	5360.90	358.206	22
7	-9	-3	2189.72	219.814	4
7	-9	-3	2358.94	225.252	3
7	9	-3	2482.20	197.090	19
-7	-9	3	2034.30	203.579	16
-7	-9	3	2413.14	221.860	22
-7	-9	3	2756.30	239.654	28
-7	-9	2	313.974	95.7347	22
7	-9	-2	402.920	97.9662	3
7	9	-2	327.431	104.739	18
-7	-9	2	295.846	83.0791	16

7	-9	-2	317.956	95.3303	4
-7	-9	2	209.357	88.6070	28
7	-9	-1	433.114	105.001	4
-7	-9	1	489.863	108.441	28
7	9	-1	328.423	102.037	18
-7	-9	1	534.143	109.106	22
-7	-9	0	2230.30	226.498	28
7	-9	0	2451.56	234.006	4
7	9	0	2674.83	231.525	18
-7	-9	0	2590.35	226.768	22
-7	-9	-1	-26.8792	64.0600	28
-7	-9	-1	-23.0286	57.3246	22
7	-9	1	-26.4526	69.8398	4
7	9	1	11.0301	59.3136	18
-7	-9	-2	216.433	79.0603	28
-7	-9	-2	139.797	71.2256	22
7	-9	2	263.809	85.9696	4
7	-9	2	159.851	67.9915	5
7	9	2	103.071	58.4163	18
-7	-9	-3	-13.8610	52.2555	9
-7	-9	-3	46.2306	56.3492	28
-7	-9	-3	58.4424	55.1341	22
7	-9	3	34.3656	59.3777	4
7	-9	3	64.4777	56.9589	5
7	9	3	51.2228	44.0235	18
7	-9	4	157.916	66.9065	4
7	-9	4	118.083	62.2138	5
7	9	4	201.350	43.9319	18
-7	-9	-4	89.7450	56.8652	28

7	-9	5	255.321	62.8889	4
7	-9	5	113.546	51.0795	5
-7	-9	-5	159.244	55.4274	28
-7	-9	-6	2.91588	20.1165	15
7	-9	6	35.8848	34.9557	5
7	-9	6	58.5614	35.7705	4
7	10	-8	677.035	71.4034	18
7	10	-7	414.922	73.5546	18
-7	-10	7	90.3165	40.8892	16
-7	-10	6	166.170	60.3541	27
-7	-10	6	21.3234	42.8422	16
7	10	-5	2459.89	195.075	18
-7	-10	5	2097.01	161.577	3
-7	-10	5	2582.21	200.240	27
-7	-10	4	258.762	68.6690	22
7	-10	-4	372.313	85.1192	3
-7	-10	4	309.733	62.8356	3
7	10	-4	372.858	92.2764	18
-7	-10	4	260.796	69.8920	16
-7	-10	4	321.957	80.2733	27
7	10	-3	9055.67	522.757	18
-7	-10	3	9675.97	505.712	22
-7	-10	3	9246.46	534.344	27
7	-10	-3	8525.98	506.315	3
7	10	-2	2618.60	219.718	18
-7	-10	2	3240.61	243.673	27
-7	-10	2	2745.59	225.892	28
-7	-10	2	2672.31	205.859	22
7	10	-1	2950.01	234.234	18

7	-10	-1	3354.03	250.841	4
-7	-10	1	2848.64	242.239	28
-7	-10	1	2970.40	226.163	22
-7	-10	0	537.805	102.651	28
-7	-10	0	689.033	98.4959	22
7	10	0	740.010	104.386	18
7	-10	0	670.691	109.874	4
-7	-10	-1	455.093	88.3618	28
-7	-10	-1	407.820	81.7275	22
7	10	1	434.880	74.9942	18
7	-10	1	418.560	89.9894	4
7	-10	2	469.936	83.5527	4
7	10	2	496.125	66.2251	18
-7	-10	-2	383.348	75.2108	28
-7	-10	-3	303.635	66.0522	28
7	-10	3	213.801	60.2949	4
7	-10	4	79.1798	38.3023	4
7	11	-5	1609.99	129.608	18
7	11	-4	6.24981	40.1659	18
7	11	-3	5881.09	344.817	18
7	11	-2	255.235	56.0823	18
7	11	-1	2405.98	170.178	18
-7	-11	0	22.1014	36.4593	9
7	11	0	71.9339	33.6608	18
-7	-11	-1	15.8396	28.7451	9
-8	0	14	247.573	44.1171	22
8	0	-13	-45.1143	61.5474	20
-8	0	13	-52.9362	69.4814	29
8	0	-12	1223.34	159.242	20

-8	0	12	1853.78	185.964	29
8	0	-11	-15.2324	80.4299	20
8	0	-10	2627.59	265.236	20
8	0	-9	23.5404	47.8681	19
8	0	-9	25.1300	55.6768	11
8	0	-9	76.8979	156.285	17
8	0	-9	85.2548	88.3605	20
8	0	-8	45468.2	2337.89	11
8	0	-8	46597.6	2336.15	19
8	0	-7	27.0701	64.5510	11
8	0	-7	65.5604	61.1006	19
8	0	-7	70.9448	144.197	17
8	0	-6	23712.5	1205.09	19
8	0	-6	22730.1	1201.20	11
-8	0	6	20137.1	1195.88	22
8	0	-5	38.6743	60.0309	19
8	0	-5	-63.3915	157.782	17
8	0	-5	13.0294	56.1731	11
8	0	-4	802.170	121.569	11
8	0	-4	914.486	126.665	19
8	0	-4	662.599	114.848	26
8	0	-4	650.380	218.489	25
8	0	-4	670.800	257.866	17
8	0	-4	654.500	119.473	12
8	0	-3	-62.5258	56.8780	11
8	0	-3	-12.5079	61.0011	19
8	0	-3	-38.5806	70.4127	12
8	0	-3	179.165	182.071	17
8	0	-3	-58.4673	145.545	1

8	0	-3	-99.6376	77.0188	26
8	0	-3	-50.8053	163.270	25
8	0	-2	31013.6	1574.20	19
8	0	-2	29201.0	1564.45	12
8	0	-2	30526.4	1568.31	11
8	0	-2	29653.9	1563.10	14
8	0	-2	34005.0	2003.66	1
8	0	-2	31377.3	1579.75	26
8	0	-2	29721.0	1939.46	25
8	0	-1	12.4216	65.5937	19
8	0	-1	11.6872	60.5631	14
8	0	-1	24.2561	59.1112	12
8	0	-1	23.1941	56.5239	18
8	0	-1	13.3195	71.6243	26
8	0	-1	108.662	146.068	25
8	0	-1	46.4657	124.924	1
8	0	0	144540.	7363.51	1
8	0	0	145130.	7077.02	26
8	0	0	145092.	7051.81	13
8	0	0	145446.	7045.30	14
8	0	0	148931.	7557.83	25
8	0	0	148708.	7058.87	18
8	0	0	151176.	7062.89	19
8	0	1	14.9613	77.5242	26
8	0	1	-48.3236	66.1136	13
8	0	1	45.0026	53.6295	14
8	0	1	39.4354	69.4122	18
8	0	1	38.2056	69.6937	19
8	0	2	2509.37	241.195	18

```
-8   0  -2 3280.12 569.184  23
 8   0   2 2456.52 222.130  14
 8   0   2 2908.36 242.303  13
 8   0   3 85.4426 75.2220  18
 8   0   3 68.2424 54.2539  14
 8   0   3-12.5088 63.5918   6
-8   0  -3 130.200 147.933  23
 8   0   4 207.004 79.1319  14
 8   0   4 273.103 95.8452   6
-8   0  -4 194.396 186.548  23
-8   0  -5 64.6181 160.843  23
 8   0   5 93.9369 73.5690   7
 8   0   5-11.9490 72.8688   6
 8   0   5 41.7685 81.2871  18
 8   0   6 30684.0 1606.47   6
 8   0   6 31329.3 1600.60   7
 8   0   6 33382.4 1638.28  18
 8   0   7-15.5497 43.9919   7
 8   0   7-11.0961 61.7595  18
 8   0   9 5.58446 36.7767   5
 8   0   9 8.59558 39.0620  26
-8   0 -10-26.5449 33.3938  20
 8   1 -14 678.300 69.1549  12
-8  -1  14 808.331 81.2786  22
 8   1 -13 3106.54 211.356  20
 8  -1 -13 3516.70 220.193  20
-8  -1  13 2319.89 208.759  22
-8   1  13 2636.58 200.643  29
 8   1 -12 179.528 78.8970  20
```

8	-1	-12	59.7779	66.6358	20
-8	1	12	100.582	74.5793	29
-8	-1	12	11.8056	65.8022	29
8	1	-11	1177.59	168.070	20
8	-1	-11	783.717	143.476	20
8	-1	-11	991.993	122.821	11
8	1	-10	9749.75	586.825	19
8	-1	-10	9730.01	589.960	11
8	1	-10	9818.95	625.588	20
8	-1	-10	10012.0	630.516	20
8	1	-9	5153.05	391.996	20
8	-1	-9	5468.21	399.972	20
8	1	-9	5033.60	370.959	19
-8	1	9	3820.39	568.934	30
-8	-1	9	4699.30	612.986	30
8	1	-9	4923.79	692.029	17
8	-1	-9	4801.88	704.687	17
8	-1	-9	5173.93	373.558	11
8	-1	-9	4071.77	615.186	1
8	1	-8	131.241	94.3461	20
8	-1	-8	196.942	98.7055	20
8	1	-8	218.688	87.6947	19
-8	-1	8	175.682	178.541	30
-8	1	8	-59.3093	180.823	30
8	1	-8	-72.0832	163.810	17
8	-1	-8	76.0469	133.876	17
8	-1	-8	162.335	84.8820	11
8	-1	-8	72.1406	146.639	1
8	1	-7	2893.74	273.078	20

8	-1	-7	3013.29	278.201	20
8	1	-7	3050.72	270.008	19
-8	1	7	4072.61	558.794	30
8	1	-7	2896.90	515.044	17
8	-1	-7	2776.60	261.665	11
8	-1	-7	3122.57	540.285	1
8	1	-6	10992.1	660.758	19
-8	-1	6	12361.5	1106.17	30
-8	1	6	13250.6	1091.43	30
8	1	-6	10036.7	653.986	20
8	-1	-6	11267.0	666.607	20
-8	-1	6	9704.71	660.065	22
8	1	-6	11012.8	641.148	11
8	-1	-6	11458.1	667.085	11
8	1	-6	11047.8	1038.31	17
-8	-1	5	1566.18	184.060	22
8	1	-5	1749.39	194.335	19
8	-1	-5	1689.11	175.187	19
8	1	-5	1506.32	171.697	11
8	-1	-5	1958.57	413.341	1
8	1	-5	1882.07	395.442	17
8	-1	-5	1675.60	187.407	11
8	-1	-4	29017.3	1557.60	11
-8	-1	4	28815.1	1550.10	22
8	1	-4	30567.7	1569.18	19
8	-1	-4	29835.1	1539.66	19
8	-1	-4	29112.3	1567.18	12
8	1	-4	30299.1	1562.38	12
8	1	-4	30127.0	1547.35	11

8	-1	-4	31836.5	2058.23	1
8	1	-4	32906.3	1943.37	25
8	1	-4	32892.6	1562.48	26
8	1	-4	26304.4	1937.06	17
8	-1	-3	1855.35	373.656	1
8	-1	-3	2090.56	193.092	19
8	1	-3	2143.78	206.325	19
8	-1	-3	1816.39	196.410	12
8	1	-3	1650.92	181.634	11
8	1	-3	1720.34	189.823	12
8	-1	-3	1909.26	194.720	11
8	1	-3	2190.63	376.445	25
8	1	-3	2174.21	204.693	26
8	1	-2	693.048	113.187	18
8	-1	-2	705.829	112.382	19
8	1	-2	816.992	128.204	19
8	1	-2	628.139	245.822	25
8	1	-2	590.727	118.146	26
8	-1	-2	683.072	118.614	26
8	-1	-2	619.452	248.088	25
8	-1	-2	951.488	265.065	1
8	1	-2	795.077	242.972	1
8	-1	-2	728.911	121.914	14
8	1	-2	593.290	103.416	14
8	-1	-2	663.065	122.277	12
8	1	-2	716.492	120.067	12
8	-1	-2	591.201	113.824	11
8	1	-1	21100.0	1160.74	14
8	-1	-1	22349.0	1182.78	14

8	1	-1	22144.9	1175.77	12
8	-1	-1	21890.1	1181.94	12
8	1	-1	21785.0	1483.61	1
8	-1	-1	24278.7	1542.66	1
8	1	-1	21973.0	1181.90	18
8	-1	-1	21404.9	1160.57	18
8	-1	-1	22373.0	1175.83	19
8	1	-1	23148.8	1194.14	19
8	-1	-1	22345.7	1190.98	26
8	1	-1	23277.3	1198.88	26
8	1	-1	24013.4	1583.42	25
8	-1	-1	24560.4	1590.62	25
8	1	0	64948.4	3170.91	18
8	-1	0	64909.5	3154.10	18
8	1	0	63088.0	3166.34	19
8	-1	0	66698.4	3162.14	19
8	1	0	63349.4	3428.67	1
8	-1	0	64933.5	3511.77	1
8	-1	0	67476.0	3185.19	26
8	1	0	65063.8	3182.17	26
8	-1	0	64965.2	3163.66	13
8	-1	0	62452.9	3156.51	14
8	1	0	62893.9	3151.71	13
8	1	0	60801.5	3137.31	14
8	1	0	64764.8	3625.07	25
8	-1	0	67087.4	3632.34	25
8	-1	1	2071.68	200.171	19
8	-1	1	2133.60	204.062	18
8	1	1	1702.95	196.917	18

8	-1	1	2182.01	340.159	1
8	1	1	1789.93	370.967	25
8	-1	1	1751.99	378.784	25
8	1	1	1942.28	187.209	13
8	-1	1	1710.12	185.253	14
8	1	1	1817.14	175.858	14
8	-1	1	1794.38	189.816	13
8	1	1	1879.02	205.774	26
8	-1	1	1900.70	206.834	26
8	1	2	16992.9	964.477	26
8	-1	2	16674.8	959.257	26
8	1	2	17292.6	938.088	19
8	-1	2	17387.4	939.766	18
8	1	2	16522.7	944.145	18
8	1	2	14460.0	1297.02	25
8	-1	2	17536.3	1368.98	25
8	1	2	16905.1	921.521	13
8	-1	2	16326.6	921.827	14
8	-1	2	17702.0	940.940	13
8	1	2	16106.3	901.656	14
-8	1	-2	20866.8	1501.50	23
8	1	3	14102.6	840.536	26
8	-1	3	14320.5	840.471	26
8	-1	3	14039.3	808.069	18
8	1	3	11499.2	1169.14	25
8	-1	3	14783.8	1262.85	25
8	-1	3	14437.5	800.066	6
8	-1	3	14430.0	794.561	14
8	1	3	13427.0	769.743	14

-8	1	-3	15312.8	1232.84	23
8	-1	4	31610.8	1634.46	26
8	-1	4	29860.5	1589.65	18
8	1	4	30155.4	1594.24	18
8	1	4	28455.5	2109.39	25
8	-1	4	29481.0	2108.05	25
8	-1	4	30766.1	1574.91	14
8	-1	4	30983.2	1579.10	6
8	1	4	29263.0	1559.89	6
8	-1	4	32283.5	1586.32	7
-8	-1	-4	29266.4	2037.32	23
-8	1	-4	30370.7	2055.25	23
8	-1	5	4261.32	308.730	6
8	1	5	3830.10	289.764	6
8	-1	5	3759.78	294.312	7
8	1	5	3698.10	306.840	18
-8	1	-5	3852.58	574.818	23
-8	-1	-5	4924.12	651.135	23
8	-1	6	13358.2	737.372	6
8	1	6	12449.5	713.965	6
8	-1	6	13245.9	726.779	7
8	1	6	12489.7	707.884	7
8	-1	6	13653.2	754.190	18
8	1	6	13084.0	746.238	18
8	-1	7	424.207	107.323	26
8	1	7	526.734	104.828	18
8	-1	7	344.869	76.9556	7
8	1	7	405.695	68.6516	7
8	-1	8	1682.65	154.163	5

8	-1	8	1463.25	124.870	7
8	1	8	1792.74	178.903	26
8	-1	8	1474.76	163.793	26
8	-1	9	291.545	56.4200	5
8	1	9	312.969	56.0017	5
8	-1	9	158.785	51.8132	26
8	1	9	297.407	66.3021	26
-8	1	-10	2139.81	140.858	20
-8	-1	-10	2566.32	151.874	20
-8	-2	14	-4.71525	27.1046	22
-8	-2	13	1727.72	159.242	22
-8	2	13	1401.27	139.064	29
8	2	-12	391.693	93.5237	20
8	-2	-12	474.777	95.9629	20
8	-2	-12	387.636	74.0416	11
-8	2	12	539.072	105.978	29
-8	-2	12	215.008	75.6602	29
8	2	-11	6914.24	455.671	19
8	-2	-11	7653.01	467.834	11
8	2	-11	7770.26	490.812	20
8	-2	-11	7493.25	487.369	20
8	2	-10	68.6896	73.9019	19
8	2	-10	61.1720	72.9232	20
8	-2	-10	-15.3831	71.6676	20
8	-2	-10	83.6559	75.0023	11
8	2	-9	30.8001	66.4240	19
8	-2	-9	77.2265	135.934	17
8	2	-9	96.0833	86.1300	20
8	-2	-9	31.9704	76.2156	20

8	-2	-9	30.8029	70.0199	11
8	2	-8	21816.0	1203.34	20
8	-2	-8	21932.0	1202.55	20
8	2	-8	22457.5	1206.37	19
-8	-2	8	23608.4	1602.17	30
-8	2	8	23076.5	1600.51	30
8	2	-8	19188.7	1588.74	17
8	-2	-8	22020.0	1709.06	17
8	-2	-8	20760.0	1623.54	1
8	-2	-8	21802.6	1201.50	11
8	2	-7	17571.8	994.134	20
8	-2	-7	18063.1	995.353	20
-8	-2	7	15401.3	1001.91	22
8	2	-7	18380.2	1006.57	19
-8	-2	7	18728.9	1378.06	30
-8	2	7	20206.0	1401.52	30
8	2	-7	15640.9	1347.65	17
8	-2	-7	20578.1	1518.51	17
8	-2	-7	19039.4	1463.03	1
8	-2	-7	17747.4	997.985	11
8	2	-6	569.502	125.202	19
-8	-2	6	560.384	246.574	30
-8	-2	6	686.120	137.624	22
8	-2	-6	606.747	121.535	11
8	2	-6	382.170	196.099	17
8	-2	-6	548.115	242.727	17
-8	-2	5	7963.99	533.837	22
8	2	-5	8464.89	537.864	19
8	2	-5	8234.30	521.029	12

8	-2	-5	8181.32	872.729	1
8	-2	-5	7799.73	523.989	11
8	2	-5	6469.78	809.626	17
-8	-2	4	1395.36	169.147	22
8	2	-4	1554.01	182.563	19
8	2	-4	1387.89	157.630	18
8	2	-4	1184.85	154.722	12
8	-2	-4	1747.68	369.103	1
8	-2	-4	1186.09	159.102	11
8	-2	-4	1179.56	314.643	17
-8	-2	3	104183.	5108.60	22
8	-2	-3	100958.	5109.13	11
8	2	-3	104983.	5100.88	18
8	2	-3	104514.	5120.92	19
8	2	-3	113329.	5548.32	25
8	-2	-3	107936.	5514.85	25
8	2	-3	113244.	5109.87	26
8	-2	-3	106669.	5101.38	26
8	-2	-3	116465.	5639.67	1
8	-2	-3	103395.	5113.96	14
8	2	-3	103105.	5088.86	12
8	-2	-3	95330.3	5570.44	17
8	-2	-2	9913.06	899.688	1
8	2	-2	9832.00	586.260	26
8	-2	-2	10299.5	591.996	26
8	2	-2	9026.07	862.693	25
8	-2	-2	9961.11	882.029	25
8	-2	-2	9832.64	593.866	14
8	-2	-2	9303.48	585.451	11

8	-2	-2	9085.91	582.598	12
8	2	-2	9021.18	568.966	12
8	2	-2	9737.26	588.268	18
8	-2	-2	9906.24	566.311	19
-8	-2	2	9636.44	585.616	22
8	-2	-1	2169.60	385.829	1
8	2	-1	1537.43	313.007	1
8	2	-1	1759.97	193.572	18
8	-2	-1	1933.32	183.845	19
8	-2	-1	1688.94	353.719	25
8	2	-1	1696.71	196.266	26
8	-2	-1	1757.70	191.978	26
8	2	-1	1687.49	372.493	25
8	-2	-1	2091.85	200.709	13
8	-2	-1	1710.38	188.491	14
8	-2	-1	1710.00	192.667	12
-8	-2	1	1829.93	189.903	22
-8	-2	0	73.4873	61.1876	22
8	2	0	67.5594	60.0686	19
8	-2	0	180.020	78.5651	14
8	-2	0	97.7340	121.739	1
8	2	0	126.562	74.4278	26
8	-2	0	54.3031	66.3579	26
8	2	0	229.869	175.258	25
8	-2	0	-55.5942	149.550	25
8	-2	0	76.2865	55.6088	18
8	2	0	97.7662	68.2569	18
8	2	1	10639.9	966.059	25
8	-2	1	10199.3	931.054	25

8	2	1	9314.44	593.495	18
8	-2	1	9483.96	575.050	18
8	2	1	9533.74	587.412	19
8	-2	1	7926.50	786.857	1
8	-2	1	11123.9	615.883	26
8	2	1	10027.5	607.204	26
-8	-2	-1	9037.77	571.159	22
-8	-2	-1	12319.2	1117.33	23
-8	2	-1	10003.9	1137.53	23
8	2	1	9191.26	561.114	13
8	-2	1	9307.10	581.878	14
8	2	2	1039.63	144.776	19
8	-2	2	1039.96	141.851	18
8	-2	2	1088.81	143.936	14
8	2	2	901.144	124.873	13
8	-2	2	754.593	259.953	25
8	2	2	1305.72	335.072	25
8	2	2	904.125	153.468	26
8	-2	2	960.547	154.443	26
-8	-2	-2	1073.74	141.168	22
-8	-2	-2	1061.64	319.780	23
-8	2	-2	1614.59	379.961	23
-8	-2	-3	8889.22	894.741	23
-8	2	-3	9652.42	920.390	23
-8	-2	-3	8366.30	519.662	22
8	2	3	8096.15	555.241	26
8	-2	3	8493.04	556.950	26
8	2	3	8554.95	545.345	18
8	-2	3	8394.95	530.563	18

8	-2	3	8697.36	532.329	6
8	-2	3	8478.36	533.694	7
8	2	3	7492.79	872.734	25
8	-2	3	7023.97	828.127	25
8	-2	3	8132.12	526.168	14
-8	-2	-4	129.229	173.721	23
-8	2	-4	185.040	188.034	23
-8	-2	-4	-12.2620	68.2693	22
8	-2	4	82.3469	71.1335	18
8	-2	4	25.9818	73.5195	14
8	-2	4	39.6461	68.4966	7
8	2	4	33.0077	61.2626	6
8	-2	4	79.1697	70.9707	6
8	2	4	72.6440	147.643	25
8	-2	4	68.5630	120.684	25
8	-2	4	82.7278	87.3790	26
8	2	4	-29.0583	72.3491	18
8	-2	5	4482.43	343.001	18
8	2	5	4711.47	351.640	18
8	-2	5	4222.09	333.322	6
8	2	5	4366.97	312.993	6
8	-2	5	4699.32	335.225	7
8	-2	5	5319.71	383.404	26
8	2	6	4839.90	336.469	18
8	2	6	4420.61	300.851	6
8	-2	6	4791.67	324.280	7
8	2	6	4364.81	295.582	7
8	-2	6	4537.55	354.189	26
8	2	7	2157.90	190.757	18

8	-2	7	1977.97	202.927	26
8	-2	7	2076.70	194.405	5
8	-2	7	1888.92	171.939	7
8	2	8	933.827	120.679	26
8	-2	8	715.932	106.469	26
8	-2	8	835.792	107.385	5
8	-2	8	635.179	82.3595	7
-8	2	-9	-4.99796	34.0690	20
8	2	9	-7.67274	38.9833	26
8	-2	9	27.7607	36.1254	5
8	2	9	8.65008	28.1416	5
-8	-2	-10	-12.3697	19.8823	20
8	-3	-14	190.324	31.7752	12
-8	-3	14	181.004	35.4998	22
8	-3	-13	2948.02	189.580	11
-8	-3	13	3712.15	240.198	22
8	-3	-12	713.314	99.0017	11
-8	-3	12	920.986	135.501	22
-8	3	12	867.245	116.214	29
8	3	-11	1046.00	131.496	19
8	-3	-11	789.326	124.255	11
-8	-3	11	635.691	141.203	22
8	-3	-11	872.651	127.314	20
-8	-3	11	539.198	103.746	29
8	3	-10	1701.48	198.760	19
8	3	-10	2070.32	208.645	20
8	-3	-10	1936.46	201.622	20
-8	-3	10	1811.42	224.363	22
8	-3	-10	1774.20	200.947	11

8	3	-9	20523.7	1120.04	19
8	-3	-9	21251.0	1126.33	11
8	3	-9	20034.8	1100.54	20
8	-3	-9	21009.9	1106.21	20
-8	-3	9	17305.6	1132.36	22
-8	-3	8	743.523	165.076	22
8	-3	-8	813.392	137.192	20
8	3	-8	986.568	157.736	19
-8	-3	8	536.712	214.059	30
-8	3	8	962.246	278.015	30
8	3	-8	376.515	204.613	17
8	-3	-8	511.746	259.669	17
8	-3	-8	799.172	142.772	11
8	-3	-8	566.192	224.152	1
-8	-3	7	4681.77	382.175	22
8	3	-7	5160.08	376.927	19
-8	-3	7	4057.49	560.277	30
-8	3	7	4952.23	610.271	30
8	-3	-7	4387.21	636.441	17
8	3	-7	2503.85	491.322	17
8	-3	-7	4349.72	623.281	1
8	-3	-7	4396.67	355.703	11
8	-3	-6	6482.50	439.746	11
-8	-3	6	6170.58	448.486	22
8	3	-6	6747.63	446.508	19
8	3	-6	5984.97	401.865	18
8	3	-6	6078.92	409.953	12
8	-3	-6	7861.70	829.193	17
8	3	-6	3335.56	576.055	17

-8	-3	6	2850.88	529.649	30
8	-3	-6	7048.43	783.884	1
-8	-3	5	2275.43	228.687	22
8	3	-5	1955.59	217.097	19
8	3	-5	1693.11	193.941	18
8	-3	-5	2074.31	221.991	12
8	3	-5	2152.04	202.782	12
8	-3	-5	1863.02	390.796	17
8	-3	-5	2151.25	222.549	11
-8	3	5	1771.08	407.103	30
8	-3	-4	9168.65	587.224	11
8	3	-4	8713.03	570.435	18
8	-3	-4	8405.36	905.473	17
8	-3	-4	10290.8	963.812	1
8	3	-4	9703.62	561.608	12
8	-3	-4	9491.98	590.409	12
-8	-3	4	9448.41	597.164	22
8	-3	-3	1735.61	192.011	12
8	3	-3	1413.30	157.137	12
8	3	-3	1523.67	180.752	18
8	3	-3	991.247	265.862	25
8	3	-3	1488.42	173.040	26
8	-3	-3	1705.99	367.504	1
8	-3	-3	900.946	288.030	17
-8	-3	3	1565.76	188.688	22
-8	-3	2	28669.0	1519.68	22
8	3	-2	28837.4	1524.95	19
8	3	-2	27537.4	1517.33	18
8	-3	-2	29640.0	1519.83	11

8	-3	-2	28229.2	1845.00	25
8	3	-2	29350.5	1505.91	26
8	-3	-2	29775.3	1502.41	26
8	3	-2	30184.3	1881.17	25
8	-3	-2	30615.6	1521.20	13
8	3	-2	28668.1	1485.23	12
8	-3	-2	28095.3	1509.48	12
8	-3	-2	30795.9	1972.96	1
8	-3	-1	21302.4	1509.12	2
8	-3	-1	26387.1	1656.18	1
8	3	-1	22965.8	1229.33	18
8	3	-1	22739.7	1228.03	19
-8	-3	1	21725.0	1215.25	22
8	3	-1	23069.1	1217.89	26
8	-3	-1	23576.8	1212.14	26
8	3	-1	20182.5	1533.04	25
8	-3	-1	22954.6	1568.40	25
8	-3	-1	24097.6	1218.06	13
8	-3	-1	21728.7	1213.58	11
8	-3	-1	22791.8	1213.82	12
8	3	0	946.979	151.178	18
-8	-3	0	1231.59	158.876	22
8	-3	0	1169.84	135.194	18
8	3	0	1536.23	173.287	19
8	3	0	879.766	123.079	13
8	-3	0	1329.63	161.873	14
8	-3	0	1316.58	158.127	13
8	3	0	1090.44	152.942	26
8	-3	0	1018.05	144.405	26

8	3	0	1185.42	302.600	25
8	-3	0	1122.75	292.189	25
8	-3	0	950.133	273.236	1
8	-3	0	702.284	228.257	2
8	-3	1	14547.9	805.029	26
8	3	1	15234.6	1193.40	25
8	-3	1	14456.3	1162.09	25
-8	-3	-1	12537.5	786.416	22
-8	-3	-1	17776.9	1333.69	23
-8	3	-1	14496.7	1236.29	23
8	-3	1	15111.2	798.746	6
8	-3	1	12934.4	1101.43	2
8	-3	1	12553.0	1069.31	1
8	-3	1	13340.0	772.005	18
8	3	1	13543.6	793.804	19
8	3	1	14400.4	810.997	26
8	3	1	12721.9	755.621	13
8	-3	1	14263.6	801.035	14
8	-3	1	15564.7	806.820	13
8	-3	2	273.189	87.7536	26
8	3	2	63.3976	128.903	25
8	-3	2	118.509	147.538	25
8	3	2	122.999	82.7656	26
-8	-3	-2	61.9414	154.224	23
-8	3	-2	179.765	161.145	23
-8	-3	-2	40.1225	56.1760	22
8	-3	2	135.557	67.5527	18
8	3	2	92.9807	67.6051	19
8	-3	2	-47.5006	127.765	1

8	-3	2	169.389	140.594	2
8	-3	2	91.3453	76.2773	6
8	3	2	120.446	82.4832	18
8	3	2	40.1480	46.9090	13
8	-3	2	80.1374	70.6344	14
-8	-3	-3	4678.39	631.288	23
-8	3	-3	4115.81	579.019	23
-8	-3	-3	4836.77	359.369	22
8	-3	3	4809.17	369.310	26
8	3	3	4926.21	380.349	26
8	-3	3	5371.26	367.528	18
8	3	3	5364.12	384.081	18
8	-3	3	5033.97	365.684	7
8	3	3	3591.35	588.645	25
8	-3	3	4498.31	616.138	25
8	-3	3	5179.85	369.827	14
-8	3	-4	57.3767	116.960	23
-8	-3	-4	249.156	167.708	23
-8	-3	-4	144.289	72.3542	22
8	-3	4	159.380	84.7737	18
8	-3	4	213.965	90.5960	14
8	-3	4	185.289	89.6715	7
8	3	4	296.712	72.3476	6
8	3	4	205.265	170.545	25
8	-3	4	250.353	168.511	25
8	3	4	230.207	99.2687	26
8	-3	4	150.083	78.2945	26
8	3	4	215.136	97.1000	18
8	-3	5	4601.25	363.008	4

8	-3	5	5919.31	392.502	6
8	3	5	5329.03	341.816	6
8	-3	5	5732.79	382.386	7
8	3	5	5795.12	412.620	26
8	-3	5	5770.07	400.454	26
8	3	5	5332.05	382.419	18
8	-3	6	4990.74	346.125	5
8	-3	6	5377.04	341.429	7
8	-3	6	4840.68	350.367	26
8	3	6	5174.23	369.451	26
8	3	6	4934.54	340.782	18
8	-3	7	2048.74	185.757	5
8	-3	7	1784.84	165.554	7
8	-3	7	1996.93	183.479	26
8	3	7	1851.34	191.268	26
8	3	7	1695.76	163.545	18
8	-3	8	882.580	108.395	5
8	-3	8	681.090	87.4552	7
8	3	8	893.261	96.2496	18
8	3	8	924.447	115.953	26
-8	-3	-9	23.7748	30.9923	20
-8	3	-9	31.0864	21.3757	20
8	-3	9	96.5066	37.9716	5
8	3	9	36.3296	22.4711	5
-8	-4	13	1818.53	145.784	22
8	-4	-13	1503.37	124.563	12
8	-4	-13	1666.90	116.937	11
-8	4	12	-23.3188	46.8232	29
8	-4	-12	144.807	58.4554	11

-8	-4	12	145.687	73.3206	22
8	4	-12	157.367	56.6146	19
-8	-4	11	519.228	115.948	22
8	-4	-11	502.395	103.073	11
-8	4	11	387.255	93.5110	29
8	4	-11	579.900	104.598	19
8	4	-10	14.3747	70.0563	19
-8	-4	10	32.2757	88.3110	22
8	-4	-10	14.3211	71.2959	11
8	4	-9	893.037	151.935	19
-8	-4	9	708.009	151.424	22
8	-4	-9	943.287	159.183	11
8	4	-8	3434.54	313.296	19
8	-4	-8	3367.84	309.417	11
-8	-4	8	3975.22	338.874	22
-8	-4	7	1556.28	207.260	22
8	4	-7	1494.35	191.952	19
8	-4	-7	1417.53	348.014	17
-8	-4	7	633.901	218.378	30
-8	4	7	952.324	264.523	30
8	-4	-7	1476.61	197.397	11
8	-4	-7	1032.53	306.265	1
8	-4	-6	5221.49	378.045	11
-8	-4	6	5294.48	390.613	22
8	-4	-6	3973.81	581.338	17
8	-4	-6	5340.65	670.306	1
-8	4	6	3778.67	548.016	30
8	-4	-6	4972.85	371.209	12
-8	-4	5	7201.02	416.946	22

8	-4	-5	8790.20	843.655	1
8	-4	-5	5986.06	683.783	17
8	-4	-5	7174.10	395.192	12
8	-4	-5	7675.33	411.188	14
-8	-4	5	334.599	279.222	30
-8	4	5	2144.98	449.076	30
8	-4	-4	8184.13	482.783	11
8	4	-4	8922.84	505.212	19
8	4	-4	9142.92	496.993	18
-8	-4	4	8571.31	502.038	22
8	-4	-4	9495.70	502.943	14
8	-4	-4	9127.39	490.321	12
8	-4	-3	3333.91	286.077	11
8	4	-3	3311.81	287.650	18
8	4	-3	3295.13	288.611	19
8	-4	-3	3388.42	280.453	12
8	-4	-3	3510.72	280.274	13
8	-4	-3	3114.22	284.146	14
8	-4	-3	3988.12	568.856	1
-8	-4	3	3209.83	292.934	22
-8	-4	2	4836.60	365.984	22
8	-4	-2	4414.32	350.779	11
8	4	-2	4560.52	361.049	18
8	4	-2	4480.17	357.414	19
8	4	-2	3766.88	520.946	25
8	-4	-2	4509.39	533.944	25
8	4	-2	4873.78	340.680	26
8	-4	-2	5132.27	336.985	26
8	-4	-2	4624.15	618.559	1

8	-4	-2	5115.73	351.137	13
8	-4	-2	4630.01	351.795	14
8	-4	-2	4669.48	344.420	12
8	-4	-2	4656.71	326.971	7
8	-4	-1	8196.28	487.098	7
8	-4	-1	7478.94	473.678	6
8	-4	-1	6995.15	760.882	2
8	-4	-1	7957.91	817.146	1
8	4	-1	7120.75	503.321	18
8	4	-1	7499.58	500.194	19
8	-4	-1	6457.44	691.336	25
8	4	-1	7658.59	483.443	26
8	-4	-1	7218.97	470.474	26
8	4	-1	7622.56	754.484	25
8	-4	-1	8320.47	495.817	13
8	-4	-1	8022.05	501.998	11
8	-4	-1	7954.29	504.827	14
-8	-4	1	7387.52	502.539	22
-8	-4	0	25310.7	1763.84	23
-8	-4	0	22040.6	1207.96	22
8	-4	0	21315.2	1163.64	5
8	4	0	22721.7	1207.24	19
8	4	0	22903.6	1197.90	26
8	-4	0	23125.8	1187.34	26
8	4	0	19905.7	1516.13	25
8	-4	0	20842.1	1509.25	25
8	-4	0	22342.7	1599.03	2
8	-4	0	23702.4	1612.71	1
8	-4	0	23754.7	1198.71	13

8	-4	0	23623.9	1214.38	14
8	-4	0	24131.6	1192.64	6
8	-4	0	23564.1	1196.54	7
8	4	1	3232.42	266.866	26
8	-4	1	2516.86	417.766	25
8	-4	1	3118.47	254.717	26
8	4	1	2939.02	458.866	25
-8	-4	-1	2717.17	473.923	23
-8	4	-1	2256.11	448.591	23
-8	-4	-1	2949.07	264.378	22
8	-4	1	2907.60	247.049	5
8	4	1	2897.30	274.805	18
8	4	1	2991.59	260.055	19
8	-4	1	3221.01	475.646	1
8	-4	1	2674.67	462.424	2
8	-4	1	2820.79	264.724	13
8	-4	1	3164.81	258.682	6
8	-4	1	2861.29	253.838	7
8	4	2	373.239	98.7795	26
8	-4	2	283.096	84.8069	26
8	4	2	59.2162	148.007	25
8	-4	2	266.099	153.551	25
-8	-4	-2	578.629	242.799	23
-8	-4	-2	227.634	88.9672	22
8	4	2	414.216	96.7034	19
8	-4	2	323.854	94.9688	6
8	-4	2	336.174	95.6245	7
8	-4	2	241.957	83.9501	5
8	-4	2	311.370	183.168	1

8	-4	2	254.643	171.679	2
8	4	2	198.086	92.5901	18
8	-4	3	181.958	75.4711	26
8	4	3	307.139	165.491	25
8	-4	3	162.258	145.800	25
8	4	3	251.081	88.1616	26
-8	-4	-3	353.533	198.863	23
-8	-4	-3	321.644	92.7121	22
8	4	3	162.209	84.1122	18
8	-4	3	263.744	88.5189	6
8	-4	3	261.117	162.832	2
8	-4	3	186.434	86.2480	5
8	-4	4	4613.78	362.188	4
8	-4	4	5764.29	387.635	5
8	-4	4	5042.11	366.106	26
8	4	4	5357.51	386.645	26
8	4	4	5541.15	385.555	18
-8	-4	-4	5216.73	381.075	22
8	-4	4	5012.92	373.050	6
8	-4	4	5675.96	380.984	7
8	4	5	12156.1	690.567	18
8	-4	5	11422.2	680.923	26
8	4	5	12610.9	710.342	26
8	-4	5	12553.5	692.451	7
8	-4	5	10961.9	679.992	4
8	-4	6	776.742	108.390	26
8	4	6	863.462	128.010	26
8	-4	6	681.104	112.873	7
8	4	6	858.839	118.038	18

8	-4	6	756.514	125.777	5
8	-4	6	698.890	112.310	4
8	-4	7	90.7872	57.6357	7
8	4	7	61.1945	49.9320	18
8	-4	7	124.275	62.3290	5
8	4	7	80.0644	58.5577	26
8	-4	8	475.762	68.9538	7
8	-4	8	451.241	74.7281	5
8	4	8	419.158	71.3038	26
8	-4	9	986.042	86.4715	5
8	-5	-12	2654.10	191.962	11
8	5	-12	2633.29	191.660	19
-8	5	11	232.547	60.6939	29
8	5	-11	544.497	94.8141	19
8	-5	-11	450.674	92.2171	11
8	-5	-11	360.165	80.1354	12
-8	-5	10	1043.26	157.781	22
-8	5	10	427.477	98.1010	29
8	-5	-10	511.674	122.592	12
8	-5	-10	1216.51	128.631	14
8	5	-10	1385.39	159.917	19
8	-5	-10	1089.50	149.934	11
-8	-5	9	552.257	128.651	22
8	-5	-9	426.918	120.730	12
8	-5	-9	821.172	136.598	11
-8	-5	8	-87.2046	97.0689	22
8	-5	-8	41.9578	75.2006	12
8	-5	-8	64.6893	82.1662	11
8	-5	-7	623.070	123.971	14

8	-5	-7	401.994	120.448	12
8	5	-7	417.866	115.941	19
-8	-5	7	394.168	121.300	22
8	5	-6	652.337	135.021	19
8	-5	-6	362.265	205.674	17
8	-5	-6	573.077	290.905	1
8	-5	-6	639.482	108.065	13
8	-5	-6	681.738	127.714	12
8	-5	-6	596.300	121.967	14
-8	-5	6	786.838	152.810	22
8	-5	-5	7552.78	514.939	11
8	5	-5	7346.40	511.357	19
-8	-5	5	8264.67	537.123	22
8	-5	-5	8074.65	864.720	1
8	-5	-5	8278.80	509.620	14
8	-5	-5	7755.06	474.053	13
8	-5	-5	8164.45	503.087	12
8	5	-4	35707.2	1910.18	19
-8	-5	4	36588.6	1925.71	22
8	-5	-4	38080.8	1903.60	14
8	-5	-4	38110.3	1891.81	12
8	-5	-4	37050.0	1866.67	13
8	-5	-4	36084.2	1909.21	11
8	-5	-3	1039.19	154.740	11
-8	-5	3	913.882	156.723	22
8	5	-3	1012.12	156.067	19
8	-5	-3	1114.54	323.112	1
8	-5	-3	1087.90	140.690	13
8	-5	-3	997.852	146.355	14

8	-5	-3	1223.00	150.632	12
8	5	-3	857.009	152.944	18
8	5	-2	4488.15	350.924	18
8	5	-2	4823.08	352.768	19
8	-5	-2	4789.77	345.841	11
8	-5	-2	5229.10	356.327	14
8	-5	-2	4868.44	332.479	12
8	-5	-2	5432.36	337.048	13
8	-5	-2	4805.54	626.948	1
-8	-5	2	4514.46	354.652	22
8	-5	-2	4291.75	302.656	7
8	-5	-1	8365.69	492.208	6
8	-5	-1	7898.40	491.793	7
8	-5	-1	6470.39	779.911	2
8	-5	-1	8775.95	885.413	1
8	5	-1	7798.20	518.054	19
8	-5	-1	7990.22	511.785	11
8	-5	-1	8574.51	526.537	14
8	-5	-1	9314.44	517.594	13
8	5	-1	8314.27	495.533	26
-8	-5	1	7774.18	532.020	22
8	-5	0	326.209	94.7489	7
8	-5	0	260.735	78.7835	6
8	-5	0	586.454	229.880	2
8	-5	0	497.048	228.147	1
-8	-5	0	291.395	77.6943	28
8	5	0	433.290	91.1415	26
8	-5	0	324.131	77.8601	26
-8	-5	0	365.610	191.971	23

-8	-5	0	320.581	109.664	22
8	5	0	452.739	102.644	19
8	-5	0	239.801	71.1480	5
8	-5	0	426.031	98.5939	13
8	-5	0	260.404	86.0922	14
8	-5	1	422.090	104.117	14
8	5	1	133.961	64.0499	26
8	-5	1	216.297	69.5054	26
-8	-5	-1	208.289	140.424	23
-8	-5	-1	149.591	85.3439	22
8	5	1	298.263	88.0963	19
8	-5	1	299.436	192.749	1
8	-5	1	266.947	179.754	2
8	-5	1	347.856	91.0589	6
8	-5	1	253.955	90.9850	7
8	-5	1	243.096	74.2768	5
8	5	1	187.676	97.2905	18
-8	-5	-2	1610.80	185.465	22
-8	-5	-2	1486.24	334.879	23
8	-5	2	1239.24	154.884	4
8	-5	2	1562.24	172.466	5
8	5	2	1339.63	179.194	18
8	5	2	1224.16	156.617	19
8	5	2	1537.71	170.872	26
8	-5	2	1463.36	152.145	26
8	-5	2	1025.06	302.161	2
8	-5	2	1594.49	179.501	14
8	-5	2	1697.29	175.540	6
8	-5	2	1271.27	165.131	7

-8	-5	-3	4928.44	367.552	22
8	5	3	5057.35	359.676	26
8	5	3	4895.05	371.071	18
8	-5	3	4496.24	354.337	4
8	-5	3	5388.94	368.359	5
8	-5	3	5572.66	378.309	14
8	-5	3	5234.99	370.250	7
-8	-5	-4	106.844	80.3636	22
8	5	4	38.4326	79.2600	18
8	-5	4	75.8825	71.6378	5
8	-5	4	89.5471	74.7548	4
8	5	4	72.5654	57.7487	26
8	5	5	87.5578	64.0265	18
8	-5	5	100.280	68.9983	7
8	-5	5	154.280	81.0053	5
8	-5	5	35.6283	56.7600	4
8	5	5	109.657	59.1125	26
8	-5	6	228.545	74.7509	7
8	5	6	128.631	58.3467	18
8	5	6	174.570	58.8456	26
8	-5	7	2128.31	180.468	5
8	-5	7	2219.65	175.989	7
8	5	7	2125.48	157.134	18
8	-5	8	19.7173	36.2079	7
8	-5	8	115.455	47.4955	5
-8	-6	12	179.365	48.6289	22
8	-6	-12	353.265	54.8663	11
8	6	-12	319.283	52.3541	19
8	-6	-11	1246.61	128.031	11

-8	-6	11	1036.79	126.456	22
8	-6	-10	401.840	93.3778	11
8	-6	-9	1669.97	192.046	11
8	6	-9	1558.18	180.972	19
8	-6	-8	3810.54	273.254	14
8	-6	-8	3704.68	277.241	12
8	6	-8	3651.96	309.056	19
8	-6	-7	4383.57	319.277	14
8	-6	-7	4697.38	326.538	12
8	-6	-7	4541.81	354.320	11
8	6	-7	4256.88	351.074	19
8	6	-6	350.708	102.484	19
8	-6	-6	232.978	87.9904	11
8	-6	-6	387.548	94.6385	12
8	-6	-6	530.585	106.180	14
-8	-6	6	400.667	96.6154	27
-8	-6	6	223.673	104.728	22
8	-6	-5	2705.85	238.468	12
8	-6	-5	2902.83	251.996	14
8	6	-5	2652.41	263.633	19
8	-6	-5	3101.21	273.224	11
-8	-6	5	3039.23	284.065	22
-8	-6	5	2867.00	254.623	27
8	-6	-5	3030.19	494.478	1
8	-6	-4	7854.17	520.892	11
-8	-6	4	8558.41	554.638	22
8	6	-4	7115.19	518.855	19
8	-6	-4	7904.27	506.693	14
8	-6	-4	8422.61	500.126	12

8	-6	-4	8534.99	876.253	1
-8	-6	3	1906.24	217.665	22
8	6	-3	1540.17	206.758	18
8	6	-3	1825.06	205.725	19
8	-6	-3	1634.78	185.899	14
8	-6	-3	1639.33	191.302	11
8	6	-2	52965.3	2801.05	19
8	-6	-2	59973.5	3367.06	1
8	-6	-2	56660.5	2801.93	11
8	-6	-2	58366.7	2800.75	14
8	-6	-2	58235.3	2765.52	13
-8	-6	2	53442.1	2823.91	22
-8	-6	1	1427.28	193.129	22
8	6	-1	1023.27	150.454	19
8	-6	-1	1469.33	351.494	1
8	-6	-1	1390.66	149.180	7
8	-6	-1	1686.02	181.877	14
-8	-6	1	1174.42	154.982	28
-8	-6	0	4984.90	372.612	22
-8	-6	0	4969.97	357.469	28
8	6	0	4713.62	374.007	18
8	6	0	4654.98	343.172	19
8	-6	0	4816.37	326.713	6
8	-6	0	4620.55	332.347	7
8	-6	0	4333.31	318.493	5
8	-6	0	5754.74	367.767	14
8	-6	0	4404.63	642.647	2
8	-6	0	5391.04	680.901	1
8	6	1	75.8931	87.2482	18

-8	-6	-1	-44.7875	78.8254	22
8	6	1	11.6877	58.1815	19
8	-6	1	23.5721	59.8808	7
-8	-6	-1	-13.9166	58.3050	28
8	-6	1	-33.5561	61.2065	5
8	-6	1	12.6974	53.1972	4
8	-6	1	25.7506	65.4148	14
8	-6	2	1820.43	190.845	14
8	6	2	1906.04	202.337	18
8	6	2	1831.97	176.843	19
8	-6	2	1551.30	180.276	4
-8	-6	-2	1938.78	205.502	22
8	-6	2	1926.11	194.502	7
8	-6	2	1827.65	185.744	5
8	6	3	8452.86	540.326	18
8	-6	3	9277.05	539.647	7
8	-6	3	9238.15	542.045	5
8	-6	3	7827.08	531.641	4
-8	-6	-3	9104.96	554.507	22
8	6	4	280.288	82.9998	18
-8	-6	-4	212.709	83.2427	22
8	-6	4	232.699	87.6808	5
8	-6	4	277.261	82.1100	4
8	-6	5	7660.75	475.407	7
8	-6	5	8546.26	491.385	5
8	-6	5	7900.51	484.237	4
8	6	5	7813.86	466.609	18
8	-6	6	49.9880	58.0955	7
8	6	6	57.6438	43.7345	18

8	-6	7	570.501	81.6714	7
8	-6	7	771.898	94.4585	5
8	-6	8	142.018	38.3172	5
8	7	-11	70.8220	40.3900	19
-8	-7	11	54.5339	38.5600	22
8	-7	-11	115.560	45.9966	11
-8	-7	10	51.7781	53.4396	22
8	7	-10	86.8371	52.3049	19
8	7	-9	130.588	69.1321	19
-8	-7	9	44.6160	62.1625	22
8	-7	-9	42.6884	62.3791	11
8	7	-8	564.366	113.710	19
8	-7	-8	550.173	112.399	11
-8	-7	8	642.909	124.510	22
-8	-7	7	4639.29	338.175	27
-8	-7	7	4832.22	371.035	22
8	-7	-7	5881.30	380.714	11
8	7	-7	5169.59	370.970	19
-8	-7	6	798.954	123.723	27
8	7	-6	820.568	140.773	19
8	-7	-6	789.682	131.567	11
-8	-7	5	4010.18	311.075	27
8	-7	-5	3800.00	275.925	14
8	-7	-5	4004.82	310.744	11
8	7	-5	3656.36	306.997	19
8	-7	-4	23727.2	1198.12	14
8	7	-4	20949.3	1240.93	18
8	7	-4	22555.6	1230.46	19
8	-7	-4	24188.8	1229.99	11

-8	-7	3	404.508	98.2196	28
8	-7	-3	469.295	97.5126	14
8	7	-3	353.936	101.771	19
8	-7	-3	489.632	104.036	11
-8	-7	2	51.9437	55.9980	28
8	-7	-2	-11.1863	54.5281	14
8	7	-2	12.8617	58.4613	19
8	-7	-2	69.5721	63.4631	11
-8	-7	1	7801.11	506.902	22
8	7	-1	7480.85	472.414	19
8	7	-1	8196.29	513.545	18
8	-7	-1	6979.75	436.063	7
-8	-7	1	7421.40	483.793	28
8	-7	-1	7021.63	468.315	4
8	-7	-1	7958.73	473.652	14
-8	-7	0	5186.26	380.247	22
8	7	0	5198.79	382.748	18
8	7	0	5224.14	348.942	19
8	-7	0	4884.88	334.902	7
8	-7	0	4773.93	330.222	5
-8	-7	0	5109.20	369.908	28
8	-7	0	5286.98	365.093	4
8	-7	0	5538.42	361.611	14
-8	-7	-1	2424.18	231.502	22
8	7	1	1806.61	209.540	18
8	7	1	2242.22	190.380	19
8	-7	1	2265.21	198.140	7
8	-7	1	1802.25	182.946	5
-8	-7	-1	2126.56	214.499	28

8	-7	1	2087.68	208.440	4
8	-7	1	2639.52	216.474	14
8	-7	2	12743.9	752.587	4
8	7	2	13737.8	763.483	18
8	-7	2	13716.8	744.076	7
8	-7	2	13225.9	743.004	5
-8	-7	-2	13410.8	766.689	22
-8	-7	-2	13158.1	764.719	28
-8	-7	-3	403.383	103.750	22
8	-7	3	514.206	99.6393	7
-8	-7	-3	462.874	97.3473	28
8	-7	3	465.353	99.6502	4
8	-7	3	452.012	93.8017	5
8	7	3	350.953	90.8378	18
8	7	4	537.631	90.9743	18
8	-7	4	714.942	107.341	7
-8	-7	-4	447.979	101.113	22
8	-7	4	632.375	106.108	5
8	-7	5	-18.0366	51.8441	5
8	7	5	39.0189	41.8004	18
8	-7	6	53.4291	47.6017	7
8	-7	6	94.8032	55.5359	5
8	7	6	60.9630	28.1234	18
-8	-8	10	598.389	69.1022	22
8	-8	-10	719.935	77.5073	11
8	8	-10	796.905	82.5320	19
8	-8	-9	158.564	54.6707	11
8	8	-9	68.4201	50.4495	19
-8	-8	9	183.384	55.6285	22

8	-8	-8	1859.79	162.160	11
8	8	-8	1583.78	158.835	19
8	8	-8	1332.34	140.307	18
-8	-8	8	2084.44	176.256	22
-8	-8	7	1129.23	129.594	27
8	-8	-7	1300.70	140.907	11
-8	-8	7	1127.34	145.142	22
8	8	-7	1075.02	136.870	18
8	8	-7	1158.48	139.869	19
8	8	-6	101.232	68.6588	19
-8	-8	6	49.9821	70.7743	22
-8	-8	6	76.9142	57.0297	27
8	8	-6	205.723	79.7618	18
8	-8	-6	20.9838	62.2555	11
-8	-8	5	11935.0	698.173	27
8	8	-5	11983.4	712.965	18
8	8	-5	11117.3	683.361	19
-8	-8	5	14090.4	728.348	22
8	-8	-5	12067.9	681.956	11
-8	-8	4	146.716	74.6471	27
8	8	-4	193.501	75.3668	19
8	-8	-4	142.836	66.0362	11
-8	-8	4	204.668	90.1954	22
-8	-8	3	35.6667	56.6822	28
8	8	-3	43.5524	56.4330	19
8	-8	-3	18.8163	54.0988	11
-8	-8	3	27.5783	79.2748	22
-8	-8	2	7202.08	476.708	28
8	8	-2	8489.84	509.444	18

8	8	-2	6798.18	449.368	19
-8	-8	2	8244.24	502.478	22
8	8	-1	3334.18	276.380	18
8	8	-1	3091.51	234.244	19
-8	-8	1	2994.83	257.012	28
8	8	0	2789.03	203.847	19
8	8	0	2990.60	251.370	18
-8	-8	0	2401.23	228.149	28
8	-8	1	57.6084	52.2867	7
-8	-8	-1	12.0646	57.5577	28
8	8	1	128.247	78.6250	18
-8	-8	-2	3082.50	254.895	28
8	-8	2	3068.10	235.554	5
8	-8	2	3350.13	237.693	7
8	8	2	3269.93	248.248	18
-8	-8	-3	1173.40	137.888	28
8	8	3	1382.38	135.676	18
8	-8	3	1350.89	138.439	5
8	-8	3	1128.35	129.090	7
-8	-8	-4	43.6693	44.3854	28
8	-8	4	7.38668	45.0465	7
8	8	4	24.0745	36.7083	18
8	-8	4	7.98510	46.6219	5
8	-8	5	53.1240	46.3268	5
8	-8	6	532.401	65.0420	5
8	9	-9	390.455	44.0667	19
8	9	-8	61.3665	38.6452	19
8	9	-7	5390.87	333.571	18
8	9	-7	5209.73	320.058	19

-8	-9	7	5656.31	332.617	22
-8	-9	6	3389.81	241.675	22
-8	-9	6	2946.03	231.378	27
8	9	-6	3532.37	248.563	18
8	9	-6	3232.05	224.664	19
8	9	-5	846.293	126.474	18
-8	-9	5	957.299	122.984	27
8	9	-5	975.905	109.139	19
-8	-9	5	1029.72	124.892	22
8	9	-4	73.6806	46.3246	19
-8	-9	4	133.762	73.5131	27
-8	-9	4	71.7685	58.1422	22
-8	-9	3	2112.23	192.003	22
8	9	-3	1811.14	154.140	19
8	9	-3	1799.73	184.722	18
-8	-9	3	2057.23	196.436	27
-8	-9	2	3336.07	251.296	22
8	9	-2	3301.36	258.035	18
-8	-9	2	2655.06	232.212	28
-8	-9	1	2081.50	195.427	22
-8	-9	1	2343.79	200.170	28
8	9	-1	2375.95	207.199	18
-8	-9	0	272.978	72.8146	28
-8	-9	0	428.895	91.9440	22
8	9	0	315.333	86.3414	18
-8	-9	-1	373.842	79.5638	28
-8	-9	-1	437.476	86.6596	22
8	9	1	394.253	82.0026	18
-8	-9	-2	-17.2589	43.8469	28

-8	-9	-2	-25.1746	56.5638	22
8	9	2	6.92251	41.0197	18
8	9	3	1638.45	126.726	18
8	10	-7	1282.18	96.6215	18
8	10	-6	1083.74	103.401	18
8	10	-4	229.865	62.0715	18
-8	-10	4	166.605	57.8269	27
-8	-10	4	225.981	55.2475	22
-8	-10	3	1057.32	110.154	22
8	10	-3	1124.49	122.836	18
-8	-10	3	1161.36	123.504	27
-8	-10	2	4237.48	269.559	22
-8	-10	2	4213.43	286.900	27
8	10	-2	4255.05	280.794	18
8	10	-1	322.660	68.0198	18
-8	-10	1	378.537	76.6482	27
-8	-10	1	360.302	71.0940	22
-8	-10	0	1212.55	103.501	22
8	10	0	1255.73	103.452	18
-8	-10	-1	440.894	69.3643	22
8	10	1	405.119	60.3683	18
8	11	-4	151.236	30.3624	18
8	11	-3	38.2183	24.7783	18
8	11	-2	759.661	65.9005	18
-9	0	13	351.357	51.3110	22
9	0	-13	510.377	71.7832	12
-9	0	12	8.29048	43.7753	22
-9	0	12	-30.8822	58.2408	29
-9	0	11	465.734	102.070	22

-9	0	10	-13.7159	73.7510	22
9	0	-9	3242.44	253.626	11
-9	0	9	2903.41	271.063	22
9	0	-8	11.7578	59.7511	19
9	0	-8	73.9699	130.193	1
9	0	-8	-73.5276	167.070	17
9	0	-8	-12.8823	61.4118	11
-9	0	8	-86.3265	73.1106	22
9	0	-7	-25.5957	58.1632	19
9	0	-7	-76.3519	134.381	1
9	0	-7	72.7556	147.861	17
9	0	-7	-13.6720	60.5623	11
-9	0	7	-13.8931	59.9002	22
9	0	-6	-26.6178	60.4975	19
9	0	-6	-74.7706	169.895	1
9	0	-6	-71.9786	146.287	17
9	0	-6	-55.9062	72.4314	11
9	0	-6	31.6551	66.3279	12
9	0	-5	16315.2	922.275	12
9	0	-5	16406.0	906.242	11
9	0	-5	16975.4	1389.65	1
9	0	-5	16041.6	899.869	19
9	0	-4	66.8469	151.886	1
9	0	-4	-54.2543	61.6394	19
9	0	-4	-13.9491	63.3914	11
9	0	-4	29.1261	67.8153	12
9	0	-3	2961.71	258.961	12
9	0	-3	2618.97	250.075	14
9	0	-3	2405.35	431.911	25

9	0	-3	2929.72	260.657	26
9	0	-3	2792.66	247.858	19
9	0	-3	2746.87	474.312	1
9	0	-2	13.5599	55.1176	19
9	0	-2	-14.4406	68.8274	26
9	0	-2	59.2046	120.321	25
9	0	-2	92.3677	72.2079	14
9	0	-2	27.3265	66.5863	12
9	0	-2	23.0265	56.1094	18
9	0	-2	-54.7545	124.411	1
9	0	-1	4412.08	326.548	14
9	0	-1	4416.14	335.835	13
9	0	-1	4852.92	360.326	26
9	0	-1	4297.87	592.194	25
9	0	-1	4176.66	323.034	18
9	0	-1	4582.36	340.068	19
9	0	-1	3846.86	511.562	1
9	0	0	54.2621	68.9237	19
9	0	0	26.4808	58.6467	18
9	0	0	15.5737	70.7731	26
-9	0	0	-169.976	228.485	23
9	0	0	26.4888	67.2921	13
9	0	0	36.7748	63.5156	14
9	0	1	18568.9	1009.22	13
9	0	1	17336.0	992.165	14
9	0	1	18345.2	1016.46	18
9	0	1	19057.3	1022.20	19
9	0	2	14.0748	74.3224	18
-9	0	-2	68.3705	120.336	23

9	0	2	77.1687	69.1628	13
9	0	2	-11.6084	55.3373	14
9	0	3	1386.60	158.544	14
-9	0	-3	1639.86	377.341	23
9	0	3	1457.53	172.850	6
9	0	3	1497.10	179.885	18
9	0	4	-47.6108	66.2676	6
9	0	4	-24.0722	67.0098	7
9	0	5	187.294	70.6394	7
9	0	5	171.291	71.0028	6
9	0	6	8.11668	48.7939	7
9	0	6	-21.1308	67.0455	18
9	0	7	3046.02	198.430	7
9	0	8	-28.4973	38.8823	5
9	0	8	-38.4023	39.0542	26
-9	0	-9	3.22997	21.7713	20
-9	-1	13	2244.31	165.418	22
9	-1	-13	2540.17	181.131	12
9	1	-13	2504.32	169.248	12
-9	-1	12	473.452	91.4364	29
-9	-1	12	658.056	97.7249	22
9	1	-12	472.610	94.4212	12
-9	1	11	75.1776	53.2576	22
-9	-1	11	62.6949	65.0433	22
9	-1	-11	62.3434	44.9039	11
-9	-1	10	2758.73	262.934	22
9	-1	-10	2952.20	238.108	11
9	1	-9	2274.04	215.356	19
-9	-1	9	2097.07	235.283	22

9	-1	-9	2656.62	232.681	11
9	1	-8	2617.99	244.482	19
9	-1	-8	2856.78	254.949	11
-9	-1	8	2630.08	260.492	22
-9	-1	7	3182.23	278.885	22
9	1	-7	3216.94	272.826	19
9	1	-7	2959.82	279.686	12
9	1	-7	2818.00	243.695	11
9	1	-7	2487.23	508.988	17
-9	1	7	3298.52	505.145	30
9	-1	-7	2671.74	262.304	11
9	-1	-6	8056.46	522.576	11
-9	-1	6	7869.82	517.827	22
9	-1	-6	7981.27	490.327	19
9	1	-6	8073.88	519.957	19
9	1	-6	8322.42	528.746	12
9	-1	-6	7813.82	533.269	12
9	1	-6	7872.55	502.828	11
9	1	-6	6718.28	846.724	17
-9	-1	6	8490.71	908.079	30
-9	1	6	6971.14	841.248	30
9	-1	-6	6965.47	864.523	1
-9	-1	5	751.788	122.436	22
9	-1	-5	697.020	113.130	19
9	1	-5	722.624	126.599	19
9	1	-5	348.363	247.239	17
9	-1	-5	418.647	226.384	1
9	1	-5	598.546	114.973	11
9	-1	-5	578.066	126.306	11

9	1	-5	650.217	126.774	12
9	-1	-5	872.377	146.726	12
-9	-1	4	10294.7	671.962	22
9	1	-4	12047.7	696.994	12
9	-1	-4	11448.1	696.154	11
9	-1	-4	11285.3	699.172	12
9	-1	-4	12254.3	710.880	14
9	-1	-4	12122.1	1104.09	1
9	1	-4	12164.3	1115.09	1
9	-1	-4	11672.3	677.099	19
9	1	-4	12871.3	712.582	19
9	1	-3	2687.74	450.819	25
9	-1	-3	2209.49	399.878	25
9	1	-3	2520.31	233.011	26
9	-1	-3	2003.67	215.378	11
9	-1	-3	2397.13	232.487	12
9	-1	-3	1980.58	215.859	14
9	1	-3	2158.83	209.527	14
9	1	-3	2182.95	218.419	12
9	-1	-3	2207.24	416.886	1
9	1	-3	2682.27	450.139	1
9	1	-3	2057.75	203.083	18
9	-1	-3	2362.06	213.709	19
9	1	-3	2128.27	224.936	19
9	-1	-2	3622.81	536.706	25
9	1	-2	4793.27	346.478	26
9	-1	-2	4255.94	337.440	26
9	1	-2	4556.52	590.427	25
9	1	-2	3697.17	305.715	14

9	-1	-2	4428.82	333.723	14
9	1	-2	4609.53	335.418	12
9	-1	-2	4362.01	336.725	12
9	1	-2	3665.25	521.784	1
9	-1	-2	4738.89	589.550	1
9	1	-2	4462.02	326.371	18
9	-1	-2	4337.36	322.848	19
9	1	-2	4443.14	337.367	19
9	1	-1	2640.02	463.371	25
9	-1	-1	2481.56	440.452	25
9	1	-1	2741.37	258.324	26
9	-1	-1	2932.93	261.254	26
9	-1	-1	3090.95	259.114	13
9	-1	-1	2712.79	245.802	14
9	1	-1	2654.28	229.211	14
9	1	-1	2450.95	234.022	13
9	-1	-1	2285.37	406.159	1
9	-1	-1	2648.26	229.712	18
9	1	-1	2664.39	248.269	19
9	-1	-1	2801.27	243.054	19
9	1	-1	2726.30	244.918	18
9	1	0	6774.99	468.007	26
9	-1	0	6756.37	468.364	26
9	1	0	6268.60	750.032	25
9	-1	0	6481.60	759.182	25
9	-1	0	7034.26	453.794	19
9	1	0	6933.21	458.734	19
9	-1	0	6644.75	439.409	18
9	1	0	6747.33	459.482	18

9	-1	0	5979.14	658.720	1
-9	1	0	8450.78	1004.51	23
9	-1	0	7167.41	462.081	13
9	1	0	6849.68	447.784	13
9	1	0	6717.10	431.235	14
9	-1	0	6615.16	448.406	14
9	-1	1	26.1272	62.2898	18
9	1	1	-14.4015	74.6413	18
9	1	1	16.2076	75.4937	26
9	-1	1	127.109	79.1152	26
9	1	1	-66.7669	117.524	25
9	-1	1	130.986	133.111	25
9	-1	1	93.8565	84.0067	13
9	1	1	49.8337	64.5772	13
9	1	1	32.4601	57.1598	14
9	-1	1	63.8113	71.0537	14
-9	1	-1	-74.7305	200.916	23
9	-1	2	5445.49	407.718	26
9	1	2	5574.34	409.316	26
9	-1	2	5143.54	371.673	18
9	1	2	4925.07	380.099	18
9	-1	2	6461.26	752.172	25
9	1	2	4109.00	632.476	25
9	-1	2	5334.07	376.666	6
9	-1	2	5032.08	362.278	14
9	-1	2	5297.44	375.586	13
9	1	2	5443.33	372.822	13
9	1	2	4935.90	344.126	14
-9	1	-2	4521.71	656.506	23

9	1	3	31206.5	1642.22	26
9	-1	3	31625.3	1641.14	26
9	-1	3	32244.9	1610.31	18
9	1	3	31414.3	2141.56	25
9	-1	3	27482.9	2055.68	25
9	-1	3	30711.2	1598.89	6
9	1	3	30741.6	1588.15	6
-9	1	-3	32124.7	2088.37	23
9	1	3	30139.6	1563.26	14
9	-1	3	30872.5	1587.97	14
9	1	4	4771.92	390.868	26
9	-1	4	4906.01	387.769	26
9	-1	4	5212.82	363.222	6
9	1	4	5038.17	350.316	6
9	-1	4	5250.08	363.237	7
9	-1	4	5269.36	368.624	18
9	1	5	1843.86	213.415	26
9	-1	5	1943.35	214.615	26
9	-1	5	2105.13	194.487	6
9	1	5	1788.82	175.991	6
9	-1	5	1822.62	183.020	7
9	1	5	1931.05	180.365	7
9	1	5	1895.05	194.337	18
9	1	6	12194.5	714.600	26
9	-1	6	12309.5	710.818	26
9	-1	6	12178.6	666.537	7
9	1	6	11832.1	652.102	7
9	1	6	12711.8	688.975	18
9	-1	7	83.0330	43.4109	7

9	-1	7	183.800	63.2135	5
9	1	7	80.7515	55.9403	18
9	1	7	118.047	61.9463	26
9	-1	7	194.400	64.3396	26
9	-1	8	74.4601	39.9951	5
9	1	8	75.0195	38.9820	5
9	1	8	37.8876	37.8210	26
9	-1	8	49.1674	39.1887	26
-9	1	-9	796.049	62.8984	20
-9	-1	-9	861.406	70.3912	20
9	-2	-13	2579.89	169.552	12
-9	-2	13	1919.63	153.010	22
-9	2	12	1519.77	145.648	29
9	2	-12	1674.86	129.958	12
9	-2	-12	1250.84	105.650	11
9	-2	-12	1291.35	143.882	12
-9	-2	12	1799.28	155.484	22
-9	-2	11	2898.92	244.249	22
9	-2	-11	3000.44	218.897	11
9	2	-11	2220.95	210.750	12
9	2	-10	180.746	70.3044	19
-9	-2	10	267.042	95.6954	22
9	-2	-10	203.971	81.2873	11
9	2	-10	84.2796	75.8622	12
9	2	-9	4060.32	323.320	19
-9	-2	9	4197.36	345.048	22
9	2	-9	3477.66	321.855	12
9	2	-8	-14.9458	69.5987	19
9	2	-8	14.0716	67.0704	12

-9	-2	8	-16.4865	67.0138	22
9	-2	-8	-45.7768	71.0568	11
9	-2	-7	1689.13	194.938	11
-9	-2	7	1582.95	198.844	22
9	2	-7	1628.62	194.185	19
9	2	-7	1526.76	182.290	12
9	2	-7	751.939	286.990	17
9	-2	-7	1599.83	383.576	17
-9	-2	7	1187.38	315.893	30
-9	2	7	1896.62	378.149	30
9	-2	-7	1718.07	401.512	1
-9	-2	6	6294.92	453.933	22
9	2	-6	7415.42	472.339	19
9	2	-6	7004.13	450.265	12
9	-2	-6	6588.31	459.075	11
9	-2	-6	7645.59	846.036	17
-9	-2	5	3446.86	301.250	22
-9	2	5	2658.92	539.805	30
9	2	-5	3687.22	309.402	19
9	-2	-5	2337.51	494.985	17
9	-2	-5	4309.87	624.373	1
9	-2	-5	3575.03	303.271	11
9	2	-5	3789.23	296.767	12
-9	-2	4	422.336	106.545	22
9	2	-4	493.874	118.023	19
9	2	-4	519.120	102.459	18
9	-2	-4	462.246	223.449	1
9	-2	-4	470.671	119.196	11
9	2	-4	404.567	97.7090	12

9	-2	-4	371.275	106.183	14
9	2	-4	339.252	94.2291	14
9	2	-3	1764.87	378.780	1
9	-2	-3	2649.44	459.302	1
9	-2	-3	2305.10	226.256	11
9	-2	-3	2198.59	228.823	14
9	2	-3	1816.41	191.779	14
9	2	-3	2115.83	205.625	12
9	2	-3	1887.48	204.083	18
9	-2	-3	2180.09	195.587	19
9	2	-3	2320.90	229.940	19
-9	-2	3	1961.06	211.148	22
9	2	-3	1515.40	342.001	25
9	-2	-3	2318.28	400.787	25
9	2	-3	2061.93	211.541	26
9	-2	-3	2337.72	217.436	26
9	2	-2	2850.88	466.853	1
9	-2	-2	3078.01	489.398	1
9	2	-2	3254.73	259.616	14
9	-2	-2	3501.87	291.960	14
9	-2	-2	3460.31	288.118	13
9	-2	-2	3732.15	296.106	11
9	2	-2	3674.89	288.568	18
9	-2	-2	2950.33	256.459	19
9	2	-2	3234.03	290.745	19
-9	-2	2	3278.51	275.148	22
9	2	-2	3438.05	513.369	25
9	-2	-2	2839.39	470.732	25
9	2	-2	3707.03	295.306	26

9	-2	-2	3604.10	289.834	26
9	-2	-1	2794.47	454.408	1
9	2	-1	2651.57	239.694	13
9	-2	-1	3131.28	261.094	14
9	2	-1	2798.85	252.969	18
-9	-2	1	2654.42	238.875	22
9	2	-1	2297.07	426.859	25
9	-2	-1	2761.97	452.967	25
9	2	-1	2459.01	247.380	26
9	-2	-1	2685.99	248.444	26
9	-2	0	27436.9	1733.12	1
9	2	0	26041.8	1377.49	18
9	-2	0	25225.3	1338.21	18
9	2	0	25833.2	1371.26	19
9	2	0	26097.9	1383.70	26
9	-2	0	26958.5	1383.11	26
9	2	0	25381.7	1802.35	25
9	-2	0	24457.0	1763.96	25
9	2	0	25507.1	1347.94	13
9	-2	0	25693.2	1364.99	14
-9	-2	0	24990.9	1347.60	22
9	2	1	50261.3	2505.77	26
9	-2	1	48182.9	2461.96	18
9	2	1	50564.0	2486.53	19
9	2	1	48738.2	2491.70	18
9	-2	1	50235.2	2497.87	26
9	2	1	49719.8	2989.02	25
9	-2	1	49725.2	2964.56	25
9	-2	1	49444.1	2476.70	6

9	-2	1	51011.5	2482.22	14
9	2	1	48365.5	2452.98	13
-9	-2	-1	46673.9	2454.63	22
-9	-2	-1	58833.0	3155.19	23
-9	2	-1	59214.7	3186.77	23
-9	-2	-2	22373.8	1550.07	23
-9	2	-2	21832.7	1533.01	23
-9	-2	-2	18392.1	1007.13	22
9	2	2	18979.0	1033.20	19
9	-2	2	18705.7	1019.54	18
9	2	2	19079.2	1044.29	18
9	2	2	18633.1	1476.98	25
9	-2	2	18483.4	1456.37	25
9	-2	2	19802.3	1061.71	26
9	2	2	18829.3	1054.89	26
9	-2	2	18475.6	1023.16	14
9	2	2	17422.5	1002.62	13
9	-2	3	14417.9	807.101	18
9	-2	3	14294.1	802.396	14
9	-2	3	14700.5	810.301	6
9	-2	3	14623.6	811.520	7
9	2	3	13021.7	777.442	6
9	-2	3	14253.3	830.443	26
9	2	3	14863.6	845.964	26
-9	-2	-3	13718.1	781.789	22
9	-2	4	14892.2	839.489	18
9	2	4	15367.5	853.220	18
9	-2	4	16053.4	851.100	6
9	2	4	14032.9	810.350	6

9	-2	4	14984.3	840.772	7
9	-2	4	15274.8	870.935	26
9	2	4	15199.5	877.619	26
9	2	5	118.415	76.2050	18
9	-2	5	13.8049	70.2518	26
9	2	5	103.837	82.6558	26
9	2	5	122.964	60.6633	7
9	-2	5	44.6273	71.8116	6
9	2	5	112.904	59.9879	6
9	-2	5	85.7325	71.5186	7
9	-2	6	4399.01	311.701	26
9	2	6	4066.73	311.894	26
9	-2	6	4393.51	301.455	5
9	-2	6	4110.84	280.647	7
9	2	6	3706.00	252.648	7
9	2	6	4546.12	299.159	18
9	-2	7	457.837	82.7445	26
9	2	7	335.119	79.2561	26
9	-2	7	425.307	81.3222	5
9	-2	7	436.552	68.5288	7
9	2	7	369.647	74.5330	18
9	-2	8	5.82089	20.9134	7
-9	2	-8	4.23436	28.8642	20
9	-2	8	5.45313	36.3362	5
-9	-2	-9	9.99858	16.0710	20
-9	-3	13	240.075	47.2636	22
9	-3	-13	283.790	47.1809	12
-9	-3	12	1939.97	164.568	22
9	-3	-12	1939.14	159.571	12

9	-3	-12	2023.38	139.714	11
-9	-3	11	416.263	95.5938	22
9	-3	-11	233.620	92.3865	12
9	3	-11	286.585	60.3527	12
9	3	-11	366.838	80.8124	19
9	-3	-11	354.502	81.8484	11
-9	3	11	309.652	86.9103	29
9	3	-10	1005.63	135.330	19
9	-3	-10	903.722	136.027	11
-9	-3	10	928.820	145.892	22
9	3	-10	878.709	114.484	12
9	3	-9	1121.24	155.680	19
9	-3	-9	975.049	149.559	11
-9	-3	9	986.700	158.672	22
9	3	-9	1297.15	146.065	12
9	3	-8	854.997	143.494	19
9	-3	-8	877.216	144.534	11
-9	-3	8	818.102	150.714	22
9	-3	-8	651.680	140.174	12
9	3	-8	546.438	105.743	12
9	3	-7	31770.4	1640.80	19
9	3	-7	30530.9	1595.90	12
9	-3	-7	31216.3	1639.77	12
9	-3	-7	30617.5	1633.64	11
-9	-3	7	31560.1	1649.27	22
-9	-3	6	3744.85	311.871	22
9	3	-6	3687.01	306.764	19
9	3	-6	3693.96	276.938	12
9	-3	-6	3553.97	302.885	12

9	-3	-6	2742.05	503.228	17
9	-3	-6	3489.71	300.518	11
-9	-3	5	69569.8	2508.87	22
9	3	-5	66854.9	2453.60	18
9	3	-5	67083.6	2507.97	19
9	-3	-5	70845.1	3226.17	1
9	-3	-5	69613.1	2510.08	11
9	3	-5	69849.5	2449.80	12
9	-3	-5	71710.2	2509.63	12
-9	-3	4	15.6090	67.2922	22
9	3	-4	13.6467	69.3346	18
9	-3	-4	201.824	193.351	1
9	-3	-4	-46.7002	88.0705	11
9	-3	-4	15.0187	76.3052	12
9	3	-4	-35.3175	67.6702	12
9	-3	-3	6681.56	793.521	1
9	3	-3	6568.05	459.543	18
-9	-3	3	6679.21	465.006	22
9	-3	-3	7067.94	468.378	13
9	3	-3	6825.14	439.706	12
9	-3	-3	7071.76	471.156	12
9	3	-3	7522.01	757.746	25
9	3	-3	7161.11	459.263	26
-9	-3	2	-28.9370	70.5079	22
9	3	-2	15.2580	84.9195	19
9	3	-2	29.4810	66.9851	18
9	-3	-2	61.9369	154.161	1
9	-3	-2	55.2024	70.1162	13
9	-3	-2	42.3791	70.3213	12

9	-3	-2	29.5602	68.8236	14
9	3	-2	-13.6301	73.2871	26
9	-3	-2	-26.1246	66.3651	26
9	3	-2	-56.0133	150.588	25
9	-3	-2	-53.6341	144.191	25
-9	-3	1	3892.17	317.866	22
9	-3	-1	4230.26	579.418	1
9	3	-1	4282.98	337.676	18
9	3	-1	3961.27	326.544	19
9	-3	-1	4309.96	323.380	13
9	-3	-1	4313.37	334.497	14
9	3	-1	3963.67	303.390	13
9	-3	-1	4277.81	558.219	25
9	3	-1	3972.81	322.486	26
9	-3	-1	4348.42	323.029	26
9	3	-1	3837.30	550.275	25
9	3	0	60.1780	136.733	25
9	3	0	29.2430	71.2535	26
9	-3	0	-13.8389	67.4400	26
9	-3	0	114.344	116.188	25
-9	-3	0	-71.0628	161.464	23
-9	3	0	74.9659	170.333	23
-9	-3	0	-13.5125	54.9220	22
9	3	0	28.7076	72.9268	19
9	-3	0	-10.0887	48.0839	18
9	-3	0	53.8119	133.938	1
9	-3	0	90.4938	70.7409	6
9	3	0	-45.4373	100.919	18
9	-3	0	13.5635	72.9290	13

9	3	0	-21.9665	55.8024	13
9	-3	0	-14.0484	68.4607	14
-9	-3	-1	-64.4458	173.356	23
-9	3	-1	129.724	161.548	23
-9	-3	-1	209.689	85.4702	22
9	3	1	41.4800	83.3241	19
9	-3	1	134.258	64.5757	18
9	-3	1	243.503	94.4200	13
9	3	1	104.453	59.3832	13
9	-3	1	206.029	89.5578	14
9	-3	1	108.734	87.5437	7
9	-3	1	262.476	88.6504	6
9	3	1	105.793	88.4126	18
9	-3	1	58.5961	133.266	25
9	3	1	225.662	91.9067	26
9	-3	1	169.954	80.3398	26
9	3	1	62.1350	126.409	25
-9	-3	-2	9172.78	911.384	23
-9	3	-2	8631.02	879.449	23
-9	-3	-2	9029.96	561.578	22
9	-3	2	8898.06	554.448	18
9	3	2	9217.66	570.280	19
9	-3	2	9712.98	577.276	6
9	3	2	9006.94	584.337	26
9	-3	2	9689.65	577.722	14
9	-3	2	9238.49	580.673	26
-9	-3	-3	3165.17	264.187	22
9	-3	3	3483.06	286.740	26
9	3	3	3193.69	282.789	18

9	-3	3	3525.43	281.920	14
9	-3	3	3619.38	284.040	7
9	3	3	3061.19	249.227	6
9	3	3	3361.23	291.746	26
9	3	4	7488.15	493.086	26
9	-3	4	7327.91	480.102	26
9	3	4	7739.33	482.105	18
9	3	4	6881.30	433.948	6
9	-3	4	7753.27	473.964	7
9	3	5	782.253	92.8072	6
9	3	5	792.995	98.6552	7
9	-3	5	734.949	118.119	7
9	-3	5	755.604	118.476	26
9	3	5	869.111	132.788	26
9	3	5	742.426	117.320	18
9	-3	6	5245.07	332.114	7
9	-3	6	4826.66	334.148	26
9	3	6	4748.64	345.224	26
9	3	6	5900.52	343.883	18
9	3	7	2081.48	175.055	26
9	3	7	2137.38	161.453	18
9	-3	7	2308.73	174.341	5
9	-3	7	1986.15	156.428	7
9	-3	8	625.329	59.1924	7
9	-3	8	877.115	82.0666	5
-9	-4	13	710.776	64.0378	22
9	-4	-12	3316.95	209.025	12
9	-4	-12	3025.71	202.828	11
-9	-4	12	3070.94	220.070	22

9	4	-11	662.118	94.8253	19
-9	-4	11	540.280	103.476	22
-9	4	11	297.167	72.9486	29
9	-4	-11	523.275	90.6965	11
9	-4	-11	551.015	90.8717	12
9	-4	-11	462.513	79.2300	14
9	-4	-10	73.2720	64.9598	14
9	-4	-10	125.691	75.4717	12
-9	-4	10	184.202	80.4285	22
9	-4	-10	143.113	70.9018	11
9	4	-10	116.919	71.5210	19
9	-4	-9	119.040	81.0617	12
-9	-4	9	165.725	88.3302	22
9	-4	-9	256.399	95.2720	11
9	4	-9	183.058	86.2474	19
9	-4	-8	999.322	149.747	12
-9	-4	8	942.990	151.956	22
9	4	-8	861.668	145.307	19
9	-4	-8	815.956	144.536	11
9	-4	-7	7018.80	460.250	12
9	-4	-7	6649.03	456.824	14
9	-4	-7	6647.05	463.811	11
-9	-4	7	6872.56	474.602	22
9	4	-7	7038.51	471.042	19
9	4	-7	5788.95	405.872	18
-9	-4	6	252.545	107.353	22
9	4	-6	214.072	84.5597	18
9	-4	-6	327.260	101.939	12
9	-4	-6	229.836	92.7063	14

9	-4	-6	128.375	88.4334	11
-9	-4	5	49.9099	86.3025	22
9	-4	-5	58.7600	71.7121	12
9	-4	-5	193.056	82.8208	13
9	-4	-5	46.1401	78.2551	14
9	4	-5	110.430	78.2103	18
-9	4	4	121.882	138.466	30
-9	-4	4	16.2515	73.8516	22
9	4	-4	16.0602	69.2369	19
9	-4	-4	-202.036	273.726	1
9	4	-4	-14.5565	72.4624	18
9	-4	-4	-14.3477	71.4230	12
9	-4	-4	26.0858	76.1345	13
9	-4	-4	30.4411	77.3304	14
9	4	-3	27458.0	1510.16	18
9	4	-3	28196.1	1522.01	19
9	-4	-3	30050.4	1522.20	11
9	-4	-3	29167.2	1499.15	13
9	-4	-3	30018.5	1519.99	14
9	-4	-3	29893.6	1509.07	12
9	-4	-3	30053.2	1998.94	1
9	-4	-3	27283.8	1455.33	7
9	-4	-3	28920.1	1465.32	6
-9	-4	3	27756.4	1515.01	22
-9	-4	2	2079.05	234.974	22
9	4	-2	2547.43	247.246	18
9	4	-2	2817.64	259.528	19
9	-4	-2	2516.64	231.387	13
9	-4	-2	2429.53	243.106	14

9	4	-2	2318.96	210.513	13
9	-4	-2	2427.21	239.952	11
9	-4	-2	3293.25	507.272	1
9	-4	-2	2289.70	210.443	7
9	-4	-2	2667.54	221.941	6
9	4	-2	2059.60	379.978	25
9	4	-2	2269.85	221.070	26
-9	-4	1	16815.5	1632.47	23
-9	-4	1	25019.5	1323.86	22
9	4	-1	22081.8	1264.37	13
9	-4	-1	27406.5	1318.70	13
9	4	-1	24123.1	1327.23	18
9	4	-1	24366.1	1321.58	19
9	-4	-1	27737.8	1780.25	1
9	-4	-1	25583.7	1300.31	7
9	-4	-1	27477.9	1306.47	6
9	4	-1	20451.9	1606.67	25
9	-4	-1	22229.8	1610.49	25
9	4	-1	25337.8	1308.27	26
9	-4	-1	25987.3	1300.33	26
9	-4	0	182.865	89.2661	13
9	4	0	110.663	137.870	25
9	4	0	120.286	74.6615	26
9	-4	0	183.444	72.7447	26
9	-4	0	152.645	136.943	25
-9	-4	0	124.700	155.322	23
-9	-4	0	227.264	86.8368	22
9	4	0	42.0189	68.5572	19
9	-4	0	114.713	130.480	1

9	-4	0	127.475	79.0525	7
9	-4	0	85.7268	68.4621	6
9	4	0	138.485	95.3231	18
9	-4	0	183.140	66.5104	5
9	-4	1	33090.0	1790.54	5
9	4	1	35065.7	1817.71	19
9	-4	1	37759.4	1819.60	6
9	-4	1	36687.5	1820.75	7
9	4	1	36791.4	1827.89	26
9	-4	1	35844.6	1810.63	26
-9	-4	-1	34474.6	1820.32	22
-9	-4	-1	36155.4	2273.68	23
9	-4	2	35.5536	65.9588	5
-9	-4	-2	-13.0828	62.3537	22
9	4	2	12.4520	61.9858	19
9	4	2	-28.8575	91.5610	18
9	-4	2	25.0565	69.7268	6
9	-4	2	-38.6185	75.1416	7
9	4	2	-27.3265	66.5835	26
9	-4	2	36.4421	56.5642	26
9	-4	2	-13.2380	69.8961	14
9	-4	3	15452.4	811.155	5
9	4	3	13919.6	813.710	18
-9	-4	-3	14548.8	805.498	22
9	-4	3	14038.0	803.863	6
9	4	3	13041.0	759.358	6
9	4	3	14919.6	818.520	26
9	-4	3	13886.7	794.108	26
9	-4	3	15883.3	822.681	14

-9	-4	-4	2392.94	215.631	22
9	-4	4	2619.43	223.465	5
9	4	4	2285.17	219.542	18
9	-4	4	2361.28	207.537	26
9	-4	4	2240.31	213.613	7
9	4	4	2904.52	237.612	26
9	4	5	107.800	62.0685	26
9	-4	5	164.402	72.9650	7
9	-4	5	20.5453	61.8585	5
9	4	5	49.6629	60.6631	18
9	4	6	461.833	78.7361	18
9	-4	6	418.519	81.2492	7
9	4	6	395.194	77.7092	26
9	4	7	1457.05	115.147	18
9	-4	7	1487.96	122.206	7
9	-4	7	1323.12	124.736	5
9	-4	8	651.661	66.0848	5
9	5	-12	301.914	44.0329	19
9	-5	-12	247.451	44.2174	11
-9	-5	12	301.413	53.5476	22
9	5	-11	110.820	50.6739	19
-9	-5	11	113.039	60.1904	22
9	-5	-11	137.157	56.6493	11
-9	-5	10	490.545	99.7219	22
9	5	-10	501.558	93.8918	19
9	-5	-10	411.815	80.0444	12
9	-5	-10	463.948	95.3376	11
9	-5	-9	12.8780	68.0041	11
9	5	-9	12.7656	64.8669	19

-9	-5	9	-40.6240	80.2398	22
9	-5	-9	-41.7052	57.0634	12
9	-5	-9	-29.4397	53.7094	14
-9	-5	8	-15.0833	73.5744	22
9	-5	-8	82.6958	67.9829	12
9	-5	-8	104.999	68.1769	14
9	-5	-8	99.5752	83.0974	11
9	5	-7	-11.0446	58.3172	18
9	-5	-7	-12.6518	68.0284	12
9	-5	-7	-25.8305	66.9191	14
-9	-5	7	32.0032	70.8761	22
9	5	-6	3591.87	306.395	19
9	5	-6	2983.33	274.519	18
9	-5	-6	3320.28	277.316	12
9	-5	-6	4065.58	302.226	14
9	-5	-6	3324.48	253.875	13
-9	-5	6	3633.09	312.813	22
-9	-5	5	23064.5	1263.26	22
9	-5	-5	24057.5	1206.75	13
9	-5	-5	24097.7	1246.85	14
9	-5	-5	24169.9	1234.65	12
9	-5	-5	22797.5	1250.29	11
9	5	-5	21486.8	1233.16	18
9	5	-5	21828.9	1247.75	19
9	-5	-4	1082.53	157.800	11
9	5	-4	1293.31	169.654	19
-9	-5	4	1187.32	173.997	22
9	-5	-4	1083.46	139.619	13
9	-5	-4	1334.81	170.862	14

9	-5	-4	1061.15	149.421	12
9	5	-4	1148.59	168.026	18
9	-5	-3	334.648	104.325	11
-9	-5	3	589.660	130.886	22
9	5	-3	502.024	120.290	19
9	-5	-3	389.841	199.599	1
9	-5	-3	571.184	106.089	13
9	-5	-3	678.851	125.245	14
9	5	-3	597.135	120.030	18
9	-5	-2	1866.33	169.461	6
9	-5	-2	1640.10	166.442	7
9	-5	-2	2104.30	418.182	1
-9	-5	2	1696.95	202.112	22
9	-5	-2	1967.34	206.199	14
9	-5	-2	1836.54	199.795	11
9	-5	-2	1797.32	181.929	13
9	5	-2	1593.11	203.159	18
9	5	-2	1726.40	200.228	19
9	5	-1	12795.9	786.347	18
9	5	-1	13819.8	780.968	19
-9	-5	1	13210.5	782.344	22
9	-5	-1	13229.2	741.391	6
9	-5	-1	12740.8	740.715	7
9	5	-1	13409.9	745.733	26
9	-5	-1	14800.1	790.476	14
9	-5	-1	13420.3	759.086	13
9	-5	0	389.404	102.638	14
-9	-5	0	604.653	118.167	22
9	5	0	459.432	106.461	19

9	-5	0	473.952	100.698	6
9	-5	0	568.798	109.042	7
9	5	0	505.122	96.5816	26
9	-5	0	368.855	81.6672	5
9	-5	1	691.012	124.058	14
-9	-5	-1	783.563	133.335	22
9	5	1	778.285	117.576	19
9	-5	1	782.443	118.922	6
9	-5	1	763.663	121.119	7
9	5	1	508.823	99.8734	26
9	-5	1	616.583	105.044	5
9	5	1	564.913	122.798	18
9	-5	2	276.396	89.2150	14
-9	-5	-2	233.188	89.0758	22
9	5	2	149.216	83.5252	18
9	-5	2	213.438	79.9428	6
9	-5	2	329.419	91.7917	7
9	-5	2	262.160	80.8886	5
9	5	2	288.269	79.7240	26
9	5	3	2996.80	232.254	26
9	5	3	2632.65	238.571	18
9	-5	3	2784.33	237.166	7
9	-5	3	2829.41	232.764	5
-9	-5	-3	2643.45	236.290	22
9	5	4	272.903	84.7633	18
9	5	4	204.004	64.2440	26
9	-5	4	287.250	82.8824	5
9	5	5	6375.84	389.057	18
9	-5	5	6367.50	394.487	7

9	-5	5	6647.38	400.841	5
9	5	6	145.714	47.3644	18
9	-5	6	230.203	62.4707	7
9	-5	7	712.771	78.6725	5
9	-5	7	678.789	72.5249	7
9	-6	-11	227.089	51.8444	11
9	6	-11	249.478	53.1303	19
9	-6	-10	753.371	103.968	11
-9	-6	10	759.148	107.489	22
9	-6	-9	32.2822	59.8909	11
-9	-6	9	-11.3290	65.1213	22
-9	-6	8	196.906	84.6341	22
9	6	-8	112.110	66.1192	19
9	6	-7	67.6465	72.7626	19
-9	-6	7	43.1132	79.9992	22
9	6	-7	117.934	66.2860	18
9	-6	-7	9.89187	50.2826	14
9	-6	-7	47.0023	51.4644	12
9	-6	-7	-39.7640	73.7872	11
9	6	-6	452.635	108.714	19
-9	-6	6	498.808	117.912	22
9	6	-6	410.195	99.7881	18
9	-6	-6	598.237	104.761	14
9	-6	-6	322.802	85.3590	12
9	-6	-6	550.464	116.438	11
9	6	-5	14.3845	73.0847	19
-9	-6	5	61.8926	80.1722	22
9	6	-5	-13.8929	70.5871	18
9	-6	-5	10.4179	53.9807	12

9	-6	-5	35.5859	63.7822	14
9	-6	-5	-13.8825	66.1671	11
-9	-6	4	5424.26	411.743	22
9	-6	-4	6176.56	398.789	14
9	-6	-4	5976.17	410.407	11
9	6	-4	5833.65	411.918	19
9	6	-4	5471.94	406.709	18
-9	-6	3	917.251	150.745	22
9	6	-3	946.304	144.397	19
9	6	-3	1039.62	158.835	18
9	-6	-3	1341.30	156.307	14
9	-6	-3	1050.57	147.860	11
9	6	-2	39.8410	66.1480	19
9	6	-2	134.262	88.4183	18
9	-6	-2	54.5388	43.4216	7
9	-6	-2	12.4409	70.4217	14
-9	-6	2	14.9200	77.3374	22
9	6	-1	1099.91	146.634	19
9	-6	-1	1079.85	128.130	7
-9	-6	1	1395.18	171.894	22
9	-6	-1	1457.02	163.974	14
-9	-6	0	716.842	131.222	22
9	6	0	665.879	114.480	19
9	-6	0	579.914	103.581	7
9	-6	0	942.551	130.810	14
9	6	0	780.059	135.529	18
-9	-6	-1	-13.0448	70.1437	22
9	6	1	10.3377	49.2755	19
9	-6	1	-10.3754	60.5681	7

9	-6	1	18.5897	52.5914	5
9	-6	1	47.0172	66.5048	14
9	6	1	66.3841	77.5032	18
-9	-6	-2	1112.43	144.627	22
9	-6	2	893.796	120.321	5
9	6	2	991.185	140.886	18
9	-6	2	1036.69	132.834	7
-9	-6	-3	689.527	114.358	22
9	-6	3	1007.48	125.666	7
9	-6	3	744.639	112.470	5
9	6	3	841.912	121.311	18
9	-6	4	-8.84296	48.4142	5
9	6	4	-51.4483	54.4353	18
9	-6	5	1492.11	137.724	5
9	6	5	1427.72	126.766	18
9	-6	6	1519.73	120.797	7
9	-6	6	1475.69	123.701	5
9	6	6	1168.55	93.9531	18
9	7	-10	23.3113	35.5319	19
-9	-7	10	-11.7706	37.8233	22
9	-7	-9	173.950	60.6391	11
9	7	-9	161.858	58.6813	19
9	-7	-8	66.8255	59.8467	11
9	7	-8	39.3338	52.9283	19
9	-7	-7	1488.78	158.746	11
9	7	-7	1447.38	150.169	18
9	7	-7	1592.71	166.542	19
9	-7	-6	1630.46	170.873	11
9	7	-6	1382.73	161.816	18

9	7	-6	1805.07	180.913	19
9	7	-5	533.525	108.967	19
9	-7	-5	662.701	113.756	11
-9	-7	5	663.862	105.661	27
-9	-7	5	674.780	125.697	22
9	7	-5	760.222	123.354	18
-9	-7	4	8809.31	550.080	27
-9	-7	4	9547.03	579.579	22
9	-7	-4	9171.25	532.543	14
9	-7	-4	9646.67	556.216	11
9	7	-4	9675.59	565.038	19
9	7	-4	8815.78	565.248	18
-9	-7	3	1907.01	201.335	22
9	-7	-3	1750.37	165.892	14
9	7	-3	1673.12	189.787	18
9	7	-3	1332.61	166.344	19
9	-7	-2	747.813	111.592	14
-9	-7	2	790.500	134.036	22
9	7	-2	925.331	126.505	19
9	7	-1	1509.69	177.738	18
9	7	-1	1336.56	148.230	19
-9	-7	1	1480.82	176.611	22
9	-7	-1	1264.45	146.098	14
9	7	0	665.341	99.3975	19
9	7	0	844.532	129.025	18
9	-7	0	706.316	96.6388	7
9	-7	0	734.156	108.950	14
-9	-7	0	816.555	129.180	22
-9	-7	-1	1903.63	185.756	22

9	7	1	1837.98	186.169	18
9	-7	1	1629.53	156.098	7
9	7	2	1046.42	132.295	18
-9	-7	-2	1074.83	135.423	22
9	-7	2	1141.07	124.136	7
9	-7	3	549.697	84.0525	5
-9	-7	-3	510.144	95.3705	22
9	7	3	626.332	90.4373	18
9	-7	3	635.571	92.1913	7
9	-7	4	115.187	52.2525	7
9	-7	4	95.6150	47.3684	5
9	7	4	128.969	47.0019	18
9	-7	5	2213.60	149.179	5
9	7	5	1735.69	123.063	18
9	-7	6	907.377	74.6859	5
9	-8	-9	1309.11	106.253	11
9	8	-9	1461.95	114.349	19
-9	-8	9	1640.32	116.881	22
9	-8	-8	236.683	53.4037	11
9	8	-8	253.106	58.3294	19
-9	-8	8	274.597	65.3969	22
9	8	-7	27.4406	38.8203	18
9	-8	-7	35.2952	43.6387	11
-9	-8	7	17.0772	49.8485	22
9	8	-7	-7.76436	45.3296	19
-9	-8	6	1361.31	145.961	22
9	8	-6	1144.71	130.152	18
9	8	-6	1342.71	135.311	19
9	-8	-6	1092.92	121.571	11

9	8	-5	2570.98	212.377	18
9	8	-5	2416.31	198.291	19
-9	-8	5	2317.04	192.409	27
9	8	-4	2482.78	219.927	18
9	8	-4	2503.93	202.438	19
-9	-8	4	2702.72	215.484	27
9	8	-3	5976.47	362.294	19
-9	-8	3	5550.14	376.843	27
9	8	-2	1733.63	175.968	18
9	8	-2	1437.25	139.756	19
9	8	-1	53.7007	72.3957	18
9	8	-1	6.78830	39.6332	19
9	8	0	5224.40	344.266	18
-9	-8	0	5071.40	344.479	22
-9	-8	-1	410.075	85.9935	22
9	8	1	341.312	82.4282	18
-9	-8	-2	33.1603	55.8959	22
9	8	2	29.0320	47.2457	18
9	8	3	5.37936	35.0012	18
9	8	4	130.521	28.4743	18
9	9	-7	1760.61	125.723	19
-9	-9	7	2553.76	148.215	22
9	9	-6	1088.28	105.271	18
9	9	-6	1050.93	90.8966	19
-9	-9	6	1201.71	108.067	22
9	9	-5	1899.56	158.731	18
9	9	-5	1721.42	132.242	19
-9	-9	5	2102.42	159.046	22
9	9	-4	450.490	60.4930	19

-9	-9	4	520.007	83.0398	22
-9	-9	3	566.966	91.2670	22
-9	-9	3	651.588	91.0487	27
-9	-9	2	1695.41	161.326	27
-9	-9	2	2045.32	168.004	22
9	9	-2	2072.64	170.635	18
-9	-9	1	23.1771	48.4256	22
9	9	-1	53.7635	50.0043	18
9	9	0	3588.40	238.504	18
9	9	1	344.953	59.6274	18
9	9	2	1080.55	87.6219	18
9	10	-5	295.275	41.5600	18
-9	-10	3	1566.74	117.392	22
9	10	-3	2018.66	133.536	18
9	10	-2	474.239	60.6180	18
-9	-10	2	471.052	56.2759	22
-9	-10	1	93.8295	31.6076	22
9	10	-1	47.1141	27.5333	18
9	10	0	132.646	27.7893	18
10	0	-13	-17.8029	21.4208	14
10	0	-12	547.252	77.2650	12
10	0	-11	-19.0365	50.2640	12
10	0	-10	982.606	141.167	12
10	0	-9	45.9693	53.6777	11
10	0	-9	-13.5400	80.2318	12
10	0	-8	23899.2	1260.11	12
10	0	-8	22906.4	1219.30	11
10	0	-7	75.5331	62.6722	11
10	0	-7	-14.7859	73.6086	12

10	0	-7	-55.1618	58.2558	19
10	0	-6	536.507	114.207	11
10	0	-6	605.653	109.520	19
10	0	-6	655.320	127.899	12
10	0	-5	-25.5781	56.6577	19
10	0	-5	-14.7381	73.3757	12
10	0	-5	-14.6539	71.4211	14
10	0	-5	-67.2347	96.6252	1
10	0	-4	49968.4	2425.85	19
10	0	-4	46418.9	2425.78	14
10	0	-4	49441.5	2440.71	12
10	0	-4	45402.8	2884.33	1
10	0	-3	66.8105	74.3684	19
10	0	-3	-14.4672	81.8499	26
10	0	-3	-59.6945	160.484	25
10	0	-3	14.1830	77.6102	12
10	0	-3	40.9739	72.1143	14
10	0	-3	-58.7312	146.182	1
10	0	-2	5560.52	378.890	18
10	0	-2	5973.76	404.205	19
10	0	-2	5217.98	638.422	1
10	0	-2	5601.54	397.179	14
10	0	-2	6072.58	415.356	13
10	0	-2	6275.40	715.266	25
10	0	-2	5851.76	414.837	26
10	0	-1	-11.9465	64.2578	18
10	0	-1	-27.2244	69.1805	13
10	0	-1	-74.8224	69.4265	14
10	0	-1	-26.8837	66.9358	19

-10	0	1	-87.9844	219.000	23
10	0	-1	-30.5824	79.2467	26
10	0	0	565.165	112.996	18
10	0	0	420.541	110.202	13
10	0	0	450.652	98.4985	14
10	0	0	588.550	112.899	19
-10	0	0	474.777	290.860	23
10	0	0	499.253	119.901	26
10	0	1	-25.6224	70.1122	18
10	0	1	-25.2751	66.7352	13
10	0	1	-22.4858	60.4620	14
10	0	2	6537.78	435.302	18
10	0	2	6222.55	410.908	14
10	0	2	7103.27	441.559	6
10	0	3	-59.9632	64.5177	18
10	0	3	-33.7652	73.2609	6
10	0	4	184.788	77.4385	18
10	0	4	316.687	86.1502	7
10	0	4	267.981	77.7191	6
10	0	5	8.10744	47.3255	6
10	0	5	-15.5315	49.9077	7
10	0	6	10.3354	36.3836	7
10	0	7	30.0994	34.9752	5
10	-1	-13	78.2070	24.8468	12
10	1	-13	64.8461	22.4607	14
10	1	-12	1168.88	99.7807	12
10	-1	-12	1368.40	115.917	12
-10	-1	11	794.922	102.374	22
10	1	-11	743.200	102.225	12

10	-1	-11	932.079	122.342	12
-10	-1	10	4892.33	345.224	22
10	1	-10	5471.57	357.786	12
10	-1	-10	5984.30	382.605	12
10	-1	-10	4917.31	325.774	11
-10	-1	9	1656.41	179.312	22
10	1	-9	1990.82	196.529	12
10	-1	-9	1733.03	198.605	12
10	-1	-9	1903.19	182.200	11
10	1	-8	976.542	147.827	12
10	-1	-8	764.736	142.808	12
10	1	-8	947.246	126.762	19
-10	-1	8	1265.82	152.735	22
10	-1	-8	942.376	136.186	11
10	1	-7	-13.8810	74.6550	12
10	-1	-7	60.5391	84.2506	12
10	-1	-7	96.0282	75.0859	11
10	1	-7	12.7809	59.5379	19
-10	-1	7	51.1626	63.6942	22
10	1	-6	238.777	81.6640	12
10	-1	-6	259.271	96.3169	12
10	-1	-6	292.481	97.2021	14
10	-1	-6	257.682	93.9488	11
10	-1	-6	305.210	73.1788	19
10	1	-6	354.234	93.6771	19
-10	-1	6	164.770	72.7505	22
10	-1	-5	21.8101	49.5824	19
10	1	-5	14.0415	71.3587	19
10	-1	-5	29.1829	67.9646	11

-10	1	5	70.7087	160.668	30
10	1	-5	125.647	72.3528	12
10	-1	-5	15.0992	71.9825	12
10	-1	-5	15.1870	67.2864	14
10	1	-5	13.7066	69.6575	14
10	-1	-5	133.330	179.231	1
10	1	-5	67.4837	167.975	1
10	-1	-4	4716.62	319.310	19
10	1	-4	3764.22	318.918	19
10	-1	-4	3972.38	328.273	14
10	1	-4	3712.48	311.462	14
10	1	-4	4305.66	324.202	12
10	-1	-4	4627.23	341.283	12
10	1	-4	3645.76	557.879	1
10	-1	-4	3973.17	578.542	1
10	-1	-3	2142.63	208.861	19
10	1	-3	1986.11	199.074	18
10	1	-3	2327.84	226.732	19
10	1	-3	2252.24	422.437	25
10	1	-3	2298.20	230.998	26
10	1	-3	2370.36	219.567	14
10	-1	-3	2426.11	231.357	14
10	1	-3	2324.04	229.157	12
10	-1	-3	2512.12	235.765	12
10	1	-3	2429.74	423.956	1
10	-1	-3	2843.15	464.368	1
10	-1	-2	13833.9	750.245	19
10	1	-2	13197.5	745.914	18
10	1	-2	13621.2	765.016	19

10	-1	-2	12955.3	1094.71	1
10	-1	-2	13461.8	769.508	13
10	1	-2	12828.5	738.082	14
10	-1	-2	11987.5	748.585	14
10	1	-2	12458.7	749.960	13
10	1	-2	11311.0	1073.00	25
10	-1	-2	12288.7	1102.18	25
10	1	-2	13878.1	775.016	26
10	-1	-2	12851.7	766.004	26
10	-1	-1	74.5943	62.2068	18
10	1	-1	-25.8937	70.8485	18
10	-1	-1	-69.1059	75.6326	13
10	1	-1	65.3949	72.7949	13
10	1	-1	-11.3173	59.7586	14
10	-1	-1	13.2652	64.6470	14
10	1	-1	-55.9140	71.0228	19
-10	1	1	87.2939	177.405	23
10	1	-1	-15.1991	80.2533	26
10	-1	-1	59.8650	76.0411	26
10	1	-1	-188.535	156.422	25
10	-1	-1	-61.9597	140.782	25
10	1	0	479.944	111.688	18
10	1	0	850.306	131.986	19
10	-1	0	588.343	104.611	18
10	-1	0	698.663	125.050	13
10	1	0	538.963	107.789	13
10	1	0	701.949	107.428	14
10	-1	0	862.369	128.539	14
-10	1	0	625.887	288.221	23

10	1	0	449.635	123.729	26
10	-1	0	761.846	139.178	26
10	1	1	2748.80	260.209	26
10	-1	1	2659.78	256.400	26
10	-1	1	2586.92	229.333	18
10	1	1	2691.10	241.424	18
10	1	1	2424.64	209.356	14
10	-1	1	2609.45	230.764	14
10	-1	1	2807.21	245.956	13
10	1	1	2451.10	227.769	13
10	-1	2	5665.03	385.350	6
10	1	2	5287.43	370.619	6
10	1	2	5327.06	402.022	26
10	-1	2	5777.69	408.859	26
10	-1	2	5618.08	378.408	18
10	1	2	5224.34	382.579	18
10	1	2	5451.65	350.836	14
10	-1	2	5606.82	373.911	14
10	-1	3	6322.62	409.175	6
10	1	3	5635.76	390.108	6
10	-1	3	6239.10	408.156	7
10	1	3	6607.46	446.646	26
10	-1	3	5490.57	421.226	26
10	-1	3	6670.31	410.738	18
10	1	3	6401.95	416.075	18
10	-1	4	311.863	85.5130	7
10	1	4	112.559	67.4804	7
10	-1	4	204.620	75.6453	6
10	1	4	221.244	73.2590	6

10	1	4	194.133	79.1004	26
10	-1	4	225.923	84.9360	26
10	-1	4	304.378	86.5770	18
10	1	5	5037.69	325.504	6
10	-1	5	5374.54	335.168	7
10	1	5	5165.39	324.945	7
10	-1	5	5488.35	364.392	26
10	1	5	5443.57	367.422	26
10	1	5	5870.58	352.804	18
10	-1	6	166.986	61.4535	5
10	1	6	18.0414	42.2563	26
10	-1	6	136.513	56.3742	26
10	1	6	150.174	59.2123	18
10	-1	6	111.853	45.6953	7
10	1	6	90.0413	34.8864	7
10	-1	7	4.95496	33.3990	5
10	1	7	-14.2717	37.4449	5
10	-1	7	14.4594	18.3275	7
-10	-2	12	29.2723	31.7969	22
10	-2	-12	92.2623	42.4302	12
10	-2	-12	-5.83128	35.6003	14
10	2	-12	48.7670	24.2839	12
-10	-2	11	174.280	58.4033	22
-10	-2	11	57.0331	54.2894	29
10	2	-11	240.626	55.9667	12
10	-2	-11	165.430	66.5527	12
10	-2	-11	240.381	52.5924	11
10	2	-10	158.517	62.5488	12
10	-2	-10	257.249	87.7050	12

10	-2	-10	173.299	60.6333	11
-10	-2	10	152.004	69.3948	22
10	2	-9	890.915	122.947	12
10	-2	-9	839.395	142.378	12
10	2	-9	941.888	125.365	19
10	-2	-9	919.315	131.980	11
-10	-2	9	851.834	130.964	22
10	2	-8	11.7930	56.2342	12
10	2	-8	25.2541	58.8242	19
10	-2	-8	53.3574	70.4534	11
-10	-2	8	80.5196	70.8788	22
10	2	-7	2598.61	230.915	12
10	-2	-7	3098.02	265.656	14
10	2	-7	2429.54	235.236	19
-10	-2	7	2189.43	227.348	22
10	-2	-7	2545.96	243.326	11
10	2	-6	434.433	102.147	19
-10	-2	6	570.157	113.375	22
10	-2	-6	473.530	109.567	11
10	2	-6	355.689	95.5940	12
10	-2	-6	568.588	117.834	14
10	2	-6	441.637	100.045	14
10	2	-5	281.450	92.9712	19
10	-2	-5	321.836	102.206	14
10	2	-5	294.215	86.0147	14
10	2	-5	356.995	89.3395	12
-10	-2	5	491.401	108.645	22
10	-2	-5	360.046	103.526	11
-10	-2	4	330.563	92.1740	22

10	2	-4	386.833	102.358	19
10	2	-4	123.266	140.723	1
10	-2	-4	325.571	187.654	1
10	2	-4	324.110	84.5959	18
10	2	-4	316.185	86.3659	12
10	-2	-4	256.033	94.1148	14
10	2	-4	199.506	71.8975	14
10	-2	-4	347.509	100.747	13
10	-2	-4	359.670	104.207	11
10	-2	-3	2605.74	234.673	13
10	2	-3	2152.86	198.931	14
10	-2	-3	2144.69	224.713	14
-10	-2	3	2100.25	208.320	22
10	-2	-3	2086.39	414.487	1
10	2	-3	2025.07	223.306	19
10	2	-3	2358.31	216.163	18
10	2	-2	3622.47	295.359	19
10	2	-2	3492.98	281.890	18
10	-2	-2	3923.80	302.895	13
10	2	-2	3229.07	256.271	14
10	-2	-2	3215.31	284.748	14
10	2	-2	3174.65	274.175	13
10	-2	-2	3824.47	538.090	1
10	2	-2	3895.74	543.926	25
10	-2	-2	2993.57	477.433	25
10	2	-2	3565.09	294.611	26
10	-2	-2	3611.45	292.012	26
-10	-2	2	3260.08	269.571	22
10	2	-1	-14.6663	83.0025	26

10	-2	-1	71.1426	80.5268	26
-10	-2	1	79.3653	180.341	23
-10	-2	1	11.3827	56.7027	22
10	2	-1	48.7859	67.9134	13
10	-2	-1	54.8907	76.4040	14
10	2	-1	122.468	80.6527	18
10	-2	-1	157.955	131.067	1
10	-2	0	10251.2	624.382	14
10	2	0	10153.9	606.514	13
10	2	0	10424.9	640.903	26
10	-2	0	10797.5	641.284	26
10	-2	0	10574.3	624.867	6
10	-2	0	10167.2	595.460	18
10	2	0	10400.8	631.630	18
10	-2	1	14043.8	799.658	18
10	2	1	14641.2	831.186	18
10	2	1	14731.5	825.923	19
10	-2	1	14301.1	817.731	14
10	-2	1	14349.7	827.483	13
10	2	1	14803.6	803.259	13
10	2	1	14238.9	842.438	26
10	-2	1	14889.8	840.190	26
10	-2	1	15395.6	829.691	6
10	-2	2	5438.55	357.962	14
10	2	2	5291.23	383.498	26
10	-2	2	4834.66	362.620	26
10	-2	2	4955.87	341.533	18
10	2	2	4449.17	350.900	18
10	-2	2	5134.83	360.874	7

10	2	2	4786.59	339.498	6
10	-2	3	799.752	123.855	14
10	2	3	1010.22	146.300	26
10	-2	3	655.025	119.382	26
10	-2	3	718.999	119.923	7
10	2	3	690.130	110.294	6
10	-2	3	738.543	121.653	6
10	-2	3	949.853	127.563	18
10	2	4	422.498	98.6014	26
10	-2	4	379.528	92.4507	26
10	2	4	520.553	99.8454	18
10	2	4	476.078	84.8792	7
10	-2	4	433.731	90.4848	6
10	2	4	348.421	75.9550	6
10	-2	4	502.713	98.2006	7
10	-2	5	339.366	77.1293	5
10	2	5	150.102	64.7994	26
10	-2	5	359.981	76.3687	26
10	2	5	279.095	57.2732	7
10	-2	5	292.414	72.0673	7
10	2	5	253.710	72.8028	18
10	2	6	187.306	57.2052	26
10	-2	6	256.352	57.1302	26
10	2	6	263.605	59.7942	18
10	-2	6	236.395	54.7810	7
10	-2	7	2891.87	167.522	5
10	-2	7	2139.22	138.116	7
-10	-3	12	13.6011	27.6643	22
10	-3	-12	-14.4477	30.5816	12

10	-3	-12	-44.6915	28.3257	14
-10	-3	11	435.781	76.0575	22
10	-3	-11	405.500	75.3309	12
10	-3	-11	508.487	77.7834	14
10	-3	-11	420.682	67.9849	11
10	-3	-10	1879.41	173.589	12
10	-3	-10	1839.55	167.856	14
10	3	-10	1199.72	119.892	12
10	-3	-10	1624.51	156.844	11
-10	-3	10	1567.58	163.159	22
10	3	-10	1645.80	149.623	19
10	3	-9	278.398	64.9269	12
10	-3	-9	266.426	94.1379	12
10	-3	-9	263.416	86.5088	14
-10	-3	9	409.772	98.9080	22
10	-3	-9	367.928	93.8714	11
10	3	-9	417.239	91.4938	19
10	-3	-8	293.431	90.6694	12
10	-3	-8	147.522	81.4782	14
10	3	-8	271.873	71.6245	12
-10	-3	8	190.108	78.9392	22
10	-3	-8	229.177	87.4348	11
10	3	-8	443.315	101.588	19
10	-3	-7	4474.62	332.965	12
10	3	-7	3362.50	280.682	14
10	3	-7	4279.96	294.888	12
10	-3	-7	4004.28	318.681	11
10	3	-7	4278.19	325.306	19
-10	-3	7	4113.29	324.468	22

10	-3	-6	1528.90	189.110	12
10	-3	-6	1675.95	195.368	11
10	3	-6	1610.82	164.595	14
10	3	-6	1591.43	167.376	12
-10	-3	6	1434.23	182.239	22
10	3	-6	1828.14	198.942	19
10	-3	-5	1381.46	170.204	13
10	3	-5	1294.45	149.703	12
10	-3	-5	1257.56	168.501	12
-10	-3	5	1079.52	158.698	22
10	3	-5	1071.10	136.020	18
10	3	-5	1188.36	165.681	19
10	-3	-5	1155.47	166.027	11
10	3	-4	1810.54	187.132	18
10	3	-4	2015.76	210.785	19
10	-3	-4	1886.98	202.963	13
10	-3	-4	1913.86	211.916	11
10	-3	-4	2117.14	213.908	12
-10	-3	4	1903.80	205.964	22
10	-3	-4	1563.99	160.968	7
10	-3	-3	405.321	104.423	13
10	-3	-3	421.602	113.985	12
10	-3	-3	306.850	102.422	14
10	3	-3	317.131	92.9771	13
10	-3	-3	372.298	87.8051	7
-10	-3	3	422.326	104.352	22
10	3	-3	617.456	115.579	18
10	-3	-2	4541.67	327.112	7
10	-3	-2	5103.24	337.011	6

10	3	-2	4291.33	335.883	18
10	3	-2	4261.96	321.031	13
10	-3	-2	4574.99	349.064	14
10	-3	-2	4794.01	344.419	13
10	3	-2	4927.04	346.148	26
-10	-3	2	3794.78	321.291	22
-10	-3	1	-12.4383	56.5306	22
10	3	-1	56.1644	71.3439	19
10	-3	-1	13.2042	71.0032	13
10	3	-1	32.5281	60.3529	13
10	-3	-1	-27.4868	71.2141	14
10	-3	-1	24.6261	69.6681	7
10	-3	-1	48.7253	63.1213	6
10	3	-1	27.2205	64.8739	26
10	-3	-1	90.8238	63.2355	26
10	3	-1	-27.8690	83.7724	18
10	3	0	264.055	99.1733	18
-10	-3	0	141.706	69.3895	22
10	3	0	147.277	83.1372	19
10	-3	0	326.324	94.9165	7
10	-3	0	135.018	82.1739	6
10	-3	0	181.361	82.5899	13
10	3	0	141.591	63.8495	13
10	-3	0	158.502	87.3459	14
10	-3	0	178.622	70.0002	5
10	3	0	124.108	74.5725	26
10	-3	0	169.277	74.0987	26
-10	-3	-1	9899.58	604.894	22
10	3	1	10907.6	630.074	19

10	3	1	10479.8	635.261	18
10	-3	1	10768.7	631.922	14
10	-3	1	10038.7	599.386	5
10	-3	1	10558.4	622.179	6
10	-3	1	10716.1	627.898	7
10	3	1	11786.9	652.511	26
10	-3	1	10241.0	626.521	26
-10	-3	-2	2139.42	192.202	22
10	3	2	2273.63	217.817	26
10	-3	2	2119.78	208.114	26
10	-3	2	2287.36	213.981	14
10	-3	2	2410.32	215.707	6
10	3	2	1998.92	188.611	6
10	-3	2	2264.39	201.738	5
10	3	3	8163.31	536.960	18
10	-3	3	9606.61	537.175	5
10	-3	3	9796.38	545.890	7
10	3	3	8811.97	511.665	6
10	3	3	8832.92	550.191	26
10	-3	3	9011.51	541.281	26
10	-3	4	727.503	108.857	5
10	3	4	694.706	95.0589	7
10	3	4	834.701	93.7176	6
10	-3	4	855.502	114.963	7
10	3	4	802.599	116.114	18
10	3	4	1028.81	130.139	26
10	-3	4	1006.91	121.175	26
10	-3	5	325.175	70.6053	5
10	3	5	208.218	64.4683	26

10	-3	5	299.269	62.7353	26
10	3	5	356.739	74.1612	18
10	-3	5	297.936	71.0492	7
10	3	5	264.295	48.7359	7
10	3	6	5551.27	313.534	18
10	-3	6	5142.45	306.790	7
10	-3	7	4.81699	16.4230	7
10	-3	7	23.1228	28.7796	5
-10	-4	12	183.221	34.0216	22
10	-4	-11	34.0018	40.8706	12
10	-4	-11	85.1728	41.7559	11
-10	-4	11	116.204	46.7271	22
10	-4	-10	549.582	89.5126	12
10	-4	-10	647.512	91.3339	14
-10	-4	10	665.897	102.376	22
10	-4	-10	602.637	96.3548	11
10	4	-10	658.843	94.4327	19
10	-4	-9	3781.91	274.106	12
10	-4	-9	3606.41	268.106	14
10	-4	-9	3856.07	281.248	11
-10	-4	9	3806.23	283.455	22
10	4	-9	3241.45	265.498	19
10	-4	-8	57.8653	63.4237	12
10	-4	-8	46.7179	64.0050	14
-10	-4	8	104.290	82.7978	22
10	-4	-8	102.669	70.3123	11
10	4	-8	75.7670	61.6361	19
10	-4	-7	5842.09	392.173	12
10	-4	-7	5506.42	365.655	13

10	-4	-7	6040.52	398.761	14
10	-4	-7	5734.60	401.738	11
-10	-4	7	5332.24	394.043	22
10	4	-7	5313.04	391.233	19
-10	-4	6	215.043	86.9456	22
10	-4	-6	314.022	95.8012	11
10	-4	-6	261.985	80.8086	13
10	-4	-6	298.892	94.3342	14
10	-4	-6	169.098	77.9228	12
10	-4	-5	243.380	80.6990	13
10	-4	-5	265.266	94.8062	14
10	-4	-5	250.894	89.7475	12
10	4	-5	220.246	74.4564	18
-10	-4	5	260.478	91.3739	22
10	4	-4	6323.32	428.863	18
-10	-4	4	5887.64	434.879	22
10	-4	-4	6452.88	392.105	6
10	-4	-4	6469.39	424.764	13
10	-4	-4	6809.42	449.293	14
10	-4	-4	7120.57	447.166	12
10	4	-3	15522.4	835.947	18
10	4	-3	14833.4	838.402	19
10	-4	-3	16234.8	828.449	13
10	4	-3	13042.7	788.310	13
-10	-4	3	15035.5	838.383	22
10	-4	-3	13941.1	779.430	7
10	-4	-3	14610.5	790.746	6
10	4	-2	879.847	143.860	19
10	-4	-2	844.541	130.093	13

10	4	-2	848.391	115.245	13
10	-4	-2	901.504	120.181	7
10	-4	-2	932.228	124.951	6
-10	-4	2	1022.19	146.210	22
10	4	-2	853.696	138.520	18
10	4	-1	15878.6	863.622	18
10	4	-1	15438.8	857.371	19
10	4	-1	15266.2	839.340	26
10	-4	-1	16451.4	853.180	13
10	4	-1	12749.5	792.127	13
10	-4	-1	15960.0	833.897	6
10	-4	-1	15766.4	833.272	7
-10	-4	1	15075.7	845.358	22
10	4	0	3389.27	271.080	19
10	4	0	3293.94	280.131	18
10	-4	0	2684.46	221.010	5
10	-4	0	3496.60	263.290	6
10	-4	0	3276.84	257.867	7
-10	-4	0	3252.78	265.024	22
10	4	0	3546.98	268.736	26
10	-4	0	3267.48	255.157	26
-10	-4	-1	13257.0	723.215	22
10	4	1	12008.1	718.242	26
10	-4	1	13148.2	714.224	26
10	-4	1	13644.2	736.631	14
10	4	1	13115.0	725.766	19
10	-4	1	11355.1	688.635	5
10	-4	1	13426.0	723.265	6
10	-4	1	12781.2	721.096	7

10	-4	2	1614.00	162.202	6
10	-4	2	1560.14	164.152	7
10	4	2	1380.60	139.911	6
10	4	2	1187.96	148.663	26
10	-4	2	1599.39	155.392	26
10	-4	2	1757.35	174.189	14
10	-4	2	1272.71	143.710	5
-10	-4	-2	1356.38	151.717	22
10	-4	3	5067.05	333.070	5
10	4	3	5082.64	340.586	26
10	4	3	5689.26	357.397	18
10	4	3	4840.67	304.616	6
10	-4	4	700.231	100.845	5
10	4	4	698.635	98.5892	26
10	4	4	941.707	114.136	18
10	4	4	762.732	84.1679	7
10	-4	4	730.046	104.942	7
10	-4	5	6.89952	42.0713	5
10	4	5	59.3851	37.3379	26
10	4	5	-6.70750	43.1125	18
10	-4	5	-6.66804	42.3199	7
10	-4	6	183.201	44.1366	7
10	-4	6	101.762	42.5003	5
10	4	6	133.598	36.4267	18
-10	-5	11	-20.1204	30.6674	22
10	5	-11	22.0755	23.3475	19
10	-5	-11	-8.72581	25.4672	11
-10	-5	10	-7.84120	48.4667	22
10	-5	-10	-7.31716	42.7133	11

10	5	-10	-6.94144	39.2732	19
10	-5	-9	6660.59	385.133	14
10	-5	-9	6557.32	385.795	12
10	-5	-9	6452.85	407.972	11
-10	-5	9	6841.61	414.462	22
10	5	-9	6282.99	402.634	19
10	-5	-8	1187.65	132.423	14
10	-5	-8	1129.20	128.049	12
10	-5	-8	1144.90	142.686	11
-10	-5	8	960.022	136.769	22
10	-5	-7	434.670	91.1363	14
10	-5	-7	362.829	84.3538	12
-10	-5	7	269.488	90.1105	22
10	-5	-7	420.758	108.819	11
10	-5	-6	476.187	101.092	14
10	-5	-6	409.823	88.1857	12
10	5	-6	315.767	95.3355	19
10	5	-6	365.156	82.8712	18
-10	-5	6	378.356	104.348	22
10	-5	-5	4167.62	294.271	13
10	-5	-5	4766.16	338.966	11
10	-5	-5	5053.02	340.843	14
10	-5	-5	4554.12	320.738	12
10	5	-5	3828.63	310.339	18
10	5	-5	5004.23	346.191	19
-10	-5	5	4292.26	335.843	22
10	-5	-4	4698.20	330.616	14
10	-5	-4	4201.61	325.502	11
10	-5	-4	4710.76	304.759	13

10	5	-4	4067.25	317.732	18
10	5	-4	4277.49	329.940	19
-10	-5	4	3749.36	319.623	22
10	-5	-3	4093.38	302.761	14
10	-5	-3	4390.28	287.266	13
-10	-5	3	3248.95	291.144	22
10	5	-3	3312.68	287.975	18
10	5	-3	3877.71	303.994	19
10	5	-2	601.278	119.025	19
10	-5	-2	760.610	111.697	13
10	-5	-2	919.097	128.241	14
10	-5	-2	657.766	96.5487	7
-10	-5	2	696.074	126.945	22
10	5	-2	770.056	132.422	18
10	5	-1	-12.0306	63.5342	19
10	-5	-1	-12.1399	67.5775	14
10	-5	-1	-35.5431	51.8839	6
10	-5	-1	-46.2847	57.9981	7
-10	-5	1	-37.6577	71.0284	22
10	5	-1	26.2368	70.5474	18
10	-5	0	2993.53	222.803	6
10	-5	0	2681.44	218.961	7
10	-5	0	2914.90	238.626	14
10	5	0	2728.39	228.512	19
10	5	0	2680.11	213.075	26
-10	-5	0	2298.40	222.315	22
10	5	1	5547.60	352.898	26
-10	-5	-1	5585.63	373.026	22
10	-5	1	5603.03	362.463	7

10	-5	2	2537.74	207.151	7
10	-5	2	2445.77	195.441	5
10	5	2	2554.53	220.180	18
-10	-5	-2	2652.57	214.156	22
10	-5	3	84.1749	56.8848	7
10	-5	3	62.1120	54.8127	5
10	5	3	127.969	65.1463	18
10	-5	4	20.6792	42.6053	5
10	5	4	-14.5303	46.6902	18
10	5	5	2043.31	145.751	18
10	-5	5	2033.69	150.425	7
10	-5	5	1956.20	148.590	5
10	-5	6	87.7403	28.1128	5
10	-5	6	74.8280	27.0412	7
10	-6	-10	181.670	46.8653	11
10	6	-10	149.240	41.7227	19
-10	-6	10	143.360	43.6627	22
10	-6	-9	60.0063	54.0242	11
-10	-6	9	102.034	57.6329	22
10	6	-8	5877.53	382.142	19
-10	-6	8	6505.55	393.768	22
10	-6	-7	1058.64	112.168	14
10	-6	-7	1201.17	136.293	11
-10	-6	7	757.463	122.244	22
10	6	-7	1056.77	133.607	19
10	6	-6	752.185	116.795	19
-10	-6	6	904.799	132.311	22
10	-6	-6	804.625	106.210	14
10	6	-6	717.257	105.409	18

10	-6	-6	864.266	123.487	11
-10	-6	5	4188.76	328.406	22
10	-6	-5	4784.03	315.643	14
10	-6	-5	5144.09	333.829	11
10	6	-5	4334.35	316.058	18
10	6	-5	4394.67	325.036	19
10	-6	-4	61.0799	60.3461	14
10	6	-4	35.0763	65.1008	19
10	6	-4	46.3017	60.0051	18
-10	-6	4	12.4839	67.1508	22
10	-6	-3	501.381	94.3520	14
10	6	-3	388.629	96.0292	19
10	6	-3	540.166	109.127	18
-10	-6	3	395.704	98.0329	22
10	-6	-2	1193.89	141.923	14
10	6	-2	1377.64	153.092	19
-10	-6	2	1289.07	157.634	22
10	6	-2	1026.46	147.611	18
10	-6	-1	133.851	64.7505	14
10	6	-1	140.973	66.5596	19
-10	-6	1	92.3719	65.5781	22
10	6	0	2766.13	219.970	19
10	-6	0	2879.20	211.424	7
-10	-6	0	2767.95	234.351	22
10	-6	0	3184.78	237.722	14
10	6	1	3133.07	247.306	18
10	-6	1	3149.00	227.123	7
-10	-6	-1	2828.94	236.286	22
10	-6	1	3610.32	250.419	14

10	-6	2	7274.89	424.056	7
-10	-6	-2	7356.89	437.012	22
10	6	2	7359.34	438.735	18
10	6	3	326.433	69.1777	18
10	-6	3	234.265	61.8187	7
10	6	4	195.297	48.6560	18
10	-6	4	82.8535	41.3150	7
10	-6	5	36.6202	24.4374	5
10	6	5	27.5380	19.2390	18
10	-7	-9	1329.74	109.129	11
-10	-7	9	1650.93	120.208	22
10	7	-9	1427.36	112.389	19
-10	-7	8	41.6678	42.9443	22
10	7	-8	-6.51727	39.7564	19
10	-7	-8	62.6265	42.7093	11
-10	-7	7	1500.84	147.646	22
10	-7	-7	1408.20	133.521	11
10	7	-7	1313.02	131.850	19
-10	-7	6	1047.16	128.054	22
10	-7	-6	1044.92	117.412	11
10	7	-6	948.748	119.698	19
10	7	-5	72.3326	59.5435	19
-10	-7	5	71.2942	60.3477	22
10	7	-5	-35.6086	56.4929	18
10	7	-4	209.643	66.8615	19
10	7	-4	195.184	72.1742	18
-10	-7	4	219.899	78.0765	22
10	-7	-3	224.019	59.1505	14
10	7	-3	238.524	65.5144	19

10	7	-3	243.816	77.2190	18
-10	-7	3	146.820	71.4033	22
-10	-7	2	2134.81	190.118	22
10	-7	-2	1980.97	167.162	14
10	7	-2	1970.91	185.834	18
10	7	-2	2073.75	173.227	19
-10	-7	1	1177.87	136.470	22
10	-7	-1	1053.87	117.099	14
10	7	-1	956.697	109.054	19
-10	-7	0	3135.88	229.992	22
10	7	0	2750.65	221.894	18
-10	-7	-1	4502.40	302.540	22
10	7	1	4993.45	308.113	18
-10	-7	-2	1392.35	128.612	22
10	7	2	1382.18	126.215	18
10	7	3	708.306	79.0923	18
10	-7	3	798.555	80.2033	7
10	7	4	92.9515	24.8723	18
10	8	-8	17.0291	17.1022	19
-10	-8	8	40.5241	23.8030	22
10	8	-7	21.8709	31.1302	19
10	8	-6	1003.83	95.0363	19
10	8	-5	1052.00	107.585	18
10	8	-5	1058.87	100.890	19
10	8	-4	335.301	69.4504	18
10	8	-4	258.762	54.4043	19
-10	-8	4	226.244	65.1544	22
10	8	-3	92.1355	52.4632	18
10	8	-3	76.2104	38.8915	19

-10	-8	3	63.6037	56.6686	22
10	8	-2	451.552	62.6020	19
-10	-8	2	711.073	94.8261	22
-10	-8	1	2945.44	209.196	22
10	8	-1	2800.85	205.958	18
-10	-8	0	2635.77	191.984	22
10	8	0	2590.03	187.925	18
10	8	1	10.7078	34.4184	18
-10	-8	-1	29.3400	37.2469	22
10	8	2	1044.61	86.5338	18
10	9	-5	203.094	32.9034	18
-10	-9	5	246.387	39.2998	22
10	9	-4	1857.50	129.144	18
-10	-9	4	1868.98	132.800	22
10	9	-2	36.4613	32.1316	18
10	9	-1	159.525	36.5334	18
10	9	0	242.187	36.9562	18
11	0	-12	-12.4297	18.6895	14
11	0	-11	1829.83	143.441	14
11	0	-11	1656.41	136.256	12
11	0	-10	41.0903	51.4770	12
11	0	-10	-26.0974	51.5427	14
11	0	-9	976.284	129.419	12
11	0	-9	974.784	130.326	14
11	0	-8	-11.2832	58.4640	12
11	0	-8	45.8500	65.8898	14
11	0	-7	618.861	111.295	14
11	0	-7	496.559	105.499	12
11	0	-6	24.9308	63.3379	14

11	0	-6	50.4464	70.1958	12
11	0	-6	-65.6314	52.1890	19
11	0	-5	26765.3	1439.14	14
11	0	-5	28335.5	1450.47	12
11	0	-5	28128.5	1421.58	19
11	0	-4	-12.2824	63.6385	14
11	0	-4	26.5015	78.5110	13
11	0	-4	11.1234	57.6337	19
11	0	-3	-12.9765	67.2844	13
11	0	-3	118.734	67.2243	14
11	0	-3	57.6994	58.6871	19
11	0	-2	13.5173	62.9445	26
11	0	-2	62.7291	76.4900	13
11	0	-2	45.0871	67.7614	14
11	0	-2	-23.4251	65.1882	19
11	0	-2	-8.52959	47.4735	18
11	0	-1	109.361	83.5110	26
11	0	-1	120.346	83.1071	13
11	0	-1	63.6087	64.8503	14
11	0	-1	152.949	61.6606	18
11	0	0	-45.5898	66.5300	13
11	0	0	9.82639	56.4837	14
11	0	0	10.0115	58.4399	18
11	0	1	10.8764	67.2289	6
11	0	1	17.9162	44.5982	14
11	0	1	9.97680	52.6811	18
11	0	2	66.3689	59.3948	18
11	0	2	19.6336	60.6820	6
11	0	2	-30.2923	62.4163	7

11	0	3	6381.89	397.320	18
11	0	3	6723.00	401.448	6
11	0	3	6734.83	401.739	7
11	0	4	-21.7101	44.1230	18
11	0	4	6.77557	45.1494	6
11	0	4	-13.2900	46.2929	7
11	0	5	3459.24	210.068	7
11	0	5	3589.76	224.272	5
11	-1	-12	193.109	30.9868	12
11	-1	-12	223.934	33.8286	14
11	-1	-11	116.590	44.1100	14
11	1	-11	189.755	45.2071	14
11	1	-11	97.8898	35.3090	12
11	-1	-11	168.011	48.0453	12
11	-1	-10	352.273	76.2731	12
11	-1	-10	233.989	68.3720	14
11	1	-10	313.287	68.8286	14
11	1	-10	253.437	61.7273	12
11	1	-9	2635.50	210.399	12
11	-1	-9	2780.97	225.204	12
11	-1	-9	2695.31	223.739	14
11	1	-9	2705.72	218.377	14
11	-1	-8	1679.69	176.603	12
11	-1	-8	1634.83	177.351	14
11	1	-8	1641.04	168.835	14
11	1	-8	1690.70	170.747	12
11	1	-7	3056.44	239.807	19
11	-1	-7	3573.20	274.649	12
11	-1	-7	3509.60	274.761	14

11	1	-7	2644.16	242.179	14
11	1	-7	3524.30	265.005	12
11	1	-6	687.451	110.611	19
11	1	-6	493.150	101.168	12
11	-1	-6	465.019	109.205	14
11	1	-6	537.006	102.615	14
11	-1	-6	361.393	96.5790	12
11	1	-5	3138.45	256.844	19
11	1	-5	3052.12	252.741	14
11	-1	-5	3516.33	277.421	14
11	1	-5	3053.89	256.190	12
11	-1	-5	3270.97	272.341	12
11	-1	-4	13336.1	725.872	19
11	1	-4	12796.3	748.266	19
11	1	-4	12863.3	738.838	14
11	-1	-4	13090.4	758.893	14
11	1	-4	13390.1	761.553	13
11	-1	-4	14128.1	771.790	12
11	1	-3	4281.23	311.752	19
11	-1	-3	4308.56	321.964	13
11	1	-3	3553.86	284.076	14
11	1	-3	4057.88	309.450	13
11	-1	-3	4295.09	316.031	14
11	1	-2	19440.5	1007.86	26
11	1	-2	17550.6	960.529	18
11	1	-2	18837.5	993.596	19
11	-1	-2	18960.1	1000.31	13
11	1	-2	17742.0	983.037	13
11	1	-2	17037.9	959.864	14

11	-1	-2	18776.9	991.487	14
11	1	-1	17374.4	963.287	26
11	-1	-1	17701.9	965.425	26
11	1	-1	17771.7	937.645	18
11	1	-1	18644.8	959.450	19
11	1	-1	16018.9	915.277	14
11	-1	-1	17123.5	943.464	14
11	-1	-1	18232.4	957.506	13
11	1	-1	17429.7	944.170	13
11	-1	0	142.171	59.3130	18
11	-1	0	174.798	78.2514	13
11	1	0	235.764	77.6082	13
11	1	0	186.309	65.6878	14
11	-1	0	213.280	74.1306	14
11	1	0	295.472	90.0147	26
11	-1	0	276.243	90.1890	26
11	1	0	225.249	81.4101	18
11	1	1	11739.1	644.646	14
11	-1	1	12086.6	670.830	14
11	1	1	12451.6	681.484	6
11	1	1	12125.3	704.104	26
11	-1	1	12619.0	704.818	26
11	-1	1	11352.9	657.976	18
11	1	1	12106.6	678.247	18
11	-1	2	873.616	122.566	7
11	1	2	793.712	111.802	6
11	-1	2	970.274	125.558	6
11	1	2	809.959	125.077	26
11	-1	2	794.863	125.593	26

11	-1	2	922.100	112.981	18
11	1	2	971.524	122.558	18
11	-1	3	111.655	59.8378	7
11	1	3	8.15984	50.4417	7
11	-1	3	60.5447	59.6140	6
11	1	3	47.4128	49.5046	6
11	1	3	-26.1399	52.3865	18
11	1	3	-33.5070	56.7517	26
11	-1	3	31.9547	50.7729	26
11	-1	4	980.349	110.695	5
11	1	4	1085.32	105.754	6
11	-1	4	1128.15	111.833	7
11	1	4	969.293	102.916	7
11	1	4	1118.30	115.112	18
11	1	4	1098.51	123.946	26
11	-1	4	1046.23	118.529	26
11	-1	5	4236.76	253.289	5
11	-1	5	4217.85	245.671	7
11	1	5	4097.97	234.236	7
11	1	6	331.637	43.2284	5
11	2	-11	3.61783	21.1252	14
11	-2	-11	32.7686	33.7609	12
11	-2	-10	722.787	97.0184	12
11	2	-10	602.844	78.2010	14
11	-2	-10	518.123	77.6450	13
11	2	-10	701.375	74.3212	12
-11	-2	10	463.413	68.2062	22
11	-2	-9	749.268	107.428	12
11	2	-9	695.622	89.6103	12

11	-2	-9	731.143	109.039	14
11	2	-9	537.160	87.8844	14
-11	-2	9	561.985	87.0226	22
-11	-2	8	2832.10	228.394	22
11	-2	-8	3087.72	250.396	12
11	-2	-8	3651.85	264.164	14
11	2	-8	3084.58	232.402	14
11	-2	-8	3050.35	242.677	11
11	2	-8	3169.50	231.810	12
11	2	-8	3197.09	236.598	19
11	2	-7	57.7956	50.8807	12
11	-2	-7	85.4725	70.2015	14
11	2	-7	9.47725	49.1258	14
11	-2	-7	-48.2272	74.5359	12
11	2	-7	42.3704	55.9475	19
-11	-2	7	48.4972	51.2335	22
11	2	-6	30.8520	55.2961	12
11	-2	-6	-23.9261	67.6823	13
11	2	-6	9.72608	48.4171	14
11	-2	-6	-12.7772	73.4452	14
11	-2	-6	12.6031	74.6739	12
11	2	-6	-23.2605	61.4078	19
-11	-2	6	-20.1378	56.0398	22
-11	-2	5	1017.67	129.205	22
11	2	-5	1313.36	157.462	19
11	-2	-5	1292.35	159.165	13
11	2	-5	1197.80	134.554	14
11	-2	-5	1288.10	162.539	14
11	2	-4	8245.90	495.008	19

11	-2	-4	8711.53	504.083	13
11	2	-4	6660.21	446.668	14
11	-2	-4	8164.70	501.642	14
11	2	-4	7303.49	478.655	13
11	2	-3	2747.60	229.902	18
11	2	-3	2966.06	256.920	19
11	2	-3	3089.33	229.327	14
11	2	-3	2819.82	245.619	13
11	-2	-3	2872.64	253.292	14
11	2	-2	3726.88	272.004	13
11	-2	-2	3322.99	273.691	14
11	-2	-2	3668.04	276.118	6
11	2	-2	3287.44	262.991	18
11	2	-2	3521.66	281.168	19
11	2	-1	629.357	104.429	13
11	-2	-1	630.406	112.312	14
11	-2	-1	663.478	116.575	7
11	-2	-1	540.443	103.008	6
11	2	-1	609.585	114.528	26
11	-2	-1	468.193	106.279	26
11	2	-1	673.972	109.021	18
11	2	0	2979.16	240.403	18
11	-2	0	2782.49	233.965	14
11	2	0	2593.07	219.321	13
11	2	0	2705.36	240.723	26
11	-2	0	2817.52	241.619	26
11	-2	0	2956.25	236.855	6
11	-2	0	2958.40	240.678	7
11	2	1	85.4151	69.1570	26

11	-2	1	151.093	71.9551	26
11	-2	1	101.253	62.7181	14
11	-2	1	114.711	71.2019	6
11	2	1	67.9893	63.3288	6
11	-2	1	32.5326	46.1972	5
11	2	1	148.225	76.9396	18
11	2	2	12344.5	682.163	18
11	2	2	13169.6	706.618	26
11	-2	2	12613.8	694.424	26
11	-2	2	12700.5	679.480	14
11	-2	2	11117.3	658.505	5
11	2	2	12365.0	670.271	6
11	-2	3	179.260	56.1382	5
11	2	3	171.459	62.6666	26
11	-2	3	166.262	61.8784	26
11	2	3	212.657	67.2571	18
11	2	3	103.445	55.0333	7
11	-2	3	125.055	63.7166	6
11	-2	3	123.442	65.0884	7
11	2	3	61.9156	48.8523	6
11	-2	4	572.900	77.1817	5
11	2	4	385.178	73.1983	26
11	-2	4	456.667	73.2893	26
11	2	4	333.191	61.3563	7
11	-2	4	553.327	79.0807	7
11	-2	5	22.3695	30.8924	7
11	-2	5	48.4625	36.9009	5
11	2	5	60.7847	38.2200	18
11	-2	6	194.292	30.3853	5

11	2	6	88.4980	23.5767	5
11	-3	-11	344.738	46.5792	14
11	-3	-11	337.755	49.4749	12
11	-3	-10	402.744	67.9687	14
11	-3	-10	368.091	67.8977	12
-11	-3	10	293.321	55.2758	22
11	-3	-9	94.8320	54.7658	14
11	3	-9	76.0730	36.8893	14
11	-3	-9	60.4360	53.4240	12
-11	-3	9	-7.83599	45.1132	22
11	-3	-9	91.7661	51.6254	11
11	3	-9	49.4892	43.1779	19
-11	-3	8	1639.49	159.799	22
11	-3	-8	1828.42	157.180	13
11	3	-8	1515.12	134.862	14
11	-3	-8	1834.41	173.066	11
11	-3	-8	2016.57	178.254	12
11	3	-8	1523.54	157.493	19
-11	-3	7	3093.57	252.278	22
11	-3	-7	3718.49	257.897	13
11	3	-7	3165.02	226.068	14
11	-3	-7	3287.41	263.026	11
11	-3	-7	3717.55	270.069	12
11	3	-7	3464.65	262.796	19
-11	-3	6	2685.55	229.464	22
11	-3	-6	3017.07	235.458	13
11	-3	-6	2989.15	246.784	12
11	3	-6	2917.07	244.087	19
11	-3	-5	641.749	112.124	13

11	-3	-5	481.120	104.973	12
11	-3	-5	509.542	108.287	14
11	3	-5	422.589	100.639	13
11	3	-5	427.749	99.0765	19
-11	-3	5	572.288	100.965	22
11	3	-4	6195.40	398.885	19
11	-3	-4	5975.55	384.452	13
11	-3	-4	5905.35	393.174	14
11	3	-4	5872.10	375.670	13
-11	-3	4	4924.05	361.825	22
11	-3	-4	5460.66	353.239	6
11	-3	-3	34.5594	68.2819	13
11	3	-3	69.6970	52.6061	13
11	-3	-3	12.3306	63.9172	14
11	-3	-3	69.2543	54.2587	7
11	-3	-3	28.5119	53.8036	6
11	3	-3	10.2584	52.1547	18
-11	-3	3	51.3187	54.2262	22
11	3	-2	1182.54	133.148	13
11	-3	-2	1280.95	154.760	14
11	-3	-2	1463.18	160.055	13
11	3	-2	1098.79	141.245	18
-11	-3	2	1021.68	129.129	22
11	-3	-2	1031.94	130.795	7
11	-3	-2	1357.97	143.480	6
11	-3	-1	2507.72	207.255	6
11	-3	-1	2335.83	201.999	7
11	-3	-1	2599.58	220.766	14
11	-3	-1	2631.58	217.663	13

11	3	-1	2073.75	184.188	13
11	3	-1	2082.48	204.751	18
11	3	-1	2447.22	212.052	19
-11	-3	1	2103.30	187.288	22
11	3	-1	2252.65	206.393	26
11	-3	0	2193.01	191.410	6
11	-3	0	1978.45	185.589	7
11	-3	0	2277.38	202.209	14
11	3	0	2127.82	195.479	18
-11	-3	0	1853.00	167.342	22
11	3	0	2251.11	200.334	26
11	3	1	1085.33	135.444	18
11	-3	1	1167.48	140.162	14
11	-3	1	1492.21	147.060	6
11	-3	1	1378.56	144.759	7
11	3	1	1107.27	126.917	6
11	3	1	1297.47	144.278	26
11	-3	1	1119.50	134.803	26
11	-3	2	77.5000	50.6274	5
11	3	2	27.6888	53.8800	18
11	3	2	-14.2048	44.4940	6
11	-3	2	-17.4783	54.0204	6
11	3	2	9.74713	46.4609	26
11	-3	2	25.9970	44.0329	26
11	-3	3	909.032	99.7173	5
11	3	3	759.829	100.615	26
11	-3	3	883.378	98.8770	26
11	3	3	846.194	87.6510	6
11	-3	3	839.019	104.957	7

11	3	3	906.515	96.4963	7
11	-3	4	183.298	51.3158	7
11	3	4	138.762	35.2075	7
11	-3	4	104.024	40.9213	5
11	3	4	80.6033	44.4880	18
11	-3	5	575.990	65.9995	7
11	-3	5	773.575	72.1433	5
11	3	5	781.860	73.2100	18
-11	-4	10	1861.78	134.466	22
11	-4	-10	1931.87	133.304	14
11	-4	-10	1753.10	131.702	12
11	-4	-9	113.393	49.5093	14
11	-4	-9	93.4538	45.6819	12
-11	-4	9	63.9939	42.3243	22
11	-4	-9	136.059	57.1063	11
11	4	-9	53.5305	43.7250	19
11	-4	-8	93.0149	55.6906	14
11	-4	-8	32.8477	49.3731	12
-11	-4	8	8.72156	49.3499	22
11	-4	-8	-18.7624	53.9305	11
11	4	-8	-8.83281	53.1050	19
11	-4	-7	1160.79	134.117	14
11	-4	-7	1209.81	139.259	11
11	-4	-7	1071.81	126.842	12
11	4	-7	883.744	122.657	19
-11	-4	7	919.249	121.689	22
11	-4	-6	228.810	64.7509	13
11	-4	-6	161.068	64.3151	12
11	-4	-6	170.679	73.0955	14

11	4	-6	158.051	67.5043	13
11	4	-6	109.917	64.5933	19
-11	-4	6	125.039	64.8350	22
-11	-4	5	3937.95	284.827	22
11	-4	-5	3814.43	268.315	13
11	-4	-5	4399.37	299.406	14
11	4	-5	3396.71	259.818	13
11	-4	-4	356.028	83.2208	13
11	4	-4	326.930	72.5862	13
11	4	-4	380.097	82.4644	18
-11	-4	4	234.418	83.1604	22
11	4	-3	1136.58	120.935	13
11	-4	-3	1339.50	144.834	13
-11	-4	3	1290.13	145.472	22
11	4	-3	1385.64	158.565	19
11	4	-3	1138.20	138.851	18
11	4	-2	167.401	74.5614	19
11	-4	-2	130.166	65.3748	13
11	4	-2	56.7469	42.9887	13
11	-4	-2	139.992	58.3286	7
11	-4	-2	137.496	56.0060	6
-11	-4	2	139.132	61.6670	22
11	4	-2	116.257	65.5516	18
11	4	-1	-10.5812	60.8279	19
11	-4	-1	76.3937	53.1757	6
11	-4	-1	16.9247	49.4042	7
11	-4	-1	10.6822	58.4599	14
-11	-4	1	-9.27432	51.6241	22
11	4	-1	31.7349	62.6823	18

11	-4	0	152.958	60.6377	6
11	-4	0	60.3379	51.3019	7
11	-4	0	140.744	68.6979	14
-11	-4	0	125.691	58.5741	22
11	4	0	61.1772	62.3532	18
-11	-4	-1	428.763	74.0493	22
11	-4	1	454.393	89.9020	14
11	4	1	368.278	69.7987	6
11	-4	1	396.476	77.6909	6
11	-4	1	347.541	79.3490	7
11	4	1	433.578	89.2819	18
11	4	1	409.354	74.9538	26
11	4	2	639.111	74.2846	6
11	-4	2	595.716	88.1790	7
11	4	2	760.457	90.8411	26
11	4	3	314.762	67.0632	18
11	4	3	286.444	51.8864	7
11	4	4	3978.90	241.687	18
11	-4	4	4039.50	242.348	7
11	4	5	316.927	40.6316	18
11	-4	5	376.432	43.3863	7
-11	-5	9	835.880	90.3991	22
11	5	-9	970.983	93.2318	19
11	-5	-8	118.629	42.0855	14
11	5	-8	59.4172	30.6405	13
-11	-5	8	193.275	55.7547	22
11	-5	-8	170.785	59.2625	11
11	5	-8	148.154	56.0692	19
-11	-5	7	-17.3397	52.8675	22

11	-5	-7	-29.9043	48.0556	14
11	5	-7	-18.7192	34.9579	13
-11	-5	6	-47.1467	53.3616	22
11	-5	-6	-8.54151	49.1131	14
-11	-5	5	69.0183	58.7510	22
11	-5	-5	231.336	72.4922	14
11	5	-5	131.401	66.8540	19
11	-5	-4	3697.46	252.738	14
11	5	-4	2981.65	230.289	18
11	5	-4	2816.32	238.636	19
-11	-5	4	3094.80	240.696	22
11	-5	-3	7006.56	411.035	14
11	5	-3	6033.64	400.506	19
11	5	-3	6305.55	399.093	18
-11	-5	3	6475.68	403.731	22
11	-5	-2	4756.77	306.531	14
11	5	-2	4409.53	299.877	19
11	5	-2	4153.34	297.184	18
-11	-5	2	4067.59	291.914	22
11	5	-1	45.0833	54.2062	19
11	5	-1	-9.53611	53.9538	18
11	-5	-1	83.8123	59.8496	14
-11	-5	1	-35.0476	50.3670	22
11	5	0	185.732	50.0963	6
11	-5	0	161.592	53.1514	7
11	5	0	126.358	62.0352	18
-11	-5	0	197.827	63.3030	22
-11	-5	-1	279.792	64.1895	22
11	5	1	314.653	49.6476	6

11	-5	1	236.813	60.5176	7
11	-5	2	939.850	98.9252	7
11	5	2	1031.68	108.534	18
11	-5	3	378.902	58.7013	7
11	5	3	280.371	56.6519	18
11	5	4	2112.32	135.711	18
-11	-6	9	3.43521	20.3543	22
-11	-6	8	565.389	73.3084	22
11	6	-7	3479.31	238.159	19
-11	-6	7	3913.17	246.228	22
-11	-6	6	-23.4343	50.8251	22
11	6	-6	45.6480	46.3850	19
11	-6	-5	1052.88	112.128	14
11	6	-5	1123.24	121.451	19
-11	-6	5	1295.21	130.993	22
11	6	-4	22.4370	44.9709	18
11	-6	-4	7.21780	42.7769	14
11	6	-4	24.6653	47.2698	19
-11	-6	4	34.2513	50.7443	22
11	6	-3	394.872	78.5040	18
11	-6	-3	413.455	77.4453	14
11	6	-3	313.338	75.6851	19
11	-6	-2	5225.66	298.446	14
11	6	-2	4422.18	287.916	19
11	6	-2	4166.17	290.172	18
-11	-6	2	4239.82	290.765	22
11	-6	-1	249.166	59.9707	14
-11	-6	1	106.498	52.3091	22
11	6	-1	103.490	55.8837	18

-11	-6	0	1135.61	113.070	22
-11	-6	-1	1903.95	146.316	22
11	6	1	1920.84	153.008	18
11	6	2	969.037	92.4741	18
11	-6	2	861.681	79.5661	7
11	6	3	214.345	38.5494	18
11	-6	3	232.792	35.1755	7
11	7	-7	544.299	59.5647	19
-11	-7	7	542.594	63.0481	22
11	7	-6	3938.57	241.686	19
-11	-7	6	4123.57	248.920	22
-11	-7	5	330.457	62.5642	22
11	7	-5	355.223	58.3688	19
-11	-7	4	59.1011	43.8419	22
11	7	-4	60.2106	40.2018	18
11	7	-4	55.9910	39.1011	19
-11	-7	3	3317.19	221.879	22
11	7	-3	3324.96	211.331	19
11	7	-3	3222.91	215.050	18
11	7	-2	123.259	50.2697	18
-11	-7	2	203.029	54.8396	22
-11	-7	1	250.926	53.0238	22
-11	-7	0	573.888	69.9235	22
11	7	1	295.085	48.8509	18
11	7	2	28.3857	17.6404	18
-11	-8	5	12.8806	19.3675	22
-11	-8	4	543.269	59.2825	22
11	8	-3	530.004	54.5958	18
-11	-8	3	504.134	58.6653	22

11	8	-2	1035.83	83.7846	18
-11	-8	2	1113.30	88.0506	22
-11	-8	1	438.795	48.5882	22
12	0	-10	42.0224	27.3788	14
12	0	-10	17.4494	28.7628	13
12	0	-9	-18.6627	38.4607	14
12	0	-8	2155.18	174.026	14
12	0	-7	24.8800	43.7906	14
12	0	-7	-18.0051	57.1295	13
12	0	-6	3355.69	245.934	13
12	0	-6	3008.14	231.046	14
12	0	-5	9.98580	57.4128	13
12	0	-5	-44.8840	52.4143	14
12	0	-4	231.823	79.3843	13
12	0	-4	221.830	68.3047	14
12	0	-3	29.7786	59.6765	13
12	0	-3	8.53095	47.4861	14
12	0	-2	114.784	63.1439	13
12	0	-2	80.3170	50.5018	14
12	0	-1	18.0947	53.6107	13
12	0	-1	14.6466	40.7653	14
12	0	0	1396.92	129.060	14
12	0	0	1625.07	150.843	6
12	0	1	-15.1674	49.9437	6
12	0	1	-7.71944	49.6125	7
12	0	2	11454.5	617.134	6
12	0	2	11835.4	619.162	7
12	0	3	-9.57828	31.5410	7
12	0	4	211.191	31.6969	7

12	0	4	169.230	28.7585	5
12	-1	-10	41.9003	25.1309	13
12	1	-10	29.4137	19.6535	14
12	-1	-10	13.2314	27.2998	14
12	1	-10	43.8792	33.6942	13
12	-1	-9	265.757	60.7786	12
12	-1	-9	264.013	56.7536	13
12	1	-9	212.218	45.7915	14
12	-1	-9	174.565	54.2400	14
12	1	-9	136.615	50.4033	13
12	-1	-8	94.3984	50.7433	12
12	-1	-8	82.4648	54.0424	13
12	1	-8	114.679	45.0641	14
12	-1	-8	109.008	52.6630	14
12	1	-8	55.0053	52.5408	13
12	-1	-7	4080.79	263.264	13
12	1	-7	3099.31	232.935	14
12	-1	-7	3824.67	257.763	14
12	1	-7	3500.57	253.371	13
12	1	-6	3429.09	241.996	14
12	-1	-6	3617.27	258.195	14
12	1	-6	3631.40	260.556	13
12	1	-5	112.529	49.8287	19
12	1	-5	48.0124	57.0244	13
12	1	-5	116.186	50.5431	14
12	-1	-5	38.1215	52.2919	14
12	1	-4	764.945	106.956	19
12	1	-4	872.940	103.409	14
12	1	-4	826.318	117.997	13

12	-1	-4	863.727	115.519	14
12	1	-3	-36.0848	49.3666	19
12	-1	-3	9.93827	56.2285	13
12	1	-3	-18.7839	56.4618	13
12	1	-3	14.4067	41.4084	14
12	-1	-3	-9.22827	57.0408	14
12	-1	-2	202.800	70.5643	13
12	1	-2	188.422	71.6859	13
12	1	-2	204.883	53.5222	14
12	-1	-2	113.957	59.6601	14
12	-1	-2	210.177	69.9904	7
12	-1	-2	145.993	61.3583	6
12	-1	-1	2984.28	213.043	14
12	1	-1	2821.86	212.541	13
12	1	-1	2505.59	186.379	14
12	-1	-1	2849.46	216.763	6
12	-1	-1	2750.31	216.412	7
12	1	-1	3043.21	224.146	6
12	-1	0	3375.93	238.788	7
12	1	0	3397.25	236.973	6
12	-1	0	3280.64	225.159	14
12	1	0	2924.31	237.202	26
12	1	1	445.114	81.0843	26
12	-1	1	436.654	77.1520	26
12	1	1	504.937	81.0234	6
12	1	1	398.958	78.2001	7
12	1	1	448.211	69.8864	18
12	1	2	203.544	58.1516	26
12	-1	2	265.652	59.8662	26

12	1	2	262.514	54.9648	18
12	1	2	221.786	55.2657	7
12	-1	2	320.343	62.6609	6
12	1	2	248.819	56.2325	6
12	-1	2	368.044	66.9194	7
12	-1	3	393.012	57.9258	7
12	1	3	394.534	56.2205	7
12	-1	4	19.5647	18.8462	7
12	1	4	5.18370	16.2436	5
12	-2	-10	26.4677	24.6351	14
12	2	-10	28.1831	23.2349	13
12	-2	-9	4.37238	29.4771	13
12	-2	-9	17.9148	37.4100	14
12	2	-9	22.3726	38.9678	13
12	-2	-8	2091.56	156.386	13
12	2	-8	1862.82	136.015	14
12	-2	-8	2325.41	172.379	14
12	2	-8	1886.94	160.548	13
12	-2	-7	3098.23	214.299	13
12	2	-7	2607.55	184.001	14
12	-2	-7	2992.63	218.853	14
12	2	-7	2781.52	209.034	13
12	-2	-6	14051.6	686.063	13
12	2	-6	11005.6	636.761	14
12	-2	-6	13184.5	686.401	14
12	2	-6	11945.5	670.251	13
12	2	-6	12918.3	667.535	19
12	-2	-5	4667.57	290.909	13
12	2	-5	4115.46	278.189	13

12	-2	-5	4578.51	292.635	14
12	2	-5	4177.82	278.369	19
12	-2	-4	445.929	75.4963	6
12	-2	-4	393.825	88.7332	13
12	2	-4	325.422	76.5919	13
12	-2	-4	351.679	82.3097	14
12	2	-4	440.636	84.2720	19
12	-2	-3	809.980	103.171	6
12	-2	-3	797.912	98.5763	7
12	-2	-3	1016.48	122.267	14
12	2	-3	731.260	102.987	13
12	2	-3	798.176	109.527	19
12	-2	-2	806.652	102.416	7
12	-2	-2	1128.26	119.439	6
12	-2	-2	849.796	113.872	14
12	2	-2	815.396	104.564	13
12	-2	-1	7158.21	417.211	6
12	-2	-1	6778.37	410.509	7
12	-2	-1	7130.72	418.025	14
12	-2	0	312.651	69.9886	7
12	2	0	286.293	67.4589	6
12	-2	0	224.102	64.5210	6
12	-2	0	242.645	64.2860	14
12	2	0	267.382	61.6043	18
12	2	1	166.556	52.1161	6
12	-2	1	280.376	62.4002	6
12	-2	1	248.993	63.1756	7
12	2	1	217.533	58.6533	26
12	2	1	133.816	52.2530	18

12	2	2	98.6008	43.7683	7
12	2	2	104.534	40.4669	6
12	2	2	88.4524	43.4736	26
12	2	2	143.025	45.0045	18
12	-2	3	451.492	59.1421	7
12	2	3	355.802	49.0397	7
12	2	3	392.439	54.5172	18
12	-2	4	107.115	23.8747	7
12	3	-9	30.8818	25.1581	13
12	3	-8	133.556	41.8161	13
12	-3	-7	1302.00	117.868	13
12	3	-7	1286.83	117.197	13
12	3	-7	1125.82	114.895	19
12	-3	-6	111.809	51.7416	13
12	3	-6	128.549	51.5247	13
12	-3	-6	192.001	61.6188	14
12	3	-6	230.009	60.3012	19
12	3	-5	719.506	92.9057	13
12	-3	-5	721.896	103.772	14
12	-3	-5	920.565	106.759	13
12	3	-5	759.252	103.294	19
12	3	-4	2457.26	188.733	13
12	-3	-4	2784.94	212.898	14
12	-3	-4	2909.48	209.239	13
12	3	-4	2505.51	205.144	19
12	-3	-3	1632.48	155.018	14
12	-3	-3	1754.09	153.851	13
12	3	-3	1971.53	160.815	19
12	3	-2	274.094	60.5475	13

12	-3	-2	410.896	84.2834	14
12	-3	-2	305.128	66.3020	6
12	-3	-1	738.359	100.740	14
12	-3	-1	748.822	92.9729	6
12	-3	-1	707.276	90.2181	7
12	3	-1	799.486	94.8625	18
12	-3	0	878.497	103.890	14
12	-3	0	711.404	91.0805	6
12	-3	0	849.025	96.1660	7
12	3	0	778.788	92.7029	6
12	3	0	845.981	97.0789	18
12	3	1	408.537	61.5243	6
12	-3	1	313.598	60.1640	7
12	3	1	305.088	59.8609	18
12	3	2	1566.77	114.349	6
12	-3	2	1913.14	135.093	7
12	3	2	1776.74	127.986	7
12	3	2	1798.67	132.416	18
12	3	3	2395.78	151.266	18
12	-4	-8	997.587	87.3706	14
12	4	-8	833.801	79.0076	19
12	4	-7	44.4379	26.1212	13
12	-4	-7	66.2677	41.0968	14
12	4	-7	41.5497	37.7015	19
-12	-4	7	8.78552	26.4576	22
12	4	-6	902.092	86.4182	13
-12	-4	6	952.389	93.7844	22
12	4	-5	619.900	70.7783	13
12	4	-5	792.053	98.8819	19

-12	-4	5	830.200	87.7190	22
12	4	-4	1261.18	111.412	13
-12	-4	4	2026.42	139.744	22
-12	-4	3	48.9078	36.7000	22
-12	-4	2	9.68070	29.9427	22
12	-4	-2	45.7046	48.9693	14
12	4	-2	52.8330	49.7179	19
12	4	-1	237.444	51.6781	6
12	-4	-1	194.228	59.3936	14
12	4	-1	224.648	54.7987	18
12	-4	0	1581.52	132.234	14
12	-4	0	1325.95	113.906	7
12	4	0	1511.70	113.995	6
12	4	0	1334.77	121.812	18
12	-4	1	49.6458	33.1132	7
12	4	1	33.1094	22.9494	6
12	4	1	25.6864	36.5798	18
12	4	2	5.18375	18.8133	7
12	-4	2	7.36970	26.7431	7
12	4	2	20.0705	26.7503	18
12	-5	-7	7.29037	21.5990	14
12	5	-7	18.0301	26.7081	19
-12	-5	7	-3.32653	21.3795	22
12	-5	-6	-14.6717	33.3368	14
-12	-5	6	4.40540	26.8596	22
12	-5	-5	2138.74	150.347	14
-12	-5	5	1728.83	135.147	22
12	-5	-4	85.2784	44.4976	14
12	5	-4	102.100	50.4865	19

-12	-5	4	132.946	41.2306	22
12	5	-3	3158.26	203.285	19
12	-5	-3	3234.59	204.761	14
-12	-5	2	805.710	80.6876	22
12	5	-2	907.747	91.1544	18
12	5	-1	265.095	42.9599	6
12	5	-1	338.345	54.8290	18
-12	-5	1	189.874	35.7603	22
12	5	0	120.901	39.6592	18
12	5	1	945.790	73.3358	7
12	5	1	1281.91	92.0236	18
12	6	-6	138.462	31.5447	19
-12	-6	5	44.0821	23.6390	22
12	6	-5	-4.11818	24.7727	19
12	-6	-4	114.577	31.2215	14
-12	-6	4	99.4577	30.6508	22
12	6	-4	135.166	36.9272	19
-12	-6	3	86.1807	29.0680	22
12	-6	-3	103.094	31.5908	14
12	6	-2	34.7759	26.3508	18
-12	-6	2	80.6193	25.5510	22
12	6	-1	487.751	52.6274	18
12	6	0	-5.50750	18.1360	18
13	0	-7	2234.38	141.157	13
13	0	-7	1513.32	115.665	14
13	0	-6	17.0067	38.2118	13
13	0	-6	4.28276	29.1958	14
13	0	-5	4102.41	240.185	6
13	0	-5	3593.05	230.359	14

13	0	-5	4290.15	253.166	13
13	0	-4	-5.66210	39.0238	6
13	0	-4	-12.4644	40.0538	13
13	0	-4	-9.06078	29.8367	14
13	0	-3	1071.21	98.0292	7
13	0	-3	1216.80	109.267	6
13	0	-3	1023.57	91.4927	14
13	0	-3	1133.03	108.199	13
13	0	-2	-5.30781	34.5399	7
13	0	-2	-17.2924	38.4123	6
13	0	-2	-14.4664	23.5386	14
13	0	-1	625.735	75.1465	7
13	0	0	-9.29723	31.3358	6
13	0	1	1216.50	93.7313	6
13	0	1	1323.56	95.5272	7
13	1	-8	407.921	48.9081	13
13	-1	-8	416.328	46.5812	14
13	-1	-8	456.776	49.4568	13
13	1	-7	1001.55	89.0177	13
13	-1	-7	905.320	83.4538	14
13	-1	-7	1043.49	89.5163	13
13	1	-6	195.569	48.4468	13
13	-1	-6	94.0137	38.8018	14
13	-1	-5	1261.44	106.220	14
13	1	-5	1032.21	102.494	13
13	1	-4	504.651	72.2863	6
13	-1	-4	603.394	69.1029	6
13	-1	-4	500.664	73.4043	13
13	1	-4	343.549	62.5752	13

13	-1	-4	457.297	64.3892	14
13	-1	-3	84.3658	40.5482	6
13	-1	-3	40.8131	35.2705	14
13	-1	-2	419.418	58.7982	7
13	-1	-2	462.052	65.6515	6
13	1	-2	399.816	64.7010	7
13	1	-2	463.683	68.3436	6
13	-1	-2	412.469	58.8898	14
13	-1	-1	1452.66	115.423	6
13	1	-1	1467.25	119.322	6
13	-1	-1	1340.73	109.767	7
13	1	0	26.7910	30.1235	7
13	-1	0	8.32595	29.6385	7
13	1	0	48.2195	30.8868	6
13	1	1	201.858	34.8194	6
13	1	1	259.858	40.1822	7
13	-2	-8	1924.85	119.607	14
13	-2	-7	200.669	36.2912	13
13	2	-7	136.288	33.8620	13
13	-2	-7	239.251	43.0040	14
13	-2	-6	1244.75	100.906	14
13	-2	-6	1314.35	99.9433	13
13	-2	-5	177.107	47.0326	13
13	2	-5	136.267	38.4980	13
13	-2	-5	169.315	46.2172	14
13	2	-4	266.088	49.5469	13
13	-2	-4	433.246	62.9522	14
13	2	-3	830.377	87.9193	6
13	-2	-3	685.382	80.0568	14

```
 13   2  -2-5.21347 35.1402   6
 13  -2  -2 4.26526 27.7517   6
 13  -2  -2 4.88354 34.0201  14
 13   2  -1 324.848 51.5216   6
 13  -2  -1 325.029 50.2321   6
 13   2   0 1047.52 86.9426   6
 13  -2   0 1038.28 83.5492   7
 13   2   0 1221.20 93.5671   7
 13  -2   1 2.51319 16.5504   7
 13   2   1-7.89770 18.3394   7
 13  -3  -7 1045.56 80.5852  14
 13  -3  -6 878.218 79.8458  14
 13  -3  -5 149.953 41.0148  14
 13  -3  -4 229.720 48.5942  14
 13   3  -3 148.157 40.7460   6
 13  -3  -3 135.307 41.3303  14
 13   3  -2 8.36808 28.5201   6
 13  -3  -2-9.03922 31.4847  14
 13   3  -1 2974.12 180.668   6
 13   3  -1 3255.90 189.855   7
 13   3   0 415.706 46.7436   7
 13   4  -4 261.037 40.6710   6
 13   4  -3-6.16820 20.7876   6
 13  -4  -3 7.51779 24.7534  14
 13   4  -1-15.4051 17.3208   7
  0   0   0    0.00    0.00   0
```

TITL JS323F_RM in P2(1)/n

REM P2(1)/n (#14 with fixed user cell!)

```
CELL 1.54184  10.810839  10.714739  12.272760  90.0000 103.2179  90.0000
ZERR    3.00   0.000612   0.000632   0.000854   0.0000   0.0064   0.0000
LATT  1
SYMM -x+1/2, y+1/2,-z+1/2
SFAC C H N O
UNIT 15.00 3.00 27.00 42.00
REM  CrysAlisPro recorded range (K): Min=297.9; max=298.0; aver:298.0
TEMP 25
SIZE 0.05 0.07 0.22
TREF
HKLF 4
END
;
_shelx_hkl_checksum              99593
_olex2_diffrn_ambient_temperature_device  'Oxford Cryosystems'
_olex2_exptl_crystal_mounting_method
 'The crystal was mounted on a nylon loop with paratone oil'
_olex2_submission_original_sample_id  'JS167K V1 Room Temperature'
_olex2_submission_special_instructions  'No special instructions were received'
_oxdiff_exptl_absorpt_empirical_details
;
Empirical correction (ABSPACK) includes:
- Absorption correction using spherical harmonics
- Frame scaling
;
_oxdiff_exptl_absorpt_empirical_full_max  1.481
_oxdiff_exptl_absorpt_empirical_full_min  0.802
```
